# New hypothesis: a gut-lipid-kidney axis in constipated CKD patients—insights from multi-omics triangulation

**DOI:** 10.1128/msphere.00914-25

**Published:** 2026-02-05

**Authors:** Yichen Liu, Jin Zhao, Jinguo Yuan, Zixian Yu, Jie Liu, Xiaoxuan Ning, Shiren Sun

**Affiliations:** 1 Department of Postgraduate Student, Xi’an Medical University, Xi’an, China; 2 Department of Nephrology, Xijing Hospital, Fourth Military Medical University, Xi’an, China; 3 Department of Geriatrics, Xijing Hospital, Fourth Military Medical University, Xi’an, China; University of Michigan-Ann Arbor, Ann Arbor, Michigan, USA

**Keywords:** chronic kidney disease, constipation, all-cause mortality, gut microbiota, phosphatidylcholine, PPARγ

## Abstract

Patients with chronic kidney disease (CKD) frequently experience constipation, often linked to gut microbiota disruptions. We hypothesized that constipation is not merely a comorbidity but a driver of adverse outcomes through a specific host-microbial metabolic axis. Employing a multi-cohort design for evidence triangulation, we first employed a large U.S. database and mortality data up to 31 December 2019 to investigate the association between constipation and overall mortality in patients with CKD using weighted Cox proportional hazards models. To explore potential microbial and metabolic mechanisms that could underlie such clinical observations, we then used genetic data from the independent FINRISK cohort to investigate causal relationships between specific gut microbiota, their metabolites, and CKD. In the observational cohort, we identified functional constipation as a potent, independent predictor of both all-cause (HR: 1.33; 95% CI: 1.11–1.58) and cardiovascular mortality. This association remained robust across sensitivity analyses and showed a distinct U-shaped dose-response relationship with stool frequency. In the causal inference phase, while the direct causal effect of constipation on CKD was not significant, MR identified a significant causal depletion of the genus *Herbidospora* driven by constipation. Further analyses revealed that *Herbidospora* exerts a protective effect against CKD. Crucially, mediation analysis demonstrated that phosphatidylcholine (14:0_18:2) [PC 14:0_18:2] metabolites mediate 12.5% of the protective pathway from *Herbidospora* to CKD. Constipation is a key and independent predictor of mortality in patients with CKD. We found that constipation leads to a reduction in the number of *Herbidospora*, thereby disrupting the protective function of the kidney. This is because PC (14:0_18:2) is lacking. This study emphasizes the importance of restoring this microbial metabolic axis as a novel therapeutic strategy for CKD.

**IMPORTANCE:**

For millions living with chronic kidney disease (CKD), a common issue such as constipation can be a hidden danger, increasing their mortality risk by over one-third. Our research uncovers why: an unhealthy gut, often indicated by constipation, lacks specific “good”" bacteria essential for producing a protective fat molecule. This natural molecule acts as a key, activating the kidney’s own defense and repair systems. This discovery of a “gut-lipid-kidney” connection offers a groundbreaking new strategy: therapies aimed at restoring gut health and supplementing this key protective fat could provide a powerful new way to slow disease progression and improve survival in CKD patients.

## INTRODUCTION

Chronic kidney disease (CKD) is emerging as a global public health issue, affecting approximately 9.1% of the population, particularly as aging populations grow (1). It involves a gradual decline in kidney function, leading to impaired waste and water removal from the body. As the disease progresses, it can result in various complications that substantially affect the quality of life of patients. Constipation is one of the most common gastrointestinal issues in CKD. Renal dysfunction can cause toxin accumulation, thereby disturbing gastrointestinal function. On the other hand, the use of medications used to treat CKD and dietary fiber restriction can exacerbate constipation (2, 3). Beyond symptom discomfort, constipation also initiates a “gut‒kidney vicious cycle” by increasing intra-abdominal pressure and renal vascular resistance (4). Despite its clinical relevance, the significance of constipation as a CKD complication is often overlooked by clinicians and patients (5), which may stem from the limited research on the impact of constipation on CKD patient outcomes.

Gut microbiota dysbiosis may be a vital link between CKD and its associated constipation. In CKD, the progressive accumulation of uremic waste products compromises intestinal barrier integrity (6), facilitating the translocation of bacterial metabolites and pro-inflammatory molecules across the intestinal–kidney axis, thereby aggravating renal injury (7). Microbial metabolic disturbances impair intestinal neuromuscular function, slow transit time, and contribute directly to constipation (8). Among the altered metabolites, indole sulfate (IS) and trimethylamine N-oxide (TMAO) are particularly noteworthy: both accumulate due to reduced renal clearance, yet also exert pathogenic effects by promoting oxidative stress, endothelial dysfunction, and nephrotoxicity (9, 10). In particular, TMAO elevation is strongly linked to CKD progression, while cardiovascular disease remains the leading cause of mortality in CKD patients (11, 12). Emerging evidence further suggests that gut-derived metabolites can modulate enteric nervous system activity and gut motility (13), raising a critical research question: could specific microbiota-driven metabolites simultaneously regulate gastrointestinal function and renal pathology, thereby influencing the clinical trajectory of CKD with constipation?

Lipid metabolism disorders are common in patients with CKD and may interact with gut microbiota in complex ways. Lipid molecules, such as phosphatidylcholines (PCs), are metabolized by the gut microbiota (e.g., *Clostridium* spp.) to produce TMAO, which accelerates renal fibrosis and cardiovascular complications (14–16). However, the protective roles of specific PC subtypes and their underlying mechanism remain unclear. This scientific gap provides an opportunity to explore whether unique gut microbiota–derived lipid metabolites could mitigate CKD progression rather than exacerbate it.

To explore these unresolved questions, we employed a multi-cohort design to triangulate evidence from observational and genetic data. Our objectives were threefold: (i) to determine whether constipation independently predicts all-cause and cardiovascular mortality in a large CKD cohort ; (ii) to investigate the causal chain linking constipation, gut microbiota dysbiosis, and lipid metabolism using Mendelian randomization (MR), specifically focusing on whether constipation drives the depletion of renoprotective taxa and metabolites; and (iii) to provide biological plausibility for our epidemiological findings, we performed an exploratory computational analysis to generate a hypothesis regarding the potential molecular interactions between specific microbial metabolites and host lipid signaling. By integrating population-level evidence with genetic causality, our work aims to characterize a previously underexplored “gut-lipid-kidney” axis, offering a novel perspective on the non-renal drivers of CKD mortality.

## MATERIALS AND METHODS

### Overall study design

This study employed a multi-disciplinary design to investigate a potential gut-lipid-kidney axis.

Stage 1 was clinical association. We first assessed the clinical relevance of gut health in a large U.S. cohort. NHANES data—retrospectively accessed for this study—are overseen by the NCHS/CDC. Ethical clearance from the NCHS Ethics Review Committee and participants’ signed informed consent documents were obtained prior to data gathering. All procedures complied with institutional and federal research regulations (17, 18).

In stage 2, to overcome the inherent limitations of observational studies, such as residual confounding and reverse causation, we employed a two-sample MR design. We identified specific types of gut bacteria and lipid metabolites that have a causal relationship with CKD. Subsequently, we investigated whether constipation affects the abundance of these bacteria and quantified the mediating role of lipids in this microbial pathway. Valid MR analysis requires IVs to (i) strongly associate with the gut microbiota, (ii) show no pleiotropy (no confounding pathways), and (iii) affect outcomes solely via microbiota exposure (19).

### Study participants

**Fig 1:**
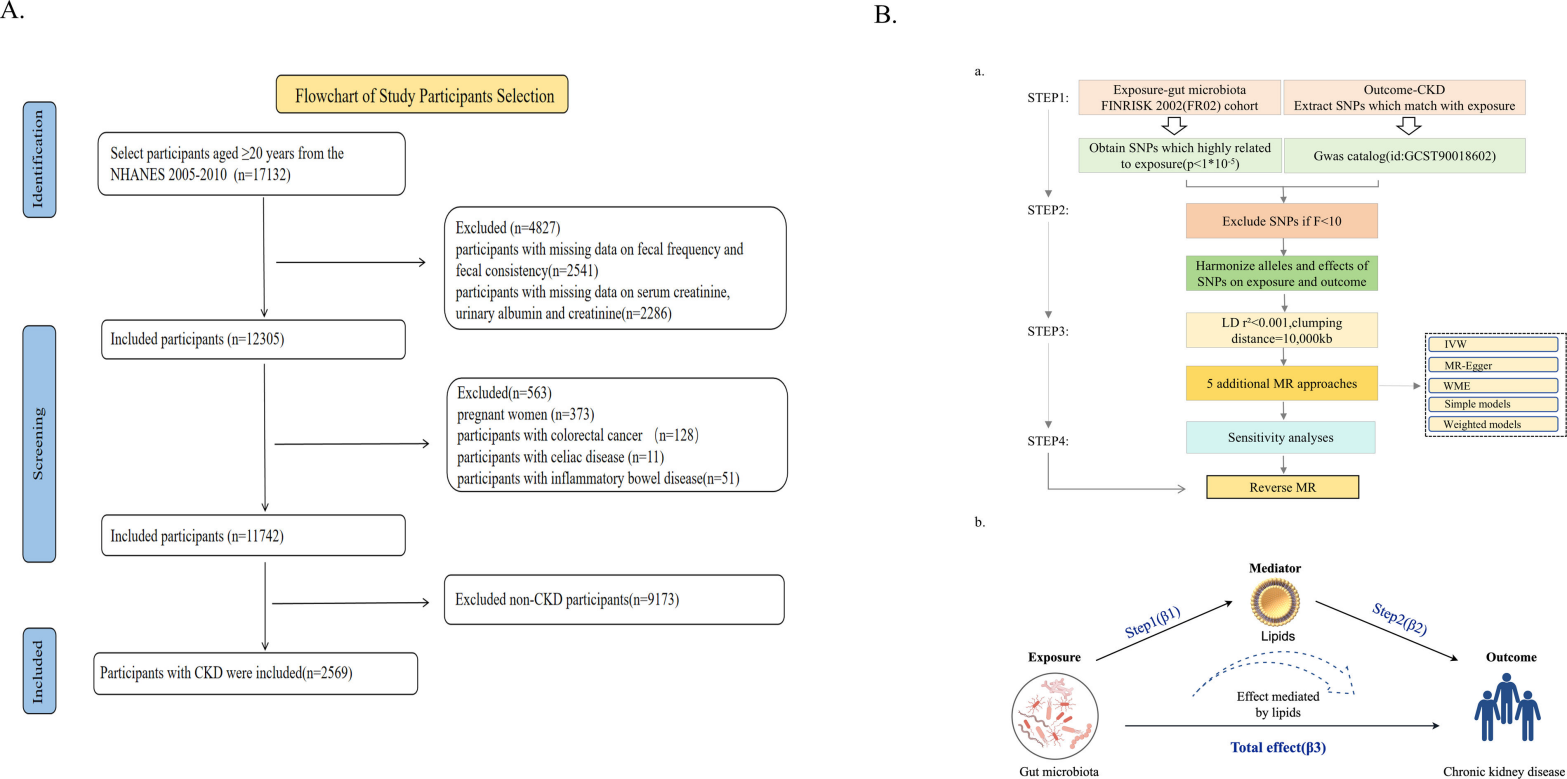
Study flowchart of participant selection and methodological framework for multi-omics causal inference. (**A**) Flowchart illustrating the inclusion and exclusion criteria for the clinical epidemiological study based on the National Health and Nutrition Examination Survey (NHANES) 2005–2010. Abbreviations: NHANES, National Health and Nutritional Examination Survey; CKD, chronic kidney disease. (**B**) Schematic overview of the Mendelian randomization (MR) design. (**A**) Illustration of the study design and workflow. (**B**) Mediated Mendelian randomization assessment detailing the effect of the gut microbiota on CKD via lipid composition. C, total effect; A*B, indirect effect; C', direct effect. Abbreviations: SNPs, single nucleotide polymorphisms.

The participants were drawn from the NHANES database from 2005 to 2010 because data on constipation were recorded over three 2-year cycles (2005–2006, 2007–2008, and 2009–2010). The study included patients who satisfied the following criteria: (i) aged >20 years; (ii) had relevant defecation data; and (iii) had serum creatinine, urine albumin (ALB), and urine creatinine data. One of these conditions was excluded: (i) pregnant woman; (ii) self-reported celiac disease, colon cancer, or inflammatory bowel conditions; and (iii) variables with missing values that could not be interpolated (missing data >15%). The data analysis included 2,569 participants (Fig. 1A).

### CKD definition

The criteria for CKD were as follows: (i) estimated glomerular filtration rate (eGFR) < 60 mL/min/1.73 m^2^; (ii) urine albumin-to-creatinine ratio (UACR) ≥ 30 mg/g; and (iii) self-reported CKD. A person is defined as having CKD if any one of these criteria is met. The eGFR is derived through the CKD-EPI equation (20).

### Definition of constipation

Constipation was defined by the frequency of feces and fecal traits according to the NHANES database (21). Using the Bristol Stool Form Scale (BSFS), the rate of transport through the colon was assessed, and participants were asked to identify their typical stool type based on visual cues. Constipation was defined as Type 1 (separate hard lumps) or Type 2 (lumpy sausage) (22). The participants reported their bowel movement frequency: “How often do you have a bowel movement?” Those with fewer than three weekly occurrences were classified as constipated.

### Data extraction

To minimize potential bias arising from subjective variable selection and to prevent model overfitting, we employed the least absolute shrinkage and selection operator (LASSO) regression algorithm to identify the most significant covariates for the final models (23). An extensive pool of candidate variables was initially screened, encompassing demographics (age, sex, education level, race, poverty income ratio, and marital status), lifestyle factors and medication history (specifically smoking status and opioid use), and major comorbidities (diabetes mellitus, hypertension, congestive heart failure, and history of dialysis). Additionally, clinical and laboratory parameters, including body mass index (BMI), alanine aminotransferase (ALT), aspartate aminotransferase (AST), blood urea nitrogen (BUN), eGFR, glycated hemoglobin (HbA1c), and serum ALB, were evaluated. The penalty parameter (λ) was determined via 10-fold cross-validation, and only variables with non-zero coefficients at the optimal λ were retained for adjustment in the final multivariable Cox proportional hazards models and propensity score matching (PSM) analysis (selection process visualized in Fig. S2).

### Missing value treatment

For variables with < 5% missing data, we applied the mice package in R to perform multiple imputation using chained equations (MICE) (Fig. S1) (24).

### MR data sources

Constipation statistics were obtained from the FinnGen consortium via the OpenGWAS database (data set ID: finn-b-K11_CONSTIPATION). This data set includes 17,246 cases and 201,546 controls of European ancestry (total *N* = 218,792). The phenotype was defined based on electronic health records, with associations adjusted for age, sex, genotyping batch, and principal components. Gut microbiota summary statistics were obtained from the FINRISK 2002 cohort (FR02), comprising 5,959 participants. Notably, the microbiome was profiled using shallow shotgun metagenomic sequencing rather than 16S rRNA methods, allowing for species-level resolution. In the source GWAS, microbial abundances underwent centered log-ratio (CLR) transformation, and associations were adjusted for age, sex, genotyping batch, and the first 10 genomic principal components (25). For the lipid mediator analysis, we utilized data from the GeneRISK cohort (*N* = 7,174), representing the Finnish population. Circulating plasma lipid levels were quantified via high-throughput mass spectrometry-based shotgun lipidomics. The source study adjusted for potential confounders, including age, sex, collection site, lipid medication status, and ancestry, followed by inverse-normal transformation of residuals (26). CKD outcomes were sourced from the Genome-Wide Association Study (GWAS) repository (ID: ebi-a-GCST90018602; 2,117 cases and 174,345 controls).

We initially identified single nucleotide polymorphisms (SNPs) through GWAS with a threshold of *P* < 1 × 10^−5^, a genetic span of 10,000 kb, and a linkage disequilibrium (LD) coefficient *r*^2^ < 0.001. To reduce bias for weak IVs, we set *F* < 10 to be excluded (27). For complete details, please refer to Fig. 1B. The remaining relevant documents are listed in the Supplementary Materials (Tables S6 to S10).

### Statistical analysis

Considering NHANES’s intricate multistage sampling strategy, the analyses were adjusted using weights (1/3 × WTSAF2YR). Continuous variables are presented as the means ± SD, whereas categorical variables are shown as frequencies (%). Comparisons between groups were performed via *t*-tests or ANOVA for continuous data and χ² tests for categorical data. Baseline characteristics were compared using weighted Student’s *t*-tests for continuous variables and weighted χ tests for categorical variables.

To robustly evaluate the association between constipation and mortality, we constructed three progressive multivariable Cox proportional hazards models. Model 1 was an unadjusted model. Model 2 adjusted for demographic and socioeconomic factors, including age, sex, race, BMI, education level, family income to poverty ratio (PIR), and smoking status. Model 3 (the fully adjusted model) further controlled for dietary factors (dietary fiber and water intake), clinical comorbidities (hypertension, diabetes, history of CVD, dialysis status), medication history (opioid use), and laboratory biomarkers (glycohemoglobin [HbA1c], ALB, BUN, and eGFR). The proportional hazards assumption was verified using Schoenfeld residuals.

To further strengthen causal inference in this observational setting, three sensitivity analyses were performed: (i) PSM was employed to balance baseline characteristics between constipated and non-constipated groups (1:1 matching, caliper 0.05); (ii) a Fine-Gray competing risk model was used to estimate sub-distribution hazard ratios (SHRs) for cardiovascular mortality, accounting for non-CVD death as a competing event; and (iii) a restricted cubic spline (RCS) analysis was conducted to visualize the dose-response relationship between stool frequency/consistency and mortality risk.

### Mendelian randomization and mediation analysis

Two-sample MR was conducted to investigate the causal pathway from gut microbiota to CKD outcomes, mediated by lipid traits. Instrumental variables (IVs) for gut microbiota and lipid metabolites were selected from large-scale GWAS summary statistics. The primary causal estimate was derived using the IVW method. To ensure robustness, we performed extensive sensitivity analyses, including MR-Egger regression (to detect pleiotropy), weighted median, and MR-PRESSO (to correct for outliers). Heterogeneity was assessed using Cochran’s Q statistic. Finally, mediation analysis was performed to quantify the proportion of the total effect of gut dysbiosis on CKD risk that is mediated by specific lipid metabolites. All analyses were conducted using R software (version 4.4.2). Two-sided *P*-values <0.05 were considered statistically significant (28, 29).

## RESULTS

### Baseline characteristics of participants with CKD

A total of 2,569 participants with CKD were included in the final analysis, among whom 278 (10.8%) reported constipation. In the unmatched cohort, patients with constipation were significantly more likely to be female (*P <* 0.001) and from lower socioeconomic backgrounds, as evidenced by lower educational attainment (*P =* 0.029) and a lower PIR (*P =* 0.004). Clinically, the constipation group presented with significantly lower serum ALB levels compared to the non-constipated group (*P <* 0.001). Significant differences were also observed in racial distribution and smoking status (*P <* 0.05). Notably, no significant differences were found in age, BMI, eGFR, BUN, or the prevalence of comorbidities—including hypertension, diabetes, heart failure, dialysis status, and opioid Use—between the two groups (all *P >* 0.05). Following PSM (1:1), 278 pairs were generated. All baseline characteristics were well-balanced between the constipated and non-constipated groups, with all standardized mean differences (SMD) < 0.10 and *P >* 0.05 (Table 1).

**TABLE 1:** Baseline characteristics of the study population before and after propensity score matching^*a*^

Characteristic		Unmatched cohort				PSM matched cohort (1:1)			
		No constipation	Constipation	*P* value	SMD	No constipation	Constipation	*P* value	SMD
*n*		2,291	278			278	278		
Age (mean [SD])		63.52 (16.44)	63.37 (16.67)	0.881	0.009	63.04 (16.81)	63.37 (16.67)	0.818	0.020
Sex (%)									
Male		1,093 (47.7)	70 (25.2)	<0.001	0.481	69 (24.8)	70 (25.2)	1.000	0.008
Female		1,198 (52.3)	208 (74.8)			209 (75.2)	208 (74.8)		
Race (%)									
Mexican American		332 (14.5)	25 (9.0)	0.022	0.220	25 (9.0)	25 (9.0)	0.901	0.087
Other Hispanic		152 (6.6)	24 (8.6)			26 (9.4)	24 (8.6)		
Non-Hispanic White		1,291 (56.4)	149 (53.6)			157 (56.5)	149 (53.6)		
Non-Hispanic Black		458 (20.0)	70 (25.2)			60 (21.6)	70 (25.2)		
Other race		58 (2.5)	10 (3.6)			10 (3.6)	10 (3.6)		
BMI (mean [SD])		30.13 (7.29)	29.60 (7.34)	0.256	0.072	29.34 (7.13)	29.60 (7.34)	0.671	0.036
HbA1c (mean [SD])		6.44 (1.58)	6.41 (1.78)	0.800	0.015	6.46 (1.57)	6.41 (1.78)	0.718	0.031
ALB (mean [SD])		41.23 (3.60)	40.40 (3.99)	<0.001	0.221	40.26 (4.33)	40.40 (3.99)	0.707	0.032
BUN (mean [SD])		17.87 (9.47)	17.92 (9.70)	0.939	0.005	18.21 (10.74)	17.92 (9.70)	0.740	0.028
eGFR (mean [SD])		70.17 (28.54)	67.18 (29.68)	0.101	0.103	68.43 (30.57)	67.18 (29.68)	0.626	0.041
Marital status (%)									
Married		1,182 (51.6)	133 (47.8)	0.892	0.082	137 (49.3)	133 (47.8)	0.929	0.099
Widowed		463 (20.2)	62 (22.3)			55 (19.8)	62 (22.3)		
Divorced		277 (12.1)	36 (12.9)			43 (15.5)	36 (12.9)		
Separated		71 (3.1)	8 (2.9)			8 (2.9)	8 (2.9)		
Never married		200 (8.7)	27 (9.7)			25 (9.0)	27 (9.7)		
Living with partner		98 (4.3)	12 (4.3)			10 (3.6)	12 (4.3)		
Education (%)									
<9th grade		347 (15.1)	54 (19.4)	0.029	0.215	50 (18.0)	54 (19.4)	0.850	0.099
9–11th grade		412 (18.0)	56 (20.1)			60 (21.6)	56 (20.1)		
High school		577 (25.2)	78 (28.1)			70 (25.2)	78 (28.1)		
Some college		576 (25.1)	60 (21.6)			69 (24.8)	60 (21.6)		
College grad		379 (16.5)	30 (10.8)			29 (10.4)	30 (10.8)		
PIR cat (%)									
<1.3		706 (30.8)	108 (38.8)	0.004	0.215	111 (39.9)	108 (38.8)	0.566	0.091
1.3–3.5		1,026 (44.8)	123 (44.2)			129 (46.4)	123 (44.2)		
≥ 3.5		559 (24.4)	47 (16.9)			38 (13.7)	47 (16.9)		
Smoking (%)									
Never		1,109 (48.4)	158 (56.8)	0.021	0.179	152 (54.7)	158 (56.8)	0.876	0.044
Previous		776 (33.9)	74 (26.6)			78 (28.1)	74 (26.6)		
Current		406 (17.7)	46 (16.5)			48 (17.3)	46 (16.5)		
Hypertension (%)									
No		1,312 (57.3)	169 (60.8)	0.290	0.072	171 (61.5)	169 (60.8)	0.931	0.015
Yes		979 (42.7)	109 (39.2)			107 (38.5)	109 (39.2)		
Diabetes (%)									
No		518 (22.6)	61 (21.9)	0.861	0.016	58 (20.9)	61 (21.9)	0.836	0.026
Yes		1,773 (77.4)	217 (78.1)			220 (79.1)	217 (78.1)		
Heart failure (%)									
No		2,077 (90.7)	251 (90.3)	0.927	0.013	252 (90.6)	251 (90.3)	1.000	0.012
Yes		214 (9.3)	27 (9.7)			26 (9.4)	27 (9.7)		
Dialysis (%)									
No		2,254 (98.4)	269 (96.8)	0.092	0.106	267 (96.0)	269 (96.8)	0.820	0.039
Yes		37 (1.6)	9 (3.2)			11 (4.0)	9 (3.2)		
Opioid use (%)									
No		2,090 (91.2)	256 (92.1)	0.713	0.031	250 (89.9)	256 (92.1)	0.459	0.075
Yes		201 (8.8)	22 (7.9)			28 (10.1)	22 (7.9)		

^*a*^ Values are presented as mean ± standard deviation (SD) for continuous variables and frequency (percentage) for categorical variables. Abbreviations: SMD, standardized mean difference; BMI, body mass index; HbA1c, glycated hemoglobin; ALB, albumin; BUN, blood urea nitrogen; eGFR, estimated glomerular filtration rate; PIR, poverty income ratio.

### Survival analysis

**Fig 2:**
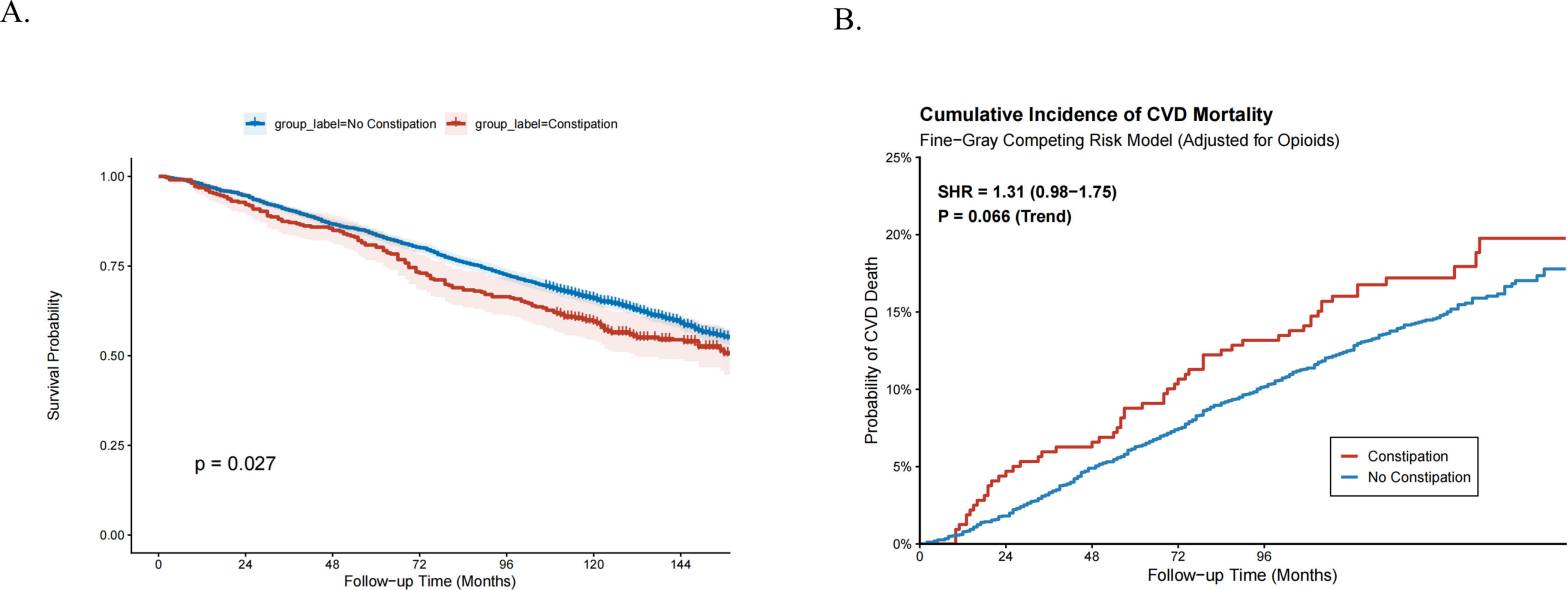
Impact of constipation on survival outcomes in patients with CKD. (**A**) Kaplan-Meier survival curves for all-cause mortality stratified by constipation status. The red line represents the constipation group, and the blue line represents the non-constipation group. Shaded areas indicate 95% confidence intervals. (**B**) Cumulative incidence function (CIF) curves for cardiovascular disease (CVD) mortality estimated using the Fine-Gray competing risk model. The analysis adjusted for potential confounders, including opioid usage, with non-CVD death treated as a competing event. The red line indicates the constipation group, showing a trend toward higher cumulative incidence of CVD death compared to the non-constipation group (blue line).

The death data were backdated to December 31, 2019, and after a median follow-up of 114 months, there were 1,742 deaths. Kaplan-Meier survival analysis demonstrated that CKD patients with constipation had a significantly lower cumulative survival probability compared to those without constipation (log-rank *P* = 0.027, Fig. 2A).

To determine whether constipation is an independent risk factor for mortality or merely a marker of poor health, we constructed three sequential multivariate Cox proportional hazards models (Table 2). In the unadjusted model (Model 1), constipation was associated with a 21% increased risk of all-cause mortality (HR: 1.21, 95% CI: 1.02–1.44, *P* = 0.027). Notably, after adjusting for demographic and socioeconomic factors (Model 2), the association strengthened (HR: 1.35, 95% CI: 1.13–1.60, *P* < 0.001), suggesting that demographic variables might partially mask the true impact of constipation in the crude analysis. In the fully adjusted model (Model 3), which extensively controlled for dietary habits (intake of water and fiber), comorbidities (hypertension, diabetes, CVD), kidney function markers (eGFR, BUN, UACR, ALB), and crucially, opioid use, the association remained robust and significant. Patients with constipation faced a 33% higher risk of mortality compared to those without (HR: 1.33, 95% CI: 1.11–1.58, *P* = 0.002).

**TABLE 2:** Association between constipation and all-cause mortality in patients with CKD^*a*^

Characteristic	HR	95% CI	*P* value^*b*^
Model 1: unadjusted	1.21	1.02, 1.44	**0.027**
Model 2: adjusted for demographics	1.35	1.13, 1.60	**<0.001**
Model 3: fully adjusted	1.33	1.11, 1.58	**0.002**

^*a*^ Values are presented as hazard ratios (HR) and 95% confidence intervals (CI). Model 1: unadjusted model. Model 2: adjusted for age, sex, race, BMI, education, family income to poverty ratio (PIR), and smoking status. Model 3: adjusted for covariates in Model 2 plus dietary factors (dietary fiber and water intake), hypertension, diabetes, history of CVD, dialysis status, opioid use, glycohemoglobin (HbA1c), albumin (ALB), blood urea nitrogen (BUN), and eGFR. Abbreviations: CKD, chronic kidney disease; BMI, body mass index; CVD, cardiovascular disease; eGFR, estimated glomerular filtration rate.

^*b*^ Bold values indicate statistical significance (*P *< 0.05).

### Cause-specific mortality and dose-response relationships

Given the high burden of cardiovascular disease in the CKD population, we further performed a competing risk analysis using the Fine-Gray model to specifically assess CVD mortality (Fig. 2B). The results revealed a notable trend toward increased CVD mortality risk in patients with constipation. Although the association did not reach the conventional threshold for statistical significance (SHR: 1.31, 95% CI: 0.98–1.75, *P* = 0.066), the cumulative incidence function (CIF) curves demonstrated a clear visual separation, with the constipation group consistently exhibiting a higher incidence of CVD death throughout the follow-up period compared to the non-constipated group. This suggests that while statistical power may be limited by the number of cause-specific events, the direction of the association is consistent with the all-cause mortality findings.

**Fig 3:**
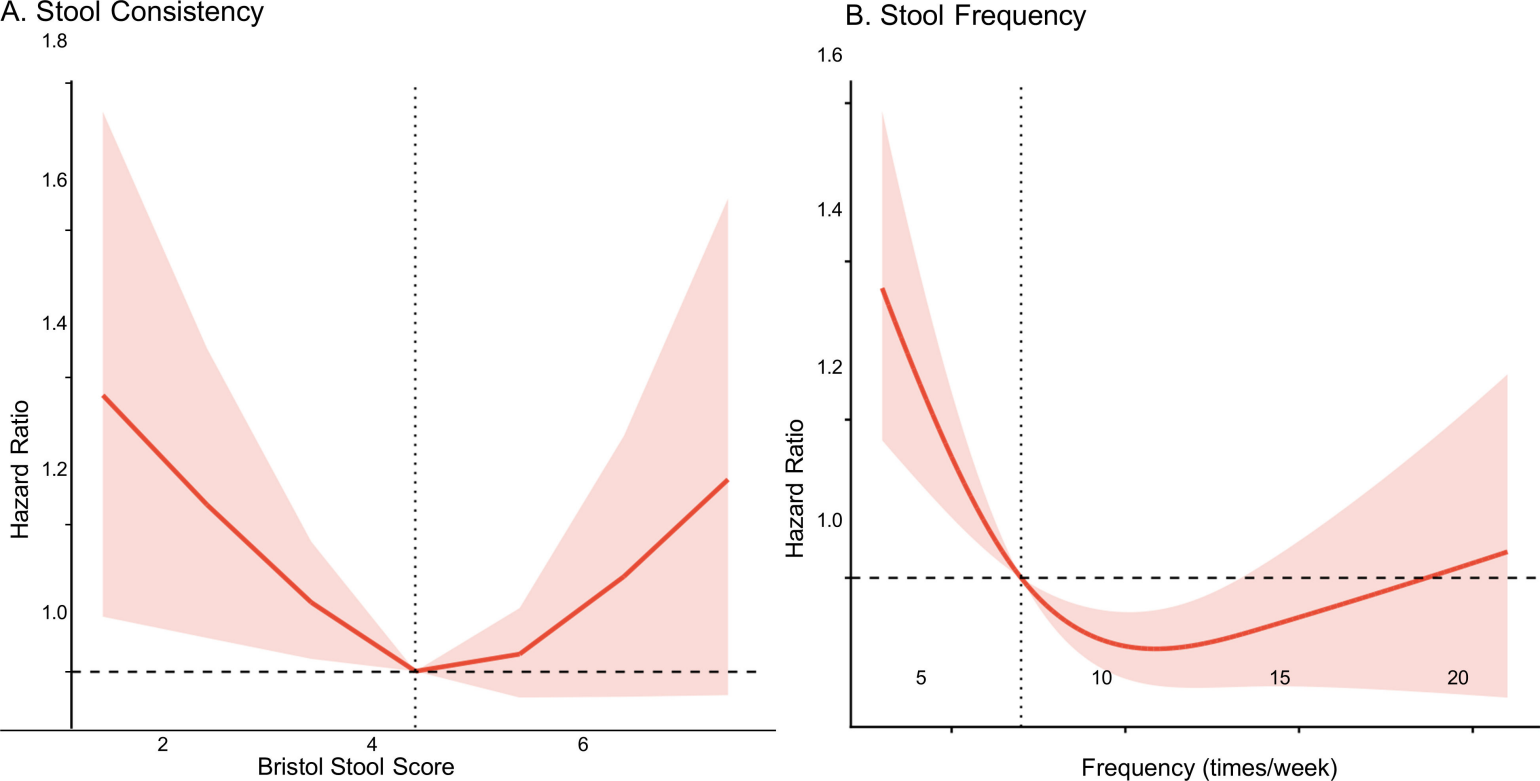
Dose-response relationship between bowel habits and all-cause mortality in CKD patients. Restricted cubic spline (RCS) models were used to visualize the non-linear association between stool characteristics and mortality hazard ratios (HRs). The solid red line represents the estimated HR, and the shaded red area indicates the 95% CI. The horizontal dashed line represents the reference hazard ratio of 1.0. (**A**) Stool consistency: analysis of the Bristol Stool Form Scale (BSFS) scores reveals a U-shaped association. (**B**) Stool frequency: analysis of stool frequency (times/week) demonstrates an L-shaped association.

To clarify the biological gradient of the associations, we utilized RCS analyses, which revealed distinct non-linear patterns (Fig. 3). Regarding stool consistency (Fig. 3A), a U-shaped relationship was observed. Using Type 4 (normal consistency) as the reference, the mortality risk increased significantly at both extremes of the BSFS. Specifically, harder stools (Types 1 and 2, indicating severe constipation) were associated with a steep increase in hazard ratios. Interestingly, loose or liquid stools (Types 6 and 7) also showed an upward trend in risk, suggesting that any deviation from normal bowel consistency—whether constipation or diarrhea—is detrimental in CKD patients. Regarding stool frequency (Fig. 3B), the analysis demonstrated a sharp inverse association. The risk of mortality rose precipitously as the weekly stool frequency dropped below seven times per week. In contrast, for frequencies exceeding seven times per week, the risk curve flattened and remained relatively stable, indicating that low bowel movement frequency is a specific and potent driver of excess mortality in this population.

### Subgroup analyses and interaction assessment

**Fig 4:**
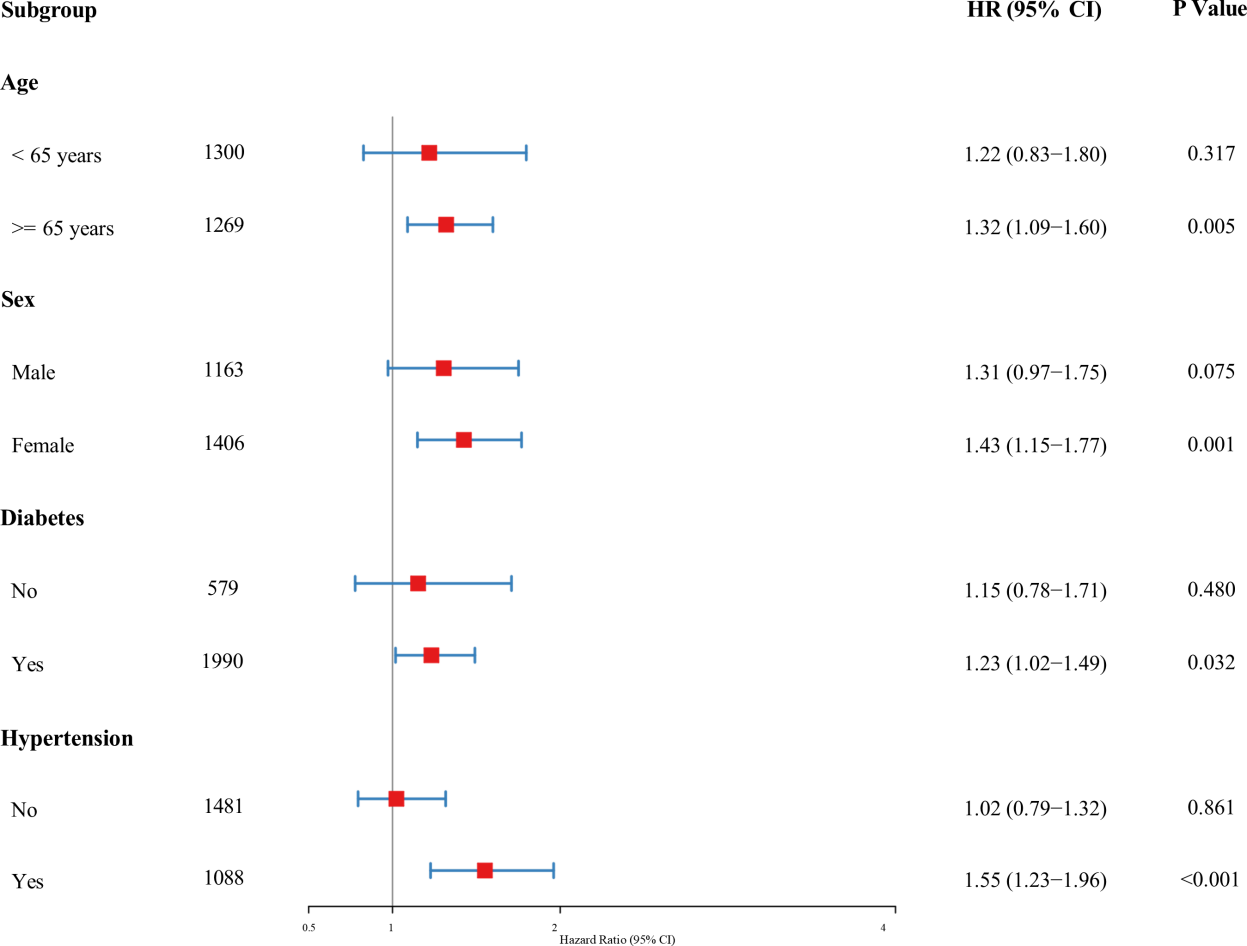
Stratified analyses of the association between constipation and all-cause mortality across key clinical subgroups. A forest plot displaying the multivariable-adjusted hazard ratios (HRs) for all-cause mortality associated with constipation. The red squares represent the point estimates of the HRs, and the horizontal blue lines indicate the 95% confidence intervals (CIs). The vertical gray line at HR = 1.0 serves as the reference for no effect.

Stratified analyses (Fig. 4) confirmed that the mortality risk associated with constipation was particularly pronounced in females (HR: 1.38, 95% CI: 1.11–1.71), participants aged ≥60 (HR: 1.28, 95% CI: 1.07–1.54), and non-Hispanic Whites (HR: 1.29). The direction of the associations remained consistent across other subgroups.

**Fig 5:**
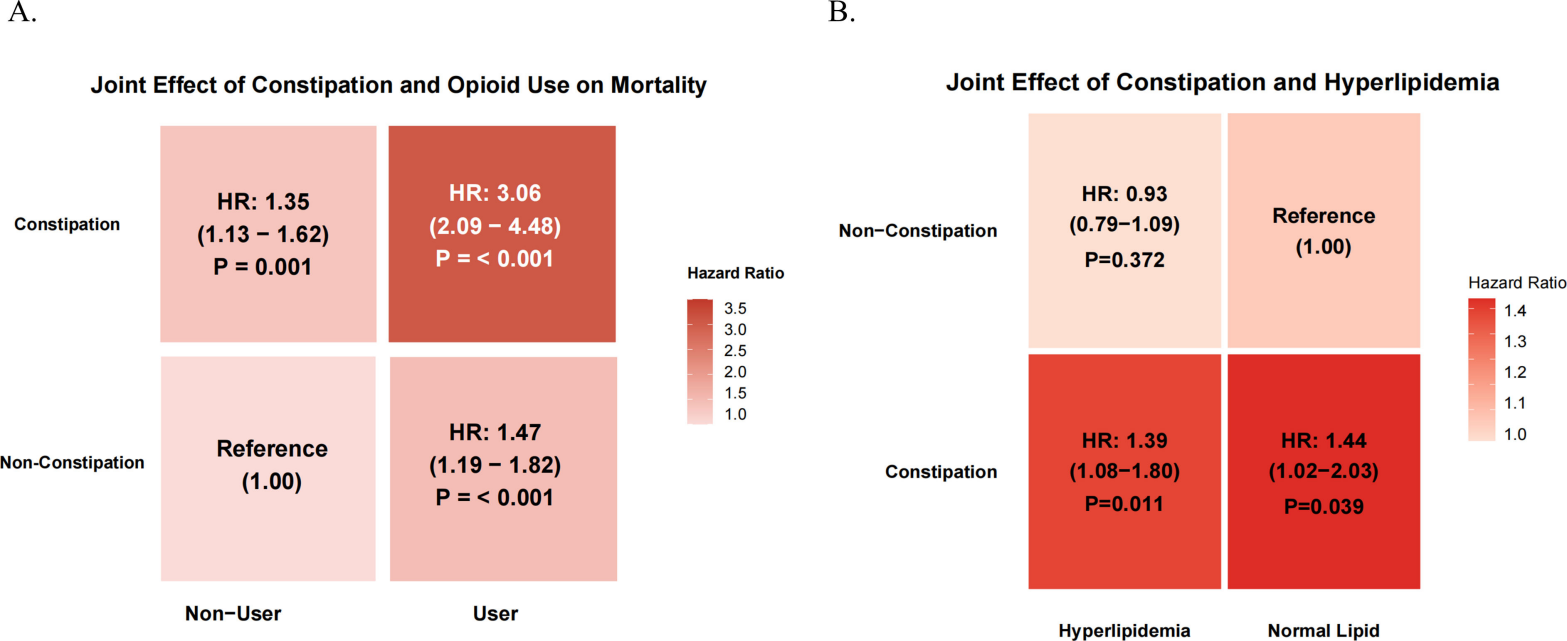
Heatmaps illustrating the joint effects of constipation and comorbidities/medications on all-cause mortality. (**A**) Joint effect of constipation and opioid use. The heatmap displays the hazard ratios (HRs) for combinations of constipation status (*y*-axis) and opioid usage (*x*-axis). The group with “Non-Constipation” and “Non-User” serves as the reference (HR = 1.00). The intensity of the red color corresponds to the magnitude of the mortality risk. (**B**) Joint effect of constipation and hyperlipidemia. This panel illustrates the interplay between constipation and hyperlipidemia status.

Joint effect analyses visualized via heatmaps (Fig. 5) revealed distinct interaction patterns. First, a strong synergistic effect was observed between constipation and opioid use (Fig. 5A); patients with both conditions faced a threefold increase in mortality risk (HR: 3.06, 95% CI: 2.09–4.48) compared to controls. Second, regarding lipid status (Fig. 5B), constipation significantly elevated mortality risk regardless of hyperlipidemia. Notably, the constipation group with normal lipid levels exhibited the highest risk (HR: 1.44, *P* < 0.05), suggesting that constipation exerts a detrimental effect independent of traditional lipid metabolic disorders.

### Sensitivity and robustness checks

To strictly rule out potential biases and validate the reliability of our findings, we performed a comprehensive set of sensitivity analyses (Fig. S4). In the “no opioids“ sensitivity model, the association between constipation and mortality remained statistically significant (HR: 1.32, 95% CI: 1.11–1.60), confirming that our results were not driven by opioid-induced constipation. Second, to mitigate reverse causality (where severe illness leads to both constipation and death), we excluded participants who died within the first two years of follow-up. The results remained consistent (HR: 1.36, 95% CI: 1.15–1.70), demonstrating that the association is not an artifact of terminal illness.

### MR mediation analysis

#### Absence of direct causal effect of constipation on CKD

To delineate the causal architecture underlying the associations observed in the NHANES cohort, two-sample MR analyses were initially performed to test for a direct causal link. The inverse variance weighted (IVW) method revealed no statistically significant direct causal effect of genetically predicted constipation on CKD susceptibility (*P* > 0.05; Fig. S5). This null result suggests that the clinical correlation between constipation and mortality is likely mediated by indirect ecological or metabolic pathways rather than direct pathogenic mechanisms.

#### Influence of the gut microbiota on CKD

Unless otherwise specified, the odds ratios (ORs) reported in this section represent the risk of CKD per 1-standard deviation (SD) increase in the genetically predicted relative abundance of the respective bacterial taxa. Multivariate regression analysis identified four bacterial groups associated with an increased risk of CKD, among which the *Parabacillus* genus showed the strongest effect (OR = 2.477, 95% CI = 1.473–4.165, *P* = 0.001), followed by *Clostridium* spp., *Eubacterium* CAG-274 spp., and *Blautia* spp.

**Fig 6:**
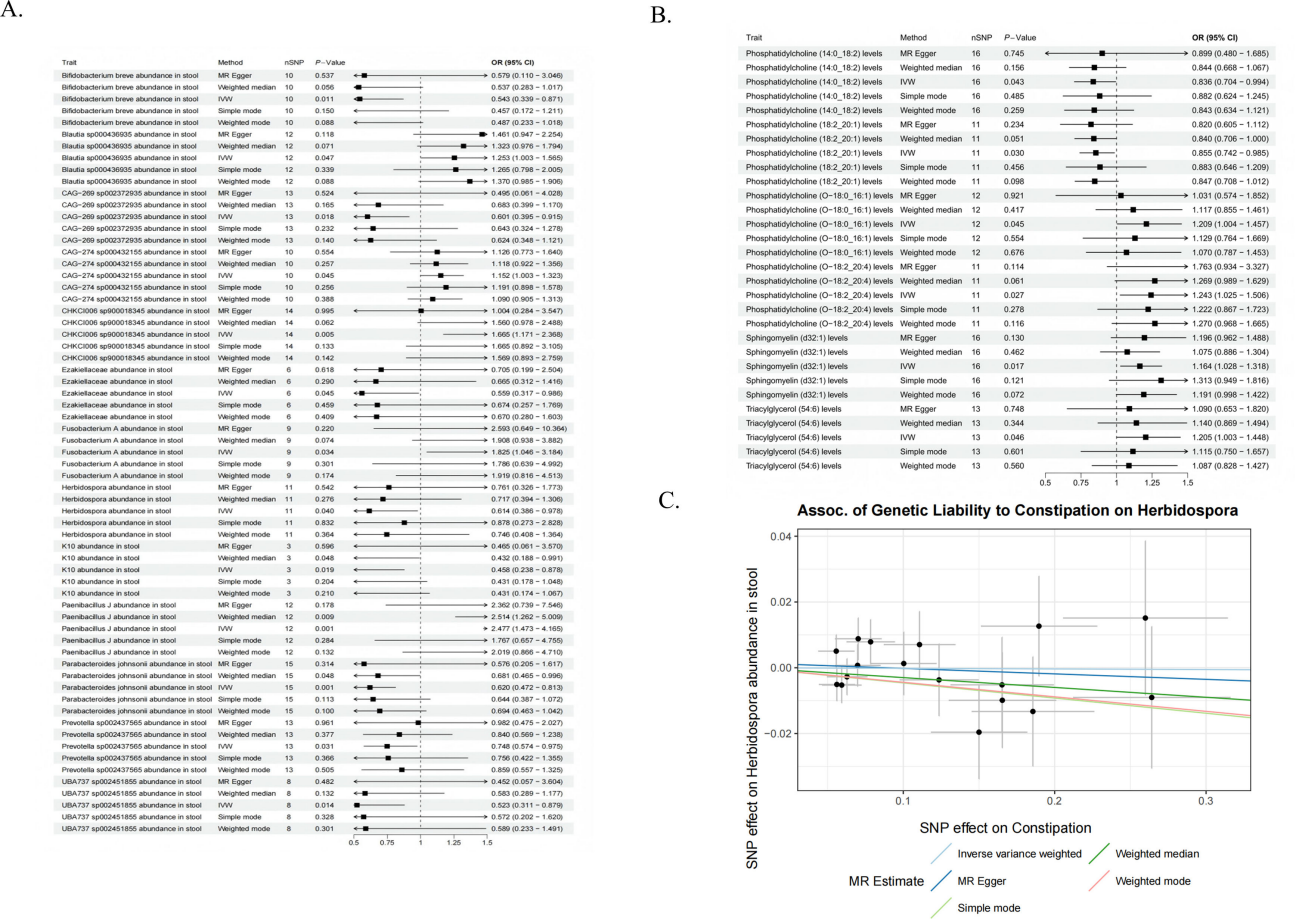
Mendelian randomization (MR) evidence elucidating the causal components of the gut-lipid-kidney axis. (**A**) Forest plot displaying the causal effects of specific gut microbiota taxa on CKD risk. Estimates are derived from the inverse variance weighted (IVW) method and sensitivity analyses (MR-Egger, weighted median). The *x*-axis represents the odds ratio (OR). Taxa with an OR < 1 are identified as renoprotective, while those with an OR > 1 are associated with increased risk. (**B**) Causal associations between genetically predicted plasma lipid metabolites and CKD. (**C**) Scatter plot demonstrating the upstream causal impact of constipation on Herbidospora abundance. The *x*-axis represents the SNP effects on constipation (exposure), and the y-axis represents the SNP effects on *Herbidospora* abundance (outcome).

Conversely, eight taxonomic groups were identified as potential protective factors. These include *Herbidospora* (OR = 0.614, 95% CI: 0.386–0.978, *P* = 0.040), as well as other bacteria with protective effects, including some well-known groups, such as *Bifidobacterium* and *Prevotella*, as well as *Parabacteroides johnsonii* spp., *Fenollaria* spp., and other bacterial species detailed in Fig. 6A and Fig. S7.

#### Impact of lipids on CKD

MR analysis revealed causal correlations linking lipid-related variables to CKD development. Specifically, four factors, namely, PC (O-18:0_16:1) levels (OR = 1.209, 95% CI: 1.004–1.457, *P* = 0.045), PC (O-18:2_20:4) levels (OR = 1.243, 95% CI: 1.025–1.506, *P* = 0.027), sphingomyelin (d32:1) levels (OR = 1.164, 95% CI: 1.028–1.318, *P* = 0.017), and triacylglycerol (54:6) levels (OR = 1.205, 95% CI: 1.003–1.448, *P* = 0.046), were positively correlated, suggesting that they may increase the risk of developing CKD. PC (14:0_18:2) levels (OR = 0.836, 95% CI: 0.704–0.994, *P* = 0.043) and PC (18:2_20:1) levels (OR = 0.855, 95% CI: 0.742–0.985, *P* = 0.03) were negatively associated with CKD risk, suggesting a potential protective effect against CKD (Fig. 6B; Fig. S8). A sensitivity analysis was also conducted to validate these findings.

#### Mediation analysis: the *Herbidospora*-PC axis

To understand the mechanism by which *Herbidospora* (a subgenus of the phylum *Actinomycetes*) confers protection, we investigated its metabolic output. Mediation analysis revealed that PC (14:0_18:2) mediates a significant proportion of the causal relationship between *Herbidospora* and CKD. The total protective effect of *Herbidospora* (beta = −0.487) was partially mediated by PC (14:0_18:2), accounting for a mediation proportion of 12.5%. This establishes a functional *Herbidospora*-PC (14:0_18:2) axis that synergistically protects against CKD.

In this mediated MR study, causal associations between 12 microbial taxa, including *Herbidospora*, and CKD risk were identified. The mediation analysis revealed that PC accounted for 12.5% of the total effect of *Herbidospora* on CKD. These findings suggest that *Herbidospora*-derived PC (14:0_18:2) may exert renoprotective effects by targeting key regulatory pathways.

#### Constipation drives the depletion of the *Herbidospora* axis

Having established the renoprotective role of the *Herbidospora*-PC axis, we sought to determine if constipation disrupts this specific pathway. MR analysis confirmed a significant inverse causal association between genetic liability to constipation and the abundance of *Herbidospora* (beta = −0.36, *P* = 0.02). This finding completes the causal chain: constipation causally drives the depletion of *Herbidospora*, thereby impairing the downstream production of protective PC (14:0_18:2) and compromising renal health (Fig. 6C).

#### Assessing the robustness of the results

Sensitivity analyses confirmed the robustness of our MR findings. No significant heterogeneity or horizontal pleiotropy was detected via MR-Egger and MR-PRESSO tests (Tables S1 to S4; Fig. S9 and S10). Significance thresholds were adjusted using the Bonferroni correction based on the number of taxa tested at each level (30); associations with *P* < 0.05 but exceeding these thresholds were considered suggestive. Finally, leave-one-out analyses demonstrated that the results were not driven by any single SNP (Fig. S11 and S12).

#### Reverse Mendelian randomization

Inverse MR analysis revealed causal associations between *Bifidobacterium* spp. (OR = 0.955, 95% CI = 0.916−0.996; *P* = 0.035) and *Paenibacillus* J*.* spp. *(*OR = 0.98, 95% CI = 0.960−0.999; *P* = 0.049) and the prevalence of CKD. These results suggest that the potential role of *Bifidobacterium* spp. and *Paenibacillus J* spp. should be considered in future studies on the impact of CKD on the gut microbiota (Table S5).

### Hypothesis generation: from molecular recognition to environmental transmission

To explore molecular feasibility, we performed molecular docking and 100 ns MD simulations. Results showed that PC (14:0_18:2) stably occupies the PPARγ hydrophobic pocket, maintained by van der Waals interactions with minimal conformational fluctuations (Fig. S6). These data suggest PC (14:0_18:2) as a putative PPARγ ligand (details in Note S1).

**Fig 7:**
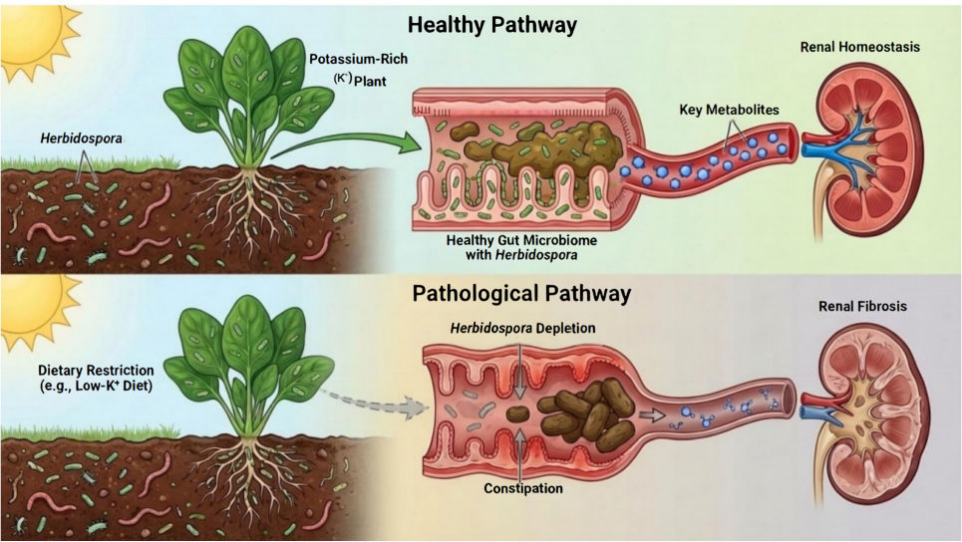
Schematic representation of the environment-diet-gut-kidney axis involving *Herbidospora.* (Top) Homeostatic pathway. Consumption of unrefined, potassium-rich plant foods permits the ingestion of soil-associated *Herbidospora*. This taxon functions as a metabolic source of phosphatidylcholine (14:0_18:2) (represented by blue hexagons). These lipid metabolites enter the systemic circulation and support the maintenance of renal tissue integrity. (Bottom) Disrupted pathway in CKD. The therapeutic implementation of a low-potassium diet necessitates food processing methods (e.g., soaking, thorough cooking) that eliminate the exogenous source of *Herbidospora*. This exclusion, compounded by the physiological stress of constipation, results in a marked depletion of *Herbidospora* abundance. The consequent deficiency in protective phospholipids diminishes the metabolic defense against renal injury, potentially facilitating fibrosis.

Beyond the molecular interaction, we further integrated the epidemiological associations with clinical dietary realities to propose a macroscopic transmission route. As illustrated in Fig. 7, we hypothesize that the depletion of *Herbidospora* in CKD patients is driven by the disruption of the “soil-plant-gut” transmission chain, primarily due to the strict low-potassium dietary management. This schematic diagram summarizes the proposed multi-level mechanism: from environmental intake interruption to the loss of renoprotective lipid metabolites.

## DISCUSSION

Our study integrates clinical epidemiology with multi-omics causal inference to elucidate the prognostic role of intestinal motility in CKD and to uncover its potential microbial underpinnings. Our findings provide robust evidence that constipation is an independent risk factor for all-cause mortality in the CKD population, distinct from the confounding effects of opioid usage or metabolic comorbidities. Beyond this clinical association, our MR analysis reveals a novel, protective role of the environmental genus *Herbidospora*, suggesting that the gut-kidney axis extends beyond resident flora to include environmental microbial exposures.

Clinical data from large cohorts emphasize that constipation in CKD is not only a benign functional disorder but also a key driver of adverse outcomes. Unlike the general population, CKD patients have long been in a uremic environment characterized by impaired intestinal barrier integrity. We believe that constipation plays a double blow in this situation: the prolonged colonic transport time to some extent increases the exposure of the epithelium to uremic toxins, promoting their systemic translocation. This mechanism explains the independent risk of death observed in our study, even when traditional cardiovascular and kidney-specific risk factors were strictly adjusted through LASSO regression.

We acknowledge that the two cohorts differ in geographical and genetic backgrounds. Despite these differences, the consistent direction of our findings across distinct populations suggests that the gut-kidney axis may be a generalizable phenomenon, though direct causal inference from our integrated approach requires further validation. Our identification of constipation-associated mortality risk in the NHANES cohort may correlate with the protective deficiency observed in the FINRISK cohort. Constipation potentially disrupts the gut microbiota balance, increasing the production of harmful substances. The accumulation of these toxins may induce oxidative stress, increase intestinal permeability, and hinder protective microbiota colonization. Consequently, this reduces beneficial metabolite generation, ultimately compromising renal health (30, 31).

We discovered that *Herbidospora mongoliensis* plays a primary role within the *Actinomycetota* phylum. This bacterium predominantly inhabits soil or grassland environments and has previously been extremely rare in the human gut. We propose an ecological hypothesis consistent with these findings and amenable to verification. The “soil-plant-gut” constitutes a continuous microbial transmission chain. Environmental microorganisms entering the human gut via the alimentary pathway may temporarily influence host metabolism or establish functional colonization. Specifically, CKD patients require a strict low-potassium diet and often need to peel, soak, and thoroughly cook root vegetables, which may unintentionally cut off the main contact route of soil-related flora (32, 33). Notably, *Herbidospora mongoliensis* is classified as a phospholipid-producing PIV strain. This isolate possesses the unique capability to synthesize mixed-acyl PC, consistent with its role in CKD protection (34). Our findings broaden therapeutic strategies and advocate for intervention measures targeting specific functional metabolites to compensate for the inevitable loss of environmental microbial exposure due to necessary dietary restrictions.

This study has several limitations. First, due to the cross-sectional design of the NHANES baseline survey, our survival analysis relied on single-point measurements of covariates. Consequently, we could not account for time-varying changes or cumulative effects of lifestyle factors (e.g., changes in smoking or alcohol habits) or medication adjustments during the follow-up period. Second, data on severe constipation and dietary variables were primarily self-reported, inevitably introducing subjective bias and recall errors. Third, differences in geographical distribution and population composition between the U.S. (NHANES) and European (GWAS) cohorts may limit direct comparability. However, the identification of a plausible gut-kidney axis signal across these diverse settings strengthens the possibility that we have uncovered a fundamental and conserved biological pathway. Fourth, while MR minimizes confounding, it cannot prove causality like randomized trials, and molecular docking only predicts structural interactions without confirming biological activation. Therefore, our findings should be interpreted as supportive mechanistic hypotheses requiring experimental validation.

### Conclusion

Evidence from this multi-cohort study outlines a novel gut-lipid-kidney axis, linking constipation-associated mortality in CKD to gut dysbiosis. The trajectory extends from genetic identification of *Herbidospora mongoliensis* and its metabolite PC (14:0_18:2) as protective factors to a structural mechanism via stable binding to PPARγ. Consequently, targeting this axis—through next-generation probiotics or metabolite supplementation—represents a translatable strategy for slowing CKD progression.

## Data Availability

The U.S. NHANES data are available online at https://wwwn.cdc.gov/nchs/nhanes/Default.aspx. The Mendelian randomization data set is publicly accessible via repository name and accession code as detailed in the supplemental material.

## SUPPLEMENTAL MATERIAL

Supplemental results msphere.00914-25-s0001.xlsx Raw results of molecular dynamics simulation.

Supplemental material msphere.00914-25-s0002.docx Supplemental figures, tables, and note.

ASM does not own the copyrights to Supplemental Material that may be linked to, or accessed through, an article. The authors have granted ASM a non-exclusive, world-wide license to publish the Supplemental Material files. Please contact the corresponding author directly for reuse.
