# Supplementary material for: New hypothesis: a gut-lipid-kidney axis in constipated CKD patients—insights from multi-omics triangulation
Source: mSphere. 2026 Feb 5;11(2):e00914-25. doi: 10.1128/msphere.00914-25 (PMC12931264; doi:10.1128/msphere.00914-25)
Supplement: Supplemental material — Supplemental figures, tables, and note. [file msphere.00914-25-s0002.docx]

**Supplementary material**

| **Numbering** | **Content Description** |
| --- | --- |
| **Supplementary Figure 1** | **Comparison of distributions between observed and imputed data using multiple imputation.** |
| **Supplementary Figure 2** | **Feature selection for the multivariate Cox proportional hazards model using Least Absolute Shrinkage and Selection Operator (LASSO) regression.** |
| **Supplementary Figure 3** | **Distribution of propensity scores before and after propensity score matching (PSM).** |
| **Supplementary Figure 4** | **Sensitivity analyses of the association between constipation and all-cause mortality in patients with CKD.** |
| **Supplementary Figure 5** | **Mendelian randomization analysis investigating the causal effect of genetically predicted constipation on CKD risk (Scatter plot, Forest plot, and Funnel plot)** |
| **Supplementary Figure 6** | **Molecular docking and molecular dynamics simulation results.** |
| **Supplementary Figure 7** | **A forest plot demonstrates causal relationship between gut microbiota and CKD.** |
| **Supplementary Figure 8** | **A forest plot demonstrates causal relationship between lipids and CKD.** |
| **Supplementary Figure 9** | **Funnel plot of causal effects of gut microbiota** |
| **Supplementary Figure 10** | **Funnel plot of causal effects of lipid** |
| **Supplementary Figure 11** | **Leave-one-out analysis of the causal effect of gut microbiota** |
| **Supplementary Figure 12** | **Leave-one-out analysis of the causal effect of lipids** |
| **Supplementary Table 1** | **MR-Egger regression analyses of gut microbiota.** |
| **Supplementary Table 2** | **MRPRESSO analysis of gut microbiota.** |
| **Supplementary Table 3** | **MR-Egger regression analyses of lipids.** |
| **Supplementary Table 4** | **MRPRESSO analysis of lipids.** |
| **Supplementary Table 5** | **Results of reverse Mendelian randomization: effects of CKD on gut microbiota.** |
| **Supplementary Table 6** | **MR results of causal links between gut microbiota and CKD.** |
| **Supplementary Table 7** | **MR results of causal links between lipids and CKD.** |
| **Supplementary Table 8** | **MR results of causal links between genus Herbidospora and phosphatidylcholine.** |
| **Supplementary Table 9** | **The ids of the data used in this study.** |
| **Supplementary Table 10** | **Single-nucleotide polymorphisms associated with exposure and outcome.** |

**Supplementary Figure 1:Comparison of distributions between observed and imputed data using multiple imputation.**


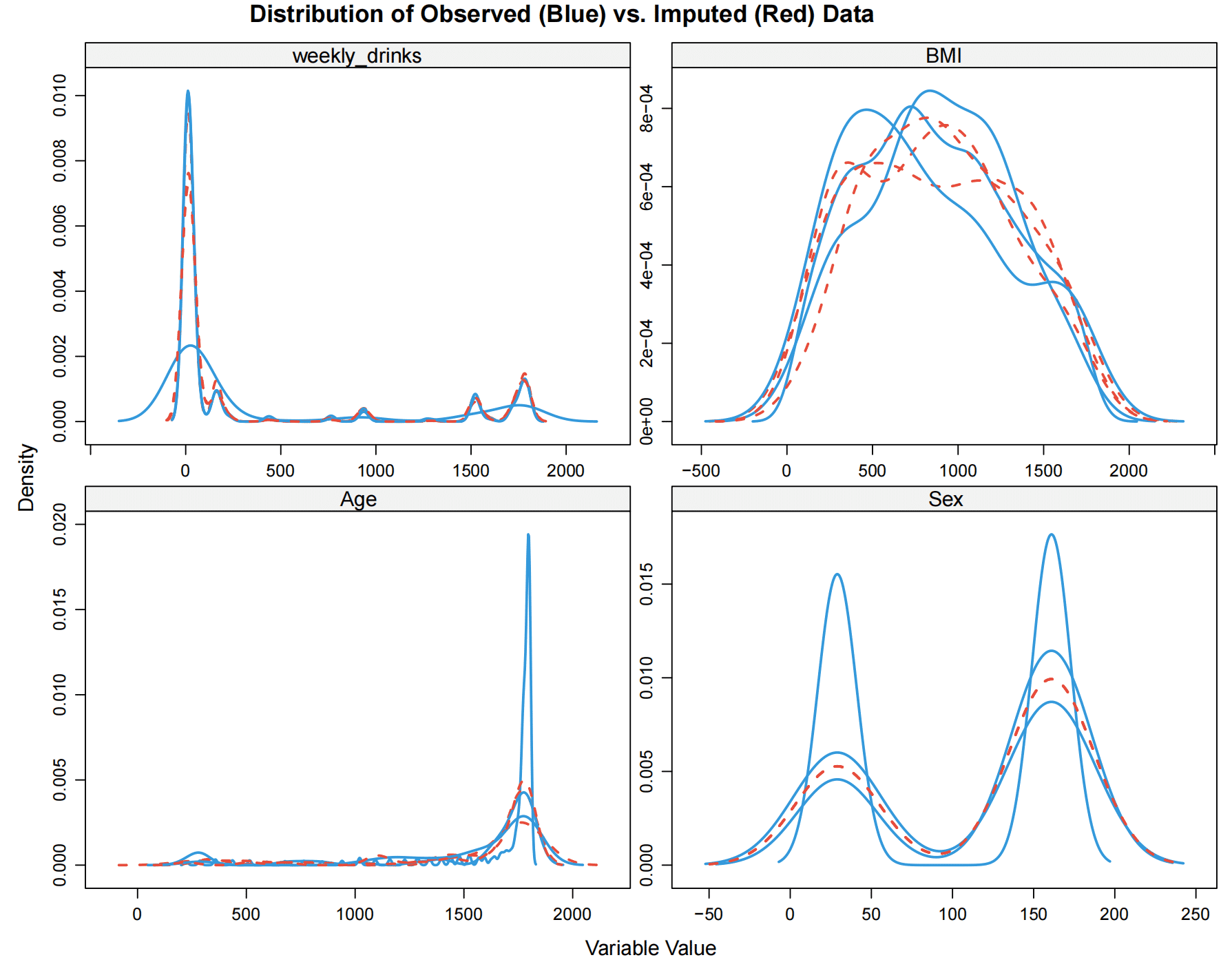


Legend:Density plots illustrate the distribution of observed data (blue solid lines) and imputed data (red dashed lines) for selected covariates, including alcohol consumption (weekly drinks), Body Mass Index (BMI), age, and sex. Multiple Imputation by Chained Equations (MICE) was employed to handle missing values.

### ****Supplementary Figure 2. Feature selection for the multivariate Cox proportional hazards model using Least Absolute Shrinkage and Selection Operator (LASSO) regression.****


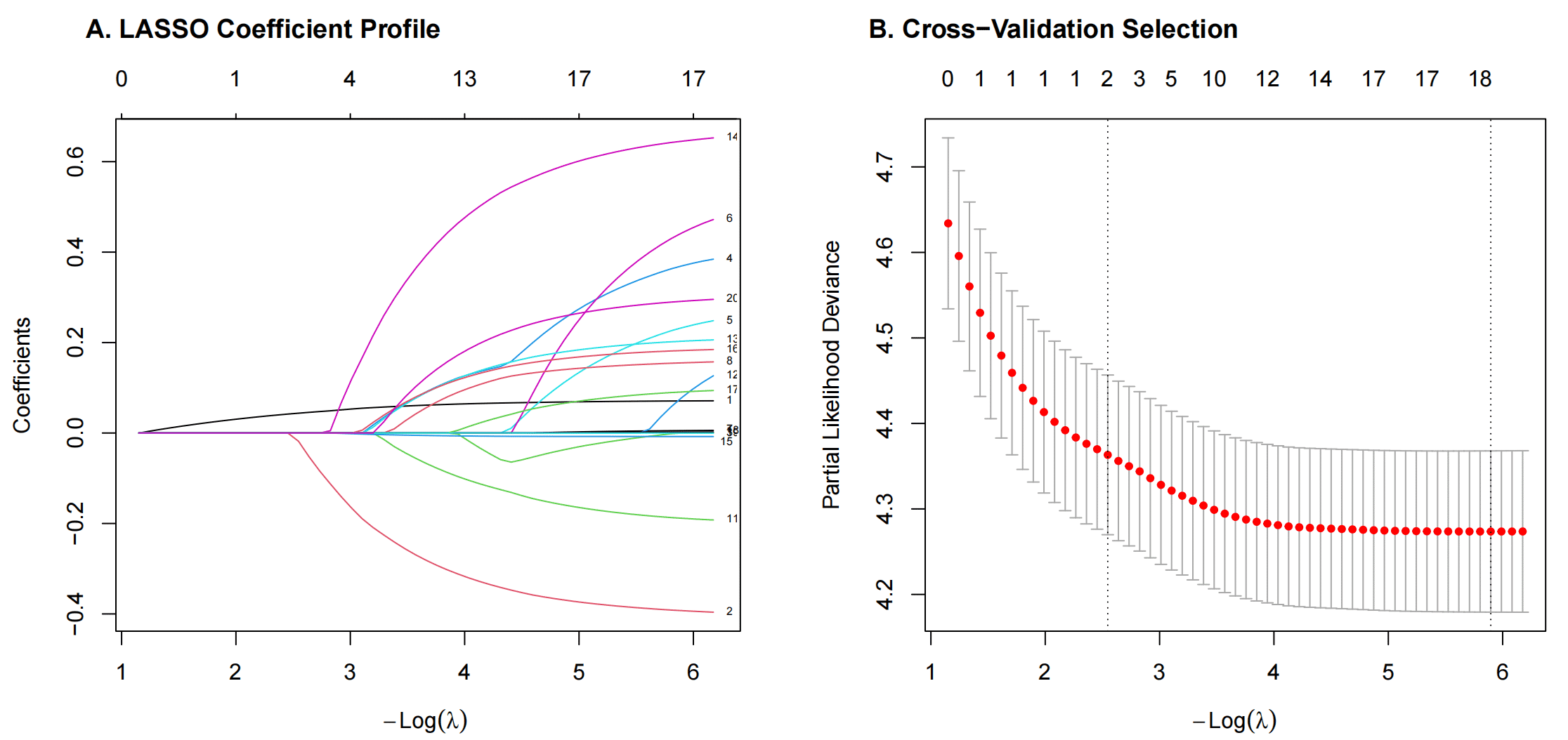


Legend:(A) LASSO coefficient profiles of the candidate covariates. Each colored line represents a variable. The vertical axis represents the coefficient value, and the lower horizontal axis represents the log(λ) sequence. As λλ increases, coefficients shrink toward zero, effectively excluding non-informative variables.
(B) Selection of the optimal tuning parameter (λ) using 10-fold cross-validation via minimum criteria and the 1-standard-error rule (1-SE). The partial likelihood deviance (y-axis) is plotted against log(λ) (x-axis). The left vertical dotted line indicates the λ value that minimizes the partial likelihood deviance (λmin), while the right vertical dotted line indicates the largest λ value within one standard error of the minimum (λ1se), which provides the most regularized model.

### ****Supplementary Figure 3. Distribution of propensity scores before and after propensity score matching (PSM).****


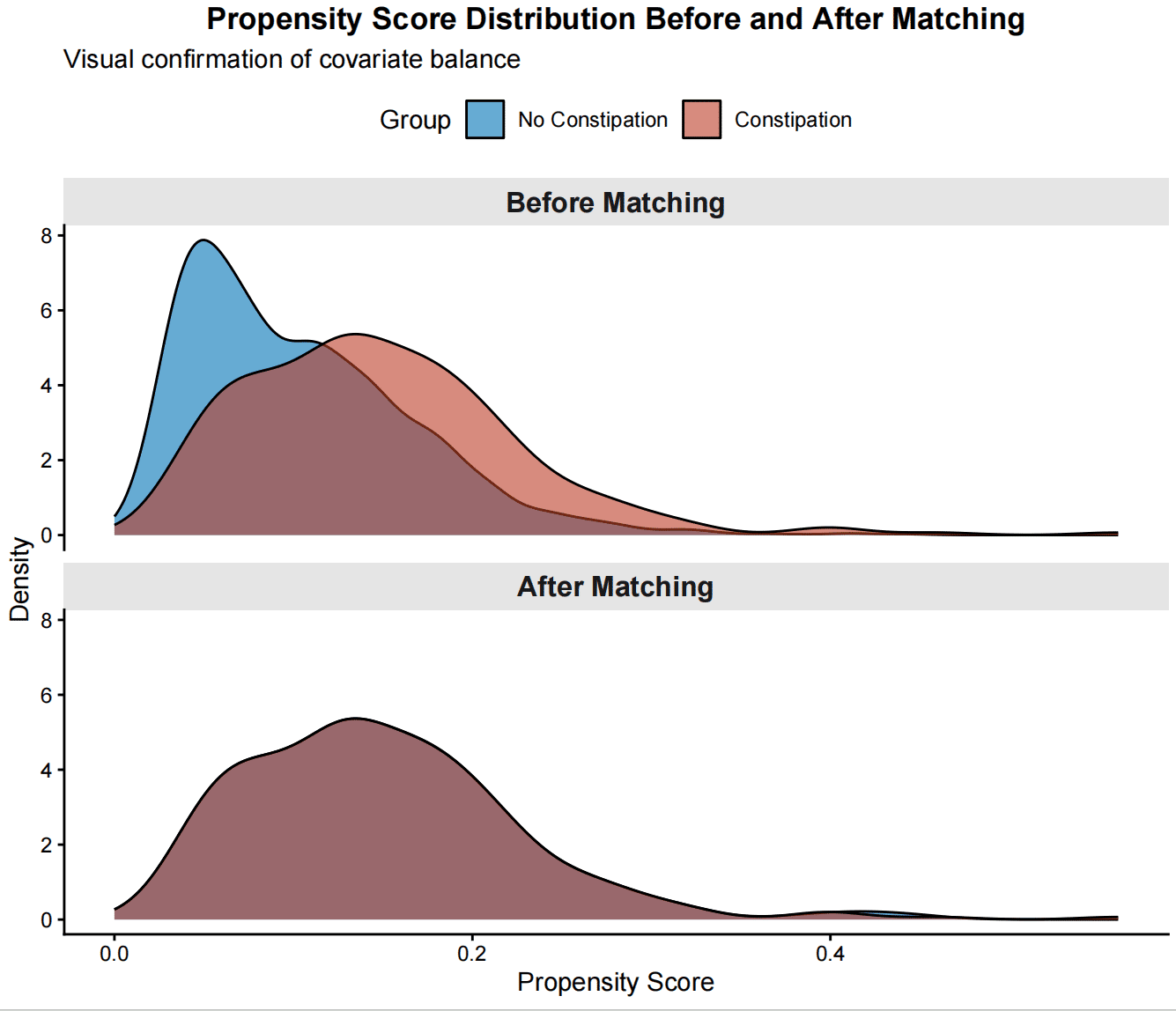


Legend:The density plots illustrate the distribution of propensity scores for patients with constipation (red) and without constipation (blue). (Top) Before matching, the distributions showed separation, indicating baseline differences between the two groups. (Bottom) After 1:1 nearest-neighbor matching, the distributions overlap , indicating that the covariates were well-balanced between the two groups and bias was effectively minimized. The x-axis represents the estimated propensity score, and the y-axis represents the density.

**Supplementary Figure 4:Sensitivity analyses of the association between constipation and all-cause mortality in patients with CKD.**


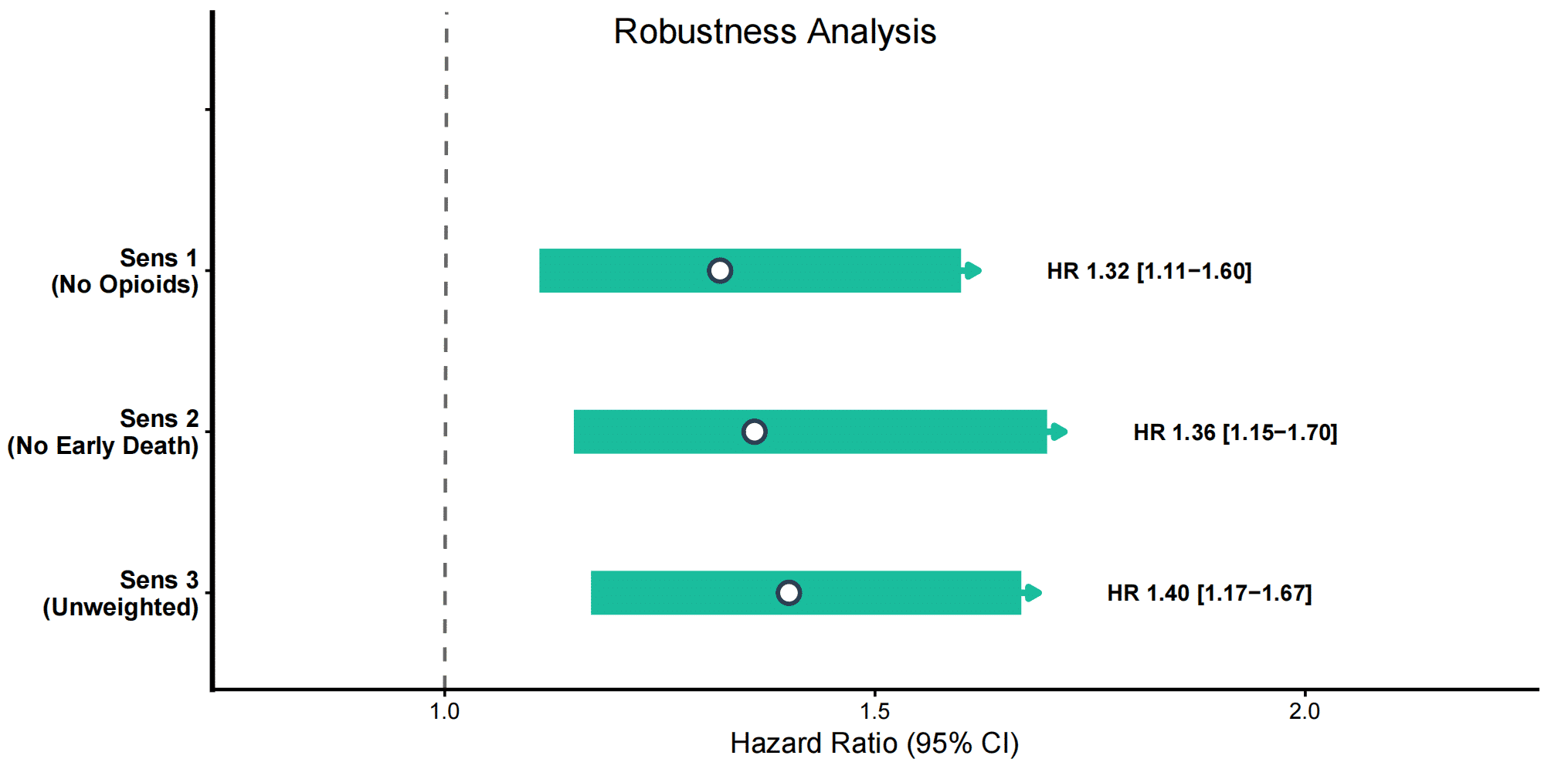


Legend:The forest plot summarizes the hazard ratios (HR) and 95% confidence intervals (CI) for the risk of all-cause mortality across different analytical scenarios.Sens 1 (No Opioids): Analysis excluding participants with opioid use to rule out drug-induced confounding.Sens 2 (No Early Death): Excluding deaths within the first 2 years to minimize reverse causality.Sens 3 (Unweighted): Unweighted Cox regression analysis to verify the stability of results without complex survey weights.The vertical dashed line indicates an HR of 1.0. The results indicate that constipation is consistently associated with an increased risk of death from any cause in the CKD population.

**Supplementary Figure 5:Mendelian randomization analysis investigating the causal effect of genetically predicted constipation on CKD risk (Scatter plot, Forest plot, and Funnel plot).**


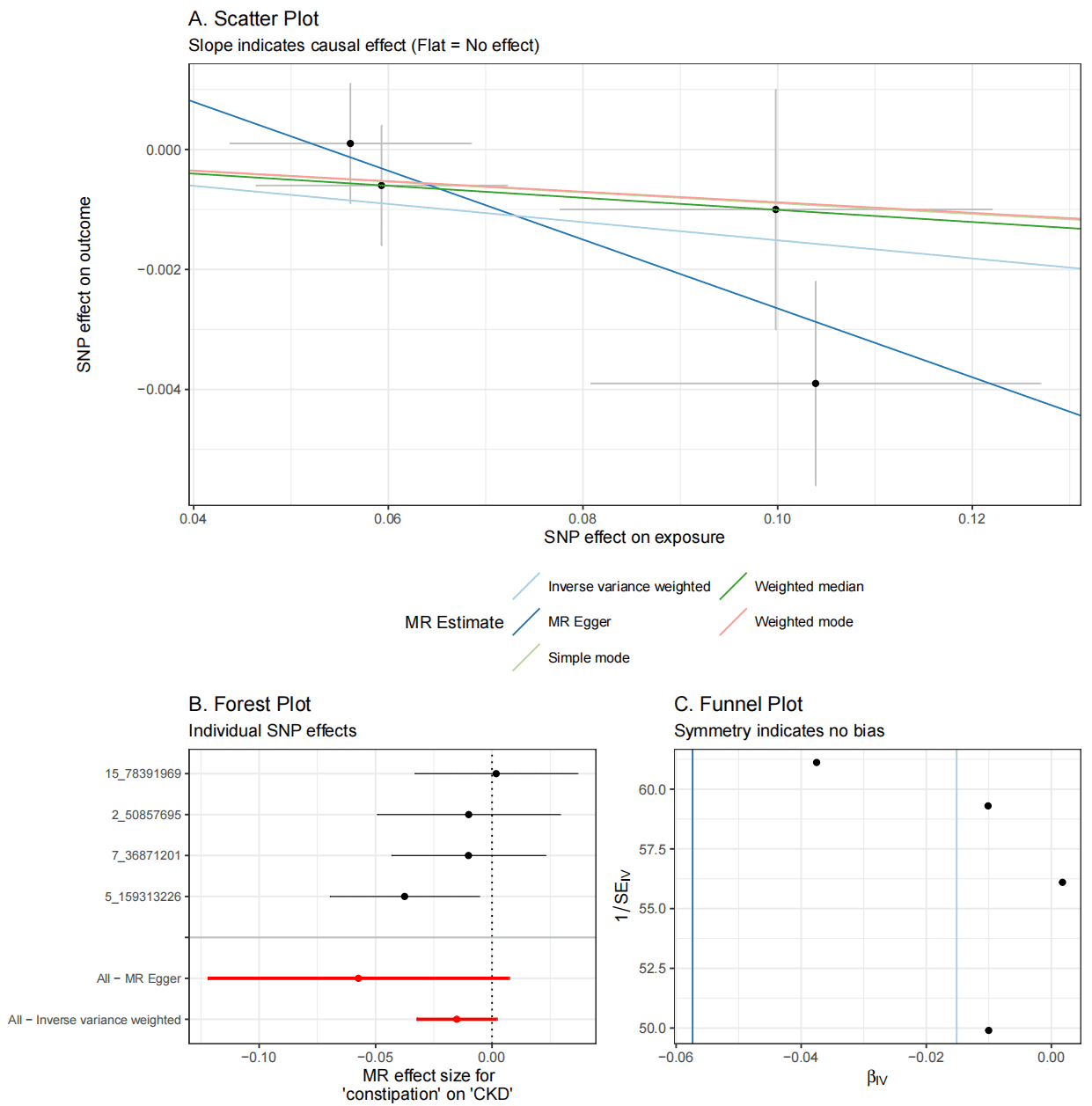


Legend:(A) Scatter plot: The x-axis represents the SNP effects on constipation (exposure), and the y-axis represents the SNP effects on CKD (outcome). The slopes of the colored lines correspond to the causal estimates derived from different MR methods (Light blue: Inverse variance weighted; Dark blue: MR Egger; etc.). (B) Forest plot: This plot visualizes the effect size (OR) and 95% confidence interval (CI) for each individual SNP (black dots) and the pooled results from different MR methods (colored lines at the bottom). (C) Funnel plot: A visual assessment for horizontal pleiotropy. The x-axis represents the causal effect estimate, and the y-axis represents the instrument precision (Inverse standard error). The symmetrical distribution of SNPs suggests no evidence of significant publication bias or directional horizontal pleiotropy.Abbreviations: MR: Mendelian randomization; SNP: Single nucleotide polymorphism; OR: Odds ratio; CI: Confidence interval; IVW: Inverse variance weighted.

**Supplementary Figure 6: Molecular docking and molecular dynamics simulation results.**


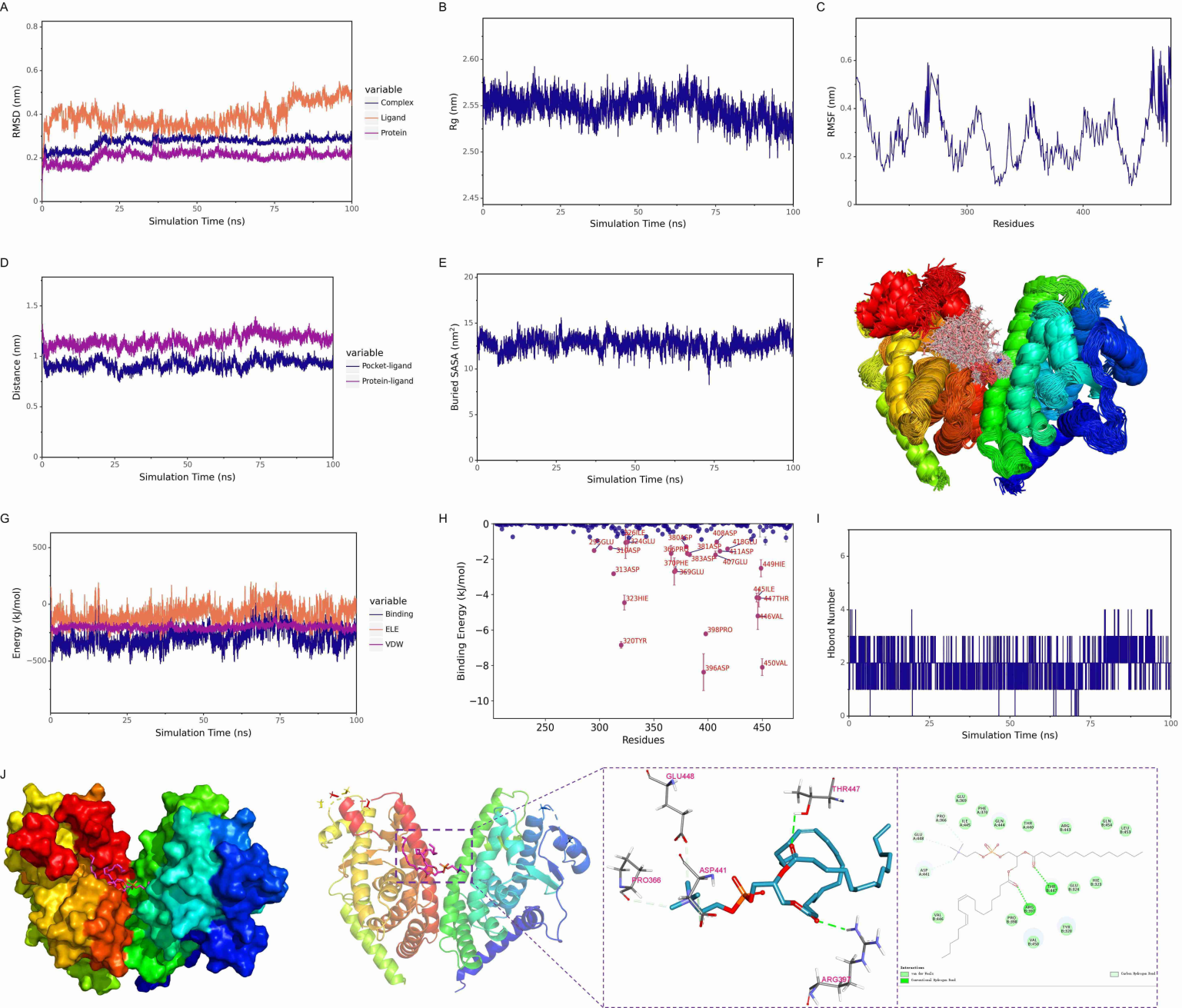


(A) RMSD of complexes, proteins and small-molecule ligands; (B) Rg of the complex; (C) RMSF of proteins in the complex; (D) protein distance to the binding site of small molecules (Dock site-ligand); (E) embedding area between small molecules and proteins (Buried SASA); (F) simulated conformation superposition; (G) binding energy between small molecules and proteins VDW and ELE; (H) contribution of amino acid binding energy; (I) hydrogen bond number (Hbond number); (J) egg Interactions between white and small molecules. Complex: PPARγ combined with PC(14:0_18:2); protein: PPARγ; small molecule ligand: PC(14:0_18:2); RMSD: root mean square deviation; Rg: radius of rotation; RMSF: root mean square fluctuation; VDW: van der Waals force; ELE: electrostatic energy

**Supplementary Figure 7：A scatter plot demonstrates causal relationship between gut microbiota and CKD.(A), Bifidobacterium spp (B),Blautia spp (C),CAG-269 spp (D),Fenollaria spp (E),Clostridium spp (F),Eubacterium CAG-274 (H),K10 spp (I),Herbidospora spp (J),Paenibacillus J spp (K),Prevotella spp(L),Parabacteroides johnsonii (M),Rumenococcaceae UBA737.**


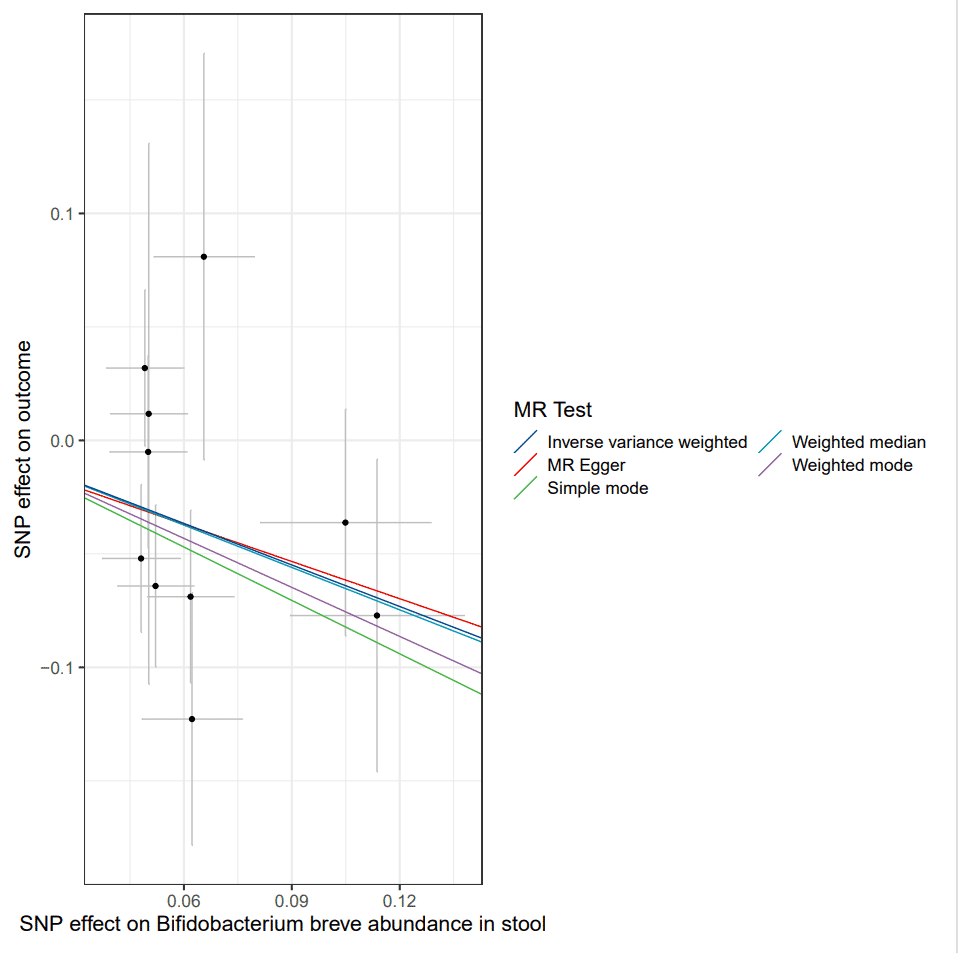

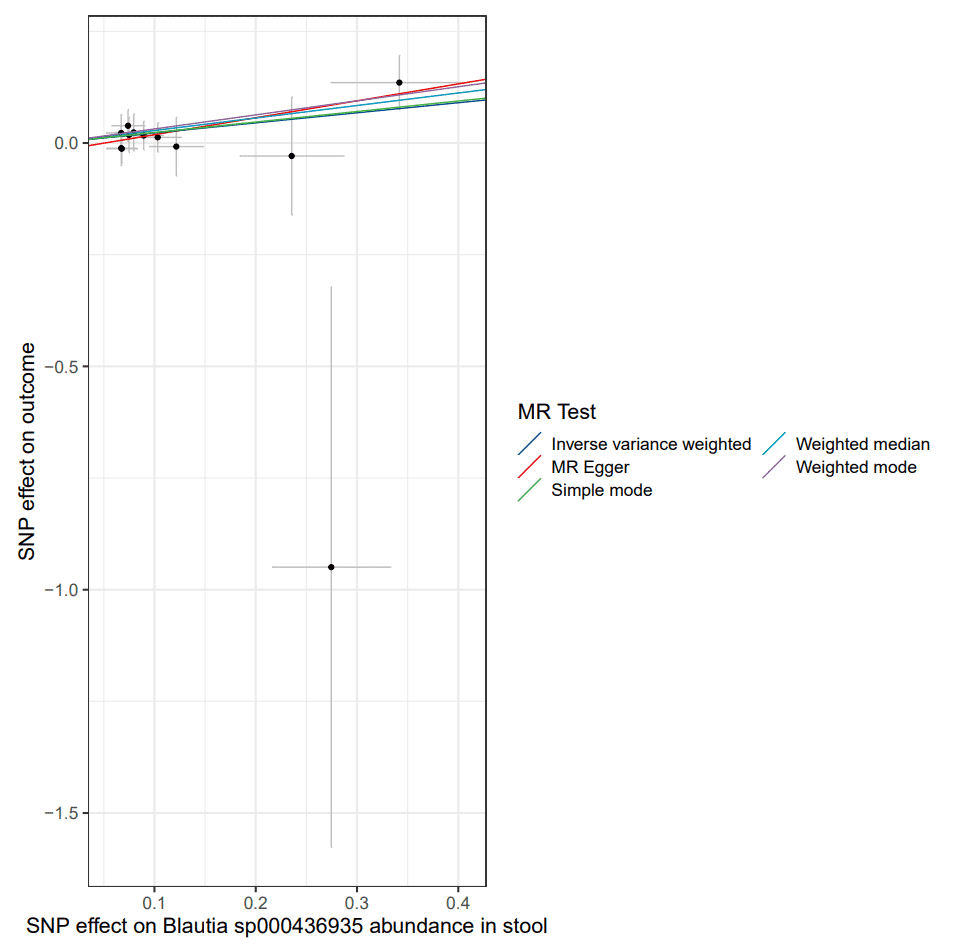

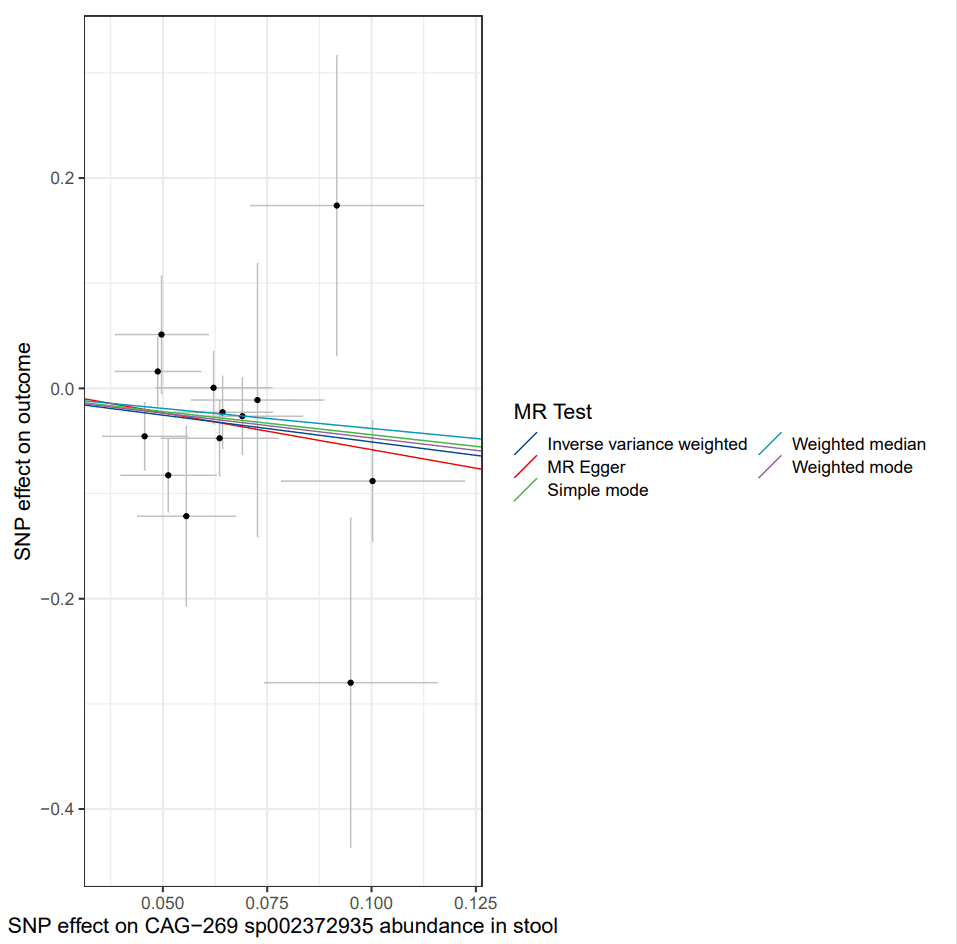


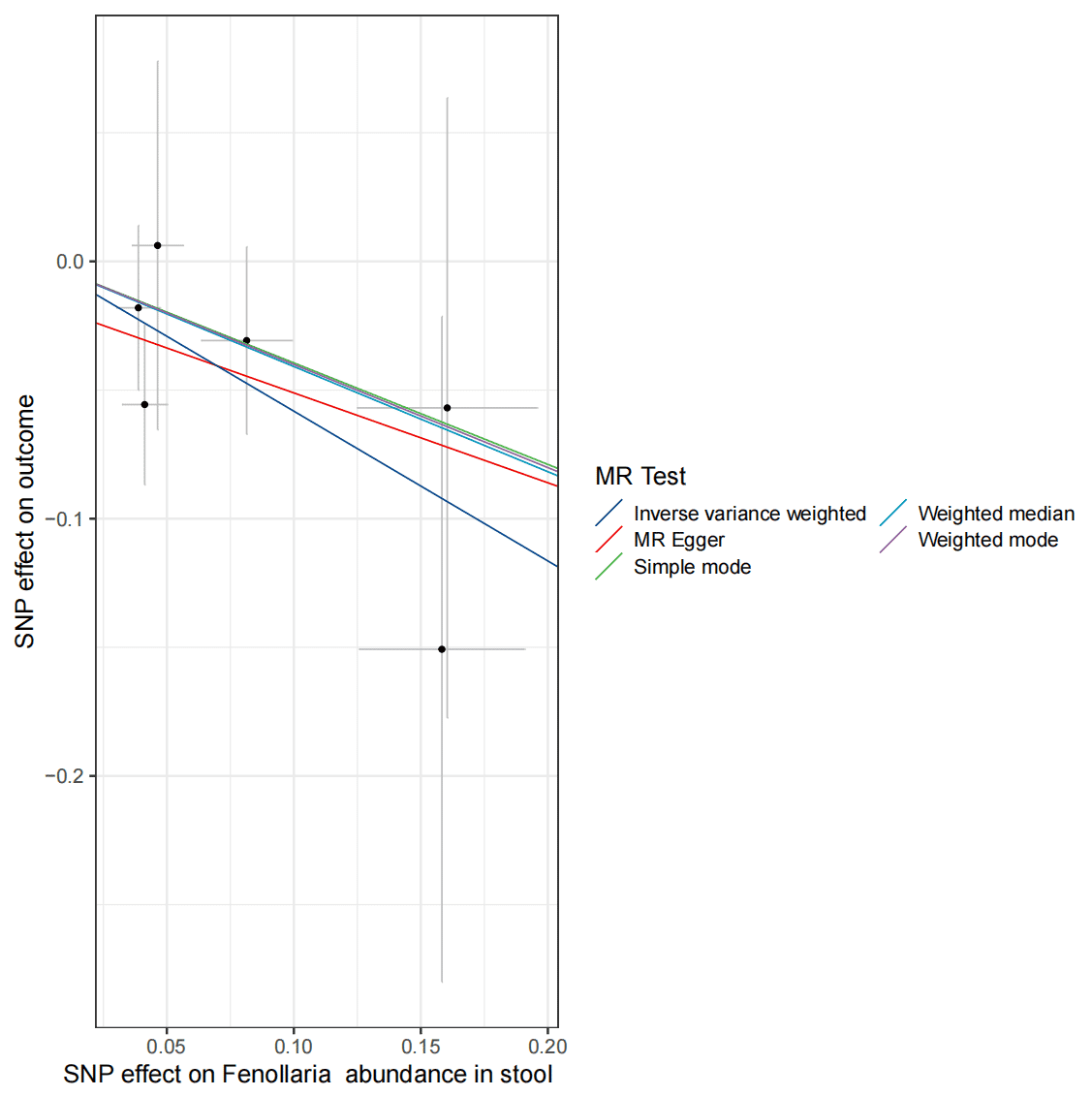

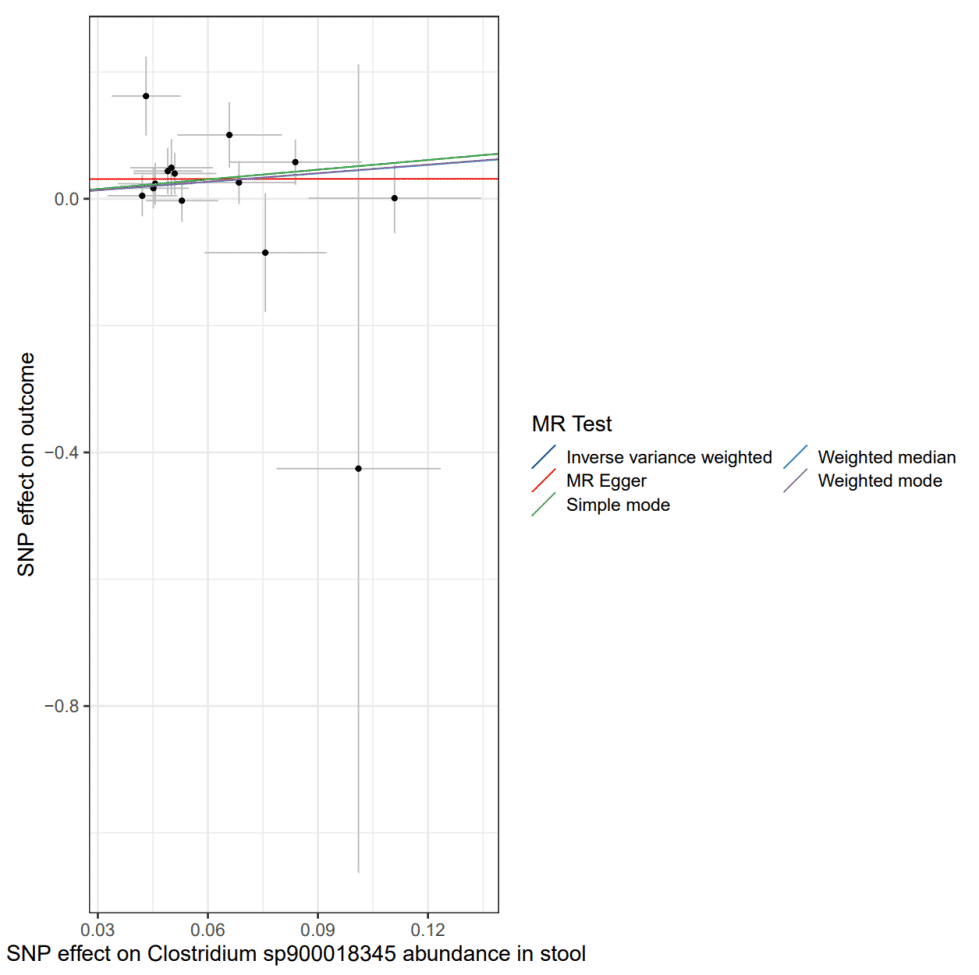

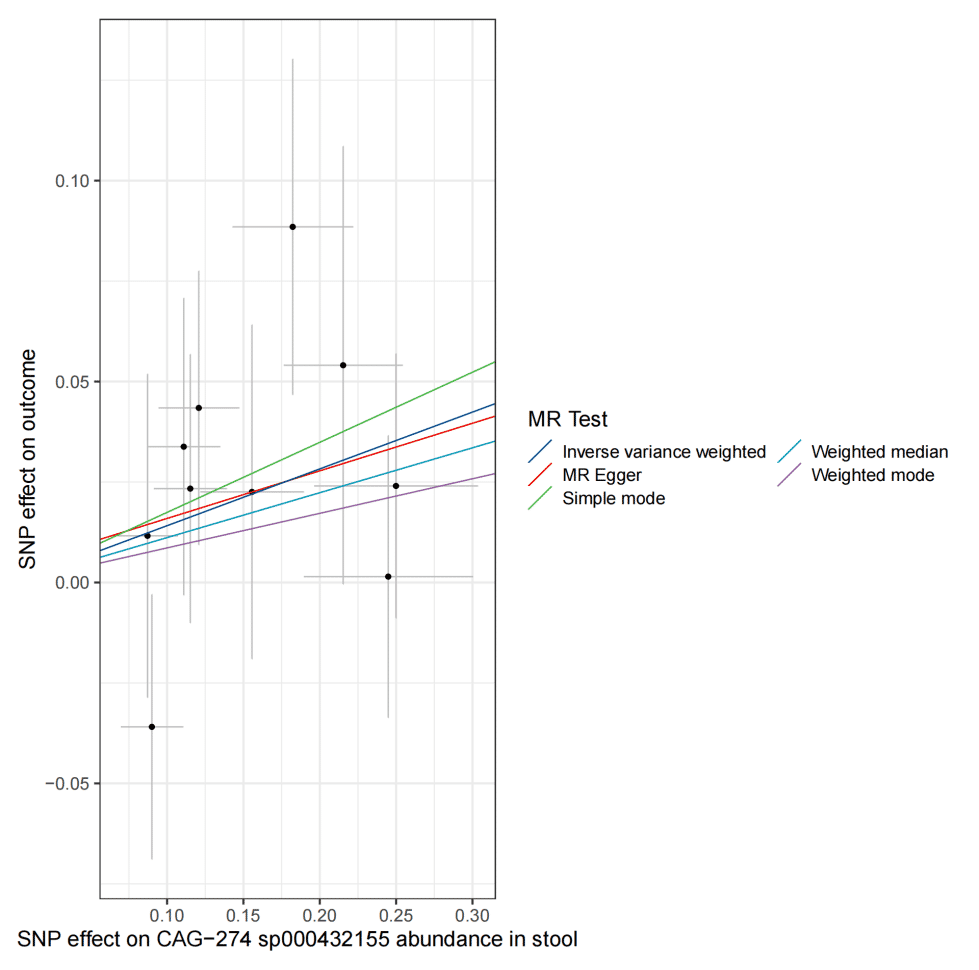


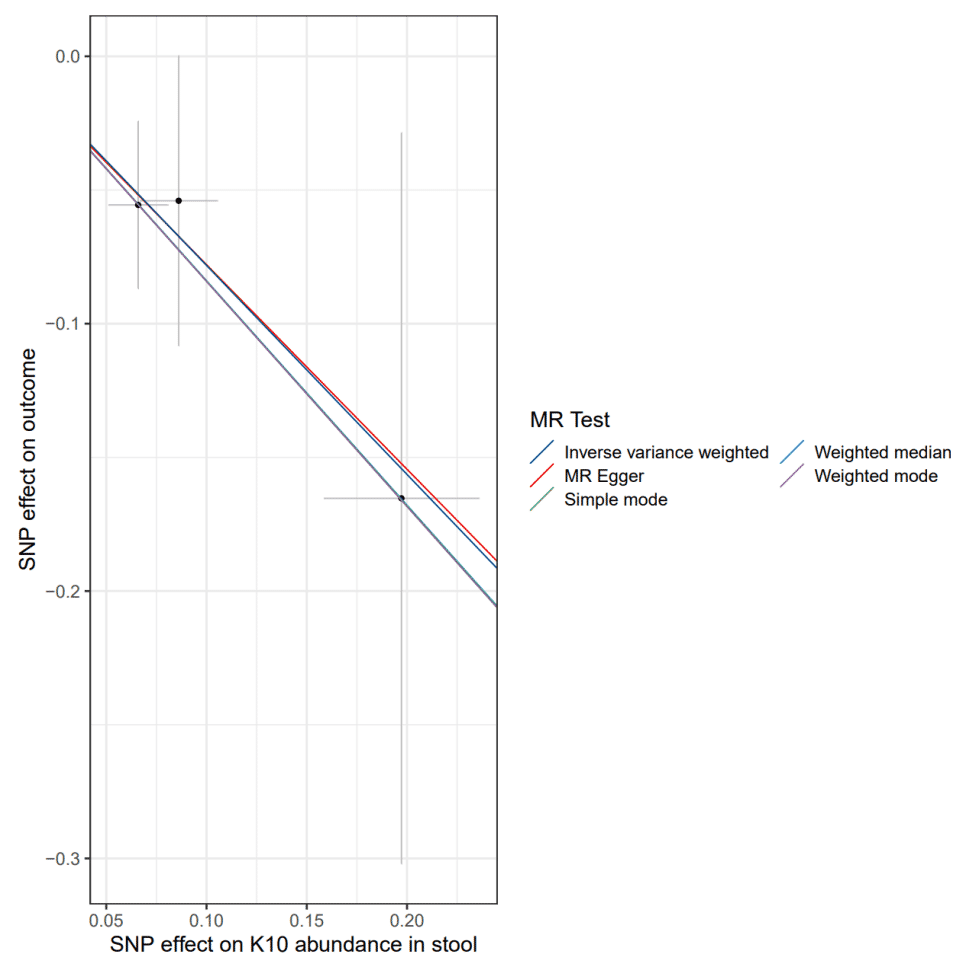

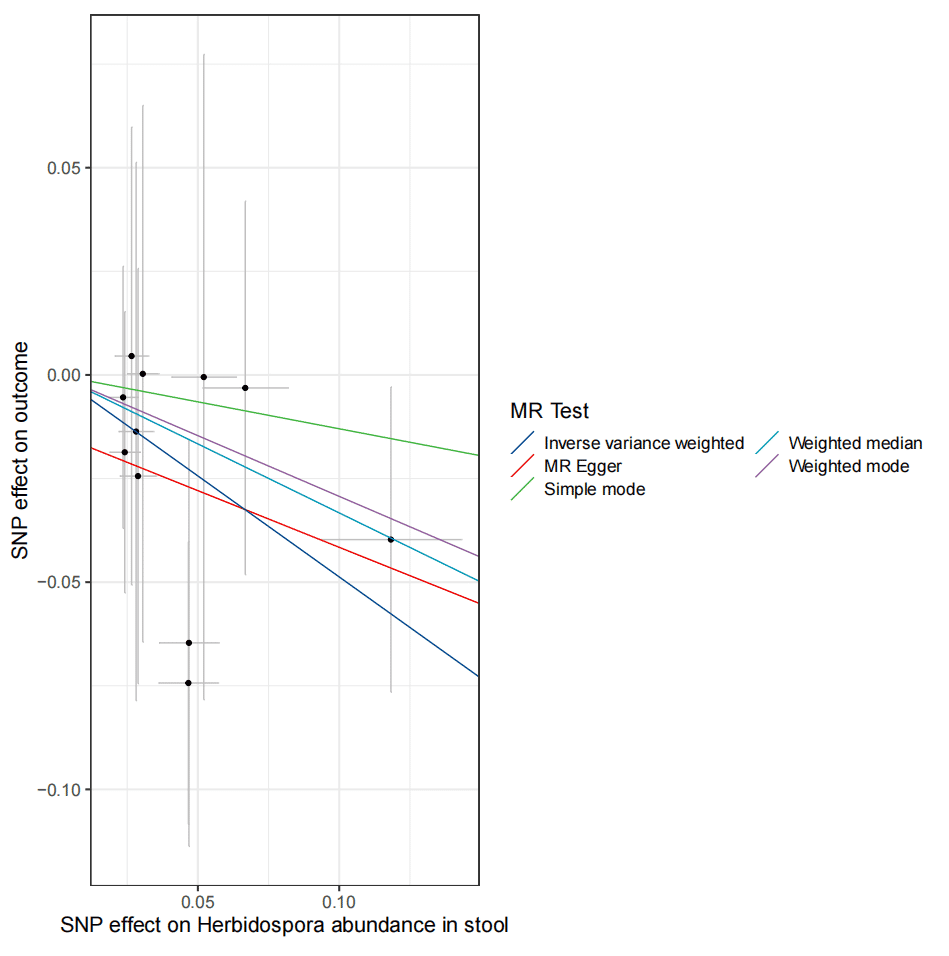

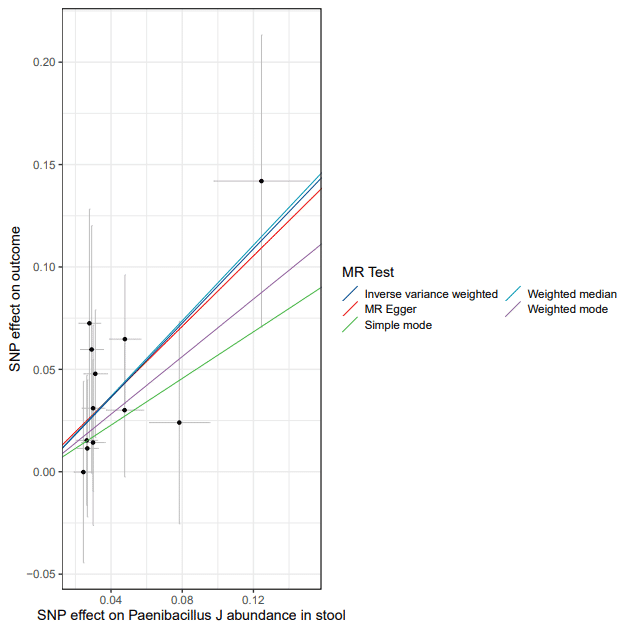


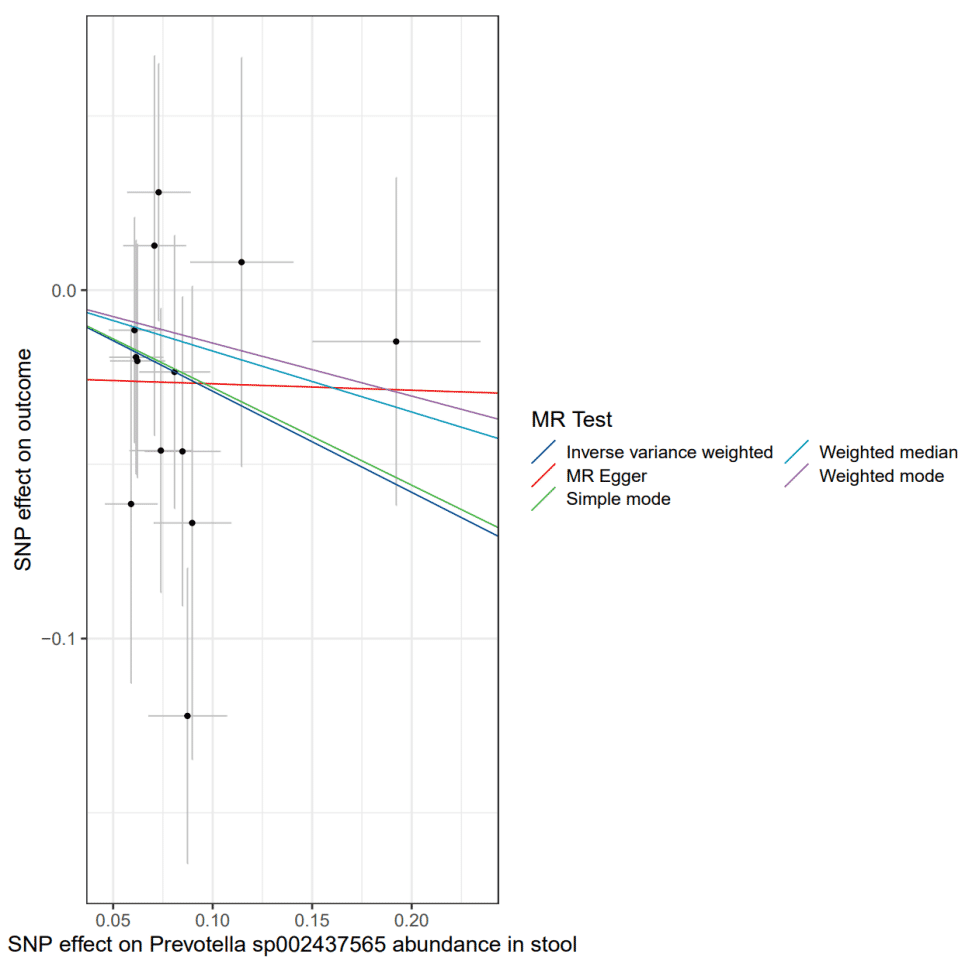

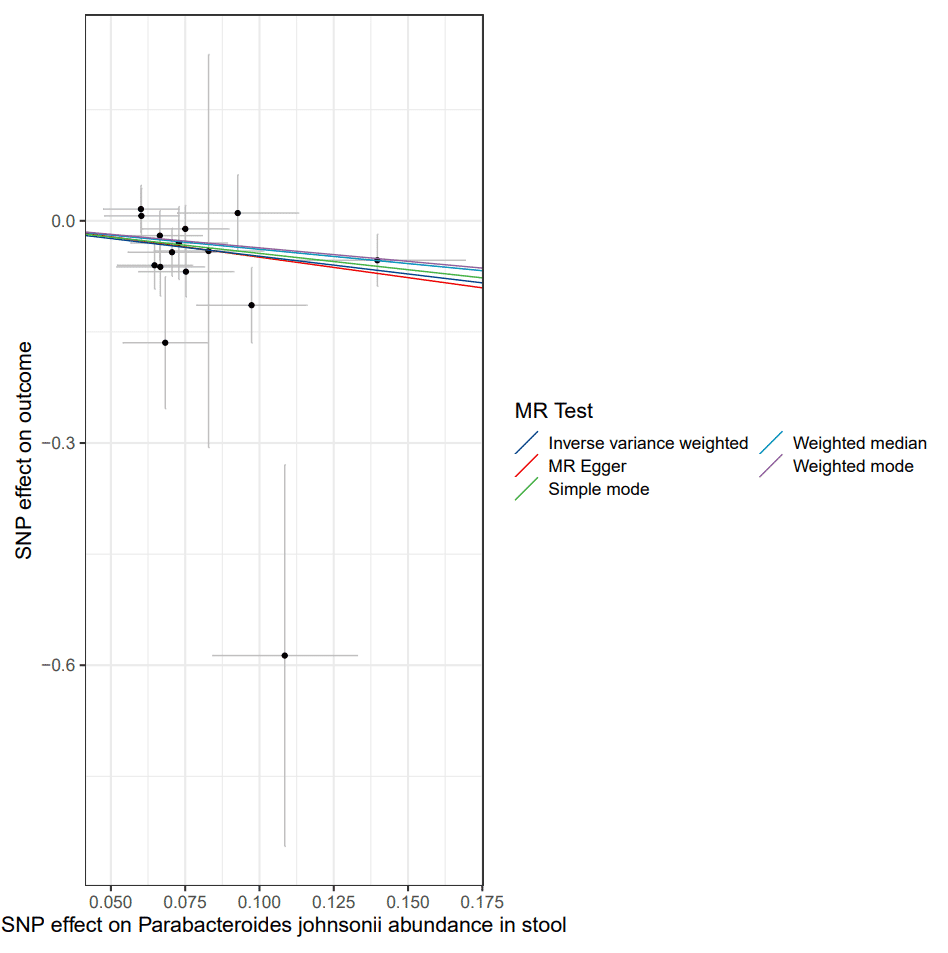

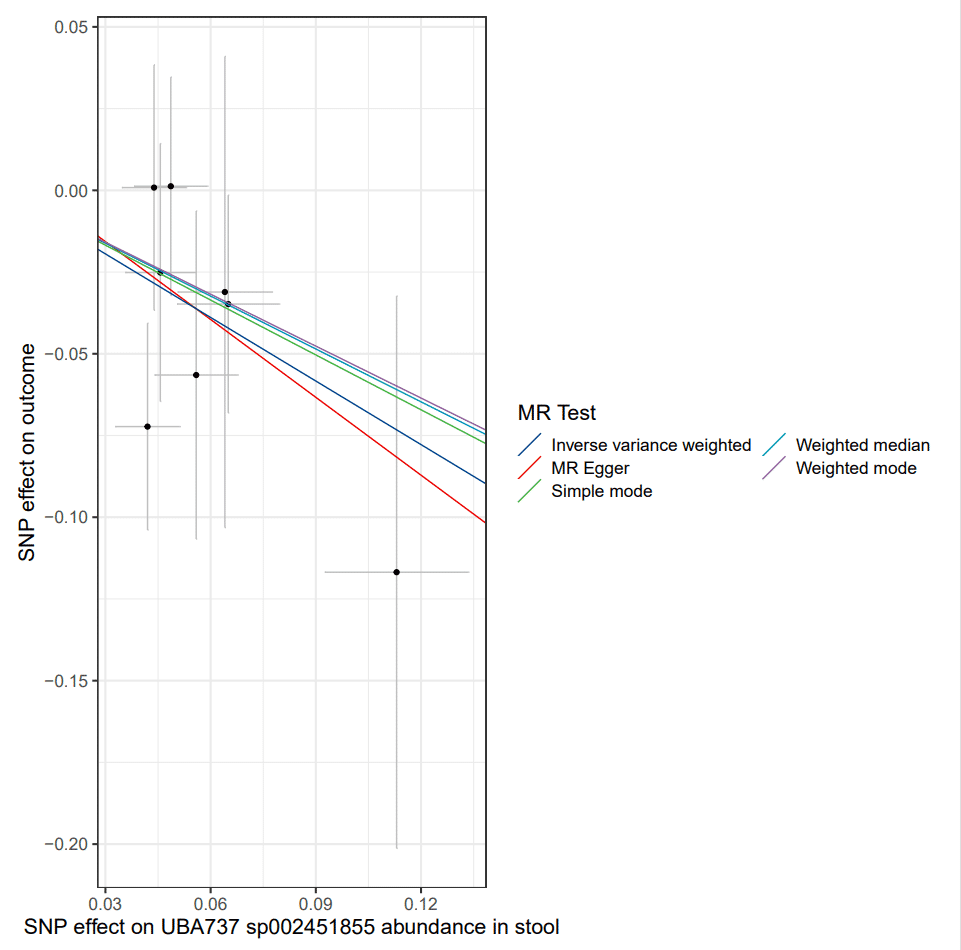


**Supplementary Figure 8: A scatter plot demonstrates causal relationship between lipids and CKD.(A),Phosphatidylcholine (14:0_18:2) (B),Phosphatidylcholine (18:2_20:1) (C),Phosphatidylcholine (O-18:0_16:1) (D),Phosphatidylcholine (O-18:2_20:4) (E),Sphingomyelin (d32:1) (F),Triacylglycerol (54:6).**


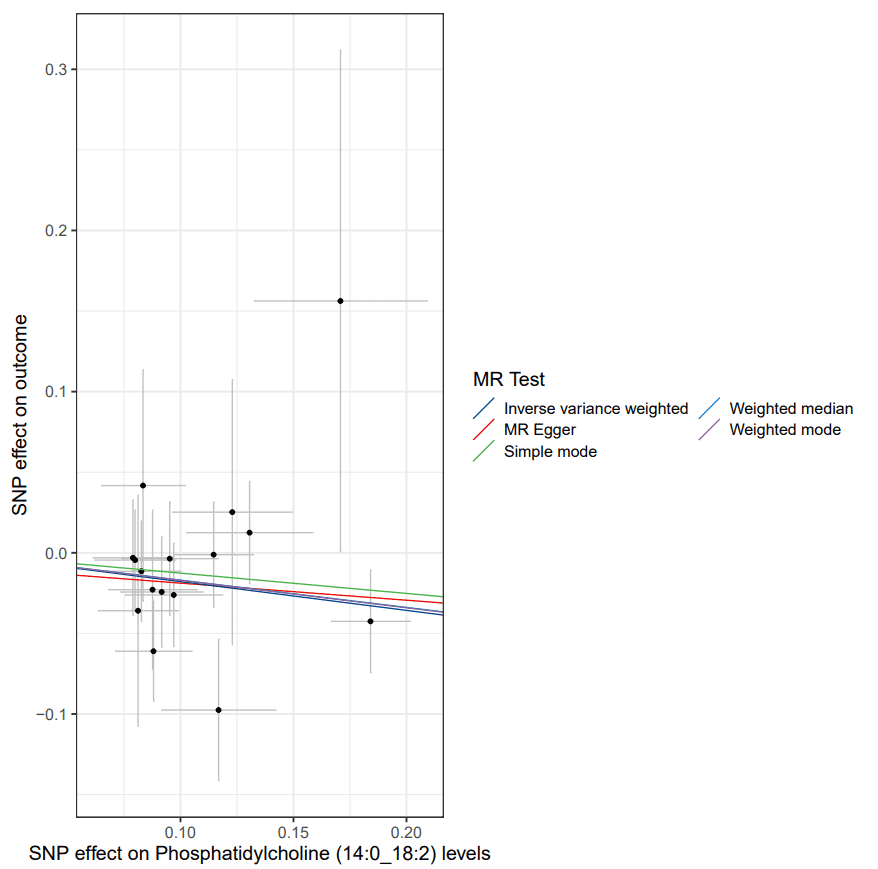

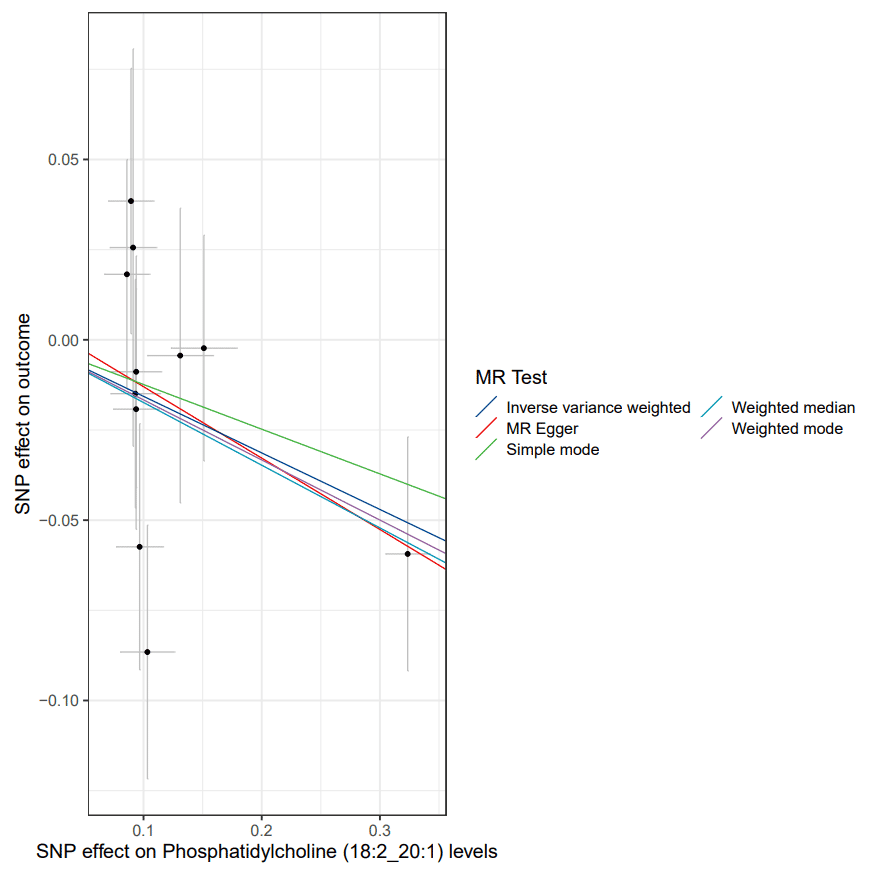

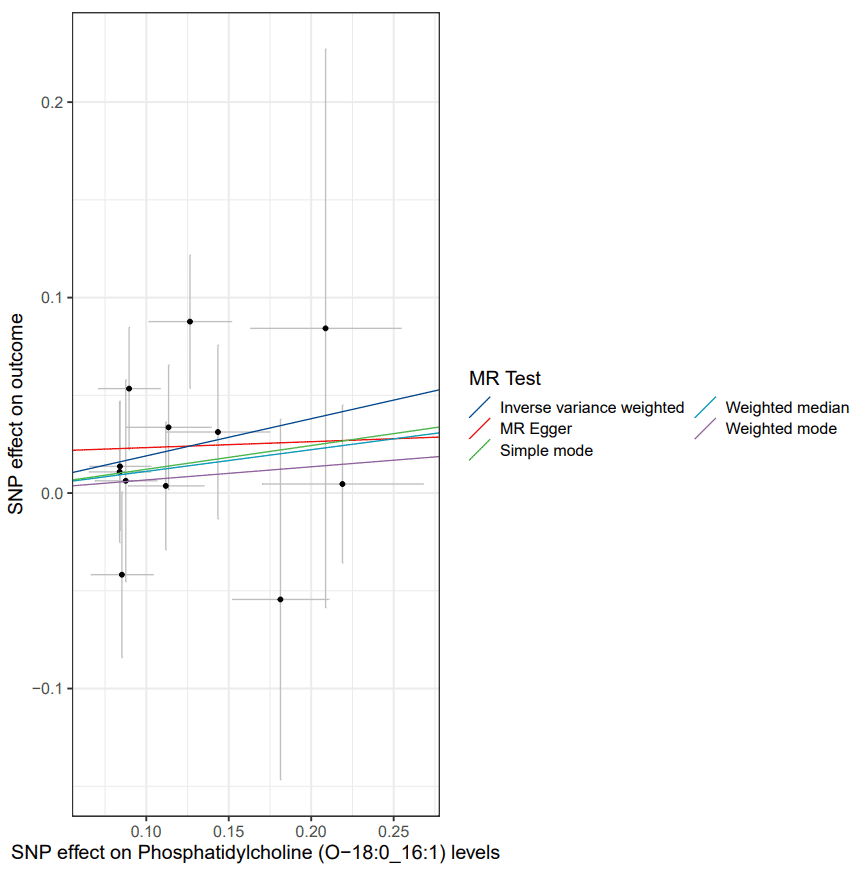


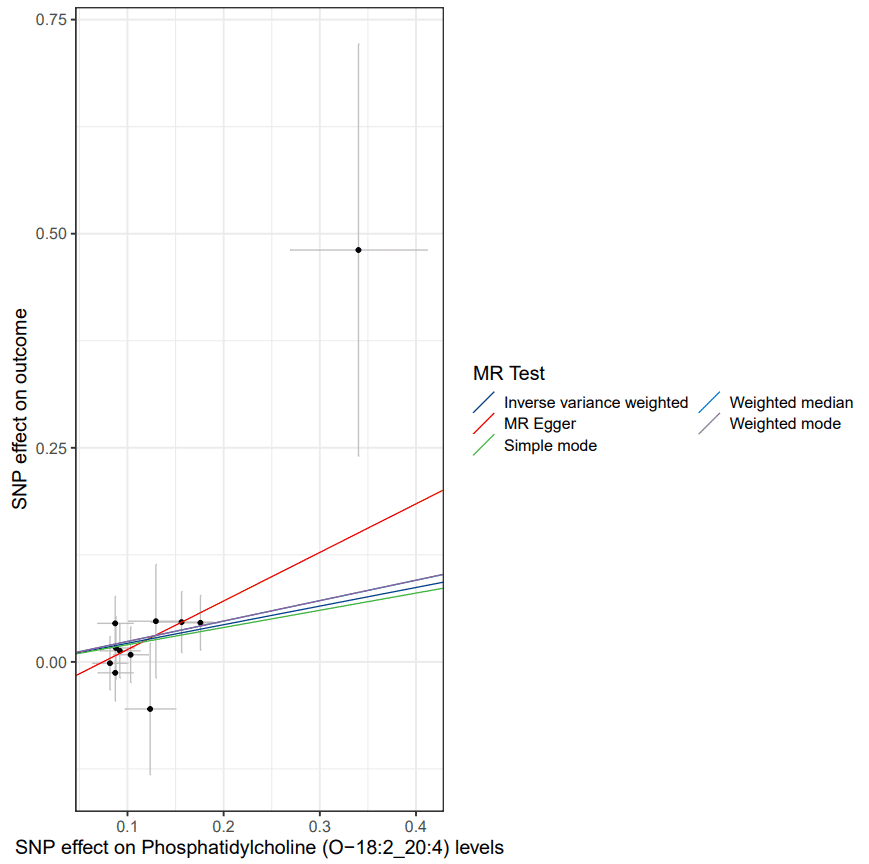

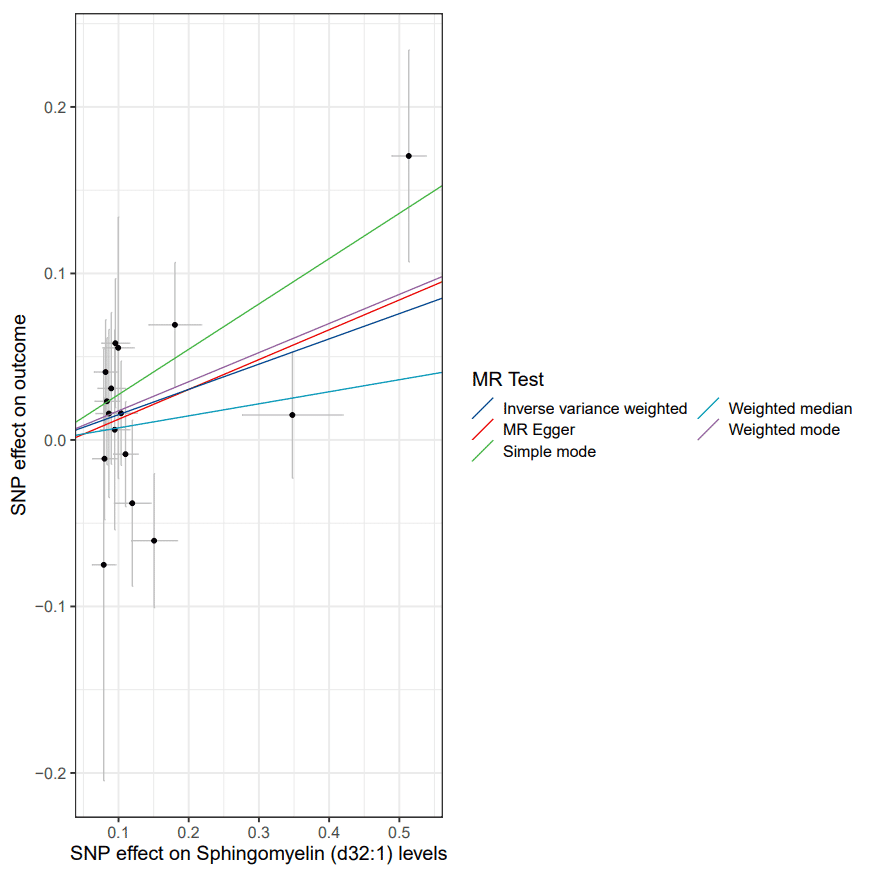

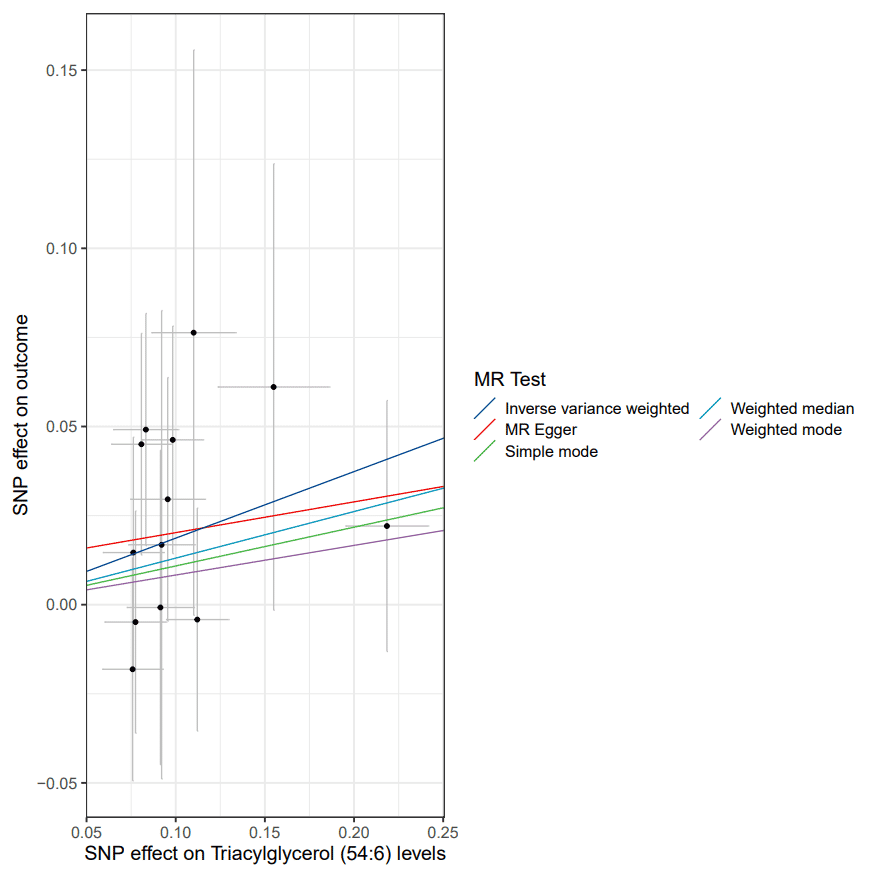


**Supplementary Figure 9:Funnel plot of causal effects of gut microbiota (A), Bifidobacterium spp (B),Blautia spp (C),CAG-269 spp (D),Eubacterium CAG-274 (E),Clostridium spp (F),Fenollaria spp (G),Actinobacteria spp (H),K10 spp (I),Paenibacillus J spp (J),Parabacteroides johnsonii (K),Prevotella spp (L),Rumenococcaceae UBA737.**


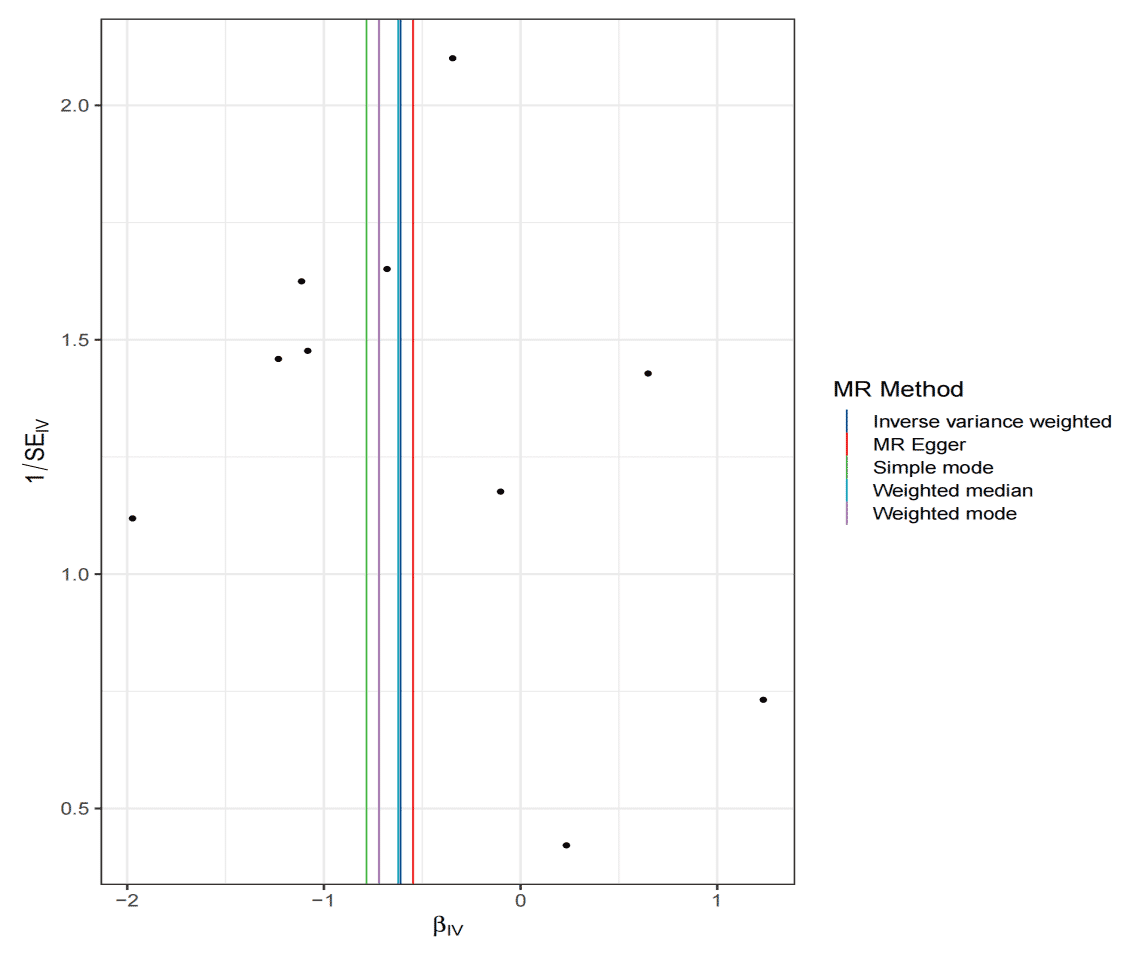

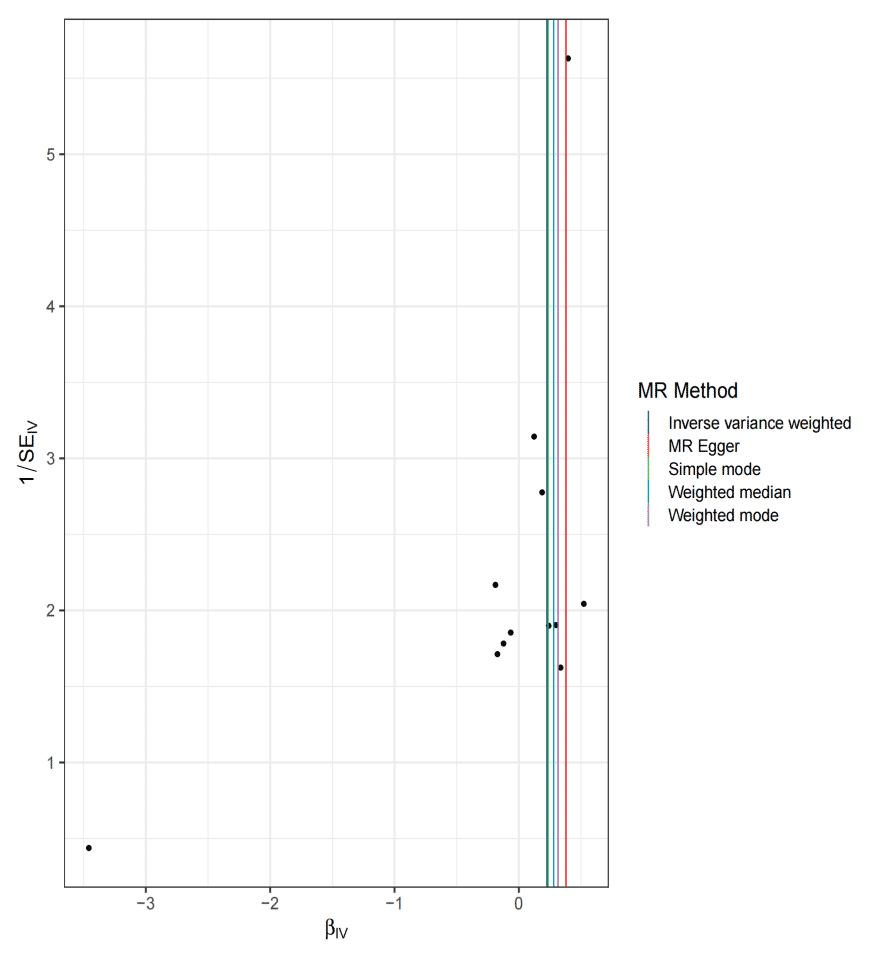

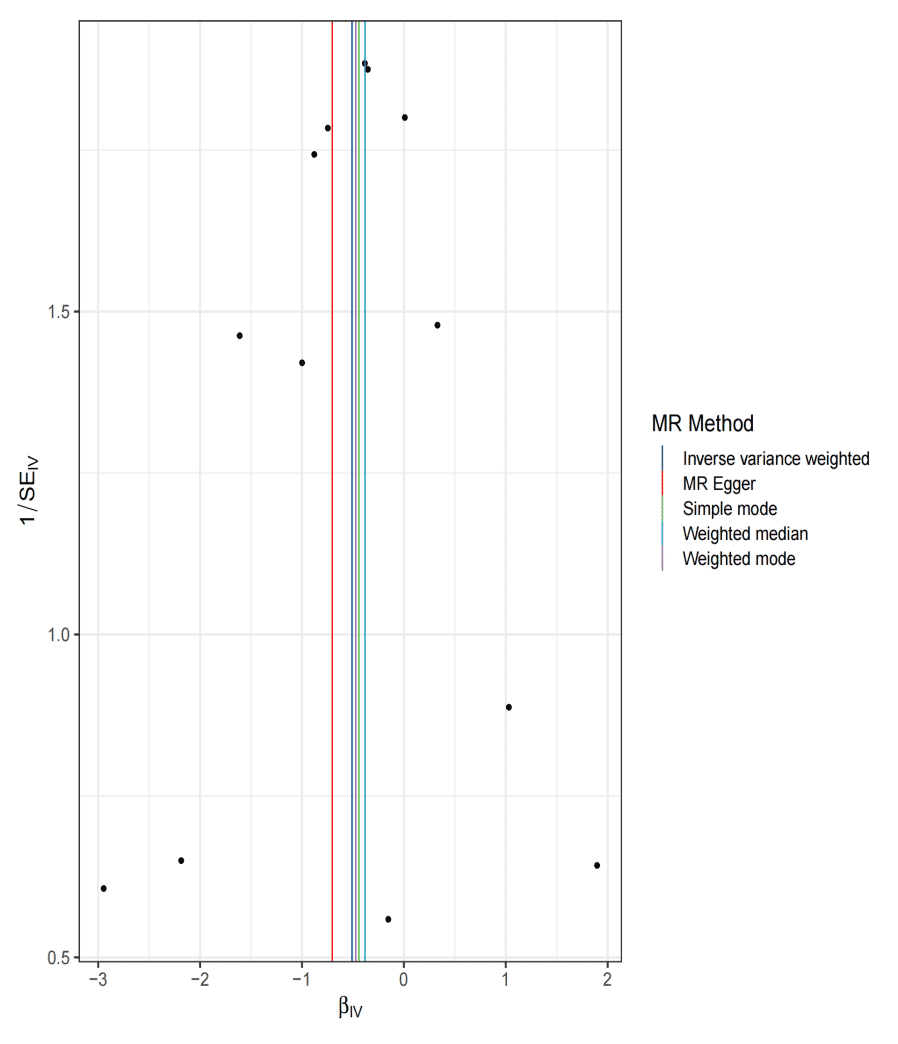


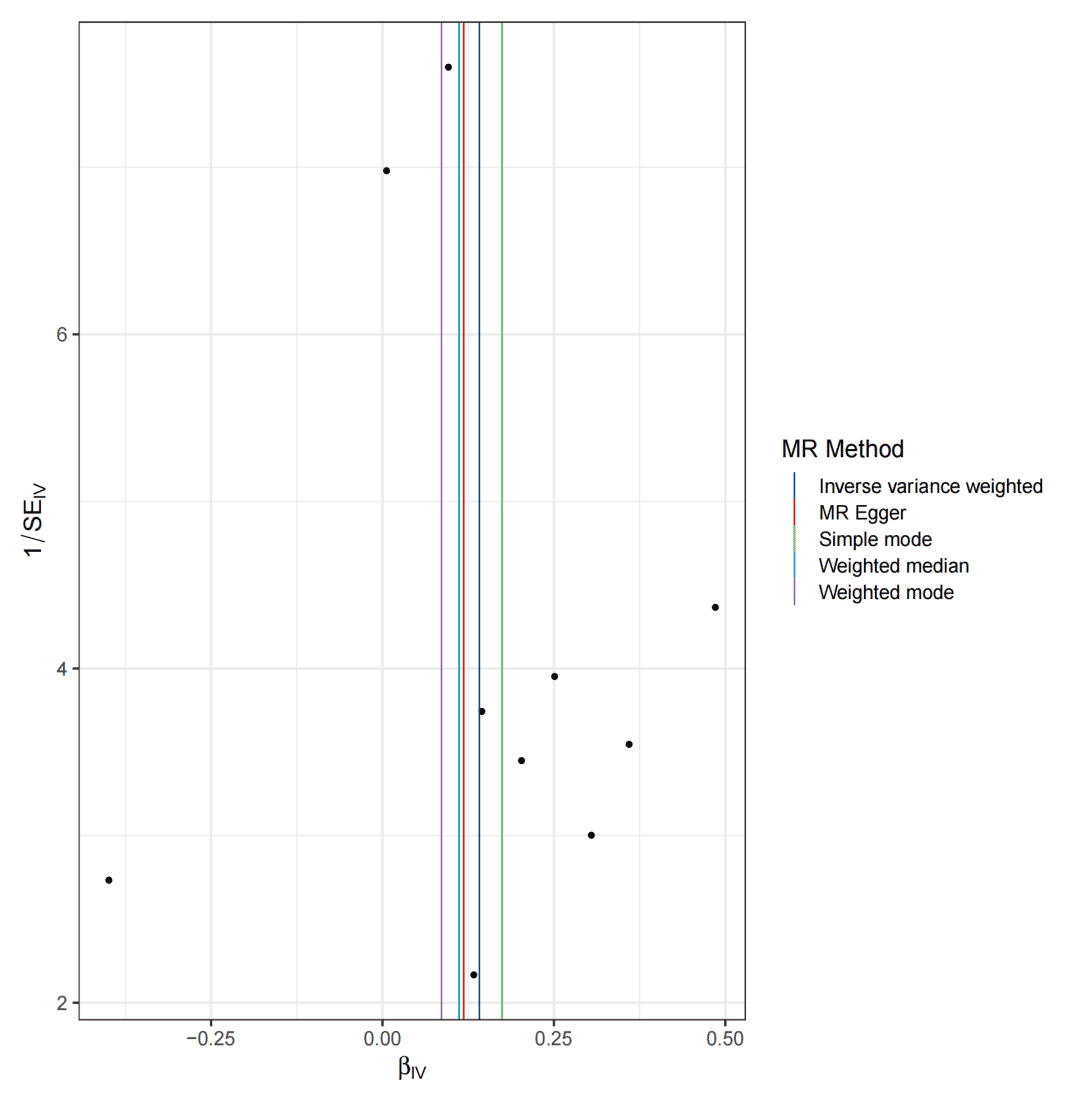

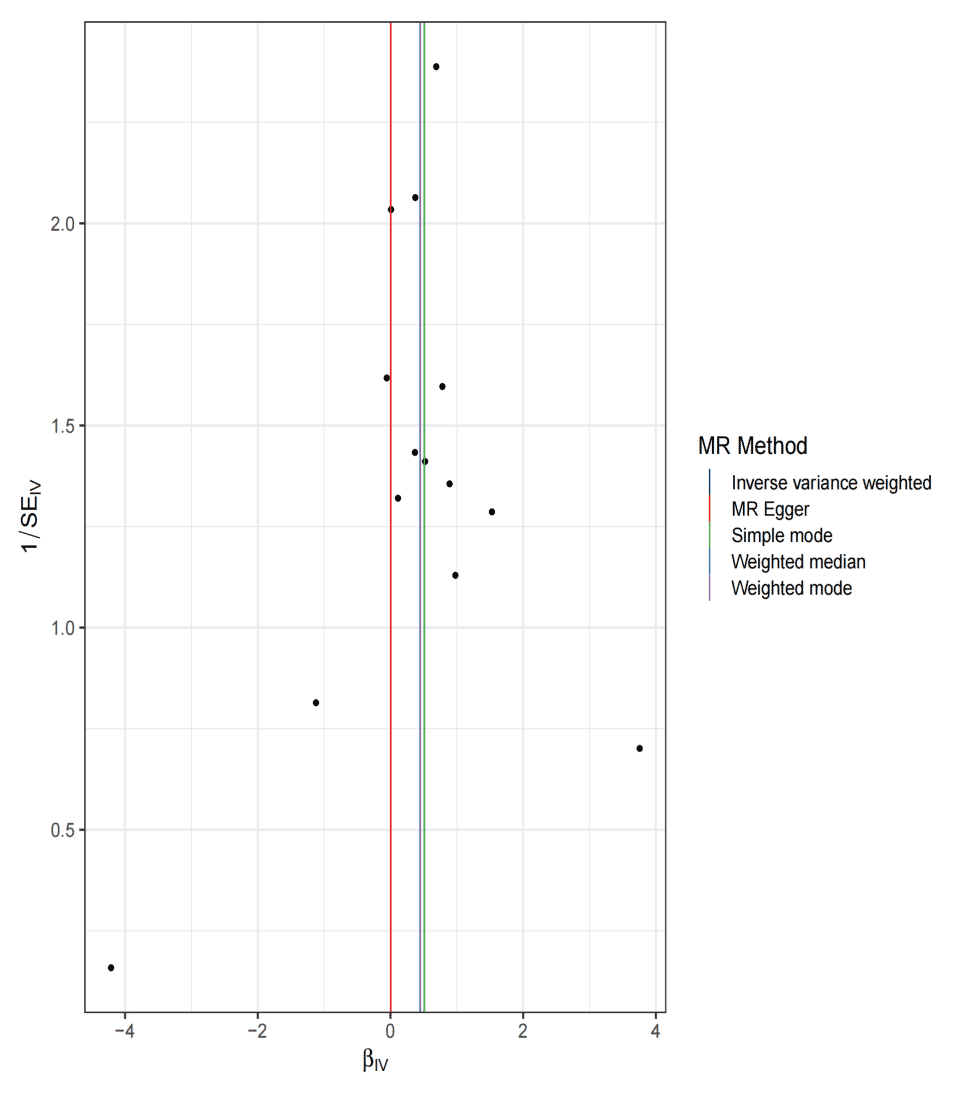

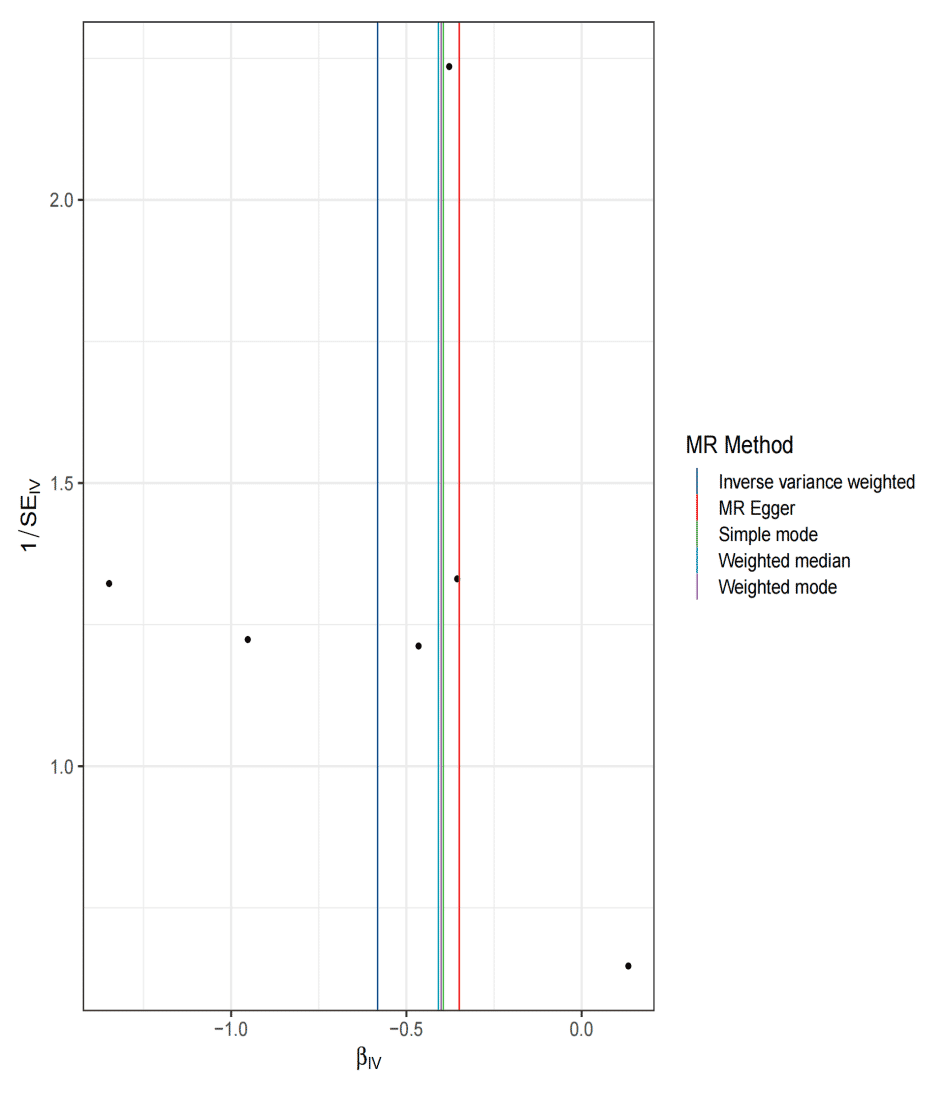


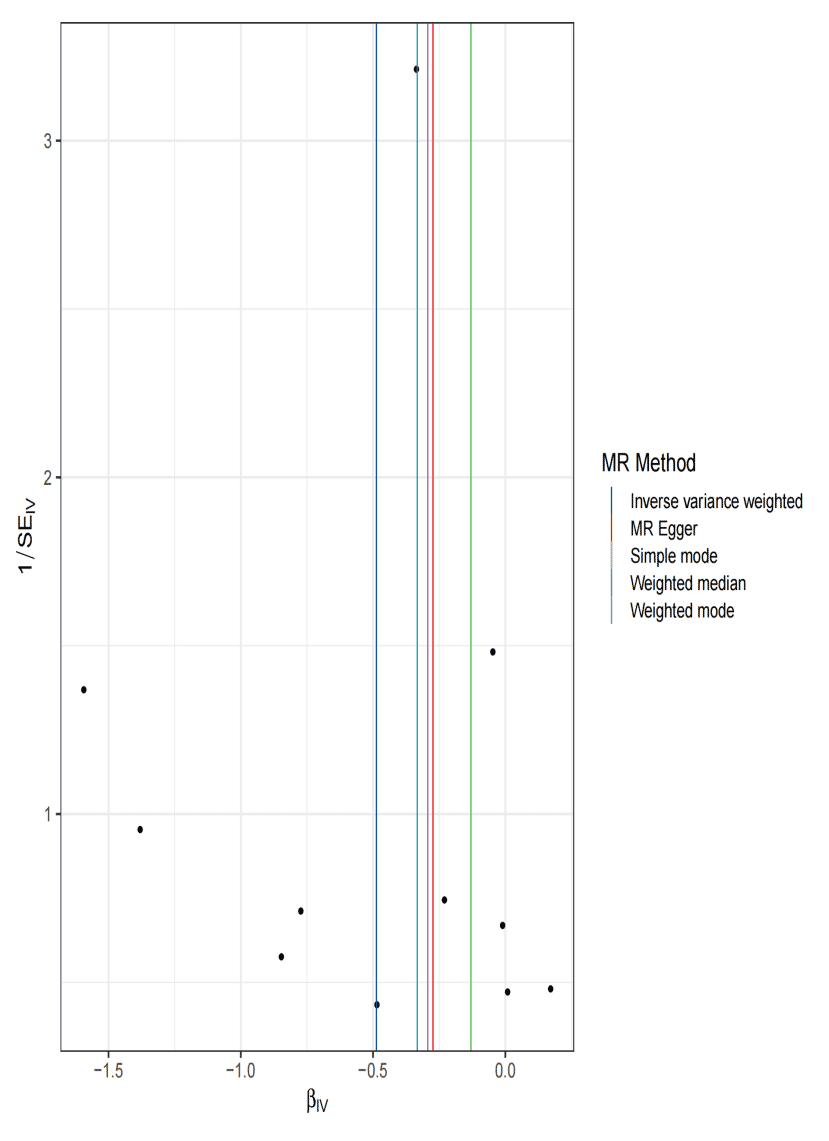

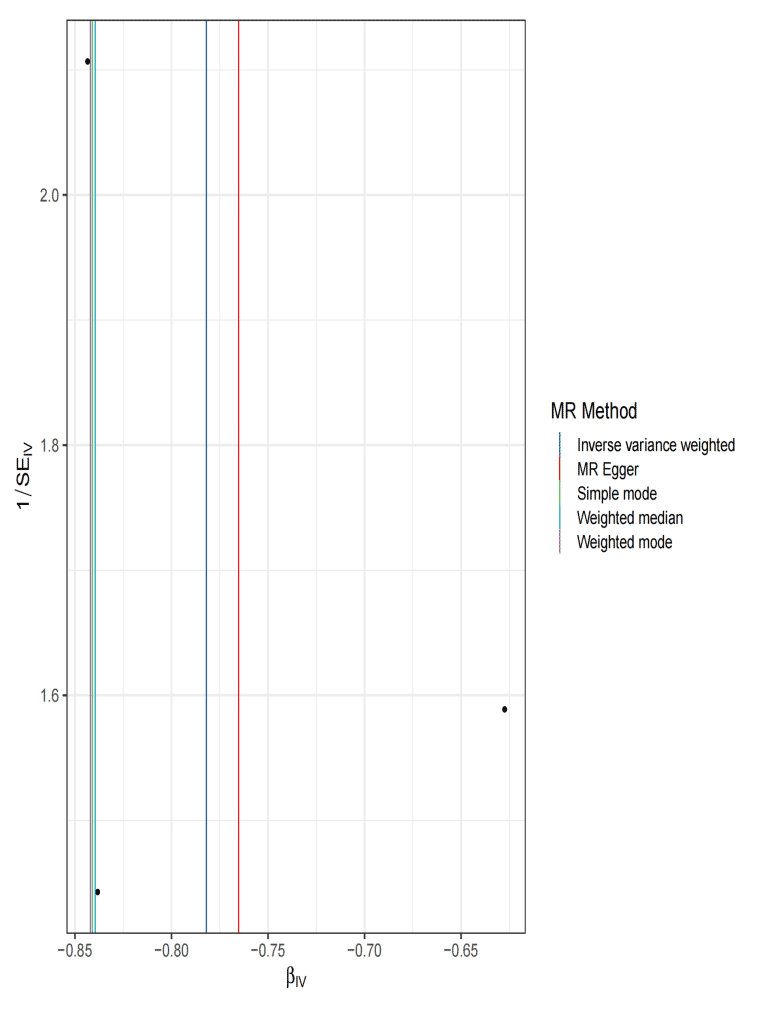

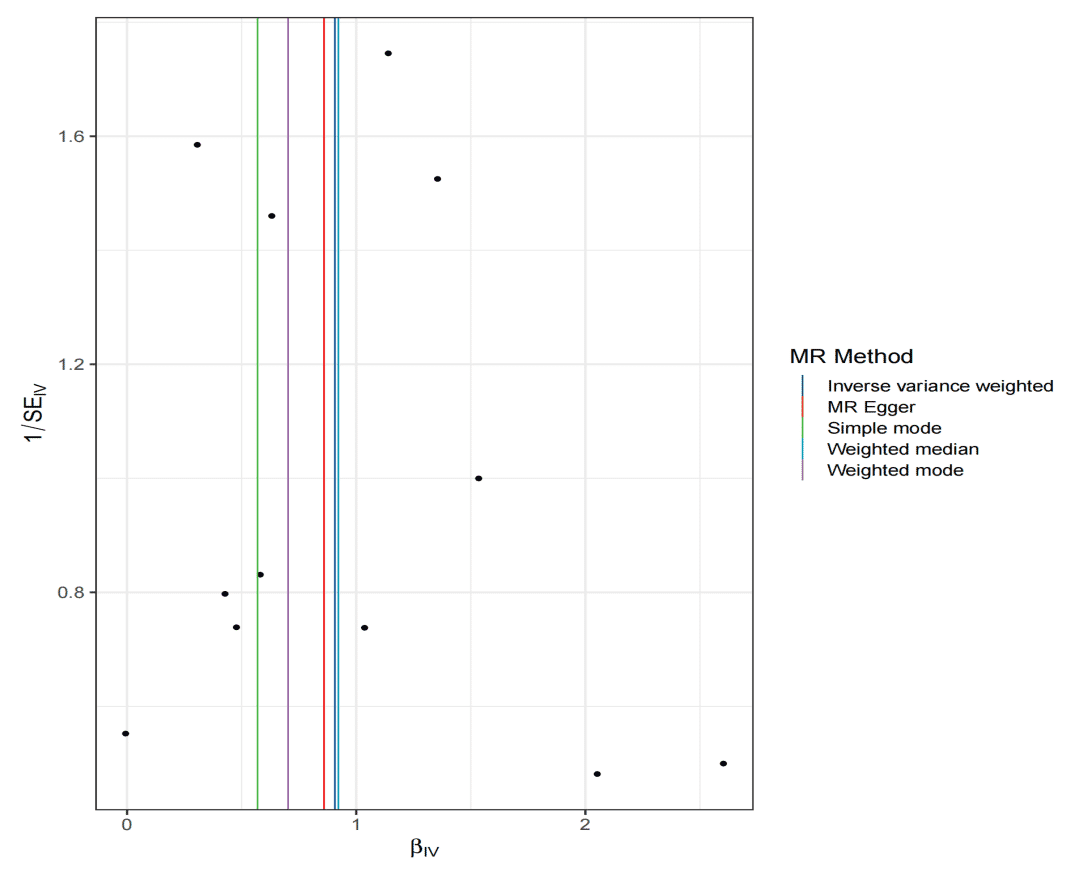


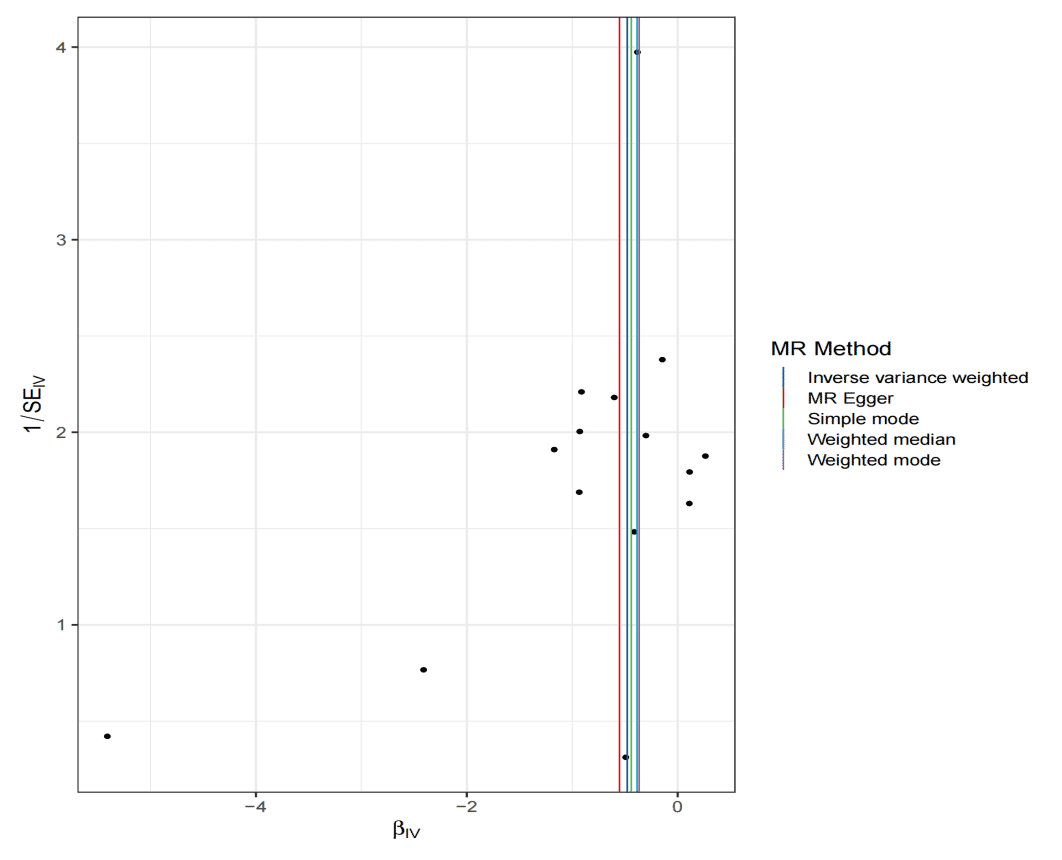

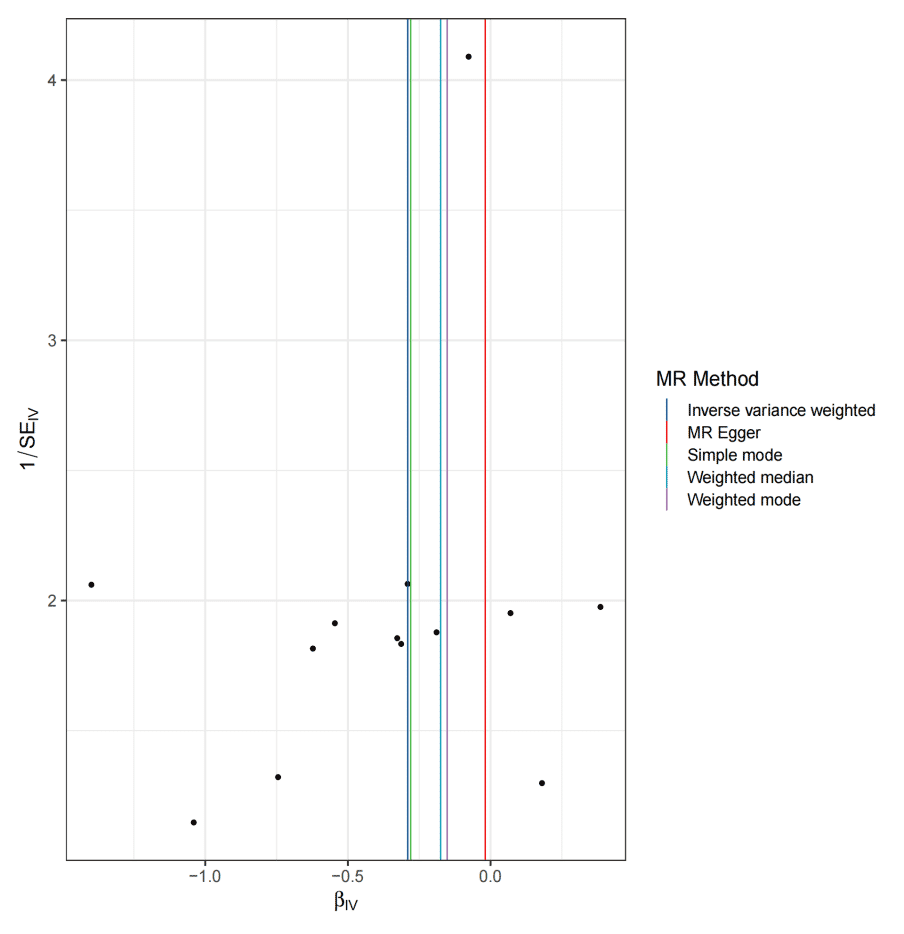

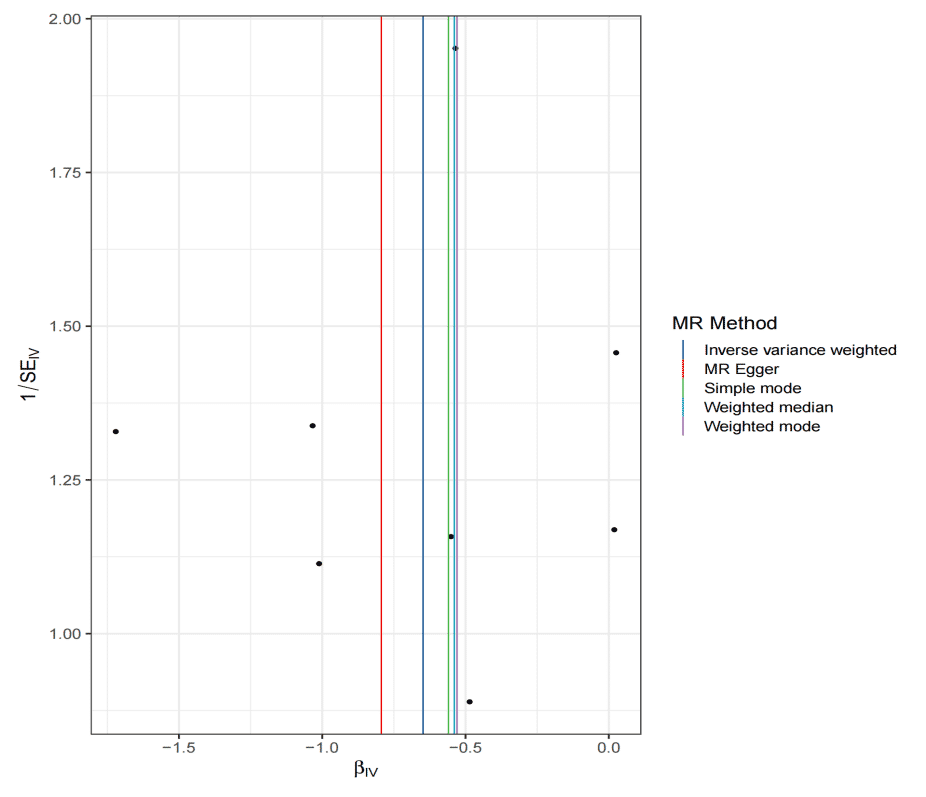


**Supplementary Figure 10:Funnel plot of causal effects of lipid (A),Phosphatidylcholine (14:0_18:2) (B),Phosphatidylcholine (18:2_20:1) (C),Phosphatidylcholine (O-18:0_16:1) (D),Phosphatidylcholine (O-18:2_20:4) (E),Sphingomyelin (d32:1) (F),Triacylglycerol (54:6).**


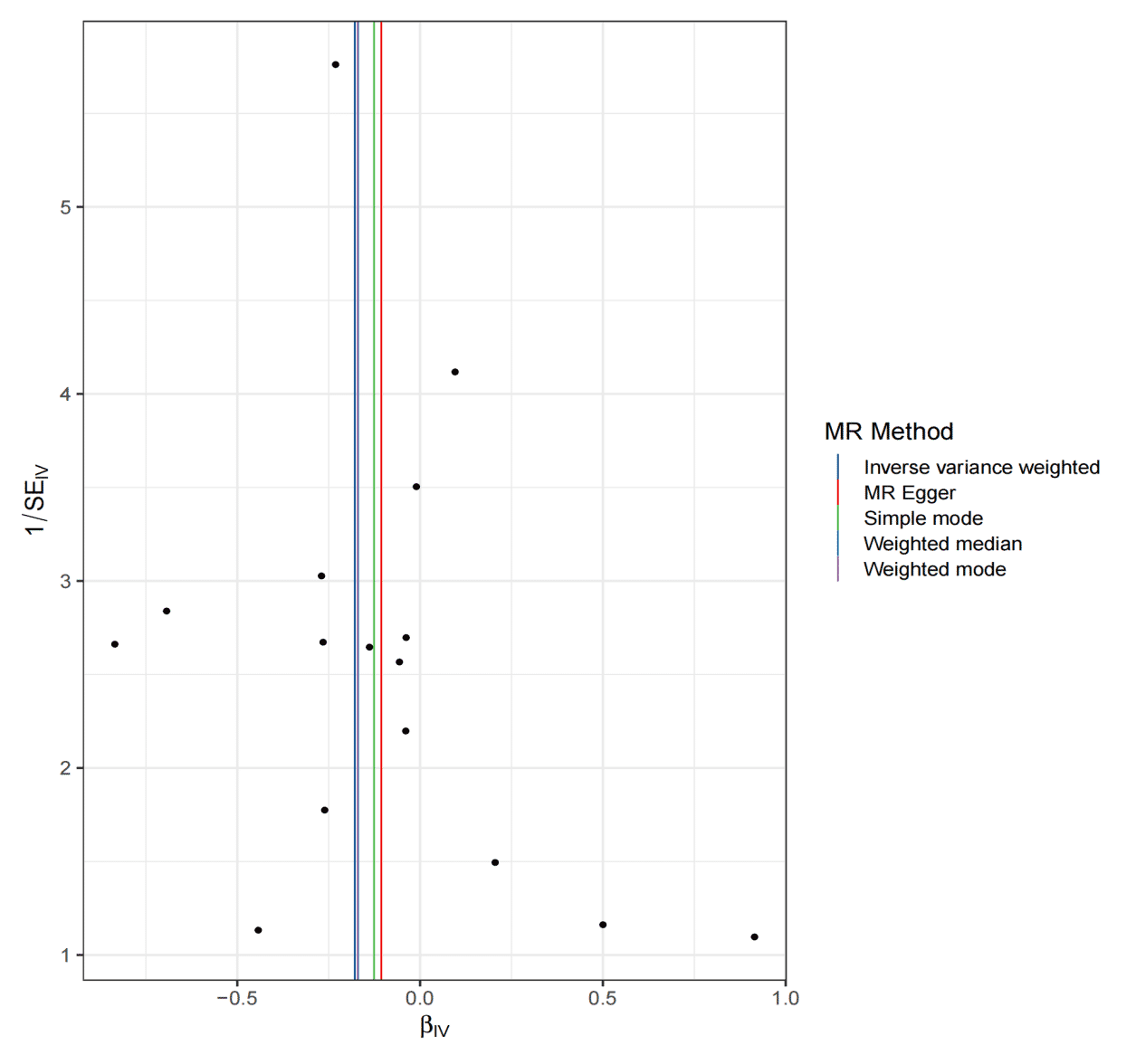

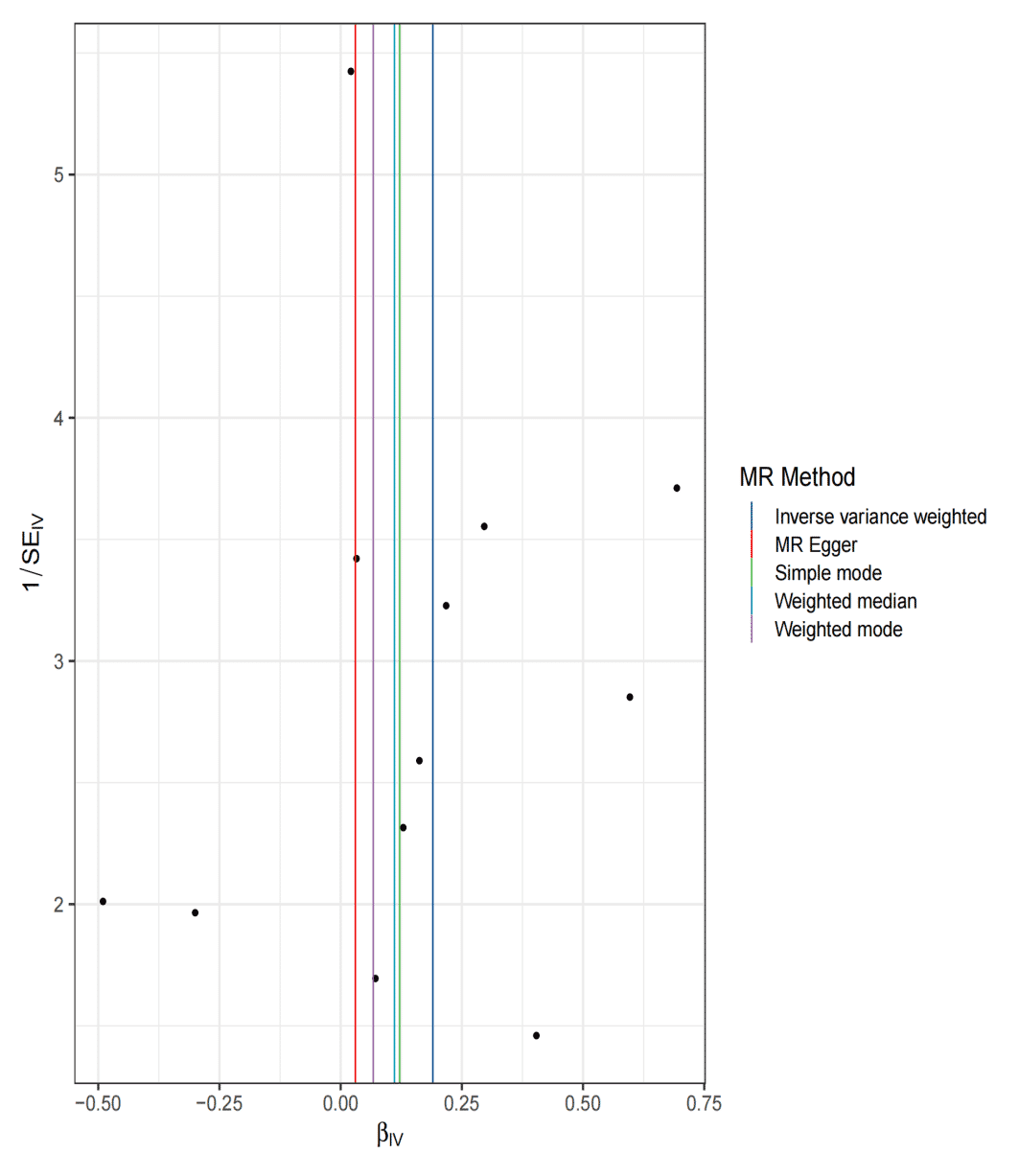

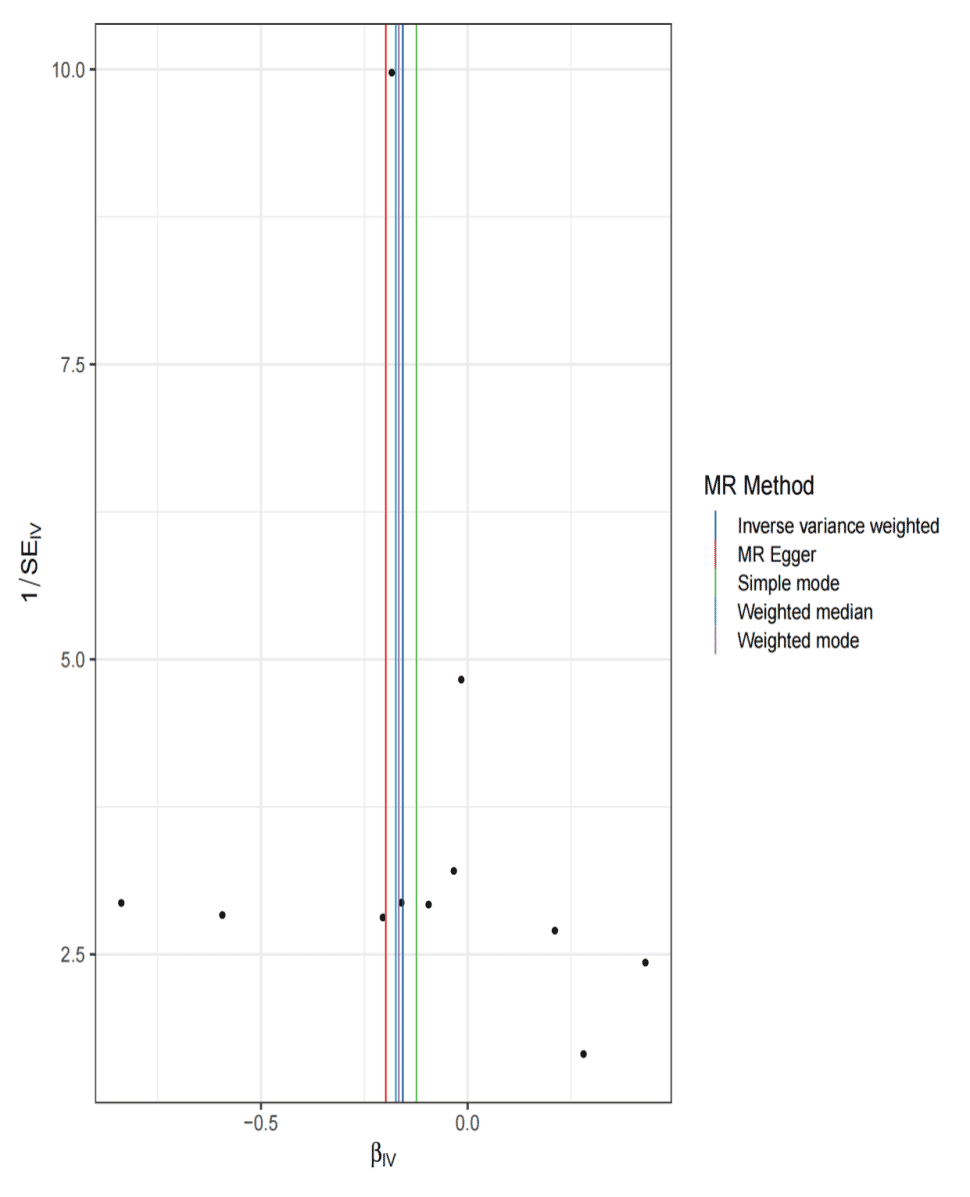


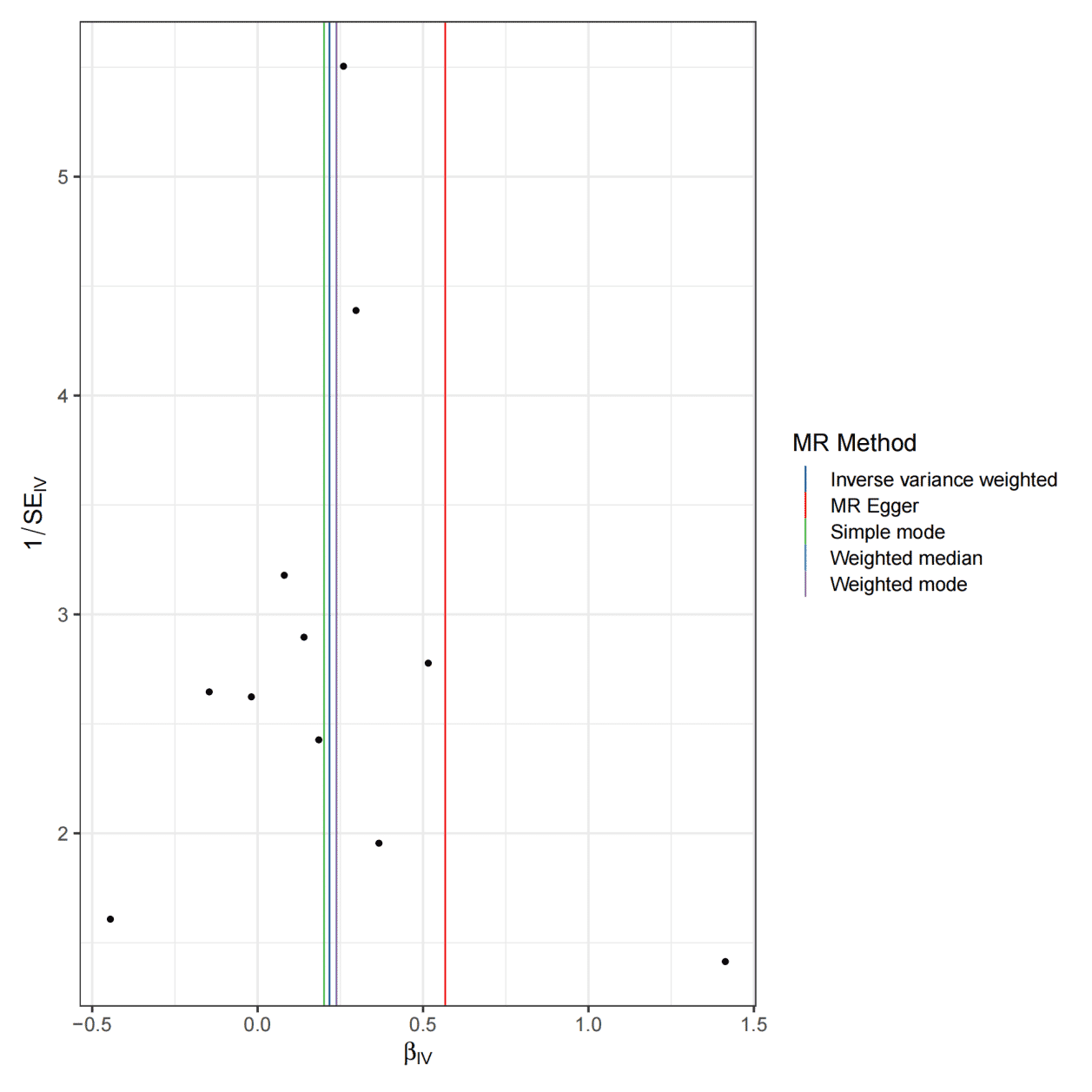

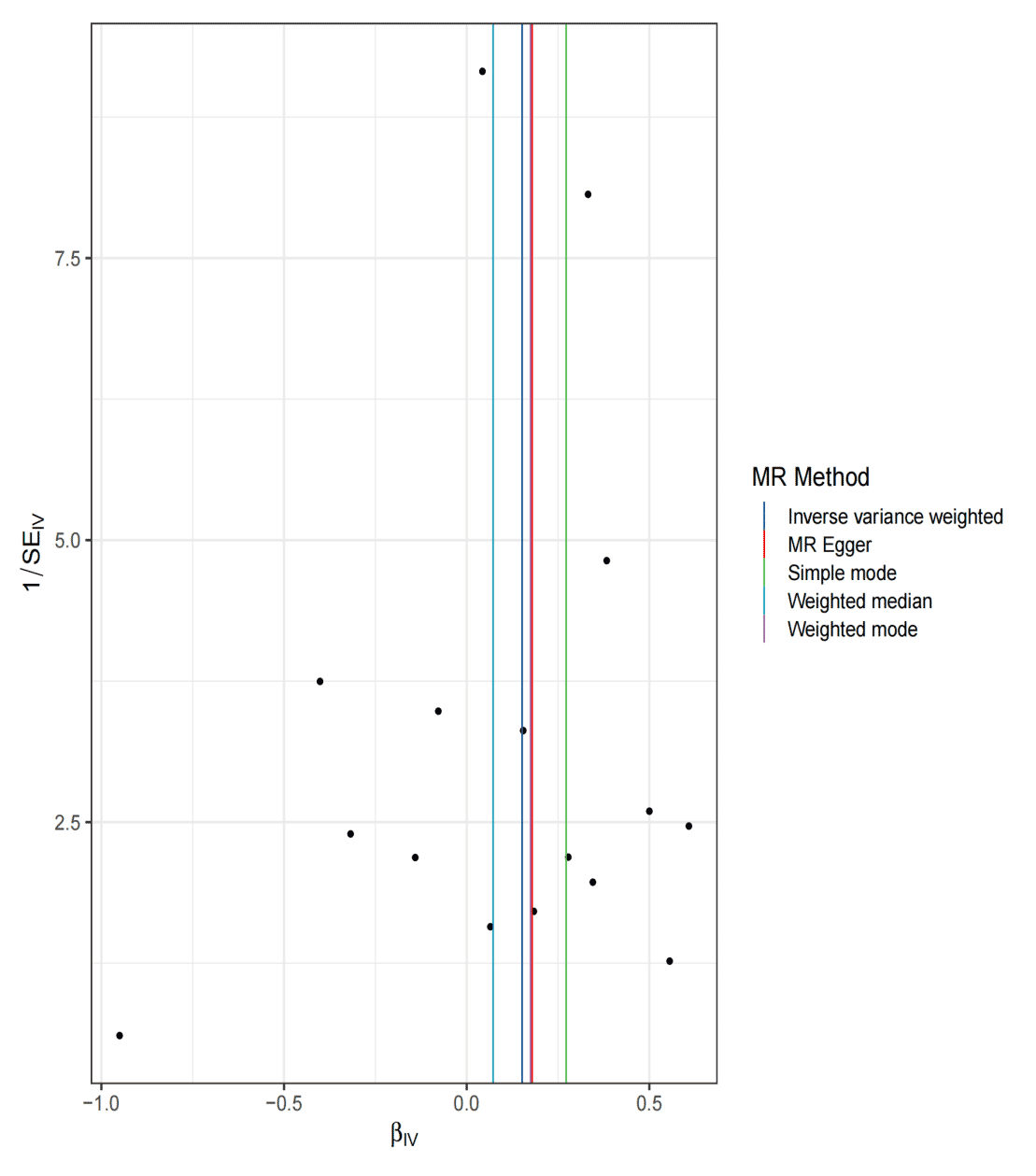

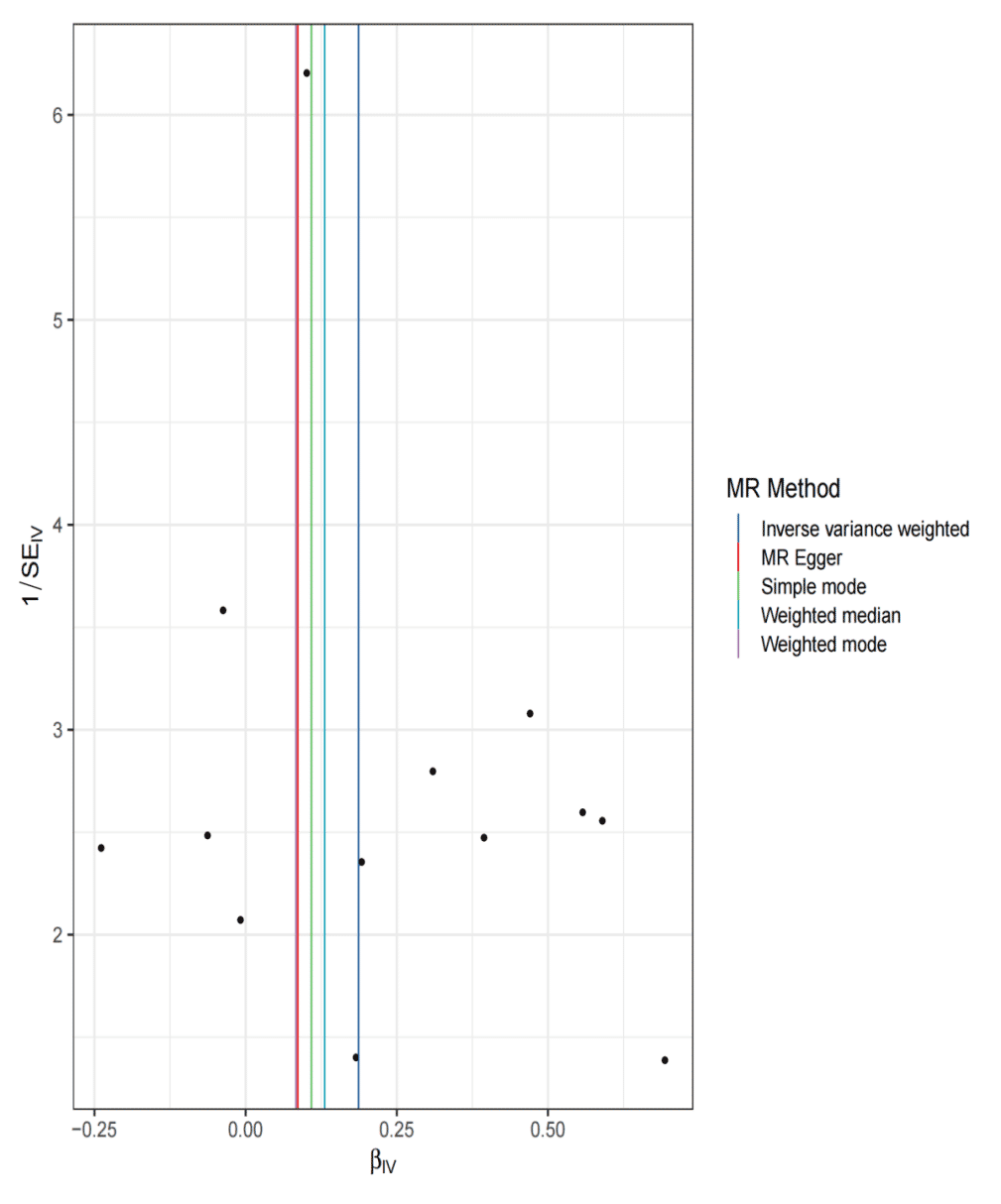


**Supplementary Figure 11: Leave-one-out analysis of the causal effect of gut microbiota(A),Bifidobacterium spp (B),Blautia spp (C),CAG-269 spp(D),Eubacterium CAG-274 (E),Clostridium spp (F),Fenollaria spp (G),Actinobacteria spp (H),K10 spp (I),Paenibacillus J spp (J),Parabacteroides johnsonii (K),Prevotella spp (L),Rumenococcaceae UBA73.**


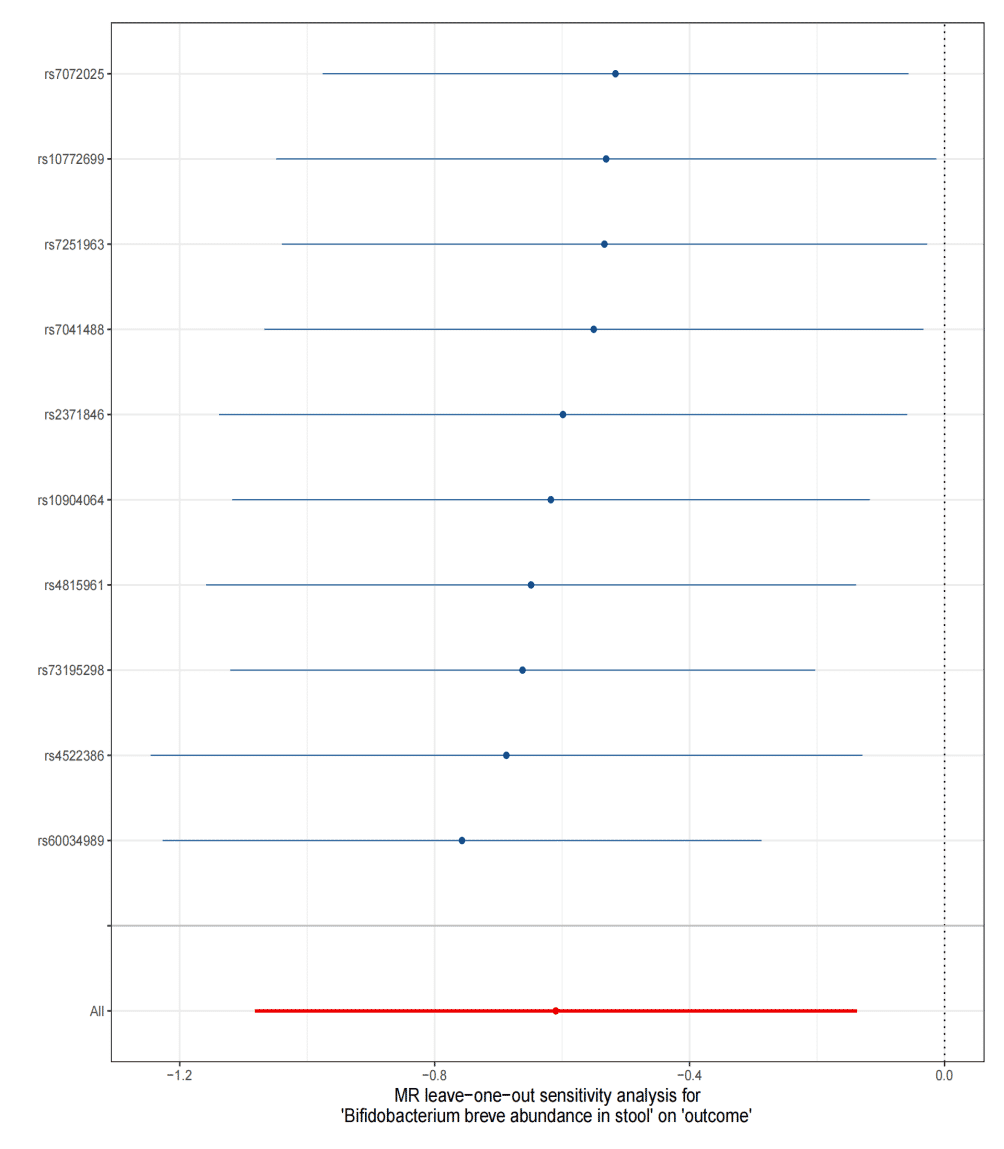

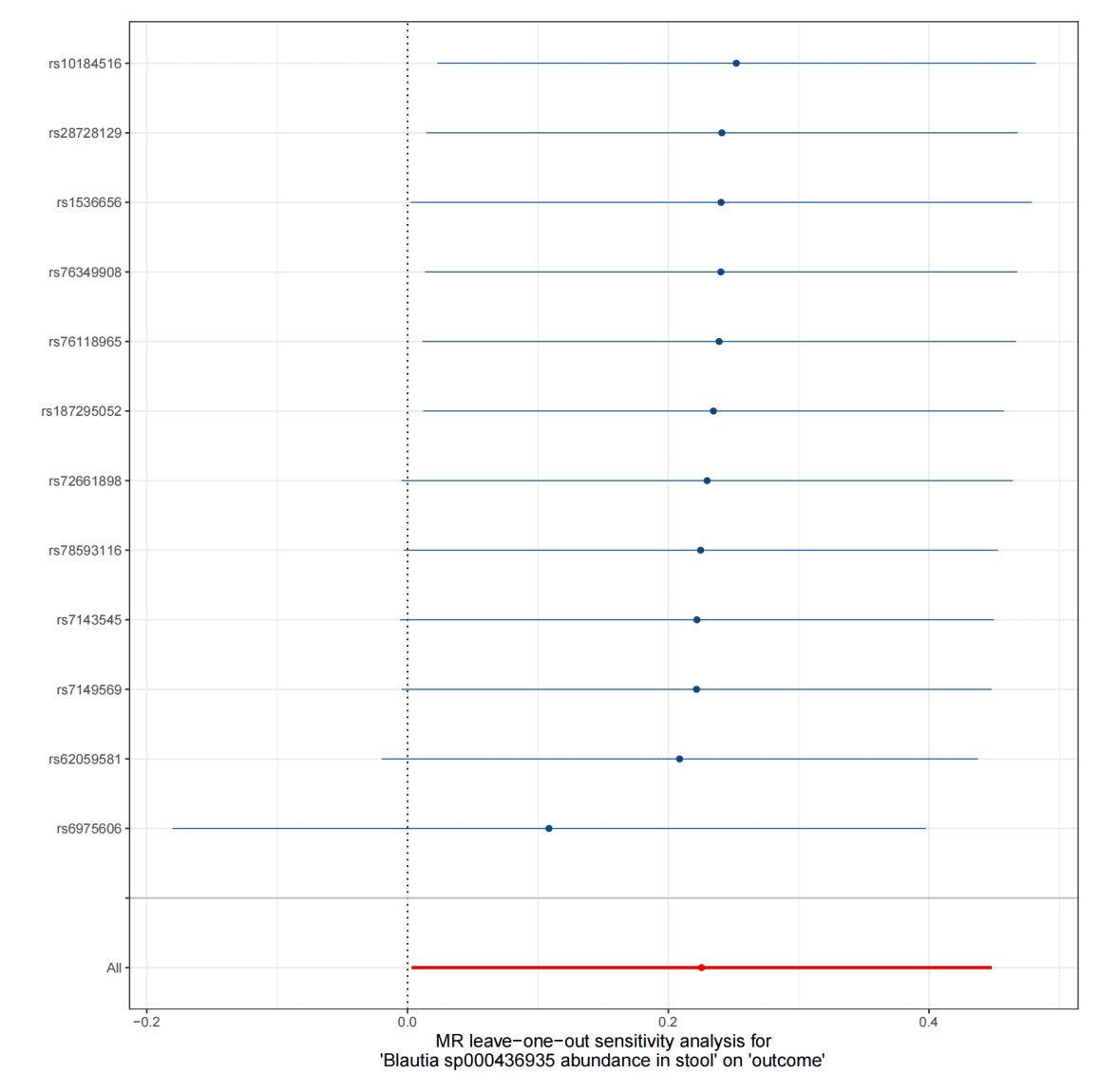

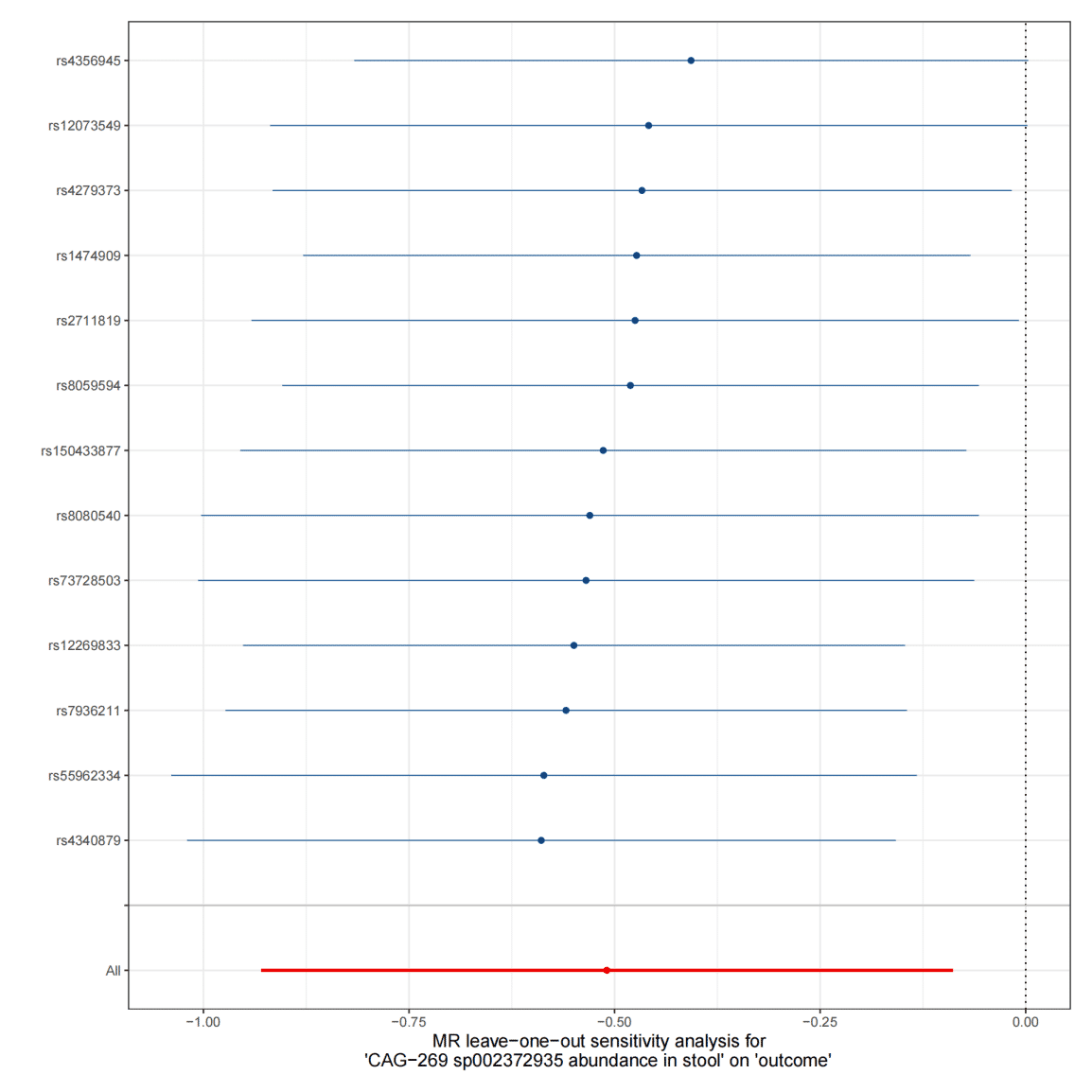


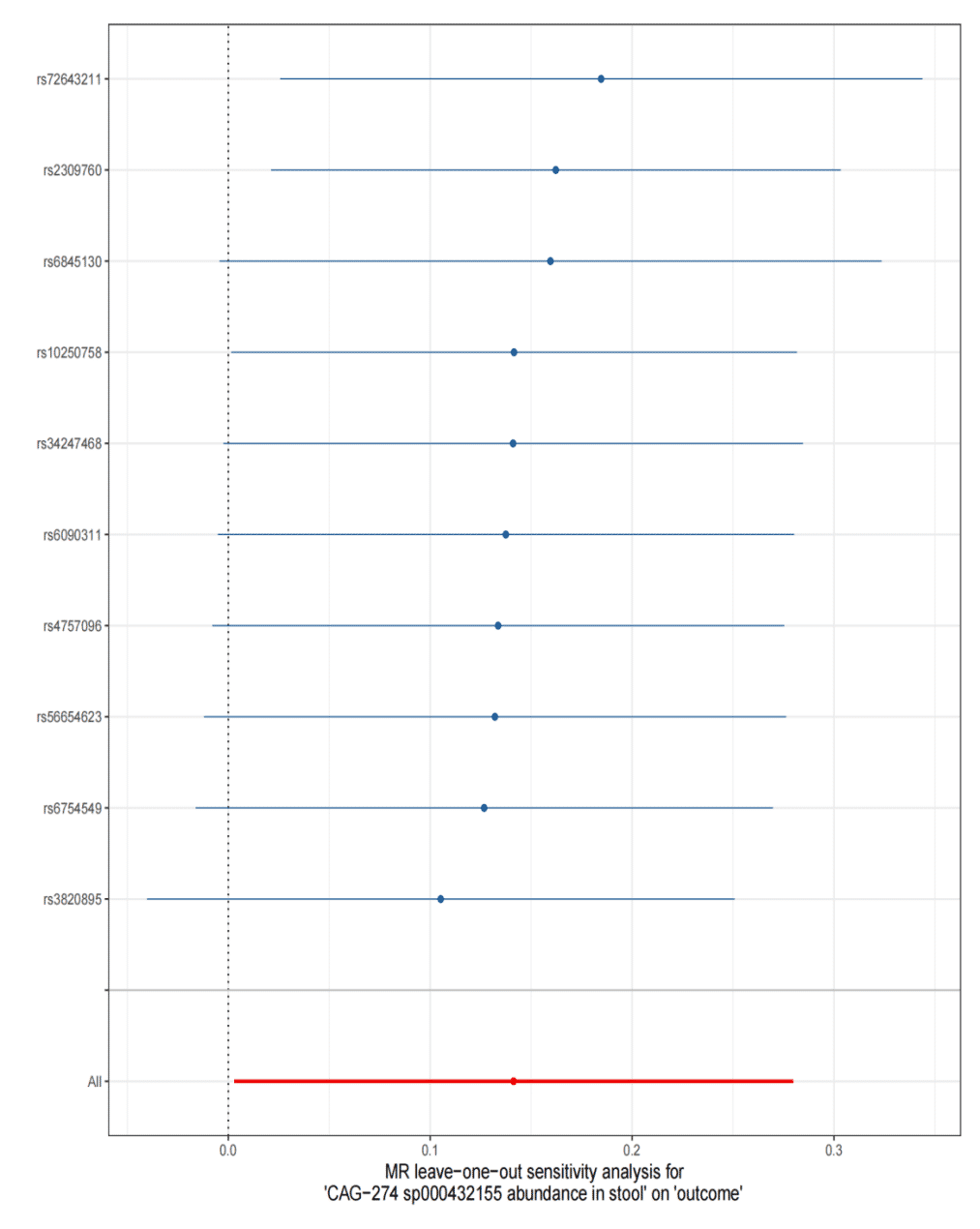

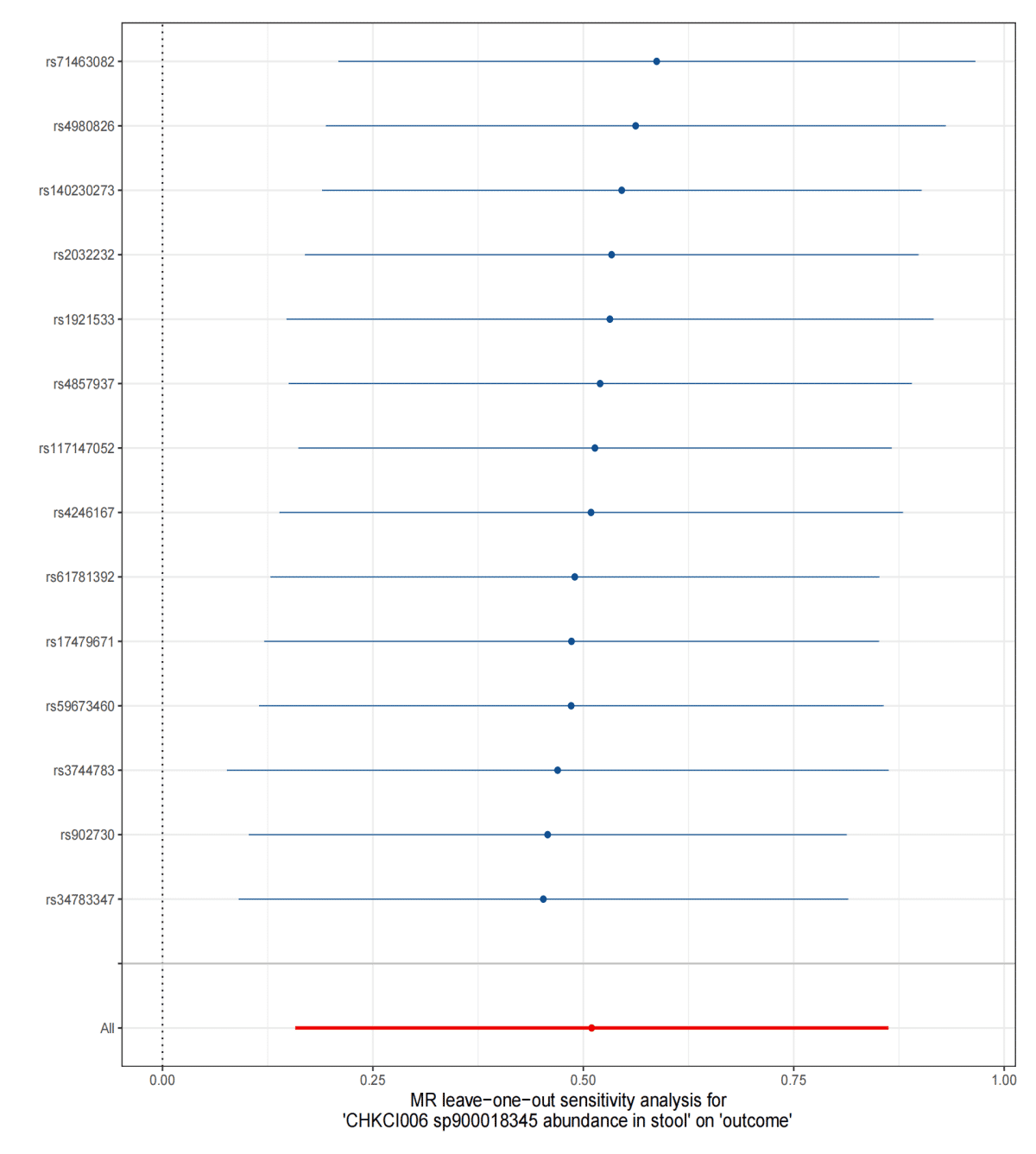

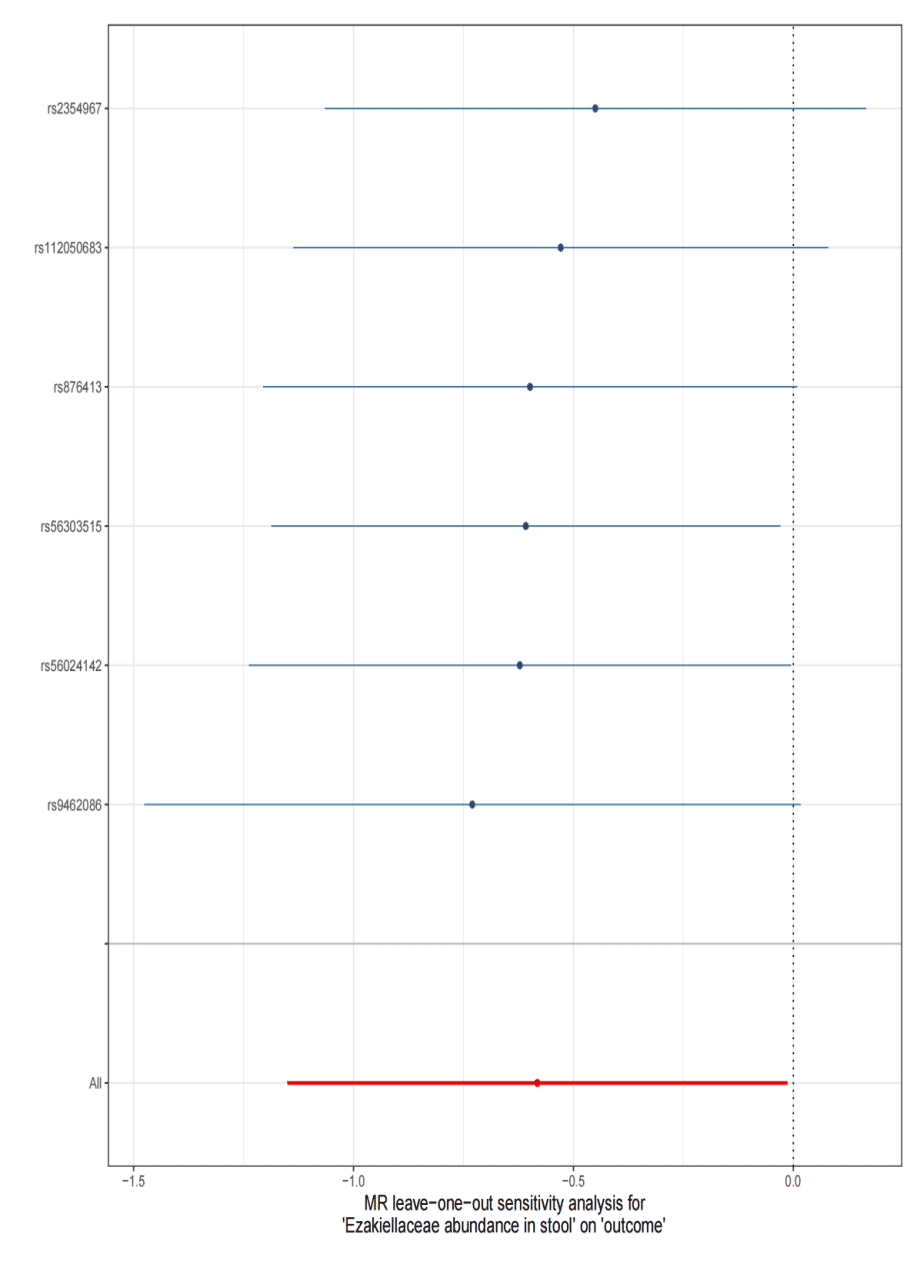


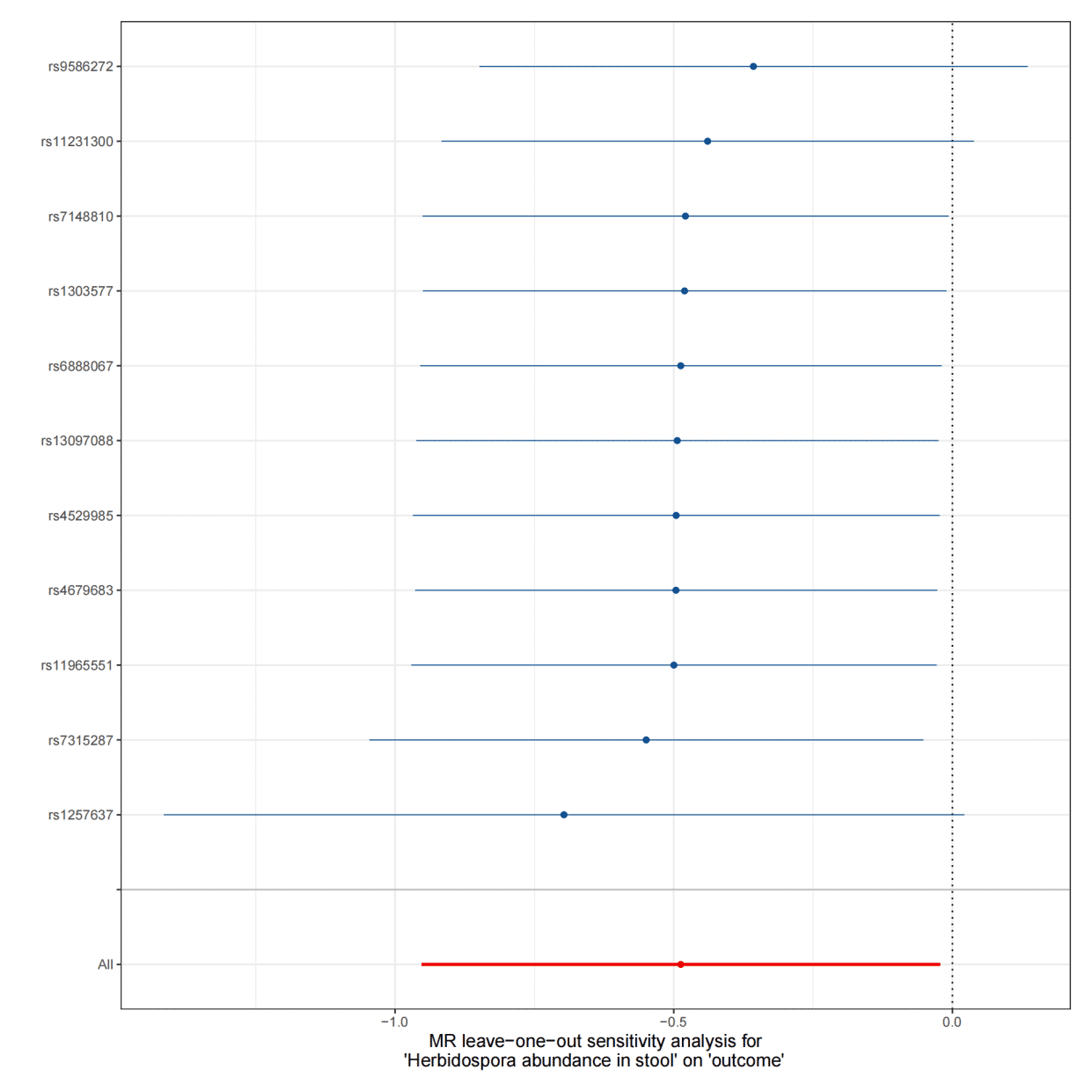

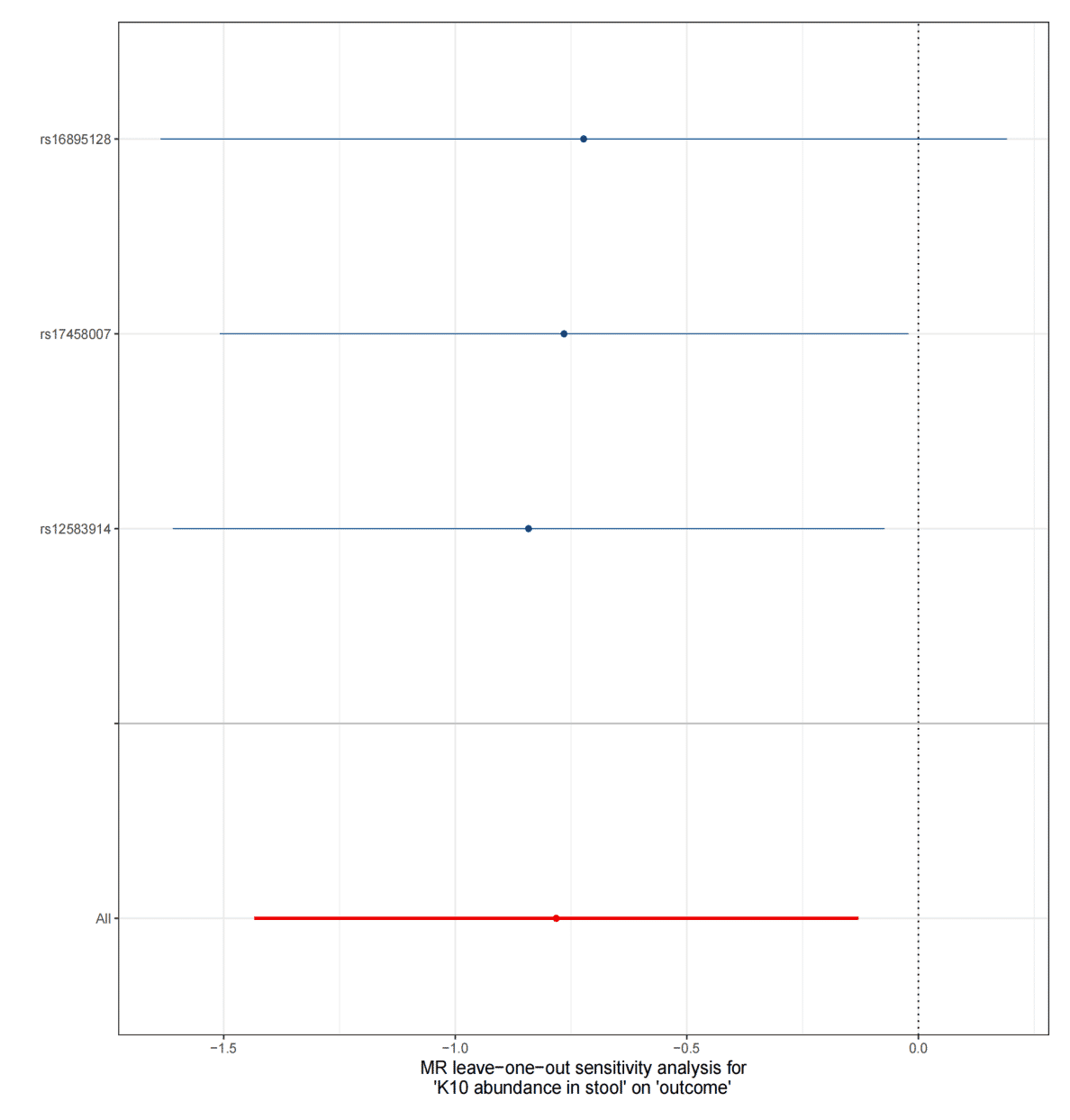

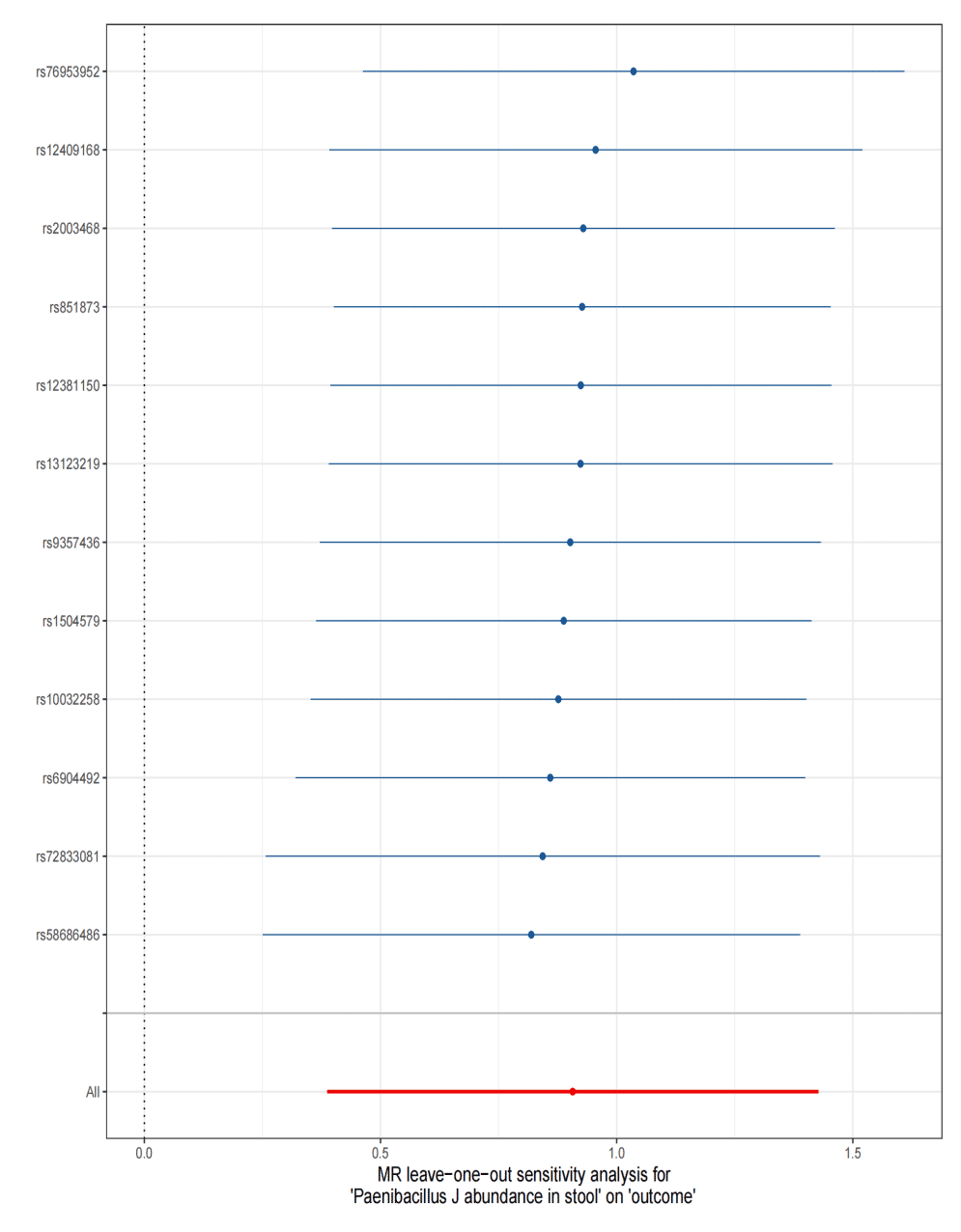


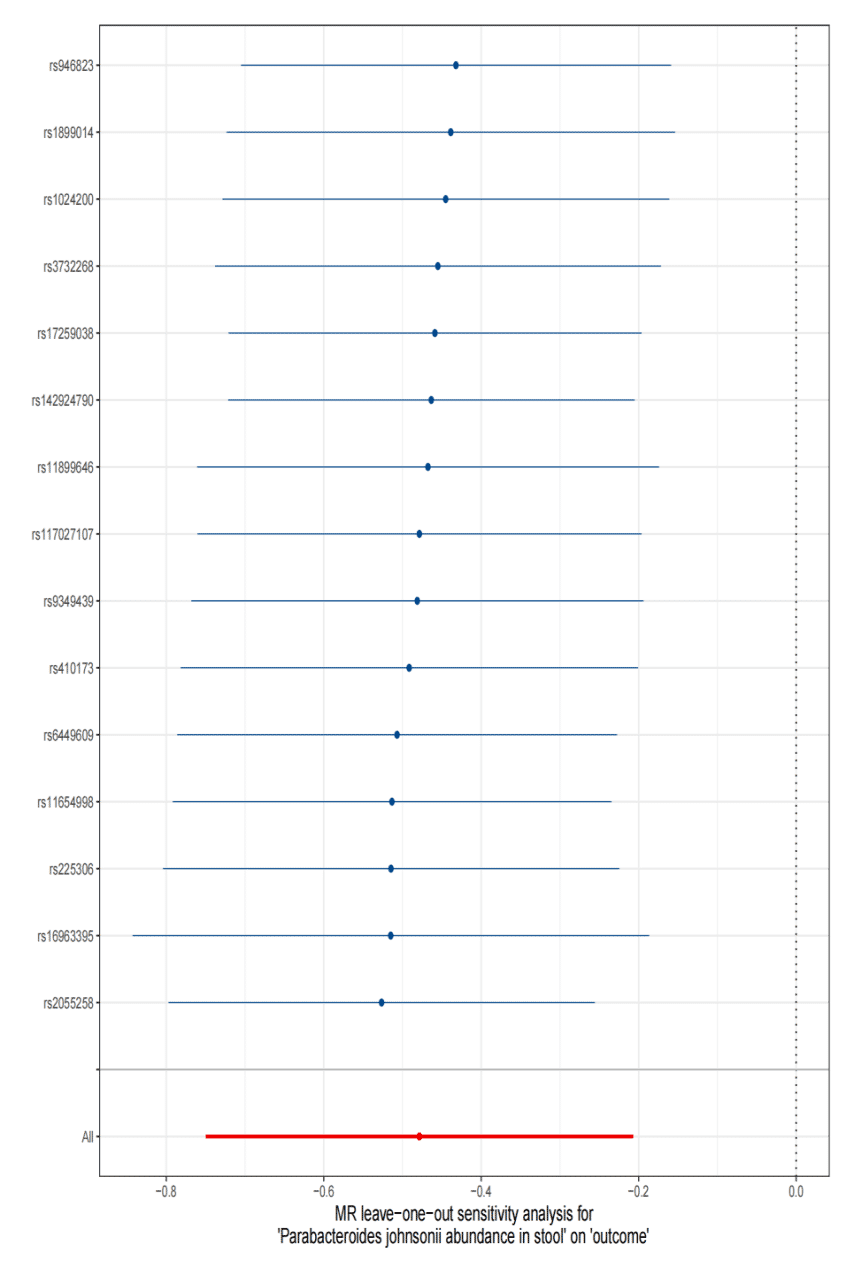

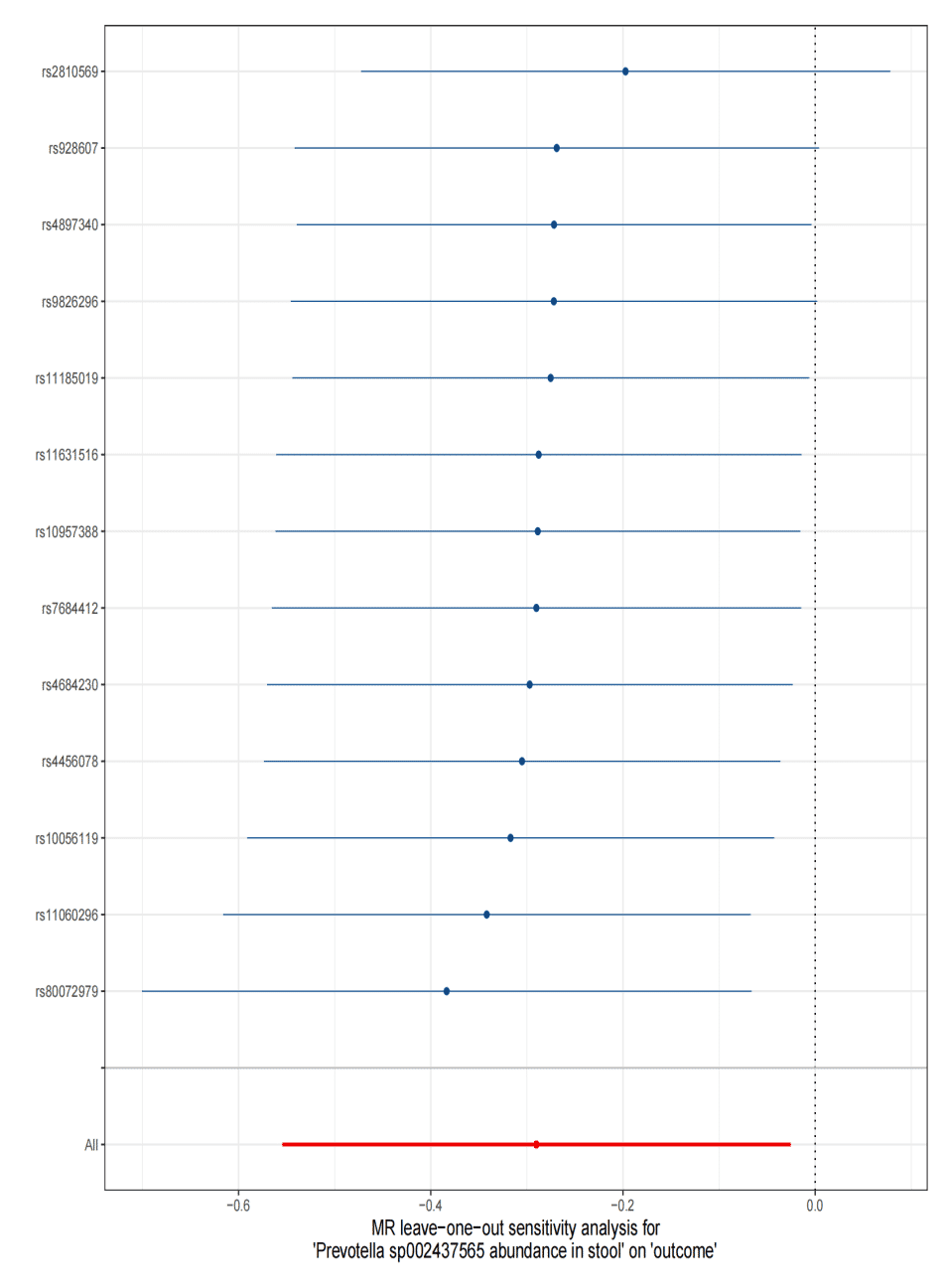

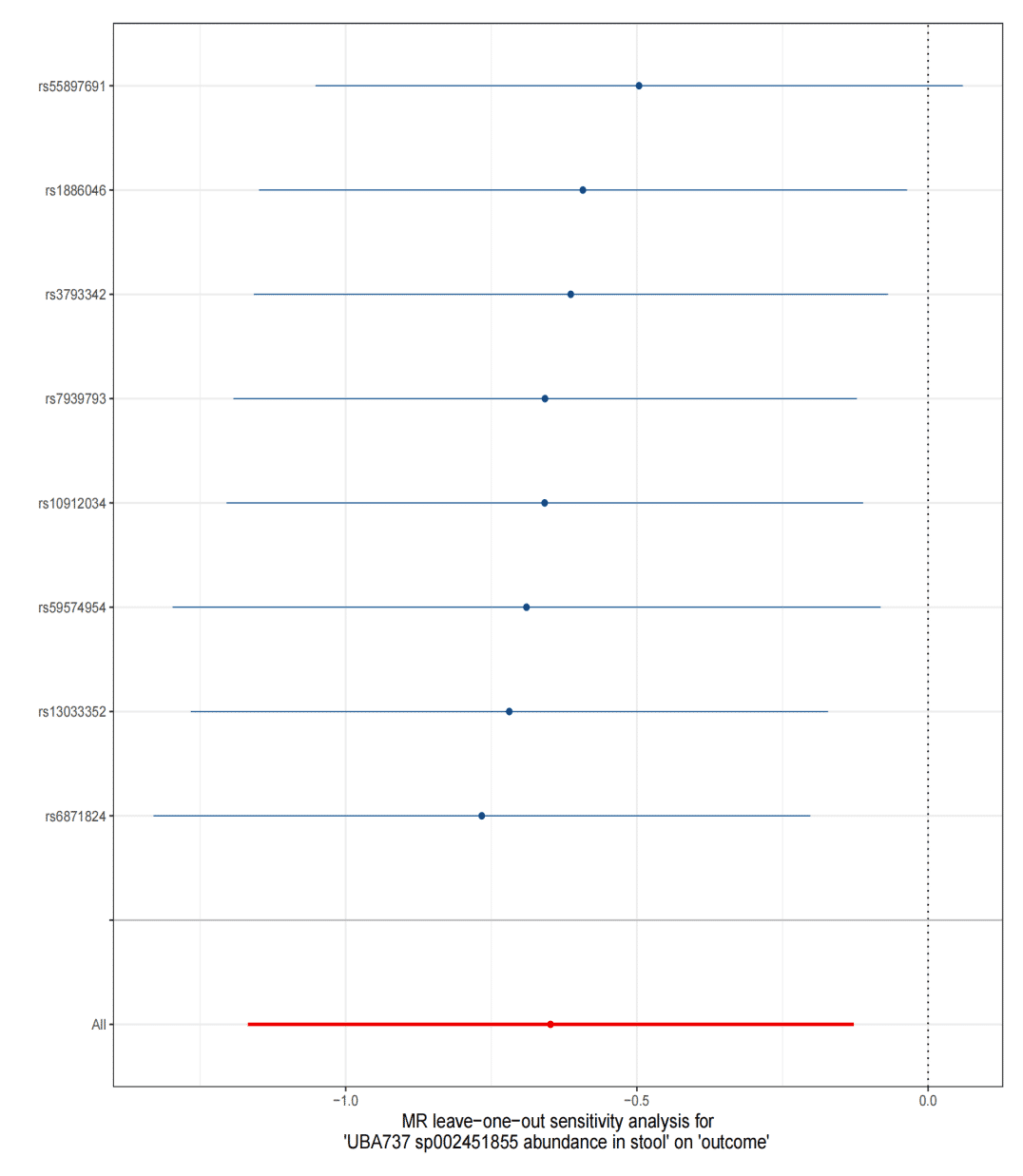


**Supplementary Figure 12: Leave-one-out analysis of the causal effect of lipids(A),Phosphatidylcholine (14:0_18:2) (B),Phosphatidylcholine (18:2_20:1) (C),Phosphatidylcholine (O-18:0_16:1) (D),Phosphatidylcholine (O-18:2_20:4) (E),Sphingomyelin (d32:1) (F),Triacylglycerol (54:6).**


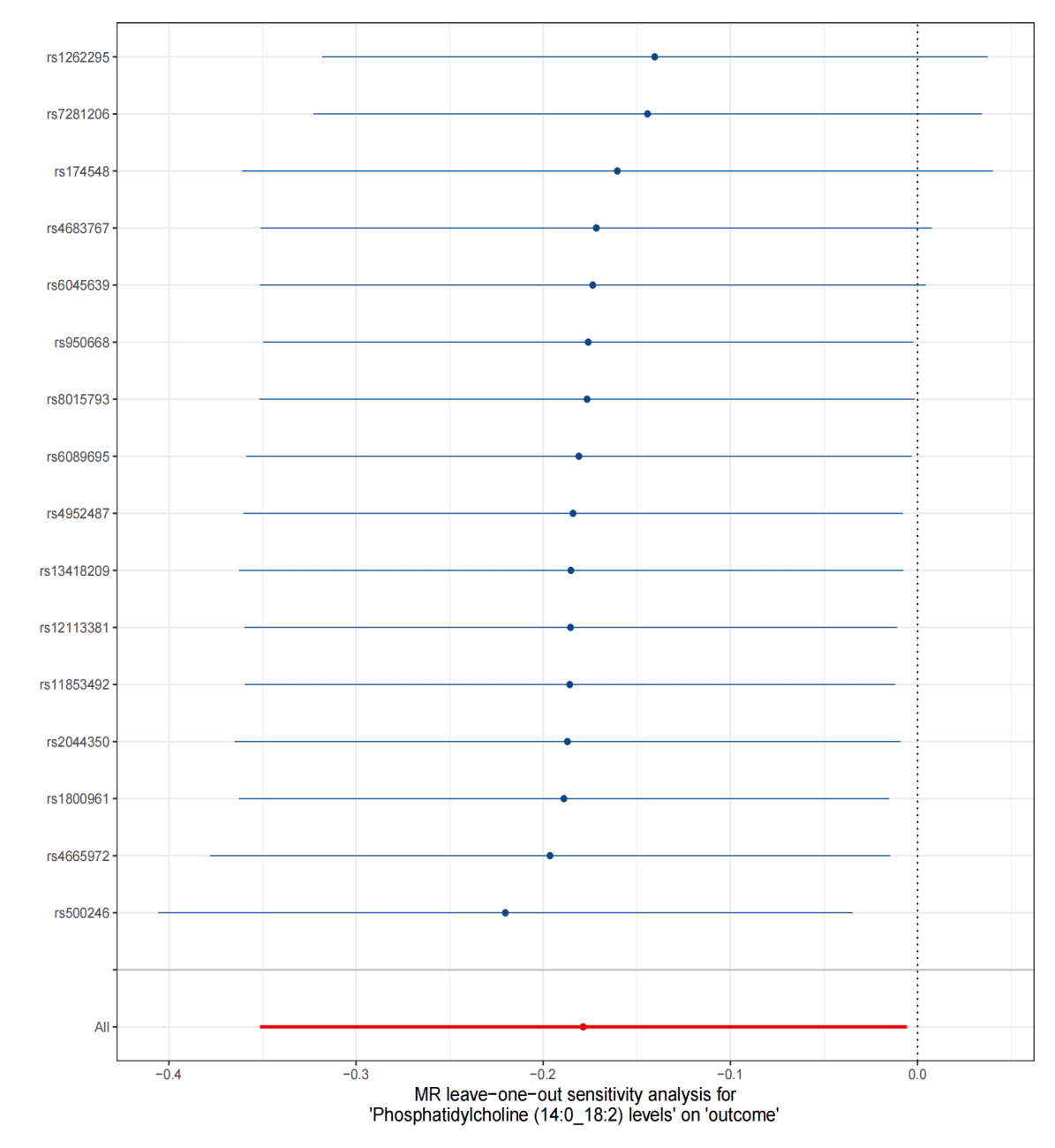

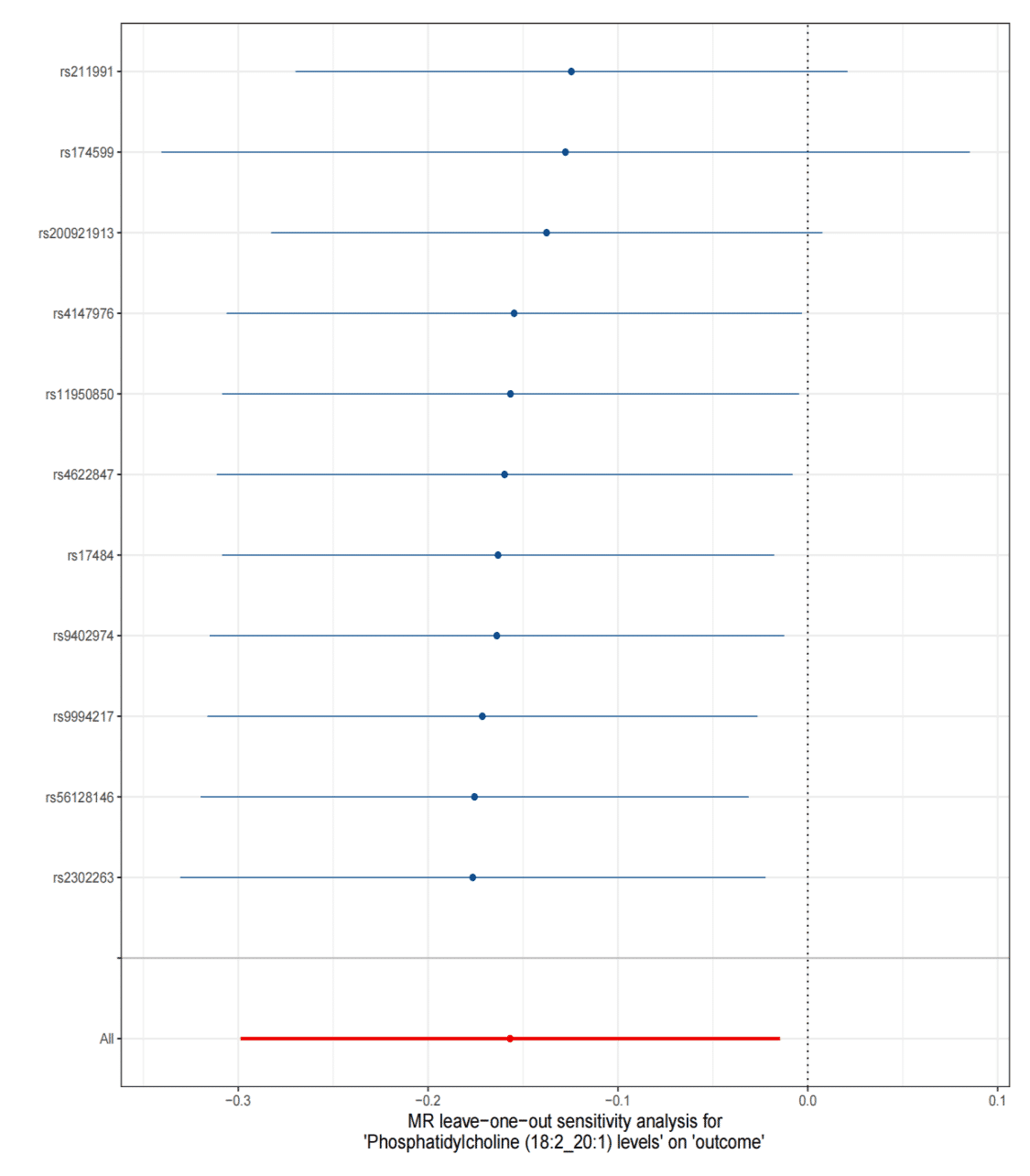

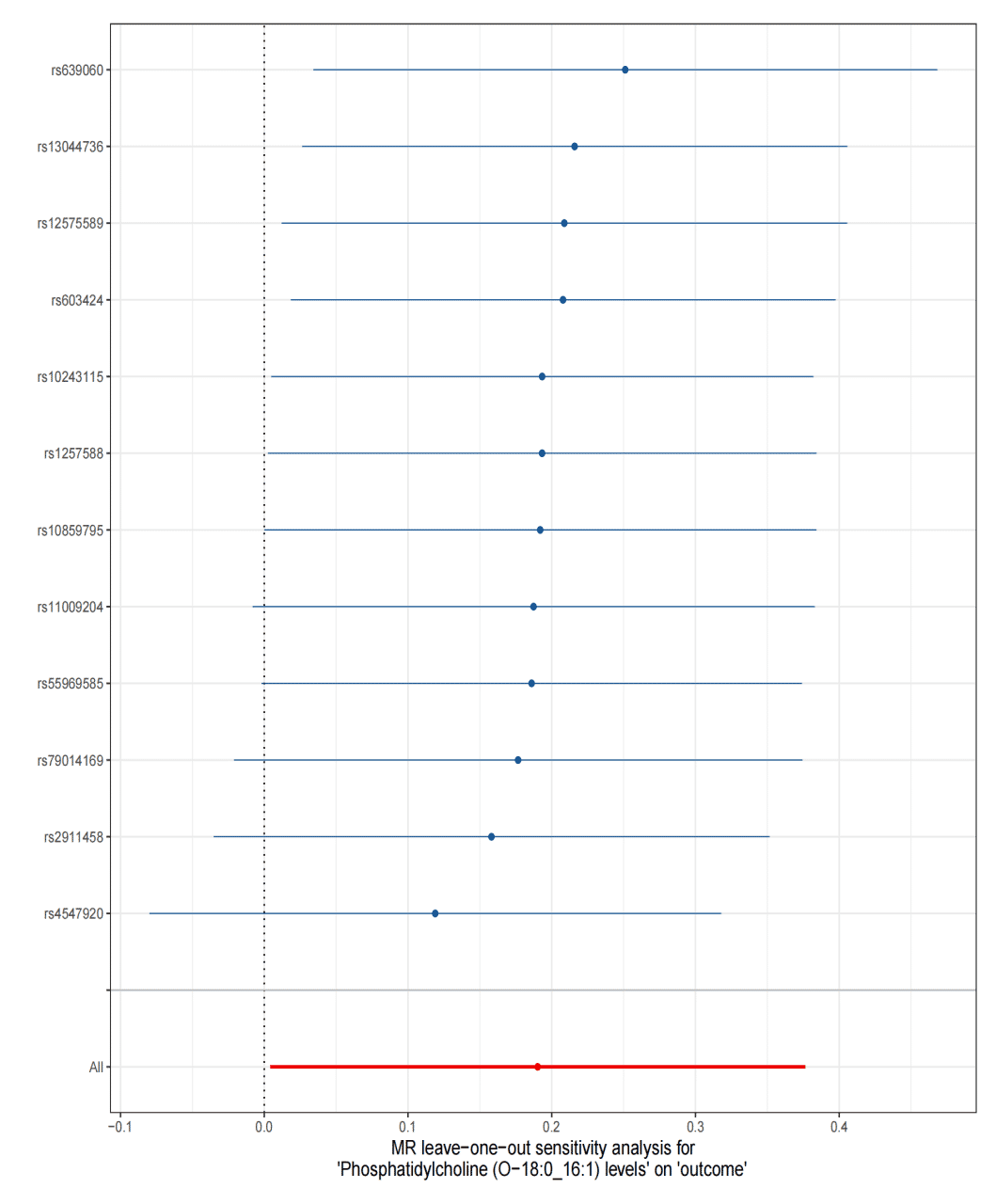


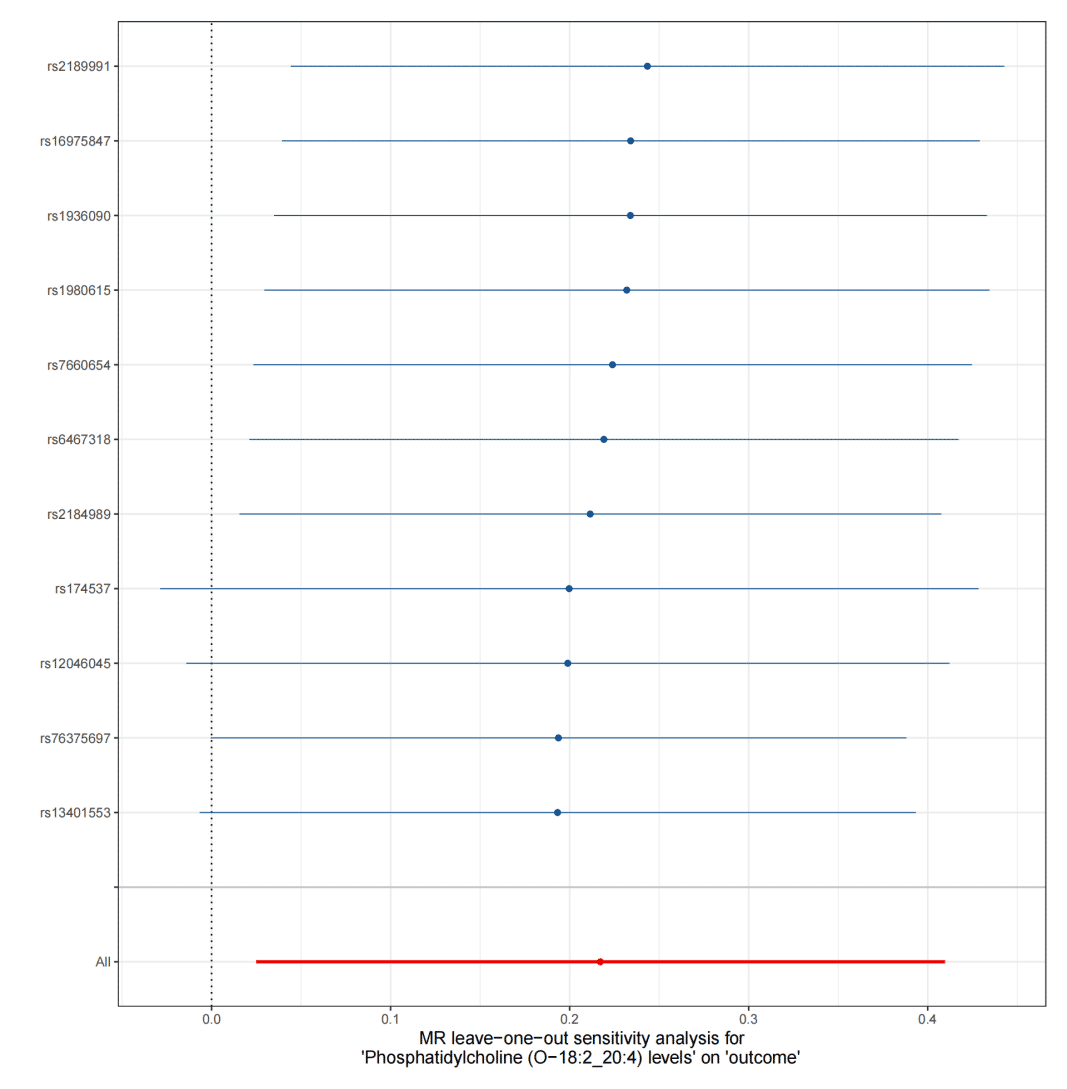

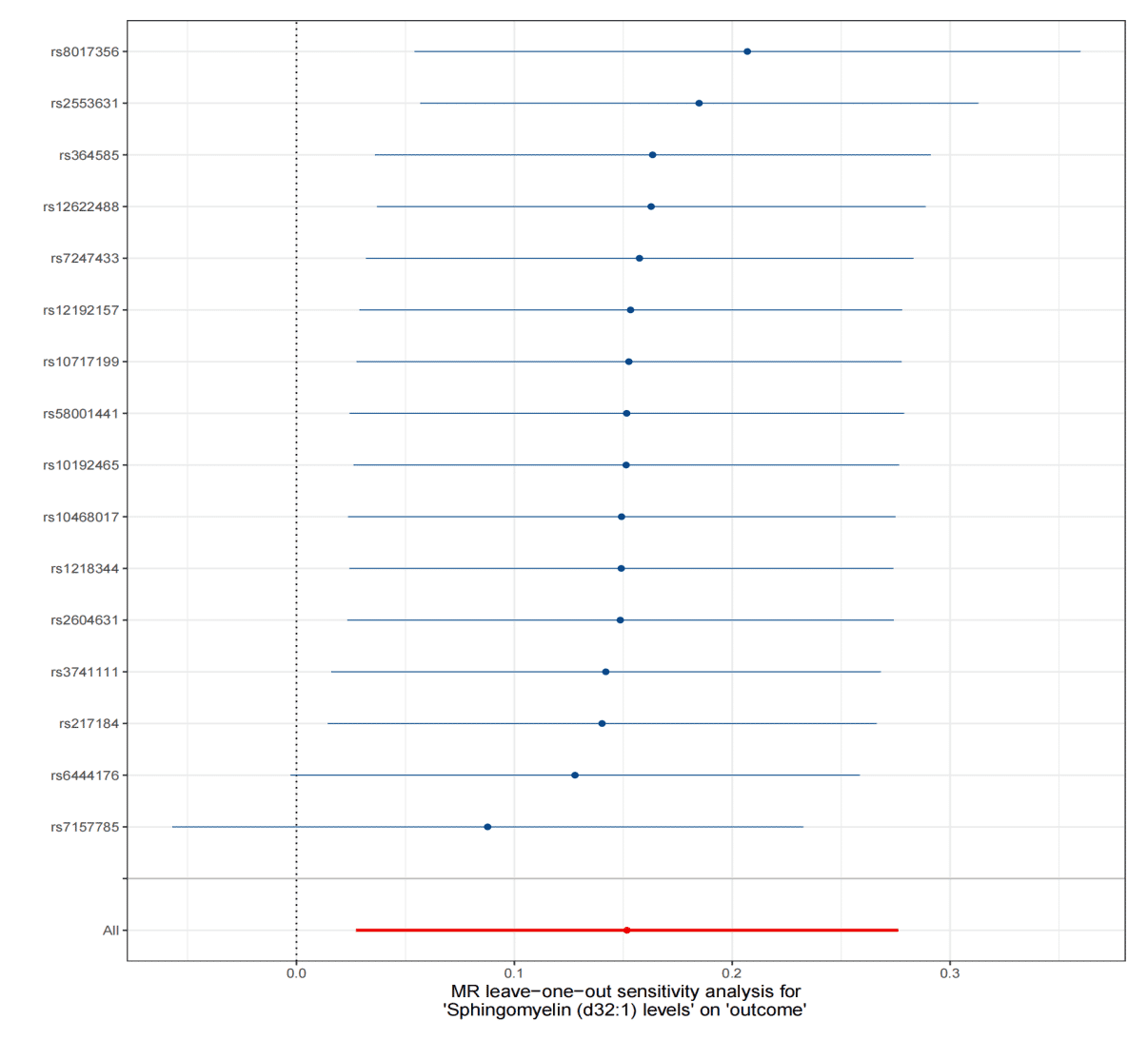

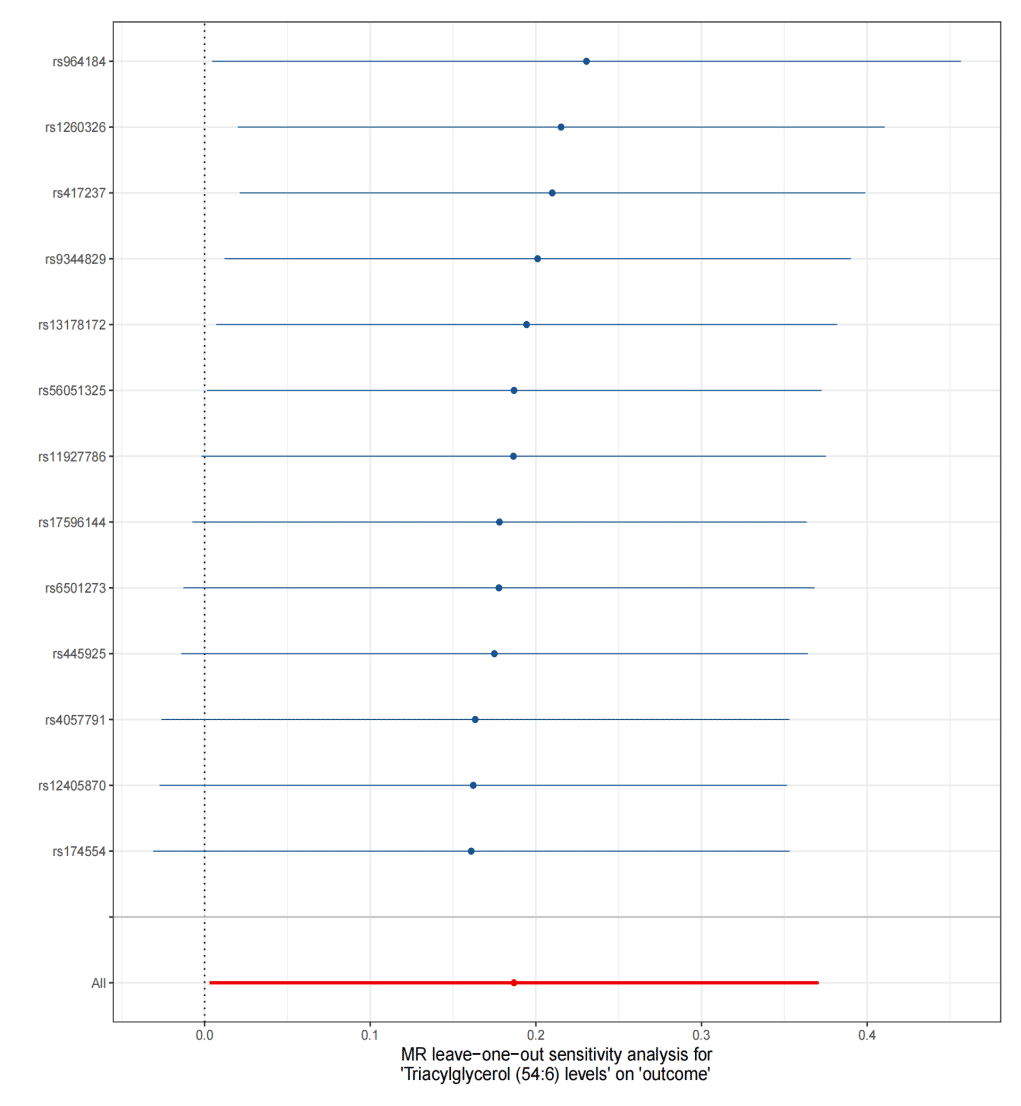


**Supplementary Table 1:MR-Egger regression analyses of gut microbiota.**

| **id.exposure** | **id.outcome** | **outcome** | **exposure** | **egger_intercept** | **se** | **pval** |
| --- | --- | --- | --- | --- | --- | --- |
| GCST90032172 | T4eVfC | outcome | Absiella dolichum abundance in stool | 0.00732292540523936 | 0.0411642692561307 | 0.865787992362142 |
| GCST90032173 | T4eVfC | outcome | Acetobacteraceae abundance in stool | 0.0102904120273057 | 0.0815369460225536 | 0.902683449900768 |
| GCST90032174 | T4eVfC | outcome | Acetobacterales abundance in stool | 0.00384943504488243 | 0.0736248768078758 | 0.959239470907159 |
| GCST90032175 | T4eVfC | outcome | Achromobacter abundance in stool | 0.026409225895713 | 0.0456582644202013 | 0.58806133839002 |
| GCST90032176 | T4eVfC | outcome | Acidaminococcus fermentans abundance in stool | 0.0346781664472938 | 0.0532530674388367 | 0.535704547804042 |
| GCST90032177 | T4eVfC | outcome | Acidaminococcus sp900315205 abundance in stool | -0.031926146 | 0.0308808400050528 | 0.328186302578062 |
| GCST90032178 | T4eVfC | outcome | Acidobacteriales abundance in stool | 0.00334991791036073 | 0.0774983305670857 | 0.966728807342562 |
| GCST90032179 | T4eVfC | outcome | Actinobacteria abundance in stool | -0.031593469 | 0.027425052660685 | 0.313490651955211 |
| GCST90032180 | T4eVfC | outcome | Actinobacteriota abundance in stool | -0.069729431 | 0.0449991348294522 | 0.155654376316038 |
| GCST90032181 | T4eVfC | outcome | Actinomycetales abundance in stool | 0.310328628487182 | 0.2633271679315 | 0.447955949183313 |
| GCST90032182 | T4eVfC | outcome | Agathobacter sp000434275 abundance in stool | 0.0233687326009874 | 0.0337945801053653 | 0.527295094915547 |
| GCST90032183 | T4eVfC | outcome | Akkermansia muciniphila B abundance in stool | 0.0186761281149423 | 0.0252263965707189 | 0.476116211751076 |
| GCST90032184 | T4eVfC | outcome | Aliivibrio abundance in stool | 0.0141011838279156 | 0.0792639795531127 | 0.863839066982697 |
| GCST90032185 | T4eVfC | outcome | Alistipes shahii abundance in stool | -0.0097476 | 0.0339318983356465 | 0.783569217397509 |
| GCST90032186 | T4eVfC | outcome | Alistipes abundance in stool | 0.010877632175041 | 0.0273511010349535 | 0.711159542073488 |
| GCST90032187 | T4eVfC | outcome | Alloprevotella abundance in stool | -0.014045463 | 0.0254042119328945 | 0.593810354087898 |
| GCST90032188 | T4eVfC | outcome | Alteromonadaceae abundance in stool | -0.019111383 | 0.0412063747547137 | 0.651093568728736 |
| GCST90032189 | T4eVfC | outcome | An181 abundance in stool | -0.004811582 | 0.0404301514507483 | 0.907413233610831 |
| GCST90032190 | T4eVfC | outcome | An7 abundance in stool | 0.0645992305453953 | 0.0516043079124869 | 0.257225206407109 |
| GCST90032191 | T4eVfC | outcome | Anaeromassilibacillus sp001305115 abundance in stool | 0.0173212628534488 | 0.0339919297851844 | 0.626020473807452 |
| GCST90032192 | T4eVfC | outcome | Aneurinibacillaceae abundance in stool | 0.111171796221014 | 0.0512030428194089 | 0.0617034886348959 |
| GCST90032193 | T4eVfC | outcome | Aneurinibacillales abundance in stool | 0.106066945837445 | 0.051183627580724 | 0.0719733202739233 |
| GCST90032194 | T4eVfC | outcome | AR31 abundance in stool | 0.0329294811051931 | 0.0771456074988763 | 0.687245114701947 |
| GCST90032195 | T4eVfC | outcome | Atopobiaceae abundance in stool | 0.0193092773266253 | 0.0548699156828872 | 0.732208568960964 |
| GCST90032196 | T4eVfC | outcome | Aureimonas abundance in stool | 0.205822710629247 | 0.103171872032239 | 0.0862593617364509 |
| GCST90032197 | T4eVfC | outcome | Azorhizobium abundance in stool | -0.003596944 | 0.0416109640061258 | 0.933535556067519 |
| GCST90032198 | T4eVfC | outcome | Bacillaceae A abundance in stool | -0.009731391 | 0.0785180418486327 | 0.907342557797389 |
| GCST90032199 | T4eVfC | outcome | Bacillales A abundance in stool | 0.0441617380250551 | 0.087582015698744 | 0.648800065888956 |
| GCST90032200 | T4eVfC | outcome | Bacilli A abundance in stool | -0.064736633 | 0.0738171475294033 | 0.409580324040479 |
| GCST90032201 | T4eVfC | outcome | Bacillus AY abundance in stool | 0.00764272784264039 | 0.0516361026162591 | 0.885996424373995 |
| GCST90032202 | T4eVfC | outcome | Bacillus C abundance in stool | -0.054732804 | 0.0359969649921228 | 0.159357779644404 |
| GCST90032203 | T4eVfC | outcome | Bacillus abundance in stool | 0.0258548914394509 | 0.0588478334515691 | 0.673650030475945 |
| GCST90032204 | T4eVfC | outcome | Bacillus U abundance in stool | 0.00165610156465483 | 0.034536392079682 | 0.962929719259705 |
| GCST90032205 | T4eVfC | outcome | Bacillus velezensis abundance in stool | -0.006669887 | 0.0353826005432221 | 0.855827428836375 |
| GCST90032206 | T4eVfC | outcome | Bacteroides A plebeius A abundance in stool | 0.057292679263832 | 0.0587422347349089 | 0.367066630848117 |
| GCST90032207 | T4eVfC | outcome | Bacteroides A plebeius abundance in stool | 0.00796745483565389 | 0.0370100753106987 | 0.833877741448074 |
| GCST90032208 | T4eVfC | outcome | Bacteroides A abundance in stool | -0.193313714 | 0.0995554025284092 | 0.147455989364463 |
| GCST90032209 | T4eVfC | outcome | Bacteroides clarus abundance in stool | -0.073737194 | 0.124699233091844 | 0.586104839432485 |
| GCST90032210 | T4eVfC | outcome | Bacteroides eggerthii abundance in stool | 0.0174420877600261 | 0.0500842026578503 | 0.736635237399609 |
| GCST90032211 | T4eVfC | outcome | Bacteroides faecis abundance in stool | -0.026095991 | 0.0514754757844163 | 0.6388534933649 |
| GCST90032212 | T4eVfC | outcome | Bacteroides intestinalis A abundance in stool | -0.056416641 | 0.0473316777652729 | 0.278285405089833 |
| GCST90032213 | T4eVfC | outcome | Bacteroides sp002160055 abundance in stool | -0.060073499 | 0.0514213947971537 | 0.269799701275669 |
| GCST90032214 | T4eVfC | outcome | Bacteroides sp003545565 abundance in stool | 0.030672763746944 | 0.0382447668033322 | 0.448911832155813 |
| GCST90032215 | T4eVfC | outcome | Bacteroides stercoris abundance in stool | -0.042876624 | 0.0662496877521832 | 0.563612800037969 |
| GCST90032216 | T4eVfC | outcome | Bacteroides thetaiotaomicron abundance in stool | 0.0403833964027414 | 0.100022166360317 | 0.707053501481771 |
| GCST90032217 | T4eVfC | outcome | Barnesiellaceae abundance in stool | -0.035534069 | 0.035790625041241 | 0.35912823389972 |
| GCST90032218 | T4eVfC | outcome | Barnesiella abundance in stool | -0.019346342 | 0.0356635037155102 | 0.607040653295313 |
| GCST90032219 | T4eVfC | outcome | Bifidobacteriaceae abundance in stool | 0.00550675436355398 | 0.0813852472969875 | 0.950311253241923 |
| GCST90032220 | T4eVfC | outcome | Bifidobacterium adolescentis abundance in stool | 0.0674464202925552 | 0.0332598742158288 | 0.0771126220021805 |
| GCST90032221 | T4eVfC | outcome | Bifidobacterium angulatum abundance in stool | 0.0117709334114852 | 0.0253363653790263 | 0.653261713763543 |
| GCST90032222 | T4eVfC | outcome | Bifidobacterium bifidum abundance in stool | 0.0471277230474387 | 0.0582052886116929 | 0.449034121326991 |
| GCST90032223 | T4eVfC | outcome | Bifidobacterium breve abundance in stool | -0.004172574 | 0.0531621735848438 | 0.939367830653294 |
| GCST90032224 | T4eVfC | outcome | Bifidobacterium catenulatum abundance in stool | 0.069727887368888 | 0.164720663561831 | 0.693837715117854 |
| GCST90032225 | T4eVfC | outcome | Bifidobacterium infantis abundance in stool | 0.110016828638795 | 0.0915198636706356 | 0.283142665422893 |
| GCST90032226 | T4eVfC | outcome | Bifidobacterium kashiwanohense abundance in stool | -0.018268684 | 0.0631224966159137 | 0.783879647075998 |
| GCST90032227 | T4eVfC | outcome | Bifidobacterium longum abundance in stool | -0.119654238 | 0.144132403312756 | 0.493759963824314 |
| GCST90032228 | T4eVfC | outcome | Bifidobacterium pseudocatenulatum abundance in stool | 0.00346993100734505 | 0.0237334880619753 | 0.889472079842579 |
| GCST90032229 | T4eVfC | outcome | Bifidobacterium ruminantium abundance in stool | -0.046209015 | 0.0409464319942233 | 0.291802208615228 |
| GCST90032230 | T4eVfC | outcome | Bifidobacterium abundance in stool | 0.0061782301640148 | 0.0738349826005518 | 0.93733423135747 |
| GCST90032231 | T4eVfC | outcome | Bin127 abundance in stool | -0.028913792 | 0.100347523997892 | 0.780566239596737 |
| GCST90032232 | T4eVfC | outcome | Blautia A sp000285855 abundance in stool | 0.0109943104789687 | 0.0361241024274898 | 0.773122183730411 |
| GCST90032233 | T4eVfC | outcome | Blautia A sp002159835 abundance in stool | 0.0385446540994843 | 0.0756549379424989 | 0.626080492794139 |
| GCST90032234 | T4eVfC | outcome | Blautia A sp900066145 abundance in stool | -0.069421214 | 0.0389120174863207 | 0.124674678132166 |
| GCST90032235 | T4eVfC | outcome | Blautia A sp900066355 abundance in stool | -0.02104393 | 0.0626902532760003 | 0.753979697823345 |
| GCST90032236 | T4eVfC | outcome | Blautia hansenii abundance in stool | -0.044083838 | 0.0334690399532627 | 0.217169627914556 |
| GCST90032237 | T4eVfC | outcome | Blautia sp000436935 abundance in stool | -0.019245802 | 0.0238477492237612 | 0.438423474934768 |
| GCST90032238 | T4eVfC | outcome | Blautia sp001304935 abundance in stool | -0.007549877 | 0.0597070740762153 | 0.904304347246383 |
| GCST90032239 | T4eVfC | outcome | Borreliaceae abundance in stool | 0.0840463818663899 | 0.0347653956122892 | 0.052037745220409 |
| GCST90032240 | T4eVfC | outcome | Borreliales abundance in stool | 0.0552065297555768 | 0.0315882550296755 | 0.12400994358013 |
| GCST90032241 | T4eVfC | outcome | Brachyspiraceae abundance in stool | -0.013554772 | 0.0258706376053982 | 0.610711348687896 |
| GCST90032242 | T4eVfC | outcome | Brachyspirae abundance in stool | -0.00498694 | 0.0261145994418279 | 0.852375391165187 |
| GCST90032243 | T4eVfC | outcome | Brachyspirales abundance in stool | -0.006575777 | 0.0248900545184984 | 0.796515556564915 |
| GCST90032244 | T4eVfC | outcome | Brachyspira abundance in stool | 0.00354868137675269 | 0.0266490844626201 | 0.896705782694645 |
| GCST90032245 | T4eVfC | outcome | Brevibacillaceae abundance in stool | 0.0398868448610374 | 0.0437299899448968 | 0.396874482052629 |
| GCST90032246 | T4eVfC | outcome | Brevibacillales abundance in stool | -0.015334615 | 0.0347618784548264 | 0.672420289774956 |
| GCST90032247 | T4eVfC | outcome | Brevibacillus B abundance in stool | -0.014095161 | 0.0406830038956646 | 0.736174148582682 |
| GCST90032248 | T4eVfC | outcome | Butyricimonas sp900258545 abundance in stool | 0.0581709369875653 | 0.0544702192858616 | 0.316710670771315 |
| GCST90032249 | T4eVfC | outcome | CAG-1000 sp000434555 abundance in stool | 0.0387201180108291 | 0.0795617605938433 | 0.647075984771268 |
| GCST90032250 | T4eVfC | outcome | CAG-1000 abundance in stool | 0.0657182682937696 | 0.058271733854723 | 0.302478139222318 |
| GCST90032251 | T4eVfC | outcome | CAG-1031 abundance in stool | 0.026743809675243 | 0.0294194662219369 | 0.379867844168004 |
| GCST90032252 | T4eVfC | outcome | CAG-110 abundance in stool | -0.02482187 | 0.0323672526598688 | 0.465178429768566 |
| GCST90032253 | T4eVfC | outcome | CAG-145 sp000435615 abundance in stool | 0.0205192081439578 | 0.060161261341763 | 0.743059216862197 |
| GCST90032254 | T4eVfC | outcome | CAG-145 sp002320005 abundance in stool | 0.00465445791723368 | 0.0698346877991109 | 0.950058965002557 |
| GCST90032255 | T4eVfC | outcome | CAG-145 abundance in stool | -0.038169535 | 0.0532176364969733 | 0.525030321031894 |
| GCST90032256 | T4eVfC | outcome | CAG-170 sp003516765 abundance in stool | 0.0009430765658447 | 0.0678831693449061 | 0.989143936999493 |
| GCST90032257 | T4eVfC | outcome | CAG-177 sp002438685 abundance in stool | -0.017196525 | 0.0289970619810214 | 0.57180609414288 |
| GCST90032258 | T4eVfC | outcome | CAG-177 sp002451755 abundance in stool | -0.087417316 | 0.0755205371925392 | 0.285021977806836 |
| g |  |  |  |  |  |  |
| GCST90032260 | T4eVfC | outcome | CAG-177 sp003538135 abundance in stool | -0.008918703 | 0.0227315583241461 | 0.701157459450358 |
| GCST90032261 | T4eVfC | outcome | CAG-177 abundance in stool | -0.009363066 | 0.0291717647126446 | 0.759123477885744 |
| GCST90032262 | T4eVfC | outcome | CAG-180 sp000432435 abundance in stool | -0.104287371 | 0.0967003544256156 | 0.306155131557439 |
| GCST90032263 | T4eVfC | outcome | CAG-194 sp002441865 abundance in stool | 0.0180431234158389 | 0.0346710836847411 | 0.615337528552016 |
| GCST90032264 | T4eVfC | outcome | CAG-245 sp000435175 abundance in stool | -0.037421532 | 0.0477209110527832 | 0.453081355921818 |
| GCST90032265 | T4eVfC | outcome | CAG-245 abundance in stool | -0.013080084 | 0.0582578732127461 | 0.831239068266259 |
| GCST90032266 | T4eVfC | outcome | CAG-269 sp001915995 abundance in stool | -0.028869991 | 0.0330746366305664 | 0.408166272007674 |
| GCST90032267 | T4eVfC | outcome | CAG-269 sp001916065 abundance in stool | -0.018260246 | 0.0735833142866687 | 0.813882233122801 |
| GCST90032268 | T4eVfC | outcome | CAG-269 sp002372935 abundance in stool | 0.0120383578312965 | 0.0649419946759994 | 0.856311910468524 |
| GCST90032269 | T4eVfC | outcome | CAG-273 sp003507395 abundance in stool | 0.0148218265165752 | 0.0413952366133696 | 0.72652045885872 |
| GCST90032270 | T4eVfC | outcome | CAG-273 sp003534295 abundance in stool | 0.0127454898345627 | 0.0529689908567959 | 0.815240023131287 |
| GCST90032271 | T4eVfC | outcome | CAG-274 sp000432155 abundance in stool | 0.00408841875885943 | 0.031934051786414 | 0.901287498502647 |
| GCST90032272 | T4eVfC | outcome | CAG-302 abundance in stool | 0.121756325886978 | 0.0796889919996414 | 0.160887926192962 |
| GCST90032273 | T4eVfC | outcome | CAG-345 sp000433315 abundance in stool | -0.026209696 | 0.0314327019519928 | 0.422111635011236 |
| GCST90032274 | T4eVfC | outcome | CAG-345 abundance in stool | -0.031215471 | 0.0323834013240582 | 0.363307953778186 |
| GCST90032275 | T4eVfC | outcome | CAG-349 abundance in stool | -0.048535586 | 0.0572127861939275 | 0.418242120352475 |
| GCST90032276 | T4eVfC | outcome | CAG-390 sp003523225 abundance in stool | -0.009966911 | 0.023907719481737 | 0.685566085308161 |
| GCST90032277 | T4eVfC | outcome | CAG-433 abundance in stool | -0.017237112 | 0.0438953160033079 | 0.702791067439635 |
| GCST90032278 | T4eVfC | outcome | CAG-448 sp000433415 abundance in stool | -0.095946956 | 0.135794944693144 | 0.499899259896158 |
| GCST90032279 | T4eVfC | outcome | CAG-448 sp003150135 abundance in stool | -0.013526695 | 0.0382652658198506 | 0.731854248483552 |
| GCST90032280 | T4eVfC | outcome | CAG-448 abundance in stool | 0.00643772175793675 | 0.0362634138338921 | 0.864936358965912 |
| GCST90032281 | T4eVfC | outcome | CAG-449 abundance in stool | 0.0484568104183151 | 0.0326856645694162 | 0.188721942071469 |
| GCST90032282 | T4eVfC | outcome | CAG-452 sp000434035 abundance in stool | -0.054183895 | 0.0596639208698272 | 0.387469002755502 |
| GCST90032283 | T4eVfC | outcome | CAG-452 abundance in stool | -0.065457897 | 0.0591325298345867 | 0.31869925329745 |
| GCST90032284 | T4eVfC | outcome | CAG-465 sp000433135 abundance in stool | -0.040681085 | 0.032926297380025 | 0.244875439304886 |
| GCST90032285 | T4eVfC | outcome | CAG-475 abundance in stool | -0.014981295 | 0.0271341653445455 | 0.592990800117713 |
| GCST90032286 | T4eVfC | outcome | CAG-485 sp002362485 abundance in stool | 0.103404470661112 | 0.0534044682855158 | 0.124914334108969 |
| GCST90032287 | T4eVfC | outcome | CAG-485 sp002404675 abundance in stool | -0.043977331 | 0.0473694731330642 | 0.38036399893241 |
| GCST90032288 | T4eVfC | outcome | CAG-488 sp000434055 abundance in stool | -0.012982375 | 0.02514550708743 | 0.621556048905897 |
| GCST90032289 | T4eVfC | outcome | CAG-488 abundance in stool | -0.008377668 | 0.0181134694218705 | 0.650353453699224 |
| GCST90032290 | T4eVfC | outcome | CAG-495 abundance in stool | -0.092419583 | 0.0887743836530246 | 0.345545361977694 |
| GCST90032291 | T4eVfC | outcome | CAG-510 sp002432425 abundance in stool | -0.050839867 | 0.0469069593232226 | 0.31001446689723 |
| GCST90032292 | T4eVfC | outcome | CAG-552 abundance in stool | -0.038247828 | 0.0529711085189427 | 0.488598011280823 |
| GCST90032293 | T4eVfC | outcome | CAG-590 sp000431135 abundance in stool | -0.026328347 | 0.0203757056244165 | 0.213596933748337 |
| GCST90032294 | T4eVfC | outcome | CAG-632 abundance in stool | -0.048252118 | 0.0378052562167066 | 0.233787634023433 |
| GCST90032295 | T4eVfC | outcome | CAG-698 abundance in stool | -0.020367562 | 0.0320979301588231 | 0.545896933369959 |
| GCST90032296 | T4eVfC | outcome | CAG-776 sp000438195 abundance in stool | 0.00590064980754494 | 0.0384381948075217 | 0.882327765262571 |
| GCST90032297 | T4eVfC | outcome | CAG-776 abundance in stool | 0.0368160078381522 | 0.0523348331359545 | 0.504481608431213 |
| GCST90032298 | T4eVfC | outcome | CAG-81 sp000435795 abundance in stool | 0.0135877539722271 | 0.0498021528097592 | 0.792853823580219 |
| GCST90032299 | T4eVfC | outcome | CAG-822 sp000432855 abundance in stool | 0.0245218064024151 | 0.0268217437561401 | 0.376062496871732 |
| GCST90032300 | T4eVfC | outcome | CAG-822 abundance in stool | 0.0339796184409229 | 0.0305763387835995 | 0.292441716183029 |
| GCST90032301 | T4eVfC | outcome | CAG-826 abundance in stool | -0.020727644 | 0.0619542950854158 | 0.749340031078338 |
| GCST90032302 | T4eVfC | outcome | CAG-83 sp000435555 abundance in stool | -0.094776578 | 0.108748357565266 | 0.447605485309811 |
| GCST90032303 | T4eVfC | outcome | CAG-83 sp002392625 abundance in stool | -0.136377712 | 0.123700554049319 | 0.296077385843334 |
| GCST90032304 | T4eVfC | outcome | CAG-841 sp002479075 abundance in stool | -0.015731986 | 0.0316423008692542 | 0.636751296394953 |
| GCST90032305 | T4eVfC | outcome | CAG-873 sp001701165 abundance in stool | 0.00601275156417524 | 0.0381035535013742 | 0.87671883246576 |
| GCST90032306 | T4eVfC | outcome | CAG-877 sp000433455 abundance in stool | -0.041112877 | 0.037436905420949 | 0.29366752251069 |
| GCST90032307 | T4eVfC | outcome | CAG-877 abundance in stool | -0.053800003 | 0.0332505693252074 | 0.129653857504533 |
| GCST90032308 | T4eVfC | outcome | CAG-882 sp003486385 abundance in stool | -0.018341844 | 0.0450303427865727 | 0.697905315455207 |
| GCST90032309 | T4eVfC | outcome | CAG-884 sp000433875 abundance in stool | -0.085857299 | 0.0364183557572928 | 0.0427742582948725 |
| GCST90032310 | T4eVfC | outcome | CAG-884 abundance in stool | -0.05499482 | 0.0403905345774149 | 0.222238751256493 |
| GCST90032311 | T4eVfC | outcome | CAG-977 abundance in stool | -0.019042765 | 0.0213139511564404 | 0.392614893195736 |
| GCST90032312 | T4eVfC | outcome | Caloranaerobacteraceae abundance in stool | 0.0115677619555284 | 0.0434529681274372 | 0.793699398308985 |
| GCST90032313 | T4eVfC | outcome | Caloranaerobacter abundance in stool | 0.0167789579844135 | 0.0391818352062964 | 0.673857715068471 |
| GCST90032314 | T4eVfC | outcome | Campylobacter D abundance in stool | -0.031355695 | 0.0586540986732809 | 0.609487708249696 |
| GCST90032315 | T4eVfC | outcome | Cetobacterium A abundance in stool | -0.040485501 | 0.0503753955605427 | 0.432678741295415 |
| GCST90032316 | T4eVfC | outcome | CHKCI006 sp900018345 abundance in stool | 0.0309871764785848 | 0.0378753769783396 | 0.429226922631901 |
| GCST90032317 | T4eVfC | outcome | Chloroflexales abundance in stool | 0.00205359997759036 | 0.0315512905150739 | 0.949386991878882 |
| GCST90032318 | T4eVfC | outcome | Chromatiales abundance in stool | 0.0424082976273978 | 0.0483414026835208 | 0.403151422830276 |
| GCST90032319 | T4eVfC | outcome | Chromobacteriaceae abundance in stool | -0.112409128 | 0.0486526821560079 | 0.0462031766592132 |
| GCST90032320 | T4eVfC | outcome | Citrobacter A abundance in stool | 0.0249086227776367 | 0.0364710786787191 | 0.520111423964202 |
| GCST90032321 | T4eVfC | outcome | Clostridia abundance in stool | -0.103559841 | 0.108755410974444 | 0.384710838956612 |
| GCST90032322 | T4eVfC | outcome | Clostridium E sporosphaeroides abundance in stool | -0.037395207 | 0.0224554151247792 | 0.130205640812339 |
| GCST90032323 | T4eVfC | outcome | Clostridium I abundance in stool | 0.0075719321441738 | 0.0537614618634017 | 0.892603436503056 |
| GCST90032324 | T4eVfC | outcome | Clostridium M clostridioforme abundance in stool | 0.0204534516120702 | 0.0248012223229316 | 0.430858715147611 |
| GCST90032325 | T4eVfC | outcome | Clostridium M sp001304855 abundance in stool | -0.043391866 | 0.0876896109492025 | 0.654688417196034 |
| GCST90032326 | T4eVfC | outcome | Clostridium P abundance in stool | -0.013982728 | 0.0438175530133599 | 0.756207223391336 |
| GCST90032327 | T4eVfC | outcome | Clostridium saudiense abundance in stool | -0.042431697 | 0.0328604763082663 | 0.219099403885372 |
| GCST90032328 | T4eVfC | outcome | Clostridium S felsineum abundance in stool | 0.00147112663503824 | 0.0339214356007207 | 0.968132802584564 |
| GCST90032329 | T4eVfC | outcome | Clostridium tertium abundance in stool | -0.01659907 | 0.0766239783189267 | 0.833918684001617 |
| GCST90032330 | T4eVfC | outcome | Collinsella abundance in stool | -0.025870715 | 0.0443170486583139 | 0.572310071542163 |
| GCST90032331 | T4eVfC | outcome | Comamonas B abundance in stool | 0.0149425923855491 | 0.0311994549181171 | 0.646582085506454 |
| GCST90032332 | T4eVfC | outcome | Comamonas abundance in stool | 0.00687988842399443 | 0.0470496224934777 | 0.886648536387513 |
| GCST90032333 | T4eVfC | outcome | Coprobacillus cateniformis abundance in stool | -0.000800712 | 0.0559618877180994 | 0.988896281687753 |
| GCST90032334 | T4eVfC | outcome | Coprobacillus abundance in stool | 0.022832828202334 | 0.0675071090137841 | 0.745108726709288 |
| GCST90032335 | T4eVfC | outcome | Coprobacter secundus abundance in stool | -0.026258073 | 0.0832437737186456 | 0.768202361635762 |
| GCST90032336 | T4eVfC | outcome | Corynebacterium abundance in stool | 0.00445033536569328 | 0.0297214402463679 | 0.883683945482633 |
| GCST90032337 | T4eVfC | outcome | Cyanobacteria abundance in stool | -0.018976019 | 0.0235201968315429 | 0.44310595312321 |
| GCST90032338 | T4eVfC | outcome | Demequinaceae abundance in stool | 0.040646954867166 | 0.0394600202548382 | 0.333106342951288 |
| GCST90032339 | T4eVfC | outcome | Demequina abundance in stool | 0.0387419086826302 | 0.0244484654272841 | 0.141355989173799 |
| GCST90032340 | T4eVfC | outcome | Desulfobacterota A abundance in stool | -0.033863214 | 0.0331392808146552 | 0.328801993320244 |
| GCST90032341 | T4eVfC | outcome | Desulfovibrionaceae abundance in stool | -0.027455097 | 0.0299993701779929 | 0.379717544149622 |
| GCST90032342 | T4eVfC | outcome | Desulfovibrionales abundance in stool | -0.022124826 | 0.031589148158311 | 0.497037283352624 |
| GCST90032343 | T4eVfC | outcome | Desulfovibrionia abundance in stool | -0.035005346 | 0.0339537671188747 | 0.324690254245685 |
| GCST90032344 | T4eVfC | outcome | Desulfovibrio piger abundance in stool | -0.027311366 | 0.0265994195505125 | 0.331334643155027 |
| GCST90032345 | T4eVfC | outcome | Dialister sp000434475 abundance in stool | -0.017329275 | 0.0336530249959359 | 0.614628856366131 |
| GCST90032346 | T4eVfC | outcome | Dokdonella abundance in stool | 0.0400424553796525 | 0.0600284466353376 | 0.516400975369503 |
| GCST90032347 | T4eVfC | outcome | Dorea phocaeense abundance in stool | 0.0188927364562612 | 0.037661515694265 | 0.625805033794354 |
| GCST90032348 | T4eVfC | outcome | Dorea abundance in stool | -0.06907289 | 0.070735511844914 | 0.3665310480026 |
| GCST90032349 | T4eVfC | outcome | DTU024 sp002411105 abundance in stool | -0.003569026 | 0.0371456998525215 | 0.925561060525264 |
| GCST90032350 | T4eVfC | outcome | Dysgonomonadaceae abundance in stool | 0.0140675720125466 | 0.0474601061855555 | 0.775519377904974 |
| GCST90032351 | T4eVfC | outcome | Eisenbergiella sp900066775 abundance in stool | -0.027653213 | 0.0296094523513032 | 0.374716696179724 |
| GCST90032352 | T4eVfC | outcome | Elusimicrobiaceae abundance in stool | 0.0480143350095799 | 0.0218693302644809 | 0.0454813545579685 |
| GCST90032353 | T4eVfC | outcome | Elusimicrobia abundance in stool | -0.026715755 | 0.0253128878518338 | 0.305193691894507 |
| GCST90032354 | T4eVfC | outcome | Elusimicrobiota abundance in stool | -0.035523691 | 0.0236587408713257 | 0.146268672218375 |
| GCST90032355 | T4eVfC | outcome | Emergencia abundance in stool | -0.039772732 | 0.0767789896421509 | 0.620412285907612 |
| GCST90032356 | T4eVfC | outcome | Endozoicomonadaceae abundance in stool | 0.00392507154280258 | 0.0341642212880724 | 0.911759703337746 |
| GCST90032357 | T4eVfC | outcome | Enorma massiliensis abundance in stool | 0.0475055833254354 | 0.0454210355464764 | 0.322885967168805 |
| GCST90032358 | T4eVfC | outcome | Ensifer abundance in stool | -0.013716296 | 0.0759188355797885 | 0.861745713781892 |
| GCST90032359 | T4eVfC | outcome | Enterobacteriaceae abundance in stool | 0.0580610795159022 | 0.0528384327701249 | 0.321910064689769 |
| GCST90032360 | T4eVfC | outcome | Enterococcaceae abundance in stool | -0.012000026 | 0.0353342530063321 | 0.739187886154999 |
| GCST90032361 | T4eVfC | outcome | Enterococcus A abundance in stool | 0.0803940519146894 | 0.0275827971940922 | 0.0140716324595946 |
| GCST90032362 | T4eVfC | outcome | Enterococcus B abundance in stool | 0.00914422006353256 | 0.0515151685692338 | 0.862655406008144 |
| GCST90032363 | T4eVfC | outcome | Enterococcus faecalis abundance in stool | 0.0316926694578206 | 0.0330357150500114 | 0.360005139146968 |
| GCST90032364 | T4eVfC | outcome | Enterococcus abundance in stool | 0.042594307535098 | 0.04964758834187 | 0.423869711078516 |
| GCST90032365 | T4eVfC | outcome | Enteroscipio abundance in stool | -0.029569766 | 0.0359205739727372 | 0.426452315180885 |
| GCST90032366 | T4eVfC | outcome | ER4 sp002437735 abundance in stool | -0.021733919 | 0.0372950326212131 | 0.576110419285596 |
| GCST90032367 | T4eVfC | outcome | Eremiobacterota abundance in stool | 0.000657187979406644 | 0.0541391303123667 | 0.990579644785161 |
| GCST90032368 | T4eVfC | outcome | Erysipelatoclostridiaceae abundance in stool | 0.0310282064324443 | 0.0448851742072087 | 0.511656716623053 |
| GCST90032369 | T4eVfC | outcome | Escherichia flexneri abundance in stool | 0.02683676513706 | 0.0328326168614048 | 0.440639941498832 |
| GCST90032370 | T4eVfC | outcome | Escherichia abundance in stool | 0.016934606966555 | 0.0710801012865618 | 0.821145645970421 |
| GCST90032371 | T4eVfC | outcome | Eubacterium callanderi abundance in stool | 0.00849535490609574 | 0.0257008296119011 | 0.749476634368335 |
| GCST90032372 | T4eVfC | outcome | Eubacterium F sp000434115 abundance in stool | 0.0575597671396781 | 0.0532339686873257 | 0.307701270771929 |
| GCST90032373 | T4eVfC | outcome | Eubacterium I ramulus A abundance in stool | -0.013243588 | 0.0282224040638073 | 0.646658057013386 |
| GCST90032374 | T4eVfC | outcome | Eubacterium Q abundance in stool | -0.059643216 | 0.0310606660815323 | 0.0910850660592237 |
| GCST90032375 | T4eVfC | outcome | Eubacterium R coprostanoligenes abundance in stool | 0.0684471942085474 | 0.0729443141278856 | 0.401191627275663 |
| GCST90032376 | T4eVfC | outcome | Eubacterium R sp000431535 abundance in stool | 0.0483772784329438 | 0.0446766915327675 | 0.30704011594243 |
| GCST90032377 | T4eVfC | outcome | Ezakiellaceae abundance in stool | -0.016193006 | 0.0401832842728824 | 0.70757336279747 |
| GCST90032378 | T4eVfC | outcome | F0428 abundance in stool | -0.041720214 | 0.0289039696831681 | 0.179491424441784 |
| GCST90032379 | T4eVfC | outcome | Faecalibacterium prausnitzii E abundance in stool | 0.0729684960861718 | 0.062542371102671 | 0.281533723405407 |
| GCST90032380 | T4eVfC | outcome | Faecalibacterium sp002160895 abundance in stool | -0.017306517 | 0.0353031580759019 | 0.635703065124432 |
| GCST90032381 | T4eVfC | outcome | Faecalicatena glycyrrhizinilyticum abundance in stool | 0.0183445828458203 | 0.0315379649813244 | 0.570740386650488 |
| GCST90032382 | T4eVfC | outcome | Faecalicatena lactaris abundance in stool | 0.00901627454963346 | 0.0532450233886382 | 0.869277653703078 |
| GCST90032383 | T4eVfC | outcome | Faecalicatena sp000364245 abundance in stool | 0.00869524752285599 | 0.0625876904268603 | 0.89405200049151 |
| GCST90032384 | T4eVfC | outcome | Faecalicatena sp001517425 abundance in stool | -0.028316328 | 0.0393624240851667 | 0.485684509688479 |
| GCST90032385 | T4eVfC | outcome | Faecalicatena sp002161355 abundance in stool | -0.120914738 | 0.0747504096679517 | 0.166676901374144 |
| GCST90032386 | T4eVfC | outcome | Faecalicatena sp002397985 abundance in stool | 0.0104993597585158 | 0.0304237753483527 | 0.741792627491215 |
| GCST90032387 | T4eVfC | outcome | Faecalicatena torques abundance in stool | 0.0514282392261899 | 0.027716312319698 | 0.0931972464641512 |
| GCST90032388 | T4eVfC | outcome | Faecalicoccus pleomorphus abundance in stool | -0.011975404 | 0.0283402181642709 | 0.690173492268261 |
| GCST90032389 | T4eVfC | outcome | Faecalicoccus abundance in stool | -0.016150893 | 0.0383320720443303 | 0.691005476044796 |
| GCST90032390 | T4eVfC | outcome | Faecalitalea cylindroides abundance in stool | -0.164637664 | 0.0929252572814056 | 0.151135881918364 |
| GCST90032391 | T4eVfC | outcome | Fervidobacteriaceae abundance in stool | -0.105866746 | 0.0433666870013545 | 0.0372911758944558 |
| GCST90032392 | T4eVfC | outcome | Fibrobacteraceae abundance in stool | -0.011170862 | 0.029610150174688 | 0.715788350284572 |
| GCST90032393 | T4eVfC | outcome | Fibrobacterales abundance in stool | -0.023812146 | 0.0321627656370627 | 0.487028953310238 |
| GCST90032394 | T4eVfC | outcome | Fibrobacteria abundance in stool | -0.014751634 | 0.026629408896428 | 0.591776869806754 |
| GCST90032395 | T4eVfC | outcome | Fimbriimonadia abundance in stool | 0.000112781112311089 | 0.0329048157158831 | 0.997321580268211 |
| GCST90032396 | T4eVfC | outcome | Firmicutes A abundance in stool | -0.048130264 | 0.0672379286084109 | 0.492255404938825 |
| GCST90032397 | T4eVfC | outcome | Firmicutes E abundance in stool | -0.072510334 | 0.0522364423528131 | 0.207674742535366 |
| GCST90032398 | T4eVfC | outcome | Firmicutes I abundance in stool | -0.058088172 | 0.0477231336709929 | 0.251475900022936 |
| GCST90032399 | T4eVfC | outcome | Flavobacteriales abundance in stool | 0.00585341028816058 | 0.0213690629848373 | 0.78845025339472 |
| GCST90032400 | T4eVfC | outcome | Flavonifractor sp002159265 abundance in stool | -0.094272784 | 0.108110632010784 | 0.412114858848189 |
| GCST90032401 | T4eVfC | outcome | Flavonifractor sp900199495 abundance in stool | 0.0152926493948808 | 0.0451362913168357 | 0.741131816074288 |
| GCST90032402 | T4eVfC | outcome | Fournierella massiliensis abundance in stool | 0.0272054285652523 | 0.0993591079246935 | 0.791167990755204 |
| GCST90032403 | T4eVfC | outcome | Francisellaceae abundance in stool | 0.0154243421129445 | 0.0612254794875329 | 0.809504980310995 |
| GCST90032404 | T4eVfC | outcome | Francisellales abundance in stool | 0.0609700354594272 | 0.0749335828181636 | 0.452833222411281 |
| GCST90032405 | T4eVfC | outcome | Fusobacteriaceae abundance in stool | 0.00302485255262591 | 0.0302705483146617 | 0.922860861413239 |
| GCST90032406 | T4eVfC | outcome | Fusobacterium A abundance in stool | -0.018155947 | 0.0334917453719687 | 0.604567681271993 |
| GCST90032407 | T4eVfC | outcome | GCA-900066135 sp900066135 abundance in stool | 0.00313761042468682 | 0.0491404404942273 | 0.950485427418834 |
| GCST90032408 | T4eVfC | outcome | GCA-900066495 sp900066495 abundance in stool | 0.0305039341951622 | 0.0363429310184112 | 0.415383646364573 |
| GCST90032409 | T4eVfC | outcome | GCA-900066495 abundance in stool | 0.0312032503659069 | 0.030173678881395 | 0.321470814980199 |
| GCST90032410 | T4eVfC | outcome | GCA-900066575 sp900066385 abundance in stool | -0.001956737 | 0.0411368027980734 | 0.962998237632209 |
| GCST90032411 | T4eVfC | outcome | GCA-900066755 sp900066755 abundance in stool | 0.0144142074208033 | 0.307911356094823 | 0.965604379882923 |
| GCST90032412 | T4eVfC | outcome | GCA-900066755 abundance in stool | 0.0497807990135323 | 0.0665602185858763 | 0.473605465126951 |
| GCST90032413 | T4eVfC | outcome | GCA-900199385 sp900320755 abundance in stool | 0.0533905621859976 | 0.113843261830123 | 0.663501206298875 |
| GCST90032414 | T4eVfC | outcome | Geminocystis abundance in stool | -5.06E-05 | 0.0374960229163403 | 0.998967823840615 |
| GCST90032415 | T4eVfC | outcome | Gemmatimonadaceae abundance in stool | -0.032400065 | 0.0366134162219067 | 0.39358426715872 |
| GCST90032416 | T4eVfC | outcome | Geobacteraceae abundance in stool | -0.046963011 | 0.0414474791765492 | 0.320494724934062 |
| GCST90032417 | T4eVfC | outcome | Geobacter C abundance in stool | 0.0313421079882538 | 0.0510599163187322 | 0.566176835998519 |
| GCST90032418 | T4eVfC | outcome | Gillisia abundance in stool | 0.054712651319442 | 0.0750507183590896 | 0.482720525538889 |
| GCST90032419 | T4eVfC | outcome | Gluconobacter abundance in stool | 0.000292430556422389 | 0.0275163374983055 | 0.991752471344635 |
| GCST90032420 | T4eVfC | outcome | Gordonibacter pamelaeae abundance in stool | 0.00522696391927943 | 0.0364868444973441 | 0.890777944828183 |
| GCST90032421 | T4eVfC | outcome | Gordonibacter abundance in stool | -0.059265756 | 0.0386301781714017 | 0.155992936863124 |
| GCST90032422 | T4eVfC | outcome | Gramella abundance in stool | 0.0149665341315196 | 0.0393462113596223 | 0.710905682128423 |
| GCST90032423 | T4eVfC | outcome | Haemophilus D sp001679485 abundance in stool | 0.0439115680392698 | 0.0273283823074364 | 0.152130899249083 |
| GCST90032424 | T4eVfC | outcome | Halarcobacter abundance in stool | -0.010016435 | 0.0371119050693815 | 0.796281362197545 |
| GCST90032425 | T4eVfC | outcome | Halomonadaceae abundance in stool | 0.0172990053993291 | 0.0256148871401793 | 0.514765796848671 |
| GCST90032426 | T4eVfC | outcome | Haloplasmatales abundance in stool | 0.0287722751786424 | 0.0435039404622029 | 0.526959178925282 |
| GCST90032427 | T4eVfC | outcome | Helicobacter abundance in stool | 0.0362136717683038 | 0.061159301599253 | 0.5753878252719 |
| GCST90032428 | T4eVfC | outcome | Herbidospora abundance in stool | -0.014249142 | 0.0240224988979111 | 0.567679826143262 |
| GCST90032429 | T4eVfC | outcome | Herbinix abundance in stool | 0.036853710936575 | 0.0323067680235773 | 0.283422004748001 |
| GCST90032430 | T4eVfC | outcome | Holdemania massiliensis abundance in stool | -0.066026509 | 0.0818277327849563 | 0.446271316234047 |
| GCST90032431 | T4eVfC | outcome | Holdemania sp900120005 abundance in stool | -0.029086485 | 0.0304716996236757 | 0.364743315290446 |
| GCST90032432 | T4eVfC | outcome | Holdemania abundance in stool | 0.0540038967387472 | 0.0440425447427645 | 0.251246162877826 |
| GCST90032433 | T4eVfC | outcome | Hungatella sp900155545 abundance in stool | -0.0183256 | 0.0877621392506227 | 0.842836884916982 |
| GCST90032434 | T4eVfC | outcome | Hydrogenophaga abundance in stool | -0.00566907 | 0.040213378442443 | 0.89250385575769 |
| GCST90032435 | T4eVfC | outcome | Hyphomonas abundance in stool | 0.0228321081222219 | 0.0474800166063078 | 0.647635636085129 |
| GCST90032436 | T4eVfC | outcome | Intestinimonas massiliensis abundance in stool | -0.103240613 | 0.0524943028010337 | 0.0899273051879762 |
| GCST90032437 | T4eVfC | outcome | Jiangellaceae abundance in stool | -0.083726953 | 0.0683818945609803 | 0.25563041821493 |
| GCST90032438 | T4eVfC | outcome | Johnsonella ignava abundance in stool | 0.0592482978553863 | 0.23967975597263 | 0.827815408968701 |
| GCST90032439 | T4eVfC | outcome | K10 sp001941205 abundance in stool | 0.0341475574733092 | 0.042884625708029 | 0.456218331295972 |
| GCST90032440 | T4eVfC | outcome | K10 abundance in stool | -0.001410634 | 0.0830927883418317 | 0.989193394815816 |
| GCST90032441 | T4eVfC | outcome | Kandleria vitulina abundance in stool | 0.00437550799110568 | 0.0350151905572307 | 0.904067946014331 |
| GCST90032442 | T4eVfC | outcome | Kineothrix abundance in stool | 0.0125688660696174 | 0.0313364199425108 | 0.694865320348026 |
| GCST90032443 | T4eVfC | outcome | KLE1615 sp900066985 abundance in stool | -0.021023595 | 0.0431691033007244 | 0.6411332879133 |
| GCST90032444 | T4eVfC | outcome | KLE1615 abundance in stool | 0.0248958318146159 | 0.0376062713201437 | 0.529146656175462 |
| GCST90032445 | T4eVfC | outcome | Klebsiella A abundance in stool | 0.0150977771579672 | 0.0185073475043655 | 0.427395255067484 |
| GCST90032446 | T4eVfC | outcome | Klebsiella pneumoniae abundance in stool | 0.00721710476645897 | 0.0317654122390663 | 0.825966670120743 |
| GCST90032447 | T4eVfC | outcome | Klebsiella abundance in stool | -0.003949782 | 0.0251649266858502 | 0.878123441036492 |
| GCST90032448 | T4eVfC | outcome | koll11 abundance in stool | -0.055541102 | 0.0404259000285183 | 0.227867392457166 |
| GCST90032449 | T4eVfC | outcome | Lachnoanaerobaculum saburreum abundance in stool | 0.0145522984187192 | 0.0392763469302814 | 0.729788242034144 |
| GCST90032450 | T4eVfC | outcome | Lachnospiraceae abundance in stool | 0.0556170469070253 | 0.112838780448593 | 0.639607570186538 |
| GCST90032451 | T4eVfC | outcome | Lachnospirales abundance in stool | 0.243811851698988 | 0.130599215572566 | 0.120898622928599 |
| GCST90032452 | T4eVfC | outcome | Lachnospira rogosae abundance in stool | -0.063717709 | 0.0493331878124086 | 0.232566885226183 |
| GCST90032453 | T4eVfC | outcome | Lachnospira sp000437735 abundance in stool | 0.0712801363024127 | 0.0535798142293057 | 0.254180743772144 |
| GCST90032454 | T4eVfC | outcome | Lactobacillus B ruminis abundance in stool | -0.000164564 | 0.0360295797861456 | 0.99650377701486 |
| GCST90032455 | T4eVfC | outcome | Lactobacillus B salivarius abundance in stool | 0.0194940294887598 | 0.023371540511751 | 0.415161375944162 |
| GCST90032456 | T4eVfC | outcome | Lactobacillus B abundance in stool | -0.004727124 | 0.0184024879920654 | 0.802483722995237 |
| GCST90032457 | T4eVfC | outcome | Lactococcus lactis abundance in stool | 0.0188554492857638 | 0.0499937822816584 | 0.725214243582232 |
| GCST90032458 | T4eVfC | outcome | Lawsonibacter sp000492175 abundance in stool | 0.00293611371040035 | 0.0421171570322318 | 0.946133176712026 |
| GCST90032459 | T4eVfC | outcome | Lawsonibacter sp002161175 abundance in stool | 0.0925304831558509 | 0.0353243856413534 | 0.0344366943920254 |
| GCST90032460 | T4eVfC | outcome | Lawsonibacter sp900066645 abundance in stool | 0.00751060085999824 | 0.0424987552332943 | 0.862447792919462 |
| GCST90032461 | T4eVfC | outcome | Leclercia abundance in stool | -0.016338127 | 0.0294817466810154 | 0.59670846213014 |
| GCST90032462 | T4eVfC | outcome | Lentimicrobiaceae abundance in stool | -0.110373535 | 0.106490894757583 | 0.327032045027516 |
| GCST90032463 | T4eVfC | outcome | Leptospirae abundance in stool | 0.0239977637621397 | 0.0398802721903728 | 0.555775143603357 |
| GCST90032464 | T4eVfC | outcome | Leptospirales abundance in stool | -0.018680858 | 0.0446623602908883 | 0.681310887511232 |
| GCST90032465 | T4eVfC | outcome | Leuconostoc mesenteroides abundance in stool | -0.023402488 | 0.0485091353801718 | 0.654709321761239 |
| GCST90032466 | T4eVfC | outcome | Leuconostoc abundance in stool | -0.015986964 | 0.0304005517067494 | 0.610436204969514 |
| GCST90032467 | T4eVfC | outcome | Magnetospirillum A abundance in stool | -0.000869189 | 0.0837162432527606 | 0.992213251545716 |
| GCST90032468 | T4eVfC | outcome | Marinilabiliaceae abundance in stool | 0.0256250415080529 | 0.0518590649397883 | 0.638782440562356 |
| GCST90032469 | T4eVfC | outcome | Massiliomicrobiota sp002160815 abundance in stool | -0.006349186 | 0.0299906779498309 | 0.835619871266417 |
| GCST90032470 | T4eVfC | outcome | Massiliomicrobiota abundance in stool | -0.066227197 | 0.0730254454349876 | 0.390954776339334 |
| GCST90032471 | T4eVfC | outcome | Megamonas funiformis abundance in stool | -0.030941951 | 0.0259410279796134 | 0.252779427526342 |
| GCST90032472 | T4eVfC | outcome | Megamonas abundance in stool | -0.023326985 | 0.0206169378139802 | 0.274540766937945 |
| GCST90032473 | T4eVfC | outcome | Megasphaera elsdenii abundance in stool | -0.024275246 | 0.0229791180524649 | 0.313432586093303 |
| GCST90032474 | T4eVfC | outcome | Megasphaera sp900066485 abundance in stool | -0.045430759 | 0.0292897405610406 | 0.171863803434452 |
| GCST90032475 | T4eVfC | outcome | Megasphaera abundance in stool | -0.02065573 | 0.0206223689057617 | 0.331436567835335 |
| GCST90032476 | T4eVfC | outcome | Merdibacter massiliensis abundance in stool | -0.001771545 | 0.0869917818311443 | 0.984540273954572 |
| GCST90032477 | T4eVfC | outcome | Methanobacterium B abundance in stool | 0.0241381505246301 | 0.0491616642100204 | 0.649148963503393 |
| GCST90032478 | T4eVfC | outcome | Methanobrevibacter B abundance in stool | 0.069403128257624 | 0.0654008853564068 | 0.323836400961152 |
| GCST90032479 | T4eVfC | outcome | Microvirga abundance in stool | 0.133441749483567 | 0.137649619176817 | 0.403836739826665 |
| GCST90032480 | T4eVfC | outcome | Monoglobaceae abundance in stool | -0.031149498 | 0.0357584867242924 | 0.404123056772967 |
| GCST90032481 | T4eVfC | outcome | Monoglobus pectinilyticus abundance in stool | -0.020546439 | 0.036253746757792 | 0.581336898430649 |
| GCST90032482 | T4eVfC | outcome | Monoglobus abundance in stool | -0.015818288 | 0.0320475024415188 | 0.631303493138555 |
| GCST90032483 | T4eVfC | outcome | Morganella abundance in stool | 0.0641001749113175 | 0.0230995328493401 | 0.012970693933839 |
| GCST90032484 | T4eVfC | outcome | Mycobacteriaceae abundance in stool | 0.017440072065089 | 0.0465483938269591 | 0.716585218196475 |
| GCST90032485 | T4eVfC | outcome | Mycoplasmataceae abundance in stool | 0.113674633507289 | 0.0707525806783237 | 0.183406987688572 |
| GCST90032486 | T4eVfC | outcome | Mycoplasmatales abundance in stool | -0.005592717 | 0.0535543751429191 | 0.919398339371222 |
| GCST90032487 | T4eVfC | outcome | Mycoplasmoidaceae abundance in stool | -0.019416112 | 0.0241231905081662 | 0.435366610360743 |
| GCST90032488 | T4eVfC | outcome | Negativibacillus massiliensis abundance in stool | 0.0117568313649286 | 0.0341428185106435 | 0.738497701638386 |
| GCST90032489 | T4eVfC | outcome | Negativibacillus sp000435195 abundance in stool | 0.0398524813724054 | 0.0404307701965289 | 0.350037914087571 |
| GCST90032490 | T4eVfC | outcome | Negativibacillus abundance in stool | 0.0902330262817925 | 0.0434184596327068 | 0.0829390010731285 |
| GCST90032491 | T4eVfC | outcome | NK4A144 abundance in stool | -0.0063788 | 0.0263217165341576 | 0.814613999249096 |
| GCST90032492 | T4eVfC | outcome | Odoribacter laneus abundance in stool | -0.005538779 | 0.0346870151324939 | 0.877092073352775 |
| GCST90032493 | T4eVfC | outcome | Olsenella C abundance in stool | 0.0361959217998683 | 0.0794035806603073 | 0.672150218880934 |
| GCST90032494 | T4eVfC | outcome | Omnitrophota abundance in stool | 0.0410312368849688 | 0.0501919882297324 | 0.434757655281751 |
| GCST90032495 | T4eVfC | outcome | Paceibacteria abundance in stool | 0.0678399603701559 | 0.0399080331804411 | 0.127570750494918 |
| GCST90032496 | T4eVfC | outcome | Paenibacillales abundance in stool | -0.013545001 | 0.0423435500756023 | 0.765068113145093 |
| GCST90032497 | T4eVfC | outcome | Paenibacillus J abundance in stool | 0.00226886600500333 | 0.0253030128665756 | 0.930321699098249 |
| GCST90032498 | T4eVfC | outcome | Pandoraea abundance in stool | 0.0434848362157346 | 0.0676221218830298 | 0.536225293982237 |
| GCST90032499 | T4eVfC | outcome | Parabacteroides johnsonii abundance in stool | 0.00604483605845099 | 0.0419087203428814 | 0.88752447792693 |
| GCST90032500 | T4eVfC | outcome | Parabacteroides sp000436495 abundance in stool | 0.0092754691432551 | 0.0507883423757039 | 0.862262533038712 |
| GCST90032501 | T4eVfC | outcome | Parabacteroides abundance in stool | -0.051445921 | 0.0453938423872633 | 0.276113998561806 |
| GCST90032502 | T4eVfC | outcome | Parachlamydiales abundance in stool | -0.043386289 | 0.0386696491306572 | 0.312844846476735 |
| GCST90032503 | T4eVfC | outcome | Paraglaciecola abundance in stool | -0.006957898 | 0.0526325648641567 | 0.897450115451531 |
| GCST90032504 | T4eVfC | outcome | Paramuribaculum sp001689565 abundance in stool | -0.038027634 | 0.0639203340813329 | 0.573631284820485 |
| GCST90032505 | T4eVfC | outcome | Pararhizobium abundance in stool | 0.0572712380771857 | 0.045496991170459 | 0.236697799565609 |
| GCST90032506 | T4eVfC | outcome | Pauljensenia sp000411415 abundance in stool | 0.011855015639734 | 0.0326774962664143 | 0.726161015064012 |
| GCST90032507 | T4eVfC | outcome | Peptococcia abundance in stool | -0.037229808 | 0.029457650694168 | 0.246745665952812 |
| GCST90032508 | T4eVfC | outcome | Phascolarctobacterium sp003150755 abundance in stool | 0.0118442547007129 | 0.0259627291953405 | 0.659053564850195 |
| GCST90032509 | T4eVfC | outcome | Phocea massiliensis abundance in stool | 0.00898062681016752 | 0.0631649959913344 | 0.895951411231582 |
| GCST90032510 | T4eVfC | outcome | Phocea abundance in stool | -0.226952609 | 0.124422446041804 | 0.209705747872947 |
| GCST90032511 | T4eVfC | outcome | Photobacterium abundance in stool | -0.112258898 | 0.103704050305428 | 0.474795030741966 |
| GCST90032512 | T4eVfC | outcome | Planococcaceae abundance in stool | 0.000328630092550736 | 0.0808676337688953 | 0.997126469556171 |
| GCST90032513 | T4eVfC | outcome | Poseidoniaceae abundance in stool | -0.009220896 | 0.0651903664398569 | 0.891502504424385 |
| GCST90032514 | T4eVfC | outcome | Prevotella bivia abundance in stool | -0.022915266 | 0.0335937828378058 | 0.525468882396345 |
| GCST90032515 | T4eVfC | outcome | Prevotella buccae abundance in stool | -0.008705505 | 0.0825926111626356 | 0.920154007525123 |
| GCST90032516 | T4eVfC | outcome | Prevotellamassilia sp000437675 abundance in stool | 0.00467319943535781 | 0.0457963972040811 | 0.920959935552154 |
| GCST90032517 | T4eVfC | outcome | Prevotellamassilia abundance in stool | -0.079079597 | 0.0446949055364261 | 0.137067596432746 |
| GCST90032518 | T4eVfC | outcome | Prevotella sp000434975 abundance in stool | -0.013476314 | 0.0696383377745174 | 0.858914195040632 |
| GCST90032519 | T4eVfC | outcome | Prevotella sp000436915 abundance in stool | -0.051077633 | 0.0322162374871889 | 0.173717874025196 |
| GCST90032520 | T4eVfC | outcome | Prevotella sp002437285 abundance in stool | 0.0136943606375922 | 0.0355347466109488 | 0.713248558100255 |
| GCST90032521 | T4eVfC | outcome | Prevotella sp002437565 abundance in stool | -0.025030705 | 0.0317392921978216 | 0.446989250900351 |
| GCST90032522 | T4eVfC | outcome | Prevotella sp002933775 abundance in stool | 0.0438253973712174 | 0.040274350188528 | 0.318286343772328 |
| GCST90032523 | T4eVfC | outcome | Prevotella sp900317685 abundance in stool | 0.00965180539378321 | 0.0866528148318667 | 0.914943893327179 |
| GCST90032524 | T4eVfC | outcome | Prevotella sp900318625 abundance in stool | -0.295664636 | 0.178679591067067 | 0.346065670138831 |
| GCST90032525 | T4eVfC | outcome | Propionibacterium freudenreichii abundance in stool | -0.059620981 | 0.0568840954384476 | 0.329420837177792 |
| GCST90032526 | T4eVfC | outcome | Proteus abundance in stool | 0.00899295567482572 | 0.0337146843344862 | 0.794200043055377 |
| GCST90032527 | T4eVfC | outcome | Provencibacterium massiliense abundance in stool | -0.011776506 | 0.0514739289593836 | 0.823646303853176 |
| GCST90032528 | T4eVfC | outcome | Provencibacterium abundance in stool | -0.023353335 | 0.0508286951425046 | 0.65412186992149 |
| GCST90032529 | T4eVfC | outcome | Providencia abundance in stool | -0.022274043 | 0.0227874597056698 | 0.353877819465185 |
| GCST90032530 | T4eVfC | outcome | Pseudomonadales abundance in stool | -0.037892839 | 0.0356315968353418 | 0.322876744714371 |
| GCST90032531 | T4eVfC | outcome | Pseudomonas aeruginosa abundance in stool | 0.109267581780718 | 0.0696738344088575 | 0.167865080474859 |
| GCST90032532 | T4eVfC | outcome | Psychroserpens abundance in stool | -0.014502304 | 0.0305096191196668 | 0.64308307592528 |
| GCST90032533 | T4eVfC | outcome | QALR01 sp003150035 abundance in stool | -0.06746334 | 0.244569798518745 | 0.800577252834174 |
| GCST90032534 | T4eVfC | outcome | Raoultella abundance in stool | 0.00714622689090975 | 0.0331944961809861 | 0.834345771672435 |
| GCST90032535 | T4eVfC | outcome | RC9 sp900317925 abundance in stool | -0.006219475 | 0.0636023671203617 | 0.924842662106619 |
| GCST90032536 | T4eVfC | outcome | Rhodanobacter abundance in stool | 0.083936074539661 | 0.0438135460658485 | 0.081739100089188 |
| GCST90032537 | T4eVfC | outcome | Rhodococcus abundance in stool | -0.080159169 | 0.0549611666746537 | 0.178708225849317 |
| GCST90032538 | T4eVfC | outcome | Rhodovulum abundance in stool | 0.261839647660885 | 0.243353413974873 | 0.360768278986553 |
| GCST90032539 | T4eVfC | outcome | Romboutsia ilealis abundance in stool | -0.037346988 | 0.0468182978144667 | 0.448072294775645 |
| GCST90032540 | T4eVfC | outcome | Roseibacillus abundance in stool | NA | NA | NA |
| GCST90032541 | T4eVfC | outcome | Rubneribacter sp002159915 abundance in stool | 0.0695741606175211 | 0.106022623348976 | 0.547513992759961 |
| GCST90032542 | T4eVfC | outcome | RUG013 sp001486445 abundance in stool | -0.022802531 | 0.128312001583905 | 0.863982701384278 |
| GCST90032543 | T4eVfC | outcome | RUG147 sp900315495 abundance in stool | -0.087243474 | 0.0515075214104048 | 0.23238374042807 |
| GCST90032544 | T4eVfC | outcome | RUG147 abundance in stool | -0.085073954 | 0.0860067209264244 | 0.42684677013438 |
| GCST90032545 | T4eVfC | outcome | RUG420 sp900317985 abundance in stool | -0.003599148 | 0.0469008856924912 | 0.940344486186992 |
| GCST90032546 | T4eVfC | outcome | RUG472 sp900319345 abundance in stool | 0.105766340656721 | 0.0761649445082982 | 0.21429485808351 |
| GCST90032547 | T4eVfC | outcome | RUG472 abundance in stool | 0.0577040059768037 | 0.0501284247104192 | 0.313809431833368 |
| GCST90032548 | T4eVfC | outcome | Ruminococcus A sp000432335 abundance in stool | -0.124706565 | 0.0618192749792788 | 0.137016186942655 |
| GCST90032549 | T4eVfC | outcome | Ruminococcus C sp000437255 abundance in stool | 0.0201119678198002 | 0.0329244780766835 | 0.554927240438195 |
| GCST90032550 | T4eVfC | outcome | Ruminococcus D bicirculans abundance in stool | -0.01509671 | 0.0569675409864939 | 0.795502503934555 |
| GCST90032551 | T4eVfC | outcome | Ruminococcus D abundance in stool | -0.028082048 | 0.0517413751971508 | 0.59649236145911 |
| GCST90032552 | T4eVfC | outcome | Ruminococcus E sp003521625 abundance in stool | -0.029997015 | 0.0518567866492575 | 0.583998318566513 |
| GCST90032553 | T4eVfC | outcome | Ruminococcus E sp900100595 abundance in stool | 0.00227627967809401 | 0.0304395146446657 | 0.941864221630847 |
| GCST90032554 | T4eVfC | outcome | Ruminococcus E sp900314705 abundance in stool | -0.035198802 | 0.0335817696569851 | 0.311164502367112 |
| GCST90032555 | T4eVfC | outcome | Ruminococcus abundance in stool | -0.034811054 | 0.082227803168796 | 0.693811468119618 |
| GCST90032556 | T4eVfC | outcome | Saccharofermentanaceae abundance in stool | -0.021178508 | 0.0667266233276551 | 0.76377191484554 |
| GCST90032557 | T4eVfC | outcome | Saccharomonospora abundance in stool | 0.00869897660554653 | 0.0327367616158149 | 0.793646667318227 |
| GCST90032558 | T4eVfC | outcome | SAR324 abundance in stool | -0.005095738 | 0.0370904455567717 | 0.895219478998334 |
| GCST90032559 | T4eVfC | outcome | SM23-33 abundance in stool | -0.008928855 | 0.043205845865947 | 0.839251985223551 |
| GCST90032560 | T4eVfC | outcome | Sorangium abundance in stool | -0.036604852 | 0.0640899020485314 | 0.58863685310898 |
| GCST90032561 | T4eVfC | outcome | Spirillospora abundance in stool | -0.027917698 | 0.0343601073304105 | 0.432326899558291 |
| GCST90032562 | T4eVfC | outcome | Spirochaetia abundance in stool | 0.0163429826621153 | 0.0291155909216146 | 0.585837087962269 |
| GCST90032563 | T4eVfC | outcome | Sporomusales abundance in stool | -0.036226991 | 0.0227520606669558 | 0.172205613472986 |
| GCST90032564 | T4eVfC | outcome | Staphylococcus A fleurettii abundance in stool | 0.033740590343989 | 0.033349714899361 | 0.341289394960146 |
| GCST90032565 | T4eVfC | outcome | Staphylococcus aureus abundance in stool | -0.089222102 | 0.0807948229913123 | 0.305968661066131 |
| GCST90032566 | T4eVfC | outcome | Stappia abundance in stool | 0.00704987725130538 | 0.0253954399809302 | 0.789336221241539 |
| GCST90032567 | T4eVfC | outcome | Streptacidiphilus abundance in stool | 0.00288609917495676 | 0.0541699493078996 | 0.95855926400415 |
| GCST90032568 | T4eVfC | outcome | Streptococcus sanguinis abundance in stool | 0.0197960619922128 | 0.033755670417562 | 0.578951578090188 |
| GCST90032569 | T4eVfC | outcome | Succiniclasticum abundance in stool | -0.02449849 | 0.0239388426844811 | 0.32047213697696 |
| GCST90032570 | T4eVfC | outcome | Succinivibrionaceae abundance in stool | 0.0323722753597331 | 0.0214102031351162 | 0.154456514862347 |
| GCST90032571 | T4eVfC | outcome | Succinivibrio abundance in stool | 0.0116706247679787 | 0.0487475705142533 | 0.814521375564169 |
| GCST90032572 | T4eVfC | outcome | Syntrophomonadia abundance in stool | 0.00889123462170755 | 0.0267810279080452 | 0.749610556370673 |
| GCST90032573 | T4eVfC | outcome | Syntrophorhabdaceae abundance in stool | -0.008034665 | 0.0230430207475652 | 0.735346741368298 |
| GCST90032574 | T4eVfC | outcome | Syntrophorhabdia abundance in stool | -0.006292919 | 0.0484726794036904 | 0.90035746992932 |
| GCST90032575 | T4eVfC | outcome | Tannerellaceae abundance in stool | -0.06530638 | 0.0398657849501833 | 0.125353432397555 |
| GCST90032576 | T4eVfC | outcome | Tepidanaerobacteraceae abundance in stool | 0.0231589870143474 | 0.07573850287152 | 0.770106872498472 |
| GCST90032577 | T4eVfC | outcome | Terrisporobacter othiniensis abundance in stool | 0.0462698355407188 | 0.0667864105245329 | 0.538247439511849 |
| GCST90032578 | T4eVfC | outcome | Terrisporobacter abundance in stool | 0.024842067260445 | 0.055279347372362 | 0.665074686387879 |
| GCST90032579 | T4eVfC | outcome | Thermococcaceae abundance in stool | -0.056576546 | 0.0510681839171852 | 0.300114944175411 |
| GCST90032580 | T4eVfC | outcome | Thermococci abundance in stool | -0.039846494 | 0.0680485295054401 | 0.579513204986039 |
| GCST90032581 | T4eVfC | outcome | Thermoplasmatota abundance in stool | -0.014334423 | 0.0277175234116954 | 0.615283356845812 |
| GCST90032582 | T4eVfC | outcome | Thermoprotei abundance in stool | 0.0632593503573575 | 0.0652131094764931 | 0.364341964361868 |
| GCST90032583 | T4eVfC | outcome | Thioalkalivibrionaceae abundance in stool | 0.00184257442263761 | 0.037361897568284 | 0.963766441821126 |
| GCST90032584 | T4eVfC | outcome | TMED109 abundance in stool | -0.010199176 | 0.0907869848531826 | 0.913018146212102 |
| GCST90032585 | T4eVfC | outcome | Treponema D abundance in stool | -0.003801496 | 0.028131876611368 | 0.893858844567332 |
| GCST90032586 | T4eVfC | outcome | Treponemataceae abundance in stool | 0.0384446300489597 | 0.0227178960240937 | 0.106931085934102 |
| GCST90032587 | T4eVfC | outcome | Turicibacteraceae abundance in stool | 0.0607906269883924 | 0.0675022401702585 | 0.418735046563369 |
| GCST90032588 | T4eVfC | outcome | Turicibacter sp001543345 abundance in stool | 0.0630541687296716 | 0.0426820020832291 | 0.16535309994341 |
| GCST90032589 | T4eVfC | outcome | Turicibacter abundance in stool | 0.0463171994042173 | 0.0680598964085573 | 0.521545977811198 |
| GCST90032590 | T4eVfC | outcome | UBA1033 sp001695555 abundance in stool | 0.0663736417191717 | 0.0517469396860778 | 0.240448353208055 |
| GCST90032591 | T4eVfC | outcome | UBA1066 sp900317515 abundance in stool | -0.104360817 | 0.127615292952618 | 0.563605542921136 |
| GCST90032592 | T4eVfC | outcome | UBA1066 abundance in stool | 0.0401316342864579 | 0.036696050447728 | 0.293971333513051 |
| GCST90032593 | T4eVfC | outcome | UBA11471 sp000434215 abundance in stool | -0.06400179 | 0.0798239432122152 | 0.449033821274559 |
| GCST90032594 | T4eVfC | outcome | UBA11471 abundance in stool | -0.06331386 | 0.0802975786776951 | 0.456278248147713 |
| GCST90032595 | T4eVfC | outcome | UBA1191 abundance in stool | -0.010595294 | 0.0263520310956342 | 0.701565607757037 |
| GCST90032596 | T4eVfC | outcome | UBA11963 sp002362595 abundance in stool | -0.000421476 | 0.0688689807388071 | 0.995315411624034 |
| GCST90032597 | T4eVfC | outcome | UBA11963 abundance in stool | -0.011428808 | 0.0397656928000272 | 0.788077872419603 |
| GCST90032598 | T4eVfC | outcome | UBA1206 sp000433115 abundance in stool | -0.0229003 | 0.0479253108520791 | 0.64555232952275 |
| GCST90032599 | T4eVfC | outcome | UBA1375 sp002305795 abundance in stool | 0.0326534200162952 | 0.0214053165911601 | 0.15812287637964 |
| GCST90032600 | T4eVfC | outcome | UBA1407 abundance in stool | -0.016679481 | 0.0688295476935787 | 0.831108198291024 |
| GCST90032601 | T4eVfC | outcome | UBA1409 abundance in stool | -0.045773595 | 0.0970058517118963 | 0.669222093604641 |
| GCST90032602 | T4eVfC | outcome | UBA1417 sp003531055 abundance in stool | 0.00129974693279112 | 0.0641259360752191 | 0.984486276737928 |
| GCST90032603 | T4eVfC | outcome | UBA1446 sp002329245 abundance in stool | -0.01034794 | 0.0472937041545131 | 0.833047163337884 |
| GCST90032604 | T4eVfC | outcome | UBA1448 sp002329405 abundance in stool | -0.004147959 | 0.0344052228453067 | 0.90797399756485 |
| GCST90032605 | T4eVfC | outcome | UBA1448 abundance in stool | 0.0254044007796589 | 0.0373326788043788 | 0.515403484546235 |
| GCST90032606 | T4eVfC | outcome | UBA1611 abundance in stool | -0.063136102 | 0.0329019966300648 | 0.0964838301986593 |
| GCST90032607 | T4eVfC | outcome | UBA1777 sp002320035 abundance in stool | 0.0990607942201486 | 1.15899753042486 | 0.945719330062833 |
| GCST90032608 | T4eVfC | outcome | UBA1777 sp900316255 abundance in stool | -0.027807493 | 0.0381032100361717 | 0.493011970849674 |
| GCST90032609 | T4eVfC | outcome | UBA1777 sp900319275 abundance in stool | 0.00105444123301147 | 0.0619980041981305 | 0.987498381339323 |
| GCST90032610 | T4eVfC | outcome | UBA1777 sp900319835 abundance in stool | -0.070241687 | 0.0376120439034235 | 0.0946703844126431 |
| GCST90032611 | T4eVfC | outcome | UBA2658 sp002841545 abundance in stool | -0.001112825 | 0.0394454228951172 | 0.978844685187903 |
| GCST90032612 | T4eVfC | outcome | UBA2821 abundance in stool | 0.0319530953525495 | 0.0393239583502233 | 0.427086852929719 |
| GCST90032613 | T4eVfC | outcome | UBA2922 sp900313925 abundance in stool | 0.0585402839921685 | 0.0476093739979293 | 0.250022642764953 |
| GCST90032614 | T4eVfC | outcome | UBA3282 sp002493835 abundance in stool | -0.075258339 | 0.0554518576159375 | 0.232771228332544 |
| GCST90032615 | T4eVfC | outcome | UBA3792 abundance in stool | 0.0812463116680701 | 0.150232600211826 | 0.603370678171058 |
| GCST90032616 | T4eVfC | outcome | UBA3855 sp900316885 abundance in stool | 0.0286938649241039 | 0.0663102969498139 | 0.678236452472736 |
| GCST90032617 | T4eVfC | outcome | UBA5394 sp002409725 abundance in stool | -0.028085591 | 0.0353276296459892 | 0.447069053564926 |
| GCST90032618 | T4eVfC | outcome | UBA6382 abundance in stool | -0.087978494 | 0.0671187172401928 | 0.23129263090515 |
| GCST90032619 | T4eVfC | outcome | UBA6398 sp002451695 abundance in stool | 0.0407535167007103 | 0.0224863163573057 | 0.107500207976729 |
| GCST90032620 | T4eVfC | outcome | UBA6398 abundance in stool | 0.0940920457272165 | 0.056376863384642 | 0.133672807788791 |
| GCST90032621 | T4eVfC | outcome | UBA644 abundance in stool | -0.005423681 | 0.0821154485845965 | 0.949484263592504 |
| GCST90032622 | T4eVfC | outcome | UBA6960 abundance in stool | -0.002396108 | 0.0524215547528493 | 0.965312021746501 |
| GCST90032623 | T4eVfC | outcome | UBA7102 sp002315655 abundance in stool | -0.034488907 | 0.0420858533747624 | 0.433671741705958 |
| GCST90032624 | T4eVfC | outcome | UBA7177 sp002491225 abundance in stool | -0.00659852 | 0.0295942204959601 | 0.827647860617102 |
| GCST90032625 | T4eVfC | outcome | UBA7177 abundance in stool | 0.020890036751389 | 0.0244820099274331 | 0.407865794574081 |
| GCST90032626 | T4eVfC | outcome | UBA7182 sp002491115 abundance in stool | -0.002105736 | 0.0539707409452404 | 0.969645367300735 |
| GCST90032627 | T4eVfC | outcome | UBA7182 abundance in stool | -0.027060468 | 0.0550828940413202 | 0.636429985328177 |
| GCST90032628 | T4eVfC | outcome | UBA737 sp002451855 abundance in stool | 0.00811714971946912 | 0.0571766405770657 | 0.891753720575666 |
| GCST90032629 | T4eVfC | outcome | UBA737 abundance in stool | -0.060623192 | 0.0725497715342255 | 0.435380971650563 |
| GCST90032630 | T4eVfC | outcome | UBA7703 abundance in stool | 0.045497339572232 | 0.0296915362620056 | 0.159803587798875 |
| GCST90032631 | T4eVfC | outcome | UBA7748 sp900314535 abundance in stool | 0.00511269529829278 | 0.0238873722606202 | 0.834822130767362 |
| GCST90032632 | T4eVfC | outcome | UBA8517 abundance in stool | -0.000571941 | 0.027700932904487 | 0.984032934880541 |
| GCST90032633 | T4eVfC | outcome | UBA8621 abundance in stool | 0.0731218814500802 | 0.0815501940426603 | 0.410995346854475 |
| GCST90032634 | T4eVfC | outcome | UBA8904 abundance in stool | -0.00839614 | 0.0516782865469057 | 0.876269829218533 |
| GCST90032635 | T4eVfC | outcome | UBA9475 sp002161235 abundance in stool | 0.0282753453492897 | 0.0835988417486452 | 0.746713595536905 |
| GCST90032636 | T4eVfC | outcome | UBA9475 sp002161675 abundance in stool | 0.153324480491948 | 0.149363689990065 | 0.35172064128662 |
| GCST90032637 | T4eVfC | outcome | UBP9 abundance in stool | 0.00683667108572415 | 0.0323221782411522 | 0.835532479462376 |
| GCST90032638 | T4eVfC | outcome | UCG-010 sp003150215 abundance in stool | -0.039899681 | 0.0646147116880897 | 0.563938106158387 |
| GCST90032639 | T4eVfC | outcome | UNC496MF abundance in stool | -0.083536628 | 0.0717533785900037 | 0.277877656463232 |
| GCST90032640 | T4eVfC | outcome | V9D3004 abundance in stool | -0.025558466 | 0.0508936823697828 | 0.627587944946538 |
| GCST90032641 | T4eVfC | outcome | Veillonellaceae abundance in stool | 0.0610481970376606 | 0.0350522142331786 | 0.119746243292331 |
| GCST90032642 | T4eVfC | outcome | Veillonella rogosae abundance in stool | 0.0313312574726802 | 0.0363302270865824 | 0.410856436618894 |
| GCST90032643 | T4eVfC | outcome | Veillonella abundance in stool | NA | NA | NA |
| GCST90032644 | T4eVfC | outcome | Victivallis sp002998355 abundance in stool | -0.004854038 | 0.0809729871922537 | 0.954520584372531 |
| GCST90032172 | T4eVfC | outcome | Absiella dolichum abundance in stool | 0.00732292540523936 | 0.0411642692561307 | 0.865787992362142 |
| GCST90032173 | T4eVfC | outcome | Acetobacteraceae abundance in stool | 0.0102904120273057 | 0.0815369460225536 | 0.902683449900768 |
| GCST90032174 | T4eVfC | outcome | Acetobacterales abundance in stool | 0.00384943504488243 | 0.0736248768078758 | 0.959239470907159 |
| GCST90032175 | T4eVfC | outcome | Achromobacter abundance in stool | 0.026409225895713 | 0.0456582644202013 | 0.58806133839002 |
| GCST90032176 | T4eVfC | outcome | Acidaminococcus fermentans abundance in stool | 0.0346781664472938 | 0.0532530674388367 | 0.535704547804042 |
| GCST90032177 | T4eVfC | outcome | Acidaminococcus sp900315205 abundance in stool | -0.031926146 | 0.0308808400050528 | 0.328186302578062 |
| GCST90032178 | T4eVfC | outcome | Acidobacteriales abundance in stool | 0.00334991791036073 | 0.0774983305670857 | 0.966728807342562 |
| GCST90032179 | T4eVfC | outcome | Actinobacteria abundance in stool | -0.031593469 | 0.027425052660685 | 0.313490651955211 |
| GCST90032180 | T4eVfC | outcome | Actinobacteriota abundance in stool | -0.069729431 | 0.0449991348294522 | 0.155654376316038 |
| GCST90032181 | T4eVfC | outcome | Actinomycetales abundance in stool | 0.310328628487182 | 0.2633271679315 | 0.447955949183313 |
| GCST90032182 | T4eVfC | outcome | Agathobacter sp000434275 abundance in stool | 0.0233687326009874 | 0.0337945801053653 | 0.527295094915547 |
| GCST90032183 | T4eVfC | outcome | Akkermansia muciniphila B abundance in stool | 0.0186761281149423 | 0.0252263965707189 | 0.476116211751076 |
| GCST90032184 | T4eVfC | outcome | Aliivibrio abundance in stool | 0.0141011838279156 | 0.0792639795531127 | 0.863839066982697 |
| GCST90032185 | T4eVfC | outcome | Alistipes shahii abundance in stool | -0.0097476 | 0.0339318983356465 | 0.783569217397509 |
| GCST90032186 | T4eVfC | outcome | Alistipes abundance in stool | 0.010877632175041 | 0.0273511010349535 | 0.711159542073488 |
| GCST90032187 | T4eVfC | outcome | Alloprevotella abundance in stool | -0.014045463 | 0.0254042119328945 | 0.593810354087898 |
| GCST90032188 | T4eVfC | outcome | Alteromonadaceae abundance in stool | -0.019111383 | 0.0412063747547137 | 0.651093568728736 |
| GCST90032189 | T4eVfC | outcome | An181 abundance in stool | -0.004811582 | 0.0404301514507483 | 0.907413233610831 |
| GCST90032190 | T4eVfC | outcome | An7 abundance in stool | 0.0645992305453953 | 0.0516043079124869 | 0.257225206407109 |
| GCST90032191 | T4eVfC | outcome | Anaeromassilibacillus sp001305115 abundance in stool | 0.0173212628534488 | 0.0339919297851844 | 0.626020473807452 |
| GCST90032192 | T4eVfC | outcome | Aneurinibacillaceae abundance in stool | 0.111171796221014 | 0.0512030428194089 | 0.0617034886348959 |
| GCST90032193 | T4eVfC | outcome | Aneurinibacillales abundance in stool | 0.106066945837445 | 0.051183627580724 | 0.0719733202739233 |
| GCST90032194 | T4eVfC | outcome | AR31 abundance in stool | 0.0329294811051931 | 0.0771456074988763 | 0.687245114701947 |
| GCST90032195 | T4eVfC | outcome | Atopobiaceae abundance in stool | 0.0193092773266253 | 0.0548699156828872 | 0.732208568960964 |
| GCST90032196 | T4eVfC | outcome | Aureimonas abundance in stool | 0.205822710629247 | 0.103171872032239 | 0.0862593617364509 |
| GCST90032197 | T4eVfC | outcome | Azorhizobium abundance in stool | -0.003596944 | 0.0416109640061258 | 0.933535556067519 |
| GCST90032198 | T4eVfC | outcome | Bacillaceae A abundance in stool | -0.009731391 | 0.0785180418486327 | 0.907342557797389 |
| GCST90032199 | T4eVfC | outcome | Bacillales A abundance in stool | 0.0441617380250551 | 0.087582015698744 | 0.648800065888956 |
| GCST90032200 | T4eVfC | outcome | Bacilli A abundance in stool | -0.064736633 | 0.0738171475294033 | 0.409580324040479 |
| GCST90032201 | T4eVfC | outcome | Bacillus AY abundance in stool | 0.00764272784264039 | 0.0516361026162591 | 0.885996424373995 |
| GCST90032202 | T4eVfC | outcome | Bacillus C abundance in stool | -0.054732804 | 0.0359969649921228 | 0.159357779644404 |
| GCST90032203 | T4eVfC | outcome | Bacillus abundance in stool | 0.0258548914394509 | 0.0588478334515691 | 0.673650030475945 |
| GCST90032204 | T4eVfC | outcome | Bacillus U abundance in stool | 0.00165610156465483 | 0.034536392079682 | 0.962929719259705 |
| GCST90032205 | T4eVfC | outcome | Bacillus velezensis abundance in stool | -0.006669887 | 0.0353826005432221 | 0.855827428836375 |
| GCST90032206 | T4eVfC | outcome | Bacteroides A plebeius A abundance in stool | 0.057292679263832 | 0.0587422347349089 | 0.367066630848117 |
| GCST90032207 | T4eVfC | outcome | Bacteroides A plebeius abundance in stool | 0.00796745483565389 | 0.0370100753106987 | 0.833877741448074 |
| GCST90032208 | T4eVfC | outcome | Bacteroides A abundance in stool | -0.193313714 | 0.0995554025284092 | 0.147455989364463 |
| GCST90032209 | T4eVfC | outcome | Bacteroides clarus abundance in stool | -0.073737194 | 0.124699233091844 | 0.586104839432485 |
| GCST90032210 | T4eVfC | outcome | Bacteroides eggerthii abundance in stool | 0.0174420877600261 | 0.0500842026578503 | 0.736635237399609 |
| GCST90032211 | T4eVfC | outcome | Bacteroides faecis abundance in stool | -0.026095991 | 0.0514754757844163 | 0.6388534933649 |
| GCST90032212 | T4eVfC | outcome | Bacteroides intestinalis A abundance in stool | -0.056416641 | 0.0473316777652729 | 0.278285405089833 |
| GCST90032213 | T4eVfC | outcome | Bacteroides sp002160055 abundance in stool | -0.060073499 | 0.0514213947971537 | 0.269799701275669 |
| GCST90032214 | T4eVfC | outcome | Bacteroides sp003545565 abundance in stool | 0.030672763746944 | 0.0382447668033322 | 0.448911832155813 |
| GCST90032215 | T4eVfC | outcome | Bacteroides stercoris abundance in stool | -0.042876624 | 0.0662496877521832 | 0.563612800037969 |
| GCST90032216 | T4eVfC | outcome | Bacteroides thetaiotaomicron abundance in stool | 0.0403833964027414 | 0.100022166360317 | 0.707053501481771 |
| GCST90032217 | T4eVfC | outcome | Barnesiellaceae abundance in stool | -0.035534069 | 0.035790625041241 | 0.35912823389972 |
| GCST90032218 | T4eVfC | outcome | Barnesiella abundance in stool | -0.019346342 | 0.0356635037155102 | 0.607040653295313 |
| GCST90032219 | T4eVfC | outcome | Bifidobacteriaceae abundance in stool | 0.00550675436355398 | 0.0813852472969875 | 0.950311253241923 |
| GCST90032220 | T4eVfC | outcome | Bifidobacterium adolescentis abundance in stool | 0.0674464202925552 | 0.0332598742158288 | 0.0771126220021805 |
| GCST90032221 | T4eVfC | outcome | Bifidobacterium angulatum abundance in stool | 0.0117709334114852 | 0.0253363653790263 | 0.653261713763543 |
| GCST90032222 | T4eVfC | outcome | Bifidobacterium bifidum abundance in stool | 0.0471277230474387 | 0.0582052886116929 | 0.449034121326991 |
| GCST90032223 | T4eVfC | outcome | Bifidobacterium breve abundance in stool | -0.004172574 | 0.0531621735848438 | 0.939367830653294 |
| GCST90032224 | T4eVfC | outcome | Bifidobacterium catenulatum abundance in stool | 0.069727887368888 | 0.164720663561831 | 0.693837715117854 |
| GCST90032225 | T4eVfC | outcome | Bifidobacterium infantis abundance in stool | 0.110016828638795 | 0.0915198636706356 | 0.283142665422893 |
| GCST90032226 | T4eVfC | outcome | Bifidobacterium kashiwanohense abundance in stool | -0.018268684 | 0.0631224966159137 | 0.783879647075998 |
| GCST90032227 | T4eVfC | outcome | Bifidobacterium longum abundance in stool | -0.119654238 | 0.144132403312756 | 0.493759963824314 |
| GCST90032228 | T4eVfC | outcome | Bifidobacterium pseudocatenulatum abundance in stool | 0.00346993100734505 | 0.0237334880619753 | 0.889472079842579 |
| GCST90032229 | T4eVfC | outcome | Bifidobacterium ruminantium abundance in stool | -0.046209015 | 0.0409464319942233 | 0.291802208615228 |
| GCST90032230 | T4eVfC | outcome | Bifidobacterium abundance in stool | 0.0061782301640148 | 0.0738349826005518 | 0.93733423135747 |
| GCST90032231 | T4eVfC | outcome | Bin127 abundance in stool | -0.028913792 | 0.100347523997892 | 0.780566239596737 |
| GCST90032232 | T4eVfC | outcome | Blautia A sp000285855 abundance in stool | 0.0109943104789687 | 0.0361241024274898 | 0.773122183730411 |
| GCST90032233 | T4eVfC | outcome | Blautia A sp002159835 abundance in stool | 0.0385446540994843 | 0.0756549379424989 | 0.626080492794139 |
| GCST90032234 | T4eVfC | outcome | Blautia A sp900066145 abundance in stool | -0.069421214 | 0.0389120174863207 | 0.124674678132166 |
| GCST90032235 | T4eVfC | outcome | Blautia A sp900066355 abundance in stool | -0.02104393 | 0.0626902532760003 | 0.753979697823345 |
| GCST90032236 | T4eVfC | outcome | Blautia hansenii abundance in stool | -0.044083838 | 0.0334690399532627 | 0.217169627914556 |
| GCST90032237 | T4eVfC | outcome | Blautia sp000436935 abundance in stool | -0.019245802 | 0.0238477492237612 | 0.438423474934768 |
| GCST90032238 | T4eVfC | outcome | Blautia sp001304935 abundance in stool | -0.007549877 | 0.0597070740762153 | 0.904304347246383 |
| GCST90032239 | T4eVfC | outcome | Borreliaceae abundance in stool | 0.0840463818663899 | 0.0347653956122892 | 0.052037745220409 |
| GCST90032240 | T4eVfC | outcome | Borreliales abundance in stool | 0.0552065297555768 | 0.0315882550296755 | 0.12400994358013 |
| GCST90032241 | T4eVfC | outcome | Brachyspiraceae abundance in stool | -0.013554772 | 0.0258706376053982 | 0.610711348687896 |
| GCST90032242 | T4eVfC | outcome | Brachyspirae abundance in stool | -0.00498694 | 0.0261145994418279 | 0.852375391165187 |
| GCST90032243 | T4eVfC | outcome | Brachyspirales abundance in stool | -0.006575777 | 0.0248900545184984 | 0.796515556564915 |
| GCST90032244 | T4eVfC | outcome | Brachyspira abundance in stool | 0.00354868137675269 | 0.0266490844626201 | 0.896705782694645 |
| GCST90032245 | T4eVfC | outcome | Brevibacillaceae abundance in stool | 0.0398868448610374 | 0.0437299899448968 | 0.396874482052629 |
| GCST90032246 | T4eVfC | outcome | Brevibacillales abundance in stool | -0.015334615 | 0.0347618784548264 | 0.672420289774956 |
| GCST90032247 | T4eVfC | outcome | Brevibacillus B abundance in stool | -0.014095161 | 0.0406830038956646 | 0.736174148582682 |
| GCST90032248 | T4eVfC | outcome | Butyricimonas sp900258545 abundance in stool | 0.0581709369875653 | 0.0544702192858616 | 0.316710670771315 |
| GCST90032249 | T4eVfC | outcome | CAG-1000 sp000434555 abundance in stool | 0.0387201180108291 | 0.0795617605938433 | 0.647075984771268 |
| GCST90032250 | T4eVfC | outcome | CAG-1000 abundance in stool | 0.0657182682937696 | 0.058271733854723 | 0.302478139222318 |
| GCST90032251 | T4eVfC | outcome | CAG-1031 abundance in stool | 0.026743809675243 | 0.0294194662219369 | 0.379867844168004 |
| GCST90032252 | T4eVfC | outcome | CAG-110 abundance in stool | -0.02482187 | 0.0323672526598688 | 0.465178429768566 |
| GCST90032253 | T4eVfC | outcome | CAG-145 sp000435615 abundance in stool | 0.0205192081439578 | 0.060161261341763 | 0.743059216862197 |
| GCST90032254 | T4eVfC | outcome | CAG-145 sp002320005 abundance in stool | 0.00465445791723368 | 0.0698346877991109 | 0.950058965002557 |
| GCST90032255 | T4eVfC | outcome | CAG-145 abundance in stool | -0.038169535 | 0.0532176364969733 | 0.525030321031894 |
| GCST90032256 | T4eVfC | outcome | CAG-170 sp003516765 abundance in stool | 0.0009430765658447 | 0.0678831693449061 | 0.989143936999493 |
| GCST90032257 | T4eVfC | outcome | CAG-177 sp002438685 abundance in stool | -0.017196525 | 0.0289970619810214 | 0.57180609414288 |
| GCST90032258 | T4eVfC | outcome | CAG-177 sp002451755 abundance in stool | -0.087417316 | 0.0755205371925392 | 0.285021977806836 |
| GCST90032259 | T4eVfC | outcome | CAG-177 sp003514385 abundance in stool | -0.047272149 | 0.028163118007821 | 0.144254035555027 |
| GCST90032260 | T4eVfC | outcome | CAG-177 sp003538135 abundance in stool | -0.008918703 | 0.0227315583241461 | 0.701157459450358 |
| GCST90032261 | T4eVfC | outcome | CAG-177 abundance in stool | -0.009363066 | 0.0291717647126446 | 0.759123477885744 |
| GCST90032262 | T4eVfC | outcome | CAG-180 sp000432435 abundance in stool | -0.104287371 | 0.0967003544256156 | 0.306155131557439 |
| GCST90032263 | T4eVfC | outcome | CAG-194 sp002441865 abundance in stool | 0.0180431234158389 | 0.0346710836847411 | 0.615337528552016 |
| GCST90032264 | T4eVfC | outcome | CAG-245 sp000435175 abundance in stool | -0.037421532 | 0.0477209110527832 | 0.453081355921818 |
| GCST90032265 | T4eVfC | outcome | CAG-245 abundance in stool | -0.013080084 | 0.0582578732127461 | 0.831239068266259 |
| GCST90032266 | T4eVfC | outcome | CAG-269 sp001915995 abundance in stool | -0.028869991 | 0.0330746366305664 | 0.408166272007674 |
| GCST90032267 | T4eVfC | outcome | CAG-269 sp001916065 abundance in stool | -0.018260246 | 0.0735833142866687 | 0.813882233122801 |
| GCST90032268 | T4eVfC | outcome | CAG-269 sp002372935 abundance in stool | 0.0120383578312965 | 0.0649419946759994 | 0.856311910468524 |
| GCST90032269 | T4eVfC | outcome | CAG-273 sp003507395 abundance in stool | 0.0148218265165752 | 0.0413952366133696 | 0.72652045885872 |
| GCST90032270 | T4eVfC | outcome | CAG-273 sp003534295 abundance in stool | 0.0127454898345627 | 0.0529689908567959 | 0.815240023131287 |
| GCST90032271 | T4eVfC | outcome | CAG-274 sp000432155 abundance in stool | 0.00408841875885943 | 0.031934051786414 | 0.901287498502647 |
| GCST90032272 | T4eVfC | outcome | CAG-302 abundance in stool | 0.121756325886978 | 0.0796889919996414 | 0.160887926192962 |
| GCST90032273 | T4eVfC | outcome | CAG-345 sp000433315 abundance in stool | -0.026209696 | 0.0314327019519928 | 0.422111635011236 |
| GCST90032274 | T4eVfC | outcome | CAG-345 abundance in stool | -0.031215471 | 0.0323834013240582 | 0.363307953778186 |
| GCST90032275 | T4eVfC | outcome | CAG-349 abundance in stool | -0.048535586 | 0.0572127861939275 | 0.418242120352475 |
| GCST90032276 | T4eVfC | outcome | CAG-390 sp003523225 abundance in stool | -0.009966911 | 0.023907719481737 | 0.685566085308161 |
| GCST90032277 | T4eVfC | outcome | CAG-433 abundance in stool | -0.017237112 | 0.0438953160033079 | 0.702791067439635 |
| GCST90032278 | T4eVfC | outcome | CAG-448 sp000433415 abundance in stool | -0.095946956 | 0.135794944693144 | 0.499899259896158 |
| GCST90032279 | T4eVfC | outcome | CAG-448 sp003150135 abundance in stool | -0.013526695 | 0.0382652658198506 | 0.731854248483552 |
| GCST90032280 | T4eVfC | outcome | CAG-448 abundance in stool | 0.00643772175793675 | 0.0362634138338921 | 0.864936358965912 |
| GCST90032281 | T4eVfC | outcome | CAG-449 abundance in stool | 0.0484568104183151 | 0.0326856645694162 | 0.188721942071469 |
| GCST90032282 | T4eVfC | outcome | CAG-452 sp000434035 abundance in stool | -0.054183895 | 0.0596639208698272 | 0.387469002755502 |
| GCST90032283 | T4eVfC | outcome | CAG-452 abundance in stool | -0.065457897 | 0.0591325298345867 | 0.31869925329745 |
| GCST90032284 | T4eVfC | outcome | CAG-465 sp000433135 abundance in stool | -0.040681085 | 0.032926297380025 | 0.244875439304886 |
| GCST90032285 | T4eVfC | outcome | CAG-475 abundance in stool | -0.014981295 | 0.0271341653445455 | 0.592990800117713 |
| GCST90032286 | T4eVfC | outcome | CAG-485 sp002362485 abundance in stool | 0.103404470661112 | 0.0534044682855158 | 0.124914334108969 |
| GCST90032287 | T4eVfC | outcome | CAG-485 sp002404675 abundance in stool | -0.043977331 | 0.0473694731330642 | 0.38036399893241 |
| GCST90032288 | T4eVfC | outcome | CAG-488 sp000434055 abundance in stool | -0.012982375 | 0.02514550708743 | 0.621556048905897 |
| GCST90032289 | T4eVfC | outcome | CAG-488 abundance in stool | -0.008377668 | 0.0181134694218705 | 0.650353453699224 |
| GCST90032290 | T4eVfC | outcome | CAG-495 abundance in stool | -0.092419583 | 0.0887743836530246 | 0.345545361977694 |
| GCST90032291 | T4eVfC | outcome | CAG-510 sp002432425 abundance in stool | -0.050839867 | 0.0469069593232226 | 0.31001446689723 |
| GCST90032292 | T4eVfC | outcome | CAG-552 abundance in stool | -0.038247828 | 0.0529711085189427 | 0.488598011280823 |
| GCST90032293 | T4eVfC | outcome | CAG-590 sp000431135 abundance in stool | -0.026328347 | 0.0203757056244165 | 0.213596933748337 |
| GCST90032294 | T4eVfC | outcome | CAG-632 abundance in stool | -0.048252118 | 0.0378052562167066 | 0.233787634023433 |
| GCST90032295 | T4eVfC | outcome | CAG-698 abundance in stool | -0.020367562 | 0.0320979301588231 | 0.545896933369959 |
| GCST90032296 | T4eVfC | outcome | CAG-776 sp000438195 abundance in stool | 0.00590064980754494 | 0.0384381948075217 | 0.882327765262571 |
| GCST90032297 | T4eVfC | outcome | CAG-776 abundance in stool | 0.0368160078381522 | 0.0523348331359545 | 0.504481608431213 |
| GCST90032298 | T4eVfC | outcome | CAG-81 sp000435795 abundance in stool | 0.0135877539722271 | 0.0498021528097592 | 0.792853823580219 |
| GCST90032299 | T4eVfC | outcome | CAG-822 sp000432855 abundance in stool | 0.0245218064024151 | 0.0268217437561401 | 0.376062496871732 |
| GCST90032300 | T4eVfC | outcome | CAG-822 abundance in stool | 0.0339796184409229 | 0.0305763387835995 | 0.292441716183029 |
| GCST90032301 | T4eVfC | outcome | CAG-826 abundance in stool | -0.020727644 | 0.0619542950854158 | 0.749340031078338 |
| GCST90032302 | T4eVfC | outcome | CAG-83 sp000435555 abundance in stool | -0.094776578 | 0.108748357565266 | 0.447605485309811 |
| GCST90032303 | T4eVfC | outcome | CAG-83 sp002392625 abundance in stool | -0.136377712 | 0.123700554049319 | 0.296077385843334 |
| GCST90032304 | T4eVfC | outcome | CAG-841 sp002479075 abundance in stool | -0.015731986 | 0.0316423008692542 | 0.636751296394953 |
| GCST90032305 | T4eVfC | outcome | CAG-873 sp001701165 abundance in stool | 0.00601275156417524 | 0.0381035535013742 | 0.87671883246576 |
| GCST90032306 | T4eVfC | outcome | CAG-877 sp000433455 abundance in stool | -0.041112877 | 0.037436905420949 | 0.29366752251069 |
| GCST90032307 | T4eVfC | outcome | CAG-877 abundance in stool | -0.053800003 | 0.0332505693252074 | 0.129653857504533 |
| GCST90032308 | T4eVfC | outcome | CAG-882 sp003486385 abundance in stool | -0.018341844 | 0.0450303427865727 | 0.697905315455207 |
| GCST90032309 | T4eVfC | outcome | CAG-884 sp000433875 abundance in stool | -0.085857299 | 0.0364183557572928 | 0.0427742582948725 |
| GCST90032310 | T4eVfC | outcome | CAG-884 abundance in stool | -0.05499482 | 0.0403905345774149 | 0.222238751256493 |
| GCST90032311 | T4eVfC | outcome | CAG-977 abundance in stool | -0.019042765 | 0.0213139511564404 | 0.392614893195736 |
| GCST90032312 | T4eVfC | outcome | Caloranaerobacteraceae abundance in stool | 0.0115677619555284 | 0.0434529681274372 | 0.793699398308985 |
| GCST90032313 | T4eVfC | outcome | Caloranaerobacter abundance in stool | 0.0167789579844135 | 0.0391818352062964 | 0.673857715068471 |
| GCST90032314 | T4eVfC | outcome | Campylobacter D abundance in stool | -0.031355695 | 0.0586540986732809 | 0.609487708249696 |
| GCST90032315 | T4eVfC | outcome | Cetobacterium A abundance in stool | -0.040485501 | 0.0503753955605427 | 0.432678741295415 |
| GCST90032316 | T4eVfC | outcome | CHKCI006 sp900018345 abundance in stool | 0.0309871764785848 | 0.0378753769783396 | 0.429226922631901 |
| GCST90032317 | T4eVfC | outcome | Chloroflexales abundance in stool | 0.00205359997759036 | 0.0315512905150739 | 0.949386991878882 |
| GCST90032318 | T4eVfC | outcome | Chromatiales abundance in stool | 0.0424082976273978 | 0.0483414026835208 | 0.403151422830276 |
| GCST90032319 | T4eVfC | outcome | Chromobacteriaceae abundance in stool | -0.112409128 | 0.0486526821560079 | 0.0462031766592132 |
| GCST90032320 | T4eVfC | outcome | Citrobacter A abundance in stool | 0.0249086227776367 | 0.0364710786787191 | 0.520111423964202 |
| GCST90032321 | T4eVfC | outcome | Clostridia abundance in stool | -0.103559841 | 0.108755410974444 | 0.384710838956612 |
| GCST90032322 | T4eVfC | outcome | Clostridium E sporosphaeroides abundance in stool | -0.037395207 | 0.0224554151247792 | 0.130205640812339 |
| GCST90032323 | T4eVfC | outcome | Clostridium I abundance in stool | 0.0075719321441738 | 0.0537614618634017 | 0.892603436503056 |
| GCST90032324 | T4eVfC | outcome | Clostridium M clostridioforme abundance in stool | 0.0204534516120702 | 0.0248012223229316 | 0.430858715147611 |
| GCST90032325 | T4eVfC | outcome | Clostridium M sp001304855 abundance in stool | -0.043391866 | 0.0876896109492025 | 0.654688417196034 |
| GCST90032326 | T4eVfC | outcome | Clostridium P abundance in stool | -0.013982728 | 0.0438175530133599 | 0.756207223391336 |
| GCST90032327 | T4eVfC | outcome | Clostridium saudiense abundance in stool | -0.042431697 | 0.0328604763082663 | 0.219099403885372 |
| GCST90032328 | T4eVfC | outcome | Clostridium S felsineum abundance in stool | 0.00147112663503824 | 0.0339214356007207 | 0.968132802584564 |
| GCST90032329 | T4eVfC | outcome | Clostridium tertium abundance in stool | -0.01659907 | 0.0766239783189267 | 0.833918684001617 |
| GCST90032330 | T4eVfC | outcome | Collinsella abundance in stool | -0.025870715 | 0.0443170486583139 | 0.572310071542163 |
| GCST90032331 | T4eVfC | outcome | Comamonas B abundance in stool | 0.0149425923855491 | 0.0311994549181171 | 0.646582085506454 |
| GCST90032332 | T4eVfC | outcome | Comamonas abundance in stool | 0.00687988842399443 | 0.0470496224934777 | 0.886648536387513 |
| GCST90032333 | T4eVfC | outcome | Coprobacillus cateniformis abundance in stool | -0.000800712 | 0.0559618877180994 | 0.988896281687753 |
| GCST90032334 | T4eVfC | outcome | Coprobacillus abundance in stool | 0.022832828202334 | 0.0675071090137841 | 0.745108726709288 |
| GCST90032335 | T4eVfC | outcome | Coprobacter secundus abundance in stool | -0.026258073 | 0.0832437737186456 | 0.768202361635762 |
| GCST90032336 | T4eVfC | outcome | Corynebacterium abundance in stool | 0.00445033536569328 | 0.0297214402463679 | 0.883683945482633 |
| GCST90032337 | T4eVfC | outcome | Cyanobacteria abundance in stool | -0.018976019 | 0.0235201968315429 | 0.44310595312321 |
| GCST90032338 | T4eVfC | outcome | Demequinaceae abundance in stool | 0.040646954867166 | 0.0394600202548382 | 0.333106342951288 |
| GCST90032339 | T4eVfC | outcome | Demequina abundance in stool | 0.0387419086826302 | 0.0244484654272841 | 0.141355989173799 |
| GCST90032340 | T4eVfC | outcome | Desulfobacterota A abundance in stool | -0.033863214 | 0.0331392808146552 | 0.328801993320244 |
| GCST90032341 | T4eVfC | outcome | Desulfovibrionaceae abundance in stool | -0.027455097 | 0.0299993701779929 | 0.379717544149622 |
| GCST90032342 | T4eVfC | outcome | Desulfovibrionales abundance in stool | -0.022124826 | 0.031589148158311 | 0.497037283352624 |
| GCST90032343 | T4eVfC | outcome | Desulfovibrionia abundance in stool | -0.035005346 | 0.0339537671188747 | 0.324690254245685 |
| GCST90032344 | T4eVfC | outcome | Desulfovibrio piger abundance in stool | -0.027311366 | 0.0265994195505125 | 0.331334643155027 |
| GCST90032345 | T4eVfC | outcome | Dialister sp000434475 abundance in stool | -0.017329275 | 0.0336530249959359 | 0.614628856366131 |
| GCST90032346 | T4eVfC | outcome | Dokdonella abundance in stool | 0.0400424553796525 | 0.0600284466353376 | 0.516400975369503 |
| GCST90032347 | T4eVfC | outcome | Dorea phocaeense abundance in stool | 0.0188927364562612 | 0.037661515694265 | 0.625805033794354 |
| GCST90032348 | T4eVfC | outcome | Dorea abundance in stool | -0.06907289 | 0.070735511844914 | 0.3665310480026 |
| GCST90032349 | T4eVfC | outcome | DTU024 sp002411105 abundance in stool | -0.003569026 | 0.0371456998525215 | 0.925561060525264 |
| GCST90032350 | T4eVfC | outcome | Dysgonomonadaceae abundance in stool | 0.0140675720125466 | 0.0474601061855555 | 0.775519377904974 |
| GCST90032351 | T4eVfC | outcome | Eisenbergiella sp900066775 abundance in stool | -0.027653213 | 0.0296094523513032 | 0.374716696179724 |
| GCST90032352 | T4eVfC | outcome | Elusimicrobiaceae abundance in stool | 0.0480143350095799 | 0.0218693302644809 | 0.0454813545579685 |
| GCST90032353 | T4eVfC | outcome | Elusimicrobia abundance in stool | -0.026715755 | 0.0253128878518338 | 0.305193691894507 |
| GCST90032354 | T4eVfC | outcome | Elusimicrobiota abundance in stool | -0.035523691 | 0.0236587408713257 | 0.146268672218375 |
| GCST90032355 | T4eVfC | outcome | Emergencia abundance in stool | -0.039772732 | 0.0767789896421509 | 0.620412285907612 |
| GCST90032356 | T4eVfC | outcome | Endozoicomonadaceae abundance in stool | 0.00392507154280258 | 0.0341642212880724 | 0.911759703337746 |
| GCST90032357 | T4eVfC | outcome | Enorma massiliensis abundance in stool | 0.0475055833254354 | 0.0454210355464764 | 0.322885967168805 |
| GCST90032358 | T4eVfC | outcome | Ensifer abundance in stool | -0.013716296 | 0.0759188355797885 | 0.861745713781892 |
| GCST90032359 | T4eVfC | outcome | Enterobacteriaceae abundance in stool | 0.0580610795159022 | 0.0528384327701249 | 0.321910064689769 |
| GCST90032360 | T4eVfC | outcome | Enterococcaceae abundance in stool | -0.012000026 | 0.0353342530063321 | 0.739187886154999 |
| GCST90032361 | T4eVfC | outcome | Enterococcus A abundance in stool | 0.0803940519146894 | 0.0275827971940922 | 0.0140716324595946 |
| GCST90032362 | T4eVfC | outcome | Enterococcus B abundance in stool | 0.00914422006353256 | 0.0515151685692338 | 0.862655406008144 |
| GCST90032363 | T4eVfC | outcome | Enterococcus faecalis abundance in stool | 0.0316926694578206 | 0.0330357150500114 | 0.360005139146968 |
| GCST90032364 | T4eVfC | outcome | Enterococcus abundance in stool | 0.042594307535098 | 0.04964758834187 | 0.423869711078516 |
| GCST90032365 | T4eVfC | outcome | Enteroscipio abundance in stool | -0.029569766 | 0.0359205739727372 | 0.426452315180885 |
| GCST90032366 | T4eVfC | outcome | ER4 sp002437735 abundance in stool | -0.021733919 | 0.0372950326212131 | 0.576110419285596 |
| GCST90032367 | T4eVfC | outcome | Eremiobacterota abundance in stool | 0.000657187979406644 | 0.0541391303123667 | 0.990579644785161 |
| GCST90032368 | T4eVfC | outcome | Erysipelatoclostridiaceae abundance in stool | 0.0310282064324443 | 0.0448851742072087 | 0.511656716623053 |
| GCST90032369 | T4eVfC | outcome | Escherichia flexneri abundance in stool | 0.02683676513706 | 0.0328326168614048 | 0.440639941498832 |
| GCST90032370 | T4eVfC | outcome | Escherichia abundance in stool | 0.016934606966555 | 0.0710801012865618 | 0.821145645970421 |
| GCST90032371 | T4eVfC | outcome | Eubacterium callanderi abundance in stool | 0.00849535490609574 | 0.0257008296119011 | 0.749476634368335 |
| GCST90032372 | T4eVfC | outcome | Eubacterium F sp000434115 abundance in stool | 0.0575597671396781 | 0.0532339686873257 | 0.307701270771929 |
| GCST90032373 | T4eVfC | outcome | Eubacterium I ramulus A abundance in stool | -0.013243588 | 0.0282224040638073 | 0.646658057013386 |
| GCST90032374 | T4eVfC | outcome | Eubacterium Q abundance in stool | -0.059643216 | 0.0310606660815323 | 0.0910850660592237 |
| GCST90032375 | T4eVfC | outcome | Eubacterium R coprostanoligenes abundance in stool | 0.0684471942085474 | 0.0729443141278856 | 0.401191627275663 |
| GCST90032376 | T4eVfC | outcome | Eubacterium R sp000431535 abundance in stool | 0.0483772784329438 | 0.0446766915327675 | 0.30704011594243 |
| GCST90032377 | T4eVfC | outcome | Ezakiellaceae abundance in stool | -0.016193006 | 0.0401832842728824 | 0.70757336279747 |
| GCST90032378 | T4eVfC | outcome | F0428 abundance in stool | -0.041720214 | 0.0289039696831681 | 0.179491424441784 |
| GCST90032379 | T4eVfC | outcome | Faecalibacterium prausnitzii E abundance in stool | 0.0729684960861718 | 0.062542371102671 | 0.281533723405407 |
| GCST90032380 | T4eVfC | outcome | Faecalibacterium sp002160895 abundance in stool | -0.017306517 | 0.0353031580759019 | 0.635703065124432 |
| GCST90032381 | T4eVfC | outcome | Faecalicatena glycyrrhizinilyticum abundance in stool | 0.0183445828458203 | 0.0315379649813244 | 0.570740386650488 |
| GCST90032382 | T4eVfC | outcome | Faecalicatena lactaris abundance in stool | 0.00901627454963346 | 0.0532450233886382 | 0.869277653703078 |
| GCST90032383 | T4eVfC | outcome | Faecalicatena sp000364245 abundance in stool | 0.00869524752285599 | 0.0625876904268603 | 0.89405200049151 |
| GCST90032384 | T4eVfC | outcome | Faecalicatena sp001517425 abundance in stool | -0.028316328 | 0.0393624240851667 | 0.485684509688479 |
| GCST90032385 | T4eVfC | outcome | Faecalicatena sp002161355 abundance in stool | -0.120914738 | 0.0747504096679517 | 0.166676901374144 |
| GCST90032386 | T4eVfC | outcome | Faecalicatena sp002397985 abundance in stool | 0.0104993597585158 | 0.0304237753483527 | 0.741792627491215 |
| GCST90032387 | T4eVfC | outcome | Faecalicatena torques abundance in stool | 0.0514282392261899 | 0.027716312319698 | 0.0931972464641512 |
| GCST90032388 | T4eVfC | outcome | Faecalicoccus pleomorphus abundance in stool | -0.011975404 | 0.0283402181642709 | 0.690173492268261 |
| GCST90032389 | T4eVfC | outcome | Faecalicoccus abundance in stool | -0.016150893 | 0.0383320720443303 | 0.691005476044796 |
| GCST90032390 | T4eVfC | outcome | Faecalitalea cylindroides abundance in stool | -0.164637664 | 0.0929252572814056 | 0.151135881918364 |
| GCST90032391 | T4eVfC | outcome | Fervidobacteriaceae abundance in stool | -0.105866746 | 0.0433666870013545 | 0.0372911758944558 |
| GCST90032392 | T4eVfC | outcome | Fibrobacteraceae abundance in stool | -0.011170862 | 0.029610150174688 | 0.715788350284572 |
| GCST90032393 | T4eVfC | outcome | Fibrobacterales abundance in stool | -0.023812146 | 0.0321627656370627 | 0.487028953310238 |
| GCST90032394 | T4eVfC | outcome | Fibrobacteria abundance in stool | -0.014751634 | 0.026629408896428 | 0.591776869806754 |
| GCST90032395 | T4eVfC | outcome | Fimbriimonadia abundance in stool | 0.000112781112311089 | 0.0329048157158831 | 0.997321580268211 |
| GCST90032396 | T4eVfC | outcome | Firmicutes A abundance in stool | -0.048130264 | 0.0672379286084109 | 0.492255404938825 |
| GCST90032397 | T4eVfC | outcome | Firmicutes E abundance in stool | -0.072510334 | 0.0522364423528131 | 0.207674742535366 |
| GCST90032398 | T4eVfC | outcome | Firmicutes I abundance in stool | -0.058088172 | 0.0477231336709929 | 0.251475900022936 |
| GCST90032399 | T4eVfC | outcome | Flavobacteriales abundance in stool | 0.00585341028816058 | 0.0213690629848373 | 0.78845025339472 |
| GCST90032400 | T4eVfC | outcome | Flavonifractor sp002159265 abundance in stool | -0.094272784 | 0.108110632010784 | 0.412114858848189 |
| GCST90032401 | T4eVfC | outcome | Flavonifractor sp900199495 abundance in stool | 0.0152926493948808 | 0.0451362913168357 | 0.741131816074288 |
| GCST90032402 | T4eVfC | outcome | Fournierella massiliensis abundance in stool | 0.0272054285652523 | 0.0993591079246935 | 0.791167990755204 |
| GCST90032403 | T4eVfC | outcome | Francisellaceae abundance in stool | 0.0154243421129445 | 0.0612254794875329 | 0.809504980310995 |
| GCST90032404 | T4eVfC | outcome | Francisellales abundance in stool | 0.0609700354594272 | 0.0749335828181636 | 0.452833222411281 |
| GCST90032405 | T4eVfC | outcome | Fusobacteriaceae abundance in stool | 0.00302485255262591 | 0.0302705483146617 | 0.922860861413239 |
| GCST90032406 | T4eVfC | outcome | Fusobacterium A abundance in stool | -0.018155947 | 0.0334917453719687 | 0.604567681271993 |
| GCST90032407 | T4eVfC | outcome | GCA-900066135 sp900066135 abundance in stool | 0.00313761042468682 | 0.0491404404942273 | 0.950485427418834 |
| GCST90032408 | T4eVfC | outcome | GCA-900066495 sp900066495 abundance in stool | 0.0305039341951622 | 0.0363429310184112 | 0.415383646364573 |
| GCST90032409 | T4eVfC | outcome | GCA-900066495 abundance in stool | 0.0312032503659069 | 0.030173678881395 | 0.321470814980199 |
| GCST90032410 | T4eVfC | outcome | GCA-900066575 sp900066385 abundance in stool | -0.001956737 | 0.0411368027980734 | 0.962998237632209 |
| GCST90032411 | T4eVfC | outcome | GCA-900066755 sp900066755 abundance in stool | 0.0144142074208033 | 0.307911356094823 | 0.965604379882923 |
| GCST90032412 | T4eVfC | outcome | GCA-900066755 abundance in stool | 0.0497807990135323 | 0.0665602185858763 | 0.473605465126951 |
| GCST90032413 | T4eVfC | outcome | GCA-900199385 sp900320755 abundance in stool | 0.0533905621859976 | 0.113843261830123 | 0.663501206298875 |
| GCST90032414 | T4eVfC | outcome | Geminocystis abundance in stool | -5.06E-05 | 0.0374960229163403 | 0.998967823840615 |
| GCST90032415 | T4eVfC | outcome | Gemmatimonadaceae abundance in stool | -0.032400065 | 0.0366134162219067 | 0.39358426715872 |
| GCST90032416 | T4eVfC | outcome | Geobacteraceae abundance in stool | -0.046963011 | 0.0414474791765492 | 0.320494724934062 |
| GCST90032417 | T4eVfC | outcome | Geobacter C abundance in stool | 0.0313421079882538 | 0.0510599163187322 | 0.566176835998519 |
| GCST90032418 | T4eVfC | outcome | Gillisia abundance in stool | 0.054712651319442 | 0.0750507183590896 | 0.482720525538889 |
| GCST90032419 | T4eVfC | outcome | Gluconobacter abundance in stool | 0.000292430556422389 | 0.0275163374983055 | 0.991752471344635 |
| GCST90032420 | T4eVfC | outcome | Gordonibacter pamelaeae abundance in stool | 0.00522696391927943 | 0.0364868444973441 | 0.890777944828183 |
| GCST90032421 | T4eVfC | outcome | Gordonibacter abundance in stool | -0.059265756 | 0.0386301781714017 | 0.155992936863124 |
| GCST90032422 | T4eVfC | outcome | Gramella abundance in stool | 0.0149665341315196 | 0.0393462113596223 | 0.710905682128423 |
| GCST90032423 | T4eVfC | outcome | Haemophilus D sp001679485 abundance in stool | 0.0439115680392698 | 0.0273283823074364 | 0.152130899249083 |
| GCST90032424 | T4eVfC | outcome | Halarcobacter abundance in stool | -0.010016435 | 0.0371119050693815 | 0.796281362197545 |
| GCST90032425 | T4eVfC | outcome | Halomonadaceae abundance in stool | 0.0172990053993291 | 0.0256148871401793 | 0.514765796848671 |
| GCST90032426 | T4eVfC | outcome | Haloplasmatales abundance in stool | 0.0287722751786424 | 0.0435039404622029 | 0.526959178925282 |
| GCST90032427 | T4eVfC | outcome | Helicobacter abundance in stool | 0.0362136717683038 | 0.061159301599253 | 0.5753878252719 |
| GCST90032428 | T4eVfC | outcome | Herbidospora abundance in stool | -0.014249142 | 0.0240224988979111 | 0.567679826143262 |
| GCST90032429 | T4eVfC | outcome | Herbinix abundance in stool | 0.036853710936575 | 0.0323067680235773 | 0.283422004748001 |
| GCST90032430 | T4eVfC | outcome | Holdemania massiliensis abundance in stool | -0.066026509 | 0.0818277327849563 | 0.446271316234047 |
| GCST90032431 | T4eVfC | outcome | Holdemania sp900120005 abundance in stool | -0.029086485 | 0.0304716996236757 | 0.364743315290446 |
| GCST90032432 | T4eVfC | outcome | Holdemania abundance in stool | 0.0540038967387472 | 0.0440425447427645 | 0.251246162877826 |
| GCST90032433 | T4eVfC | outcome | Hungatella sp900155545 abundance in stool | -0.0183256 | 0.0877621392506227 | 0.842836884916982 |
| GCST90032434 | T4eVfC | outcome | Hydrogenophaga abundance in stool | -0.00566907 | 0.040213378442443 | 0.89250385575769 |
| GCST90032435 | T4eVfC | outcome | Hyphomonas abundance in stool | 0.0228321081222219 | 0.0474800166063078 | 0.647635636085129 |
| GCST90032436 | T4eVfC | outcome | Intestinimonas massiliensis abundance in stool | -0.103240613 | 0.0524943028010337 | 0.0899273051879762 |
| GCST90032437 | T4eVfC | outcome | Jiangellaceae abundance in stool | -0.083726953 | 0.0683818945609803 | 0.25563041821493 |
| GCST90032438 | T4eVfC | outcome | Johnsonella ignava abundance in stool | 0.0592482978553863 | 0.23967975597263 | 0.827815408968701 |
| GCST90032439 | T4eVfC | outcome | K10 sp001941205 abundance in stool | 0.0341475574733092 | 0.042884625708029 | 0.456218331295972 |
| GCST90032440 | T4eVfC | outcome | K10 abundance in stool | -0.001410634 | 0.0830927883418317 | 0.989193394815816 |
| GCST90032441 | T4eVfC | outcome | Kandleria vitulina abundance in stool | 0.00437550799110568 | 0.0350151905572307 | 0.904067946014331 |
| GCST90032442 | T4eVfC | outcome | Kineothrix abundance in stool | 0.0125688660696174 | 0.0313364199425108 | 0.694865320348026 |
| GCST90032443 | T4eVfC | outcome | KLE1615 sp900066985 abundance in stool | -0.021023595 | 0.0431691033007244 | 0.6411332879133 |
| GCST90032444 | T4eVfC | outcome | KLE1615 abundance in stool | 0.0248958318146159 | 0.0376062713201437 | 0.529146656175462 |
| GCST90032445 | T4eVfC | outcome | Klebsiella A abundance in stool | 0.0150977771579672 | 0.0185073475043655 | 0.427395255067484 |
| GCST90032446 | T4eVfC | outcome | Klebsiella pneumoniae abundance in stool | 0.00721710476645897 | 0.0317654122390663 | 0.825966670120743 |
| GCST90032447 | T4eVfC | outcome | Klebsiella abundance in stool | -0.003949782 | 0.0251649266858502 | 0.878123441036492 |
| GCST90032448 | T4eVfC | outcome | koll11 abundance in stool | -0.055541102 | 0.0404259000285183 | 0.227867392457166 |
| GCST90032449 | T4eVfC | outcome | Lachnoanaerobaculum saburreum abundance in stool | 0.0145522984187192 | 0.0392763469302814 | 0.729788242034144 |
| GCST90032450 | T4eVfC | outcome | Lachnospiraceae abundance in stool | 0.0556170469070253 | 0.112838780448593 | 0.639607570186538 |
| GCST90032451 | T4eVfC | outcome | Lachnospirales abundance in stool | 0.243811851698988 | 0.130599215572566 | 0.120898622928599 |
| GCST90032452 | T4eVfC | outcome | Lachnospira rogosae abundance in stool | -0.063717709 | 0.0493331878124086 | 0.232566885226183 |
| GCST90032453 | T4eVfC | outcome | Lachnospira sp000437735 abundance in stool | 0.0712801363024127 | 0.0535798142293057 | 0.254180743772144 |
| GCST90032454 | T4eVfC | outcome | Lactobacillus B ruminis abundance in stool | -0.000164564 | 0.0360295797861456 | 0.99650377701486 |
| GCST90032455 | T4eVfC | outcome | Lactobacillus B salivarius abundance in stool | 0.0194940294887598 | 0.023371540511751 | 0.415161375944162 |
| GCST90032456 | T4eVfC | outcome | Lactobacillus B abundance in stool | -0.004727124 | 0.0184024879920654 | 0.802483722995237 |
| GCST90032457 | T4eVfC | outcome | Lactococcus lactis abundance in stool | 0.0188554492857638 | 0.0499937822816584 | 0.725214243582232 |
| GCST90032458 | T4eVfC | outcome | Lawsonibacter sp000492175 abundance in stool | 0.00293611371040035 | 0.0421171570322318 | 0.946133176712026 |
| GCST90032459 | T4eVfC | outcome | Lawsonibacter sp002161175 abundance in stool | 0.0925304831558509 | 0.0353243856413534 | 0.0344366943920254 |
| GCST90032460 | T4eVfC | outcome | Lawsonibacter sp900066645 abundance in stool | 0.00751060085999824 | 0.0424987552332943 | 0.862447792919462 |
| GCST90032461 | T4eVfC | outcome | Leclercia abundance in stool | -0.016338127 | 0.0294817466810154 | 0.59670846213014 |
| GCST90032462 | T4eVfC | outcome | Lentimicrobiaceae abundance in stool | -0.110373535 | 0.106490894757583 | 0.327032045027516 |
| GCST90032463 | T4eVfC | outcome | Leptospirae abundance in stool | 0.0239977637621397 | 0.0398802721903728 | 0.555775143603357 |
| GCST90032464 | T4eVfC | outcome | Leptospirales abundance in stool | -0.018680858 | 0.0446623602908883 | 0.681310887511232 |
| GCST90032465 | T4eVfC | outcome | Leuconostoc mesenteroides abundance in stool | -0.023402488 | 0.0485091353801718 | 0.654709321761239 |
| GCST90032466 | T4eVfC | outcome | Leuconostoc abundance in stool | -0.015986964 | 0.0304005517067494 | 0.610436204969514 |
| GCST90032467 | T4eVfC | outcome | Magnetospirillum A abundance in stool | -0.000869189 | 0.0837162432527606 | 0.992213251545716 |
| GCST90032468 | T4eVfC | outcome | Marinilabiliaceae abundance in stool | 0.0256250415080529 | 0.0518590649397883 | 0.638782440562356 |
| GCST90032469 | T4eVfC | outcome | Massiliomicrobiota sp002160815 abundance in stool | -0.006349186 | 0.0299906779498309 | 0.835619871266417 |
| GCST90032470 | T4eVfC | outcome | Massiliomicrobiota abundance in stool | -0.066227197 | 0.0730254454349876 | 0.390954776339334 |
| GCST90032471 | T4eVfC | outcome | Megamonas funiformis abundance in stool | -0.030941951 | 0.0259410279796134 | 0.252779427526342 |
| GCST90032472 | T4eVfC | outcome | Megamonas abundance in stool | -0.023326985 | 0.0206169378139802 | 0.274540766937945 |
| GCST90032473 | T4eVfC | outcome | Megasphaera elsdenii abundance in stool | -0.024275246 | 0.0229791180524649 | 0.313432586093303 |
| GCST90032474 | T4eVfC | outcome | Megasphaera sp900066485 abundance in stool | -0.045430759 | 0.0292897405610406 | 0.171863803434452 |
| GCST90032475 | T4eVfC | outcome | Megasphaera abundance in stool | -0.02065573 | 0.0206223689057617 | 0.331436567835335 |
| GCST90032476 | T4eVfC | outcome | Merdibacter massiliensis abundance in stool | -0.001771545 | 0.0869917818311443 | 0.984540273954572 |
| GCST90032477 | T4eVfC | outcome | Methanobacterium B abundance in stool | 0.0241381505246301 | 0.0491616642100204 | 0.649148963503393 |
| GCST90032478 | T4eVfC | outcome | Methanobrevibacter B abundance in stool | 0.069403128257624 | 0.0654008853564068 | 0.323836400961152 |
| GCST90032479 | T4eVfC | outcome | Microvirga abundance in stool | 0.133441749483567 | 0.137649619176817 | 0.403836739826665 |
| GCST90032480 | T4eVfC | outcome | Monoglobaceae abundance in stool | -0.031149498 | 0.0357584867242924 | 0.404123056772967 |
| GCST90032481 | T4eVfC | outcome | Monoglobus pectinilyticus abundance in stool | -0.020546439 | 0.036253746757792 | 0.581336898430649 |
| GCST90032482 | T4eVfC | outcome | Monoglobus abundance in stool | -0.015818288 | 0.0320475024415188 | 0.631303493138555 |
| GCST90032483 | T4eVfC | outcome | Morganella abundance in stool | 0.0641001749113175 | 0.0230995328493401 | 0.012970693933839 |
| GCST90032484 | T4eVfC | outcome | Mycobacteriaceae abundance in stool | 0.017440072065089 | 0.0465483938269591 | 0.716585218196475 |
| GCST90032485 | T4eVfC | outcome | Mycoplasmataceae abundance in stool | 0.113674633507289 | 0.0707525806783237 | 0.183406987688572 |
| GCST90032486 | T4eVfC | outcome | Mycoplasmatales abundance in stool | -0.005592717 | 0.0535543751429191 | 0.919398339371222 |
| GCST90032487 | T4eVfC | outcome | Mycoplasmoidaceae abundance in stool | -0.019416112 | 0.0241231905081662 | 0.435366610360743 |
| GCST90032488 | T4eVfC | outcome | Negativibacillus massiliensis abundance in stool | 0.0117568313649286 | 0.0341428185106435 | 0.738497701638386 |
| GCST90032489 | T4eVfC | outcome | Negativibacillus sp000435195 abundance in stool | 0.0398524813724054 | 0.0404307701965289 | 0.350037914087571 |
| GCST90032490 | T4eVfC | outcome | Negativibacillus abundance in stool | 0.0902330262817925 | 0.0434184596327068 | 0.0829390010731285 |
| GCST90032491 | T4eVfC | outcome | NK4A144 abundance in stool | -0.0063788 | 0.0263217165341576 | 0.814613999249096 |
| GCST90032492 | T4eVfC | outcome | Odoribacter laneus abundance in stool | -0.005538779 | 0.0346870151324939 | 0.877092073352775 |
| GCST90032493 | T4eVfC | outcome | Olsenella C abundance in stool | 0.0361959217998683 | 0.0794035806603073 | 0.672150218880934 |
| GCST90032494 | T4eVfC | outcome | Omnitrophota abundance in stool | 0.0410312368849688 | 0.0501919882297324 | 0.434757655281751 |
| GCST90032495 | T4eVfC | outcome | Paceibacteria abundance in stool | 0.0678399603701559 | 0.0399080331804411 | 0.127570750494918 |
| GCST90032496 | T4eVfC | outcome | Paenibacillales abundance in stool | -0.013545001 | 0.0423435500756023 | 0.765068113145093 |
| GCST90032497 | T4eVfC | outcome | Paenibacillus J abundance in stool | 0.00226886600500333 | 0.0253030128665756 | 0.930321699098249 |
| GCST90032498 | T4eVfC | outcome | Pandoraea abundance in stool | 0.0434848362157346 | 0.0676221218830298 | 0.536225293982237 |
| GCST90032499 | T4eVfC | outcome | Parabacteroides johnsonii abundance in stool | 0.00604483605845099 | 0.0419087203428814 | 0.88752447792693 |
| GCST90032500 | T4eVfC | outcome | Parabacteroides sp000436495 abundance in stool | 0.0092754691432551 | 0.0507883423757039 | 0.862262533038712 |
| GCST90032501 | T4eVfC | outcome | Parabacteroides abundance in stool | -0.051445921 | 0.0453938423872633 | 0.276113998561806 |
| GCST90032502 | T4eVfC | outcome | Parachlamydiales abundance in stool | -0.043386289 | 0.0386696491306572 | 0.312844846476735 |
| GCST90032503 | T4eVfC | outcome | Paraglaciecola abundance in stool | -0.006957898 | 0.0526325648641567 | 0.897450115451531 |
| GCST90032504 | T4eVfC | outcome | Paramuribaculum sp001689565 abundance in stool | -0.038027634 | 0.0639203340813329 | 0.573631284820485 |
| GCST90032505 | T4eVfC | outcome | Pararhizobium abundance in stool | 0.0572712380771857 | 0.045496991170459 | 0.236697799565609 |
| GCST90032506 | T4eVfC | outcome | Pauljensenia sp000411415 abundance in stool | 0.011855015639734 | 0.0326774962664143 | 0.726161015064012 |
| GCST90032507 | T4eVfC | outcome | Peptococcia abundance in stool | -0.037229808 | 0.029457650694168 | 0.246745665952812 |
| GCST90032508 | T4eVfC | outcome | Phascolarctobacterium sp003150755 abundance in stool | 0.0118442547007129 | 0.0259627291953405 | 0.659053564850195 |
| GCST90032509 | T4eVfC | outcome | Phocea massiliensis abundance in stool | 0.00898062681016752 | 0.0631649959913344 | 0.895951411231582 |
| GCST90032510 | T4eVfC | outcome | Phocea abundance in stool | -0.226952609 | 0.124422446041804 | 0.209705747872947 |
| GCST90032511 | T4eVfC | outcome | Photobacterium abundance in stool | -0.112258898 | 0.103704050305428 | 0.474795030741966 |
| GCST90032512 | T4eVfC | outcome | Planococcaceae abundance in stool | 0.000328630092550736 | 0.0808676337688953 | 0.997126469556171 |
| GCST90032513 | T4eVfC | outcome | Poseidoniaceae abundance in stool | -0.009220896 | 0.0651903664398569 | 0.891502504424385 |
| GCST90032514 | T4eVfC | outcome | Prevotella bivia abundance in stool | -0.022915266 | 0.0335937828378058 | 0.525468882396345 |
| GCST90032515 | T4eVfC | outcome | Prevotella buccae abundance in stool | -0.008705505 | 0.0825926111626356 | 0.920154007525123 |
| GCST90032516 | T4eVfC | outcome | Prevotellamassilia sp000437675 abundance in stool | 0.00467319943535781 | 0.0457963972040811 | 0.920959935552154 |
| GCST90032517 | T4eVfC | outcome | Prevotellamassilia abundance in stool | -0.079079597 | 0.0446949055364261 | 0.137067596432746 |
| GCST90032518 | T4eVfC | outcome | Prevotella sp000434975 abundance in stool | -0.013476314 | 0.0696383377745174 | 0.858914195040632 |
| GCST90032519 | T4eVfC | outcome | Prevotella sp000436915 abundance in stool | -0.051077633 | 0.0322162374871889 | 0.173717874025196 |
| GCST90032520 | T4eVfC | outcome | Prevotella sp002437285 abundance in stool | 0.0136943606375922 | 0.0355347466109488 | 0.713248558100255 |
| GCST90032521 | T4eVfC | outcome | Prevotella sp002437565 abundance in stool | -0.025030705 | 0.0317392921978216 | 0.446989250900351 |
| GCST90032522 | T4eVfC | outcome | Prevotella sp002933775 abundance in stool | 0.0438253973712174 | 0.040274350188528 | 0.318286343772328 |
| GCST90032523 | T4eVfC | outcome | Prevotella sp900317685 abundance in stool | 0.00965180539378321 | 0.0866528148318667 | 0.914943893327179 |
| GCST90032524 | T4eVfC | outcome | Prevotella sp900318625 abundance in stool | -0.295664636 | 0.178679591067067 | 0.346065670138831 |
| GCST90032525 | T4eVfC | outcome | Propionibacterium freudenreichii abundance in stool | -0.059620981 | 0.0568840954384476 | 0.329420837177792 |
| GCST90032526 | T4eVfC | outcome | Proteus abundance in stool | 0.00899295567482572 | 0.0337146843344862 | 0.794200043055377 |
| GCST90032527 | T4eVfC | outcome | Provencibacterium massiliense abundance in stool | -0.011776506 | 0.0514739289593836 | 0.823646303853176 |
| GCST90032528 | T4eVfC | outcome | Provencibacterium abundance in stool | -0.023353335 | 0.0508286951425046 | 0.65412186992149 |
| GCST90032529 | T4eVfC | outcome | Providencia abundance in stool | -0.022274043 | 0.0227874597056698 | 0.353877819465185 |
| GCST90032530 | T4eVfC | outcome | Pseudomonadales abundance in stool | -0.037892839 | 0.0356315968353418 | 0.322876744714371 |
| GCST90032531 | T4eVfC | outcome | Pseudomonas aeruginosa abundance in stool | 0.109267581780718 | 0.0696738344088575 | 0.167865080474859 |
| GCST90032532 | T4eVfC | outcome | Psychroserpens abundance in stool | -0.014502304 | 0.0305096191196668 | 0.64308307592528 |
| GCST90032533 | T4eVfC | outcome | QALR01 sp003150035 abundance in stool | -0.06746334 | 0.244569798518745 | 0.800577252834174 |
| GCST90032534 | T4eVfC | outcome | Raoultella abundance in stool | 0.00714622689090975 | 0.0331944961809861 | 0.834345771672435 |
| GCST90032535 | T4eVfC | outcome | RC9 sp900317925 abundance in stool | -0.006219475 | 0.0636023671203617 | 0.924842662106619 |
| GCST90032536 | T4eVfC | outcome | Rhodanobacter abundance in stool | 0.083936074539661 | 0.0438135460658485 | 0.081739100089188 |
| GCST90032537 | T4eVfC | outcome | Rhodococcus abundance in stool | -0.080159169 | 0.0549611666746537 | 0.178708225849317 |
| GCST90032538 | T4eVfC | outcome | Rhodovulum abundance in stool | 0.261839647660885 | 0.243353413974873 | 0.360768278986553 |
| GCST90032539 | T4eVfC | outcome | Romboutsia ilealis abundance in stool | -0.037346988 | 0.0468182978144667 | 0.448072294775645 |
| GCST90032540 | T4eVfC | outcome | Roseibacillus abundance in stool | NA | NA | NA |
| GCST90032541 | T4eVfC | outcome | Rubneribacter sp002159915 abundance in stool | 0.0695741606175211 | 0.106022623348976 | 0.547513992759961 |
| GCST90032542 | T4eVfC | outcome | RUG013 sp001486445 abundance in stool | -0.022802531 | 0.128312001583905 | 0.863982701384278 |
| GCST90032543 | T4eVfC | outcome | RUG147 sp900315495 abundance in stool | -0.087243474 | 0.0515075214104048 | 0.23238374042807 |
| GCST90032544 | T4eVfC | outcome | RUG147 abundance in stool | -0.085073954 | 0.0860067209264244 | 0.42684677013438 |
| GCST90032545 | T4eVfC | outcome | RUG420 sp900317985 abundance in stool | -0.003599148 | 0.0469008856924912 | 0.940344486186992 |
| GCST90032546 | T4eVfC | outcome | RUG472 sp900319345 abundance in stool | 0.105766340656721 | 0.0761649445082982 | 0.21429485808351 |
| GCST90032547 | T4eVfC | outcome | RUG472 abundance in stool | 0.0577040059768037 | 0.0501284247104192 | 0.313809431833368 |
| GCST90032548 | T4eVfC | outcome | Ruminococcus A sp000432335 abundance in stool | -0.124706565 | 0.0618192749792788 | 0.137016186942655 |
| GCST90032549 | T4eVfC | outcome | Ruminococcus C sp000437255 abundance in stool | 0.0201119678198002 | 0.0329244780766835 | 0.554927240438195 |
| GCST90032550 | T4eVfC | outcome | Ruminococcus D bicirculans abundance in stool | -0.01509671 | 0.0569675409864939 | 0.795502503934555 |
| GCST90032551 | T4eVfC | outcome | Ruminococcus D abundance in stool | -0.028082048 | 0.0517413751971508 | 0.59649236145911 |
| GCST90032552 | T4eVfC | outcome | Ruminococcus E sp003521625 abundance in stool | -0.029997015 | 0.0518567866492575 | 0.583998318566513 |
| GCST90032553 | T4eVfC | outcome | Ruminococcus E sp900100595 abundance in stool | 0.00227627967809401 | 0.0304395146446657 | 0.941864221630847 |
| GCST90032554 | T4eVfC | outcome | Ruminococcus E sp900314705 abundance in stool | -0.035198802 | 0.0335817696569851 | 0.311164502367112 |
| GCST90032555 | T4eVfC | outcome | Ruminococcus abundance in stool | -0.034811054 | 0.082227803168796 | 0.693811468119618 |
| GCST90032556 | T4eVfC | outcome | Saccharofermentanaceae abundance in stool | -0.021178508 | 0.0667266233276551 | 0.76377191484554 |
| GCST90032557 | T4eVfC | outcome | Saccharomonospora abundance in stool | 0.00869897660554653 | 0.0327367616158149 | 0.793646667318227 |
| GCST90032558 | T4eVfC | outcome | SAR324 abundance in stool | -0.005095738 | 0.0370904455567717 | 0.895219478998334 |
| GCST90032559 | T4eVfC | outcome | SM23-33 abundance in stool | -0.008928855 | 0.043205845865947 | 0.839251985223551 |
| GCST90032560 | T4eVfC | outcome | Sorangium abundance in stool | -0.036604852 | 0.0640899020485314 | 0.58863685310898 |
| GCST90032561 | T4eVfC | outcome | Spirillospora abundance in stool | -0.027917698 | 0.0343601073304105 | 0.432326899558291 |
| GCST90032562 | T4eVfC | outcome | Spirochaetia abundance in stool | 0.0163429826621153 | 0.0291155909216146 | 0.585837087962269 |
| GCST90032563 | T4eVfC | outcome | Sporomusales abundance in stool | -0.036226991 | 0.0227520606669558 | 0.172205613472986 |
| GCST90032564 | T4eVfC | outcome | Staphylococcus A fleurettii abundance in stool | 0.033740590343989 | 0.033349714899361 | 0.341289394960146 |
| GCST90032565 | T4eVfC | outcome | Staphylococcus aureus abundance in stool | -0.089222102 | 0.0807948229913123 | 0.305968661066131 |
| GCST90032566 | T4eVfC | outcome | Stappia abundance in stool | 0.00704987725130538 | 0.0253954399809302 | 0.789336221241539 |
| GCST90032567 | T4eVfC | outcome | Streptacidiphilus abundance in stool | 0.00288609917495676 | 0.0541699493078996 | 0.95855926400415 |
| GCST90032568 | T4eVfC | outcome | Streptococcus sanguinis abundance in stool | 0.0197960619922128 | 0.033755670417562 | 0.578951578090188 |
| GCST90032569 | T4eVfC | outcome | Succiniclasticum abundance in stool | -0.02449849 | 0.0239388426844811 | 0.32047213697696 |
| GCST90032570 | T4eVfC | outcome | Succinivibrionaceae abundance in stool | 0.0323722753597331 | 0.0214102031351162 | 0.154456514862347 |
| GCST90032571 | T4eVfC | outcome | Succinivibrio abundance in stool | 0.0116706247679787 | 0.0487475705142533 | 0.814521375564169 |
| GCST90032572 | T4eVfC | outcome | Syntrophomonadia abundance in stool | 0.00889123462170755 | 0.0267810279080452 | 0.749610556370673 |
| GCST90032573 | T4eVfC | outcome | Syntrophorhabdaceae abundance in stool | -0.008034665 | 0.0230430207475652 | 0.735346741368298 |
| GCST90032574 | T4eVfC | outcome | Syntrophorhabdia abundance in stool | -0.006292919 | 0.0484726794036904 | 0.90035746992932 |
| GCST90032575 | T4eVfC | outcome | Tannerellaceae abundance in stool | -0.06530638 | 0.0398657849501833 | 0.125353432397555 |
| GCST90032576 | T4eVfC | outcome | Tepidanaerobacteraceae abundance in stool | 0.0231589870143474 | 0.07573850287152 | 0.770106872498472 |
| GCST90032577 | T4eVfC | outcome | Terrisporobacter othiniensis abundance in stool | 0.0462698355407188 | 0.0667864105245329 | 0.538247439511849 |
| GCST90032578 | T4eVfC | outcome | Terrisporobacter abundance in stool | 0.024842067260445 | 0.055279347372362 | 0.665074686387879 |
| GCST90032579 | T4eVfC | outcome | Thermococcaceae abundance in stool | -0.056576546 | 0.0510681839171852 | 0.300114944175411 |
| GCST90032580 | T4eVfC | outcome | Thermococci abundance in stool | -0.039846494 | 0.0680485295054401 | 0.579513204986039 |
| GCST90032581 | T4eVfC | outcome | Thermoplasmatota abundance in stool | -0.014334423 | 0.0277175234116954 | 0.615283356845812 |
| GCST90032582 | T4eVfC | outcome | Thermoprotei abundance in stool | 0.0632593503573575 | 0.0652131094764931 | 0.364341964361868 |
| GCST90032583 | T4eVfC | outcome | Thioalkalivibrionaceae abundance in stool | 0.00184257442263761 | 0.037361897568284 | 0.963766441821126 |
| GCST90032584 | T4eVfC | outcome | TMED109 abundance in stool | -0.010199176 | 0.0907869848531826 | 0.913018146212102 |
| GCST90032585 | T4eVfC | outcome | Treponema D abundance in stool | -0.003801496 | 0.028131876611368 | 0.893858844567332 |
| GCST90032586 | T4eVfC | outcome | Treponemataceae abundance in stool | 0.0384446300489597 | 0.0227178960240937 | 0.106931085934102 |
| GCST90032587 | T4eVfC | outcome | Turicibacteraceae abundance in stool | 0.0607906269883924 | 0.0675022401702585 | 0.418735046563369 |
| GCST90032588 | T4eVfC | outcome | Turicibacter sp001543345 abundance in stool | 0.0630541687296716 | 0.0426820020832291 | 0.16535309994341 |
| GCST90032589 | T4eVfC | outcome | Turicibacter abundance in stool | 0.0463171994042173 | 0.0680598964085573 | 0.521545977811198 |
| GCST90032590 | T4eVfC | outcome | UBA1033 sp001695555 abundance in stool | 0.0663736417191717 | 0.0517469396860778 | 0.240448353208055 |
| GCST90032591 | T4eVfC | outcome | UBA1066 sp900317515 abundance in stool | -0.104360817 | 0.127615292952618 | 0.563605542921136 |
| GCST90032592 | T4eVfC | outcome | UBA1066 abundance in stool | 0.0401316342864579 | 0.036696050447728 | 0.293971333513051 |
| GCST90032593 | T4eVfC | outcome | UBA11471 sp000434215 abundance in stool | -0.06400179 | 0.0798239432122152 | 0.449033821274559 |
| GCST90032594 | T4eVfC | outcome | UBA11471 abundance in stool | -0.06331386 | 0.0802975786776951 | 0.456278248147713 |
| GCST90032595 | T4eVfC | outcome | UBA1191 abundance in stool | -0.010595294 | 0.0263520310956342 | 0.701565607757037 |
| GCST90032596 | T4eVfC | outcome | UBA11963 sp002362595 abundance in stool | -0.000421476 | 0.0688689807388071 | 0.995315411624034 |
| GCST90032597 | T4eVfC | outcome | UBA11963 abundance in stool | -0.011428808 | 0.0397656928000272 | 0.788077872419603 |
| GCST90032598 | T4eVfC | outcome | UBA1206 sp000433115 abundance in stool | -0.0229003 | 0.0479253108520791 | 0.64555232952275 |
| GCST90032599 | T4eVfC | outcome | UBA1375 sp002305795 abundance in stool | 0.0326534200162952 | 0.0214053165911601 | 0.15812287637964 |
| GCST90032600 | T4eVfC | outcome | UBA1407 abundance in stool | -0.016679481 | 0.0688295476935787 | 0.831108198291024 |
| GCST90032601 | T4eVfC | outcome | UBA1409 abundance in stool | -0.045773595 | 0.0970058517118963 | 0.669222093604641 |
| GCST90032602 | T4eVfC | outcome | UBA1417 sp003531055 abundance in stool | 0.00129974693279112 | 0.0641259360752191 | 0.984486276737928 |
| GCST90032603 | T4eVfC | outcome | UBA1446 sp002329245 abundance in stool | -0.01034794 | 0.0472937041545131 | 0.833047163337884 |
| GCST90032604 | T4eVfC | outcome | UBA1448 sp002329405 abundance in stool | -0.004147959 | 0.0344052228453067 | 0.90797399756485 |
| GCST90032605 | T4eVfC | outcome | UBA1448 abundance in stool | 0.0254044007796589 | 0.0373326788043788 | 0.515403484546235 |
| GCST90032606 | T4eVfC | outcome | UBA1611 abundance in stool | -0.063136102 | 0.0329019966300648 | 0.0964838301986593 |
| GCST90032607 | T4eVfC | outcome | UBA1777 sp002320035 abundance in stool | 0.0990607942201486 | 1.15899753042486 | 0.945719330062833 |
| GCST90032608 | T4eVfC | outcome | UBA1777 sp900316255 abundance in stool | -0.027807493 | 0.0381032100361717 | 0.493011970849674 |
| GCST90032609 | T4eVfC | outcome | UBA1777 sp900319275 abundance in stool | 0.00105444123301147 | 0.0619980041981305 | 0.987498381339323 |
| GCST90032610 | T4eVfC | outcome | UBA1777 sp900319835 abundance in stool | -0.070241687 | 0.0376120439034235 | 0.0946703844126431 |
| GCST90032611 | T4eVfC | outcome | UBA2658 sp002841545 abundance in stool | -0.001112825 | 0.0394454228951172 | 0.978844685187903 |
| GCST90032612 | T4eVfC | outcome | UBA2821 abundance in stool | 0.0319530953525495 | 0.0393239583502233 | 0.427086852929719 |
| GCST90032613 | T4eVfC | outcome | UBA2922 sp900313925 abundance in stool | 0.0585402839921685 | 0.0476093739979293 | 0.250022642764953 |
| GCST90032614 | T4eVfC | outcome | UBA3282 sp002493835 abundance in stool | -0.075258339 | 0.0554518576159375 | 0.232771228332544 |
| GCST90032615 | T4eVfC | outcome | UBA3792 abundance in stool | 0.0812463116680701 | 0.150232600211826 | 0.603370678171058 |
| GCST90032616 | T4eVfC | outcome | UBA3855 sp900316885 abundance in stool | 0.0286938649241039 | 0.0663102969498139 | 0.678236452472736 |
| GCST90032617 | T4eVfC | outcome | UBA5394 sp002409725 abundance in stool | -0.028085591 | 0.0353276296459892 | 0.447069053564926 |
| GCST90032618 | T4eVfC | outcome | UBA6382 abundance in stool | -0.087978494 | 0.0671187172401928 | 0.23129263090515 |
| GCST90032619 | T4eVfC | outcome | UBA6398 sp002451695 abundance in stool | 0.0407535167007103 | 0.0224863163573057 | 0.107500207976729 |
| GCST90032620 | T4eVfC | outcome | UBA6398 abundance in stool | 0.0940920457272165 | 0.056376863384642 | 0.133672807788791 |
| GCST90032621 | T4eVfC | outcome | UBA644 abundance in stool | -0.005423681 | 0.0821154485845965 | 0.949484263592504 |
| GCST90032622 | T4eVfC | outcome | UBA6960 abundance in stool | -0.002396108 | 0.0524215547528493 | 0.965312021746501 |
| GCST90032623 | T4eVfC | outcome | UBA7102 sp002315655 abundance in stool | -0.034488907 | 0.0420858533747624 | 0.433671741705958 |
| GCST90032624 | T4eVfC | outcome | UBA7177 sp002491225 abundance in stool | -0.00659852 | 0.0295942204959601 | 0.827647860617102 |
| GCST90032625 | T4eVfC | outcome | UBA7177 abundance in stool | 0.020890036751389 | 0.0244820099274331 | 0.407865794574081 |
| GCST90032626 | T4eVfC | outcome | UBA7182 sp002491115 abundance in stool | -0.002105736 | 0.0539707409452404 | 0.969645367300735 |
| GCST90032627 | T4eVfC | outcome | UBA7182 abundance in stool | -0.027060468 | 0.0550828940413202 | 0.636429985328177 |
| GCST90032628 | T4eVfC | outcome | UBA737 sp002451855 abundance in stool | 0.00811714971946912 | 0.0571766405770657 | 0.891753720575666 |
| GCST90032629 | T4eVfC | outcome | UBA737 abundance in stool | -0.060623192 | 0.0725497715342255 | 0.435380971650563 |
| GCST90032630 | T4eVfC | outcome | UBA7703 abundance in stool | 0.045497339572232 | 0.0296915362620056 | 0.159803587798875 |
| GCST90032631 | T4eVfC | outcome | UBA7748 sp900314535 abundance in stool | 0.00511269529829278 | 0.0238873722606202 | 0.834822130767362 |
| GCST90032632 | T4eVfC | outcome | UBA8517 abundance in stool | -0.000571941 | 0.027700932904487 | 0.984032934880541 |
| GCST90032633 | T4eVfC | outcome | UBA8621 abundance in stool | 0.0731218814500802 | 0.0815501940426603 | 0.410995346854475 |
| GCST90032634 | T4eVfC | outcome | UBA8904 abundance in stool | -0.00839614 | 0.0516782865469057 | 0.876269829218533 |
| GCST90032635 | T4eVfC | outcome | UBA9475 sp002161235 abundance in stool | 0.0282753453492897 | 0.0835988417486452 | 0.746713595536905 |
| GCST90032636 | T4eVfC | outcome | UBA9475 sp002161675 abundance in stool | 0.153324480491948 | 0.149363689990065 | 0.35172064128662 |
| GCST90032637 | T4eVfC | outcome | UBP9 abundance in stool | 0.00683667108572415 | 0.0323221782411522 | 0.835532479462376 |
| GCST90032638 | T4eVfC | outcome | UCG-010 sp003150215 abundance in stool | -0.039899681 | 0.0646147116880897 | 0.563938106158387 |
| GCST90032639 | T4eVfC | outcome | UNC496MF abundance in stool | -0.083536628 | 0.0717533785900037 | 0.277877656463232 |
| GCST90032640 | T4eVfC | outcome | V9D3004 abundance in stool | -0.025558466 | 0.0508936823697828 | 0.627587944946538 |
| GCST90032641 | T4eVfC | outcome | Veillonellaceae abundance in stool | 0.0610481970376606 | 0.0350522142331786 | 0.119746243292331 |
| GCST90032642 | T4eVfC | outcome | Veillonella rogosae abundance in stool | 0.0313312574726802 | 0.0363302270865824 | 0.410856436618894 |
| GCST90032643 | T4eVfC | outcome | Veillonella abundance in stool | NA | NA | NA |
| GCST90032644 | T4eVfC | outcome | Victivallis sp002998355 abundance in stool | -0.004854038 | 0.0809729871922537 | 0.954520584372531 |

**Supplementary Table 2:MRPRESSO analysis of gut microbiota.**

|  | Exposure | MR Analysis | Causal Estimate | Sd | T-stat | P-value | RSSobs | Pvalue | phe |
| --- | --- | --- | --- | --- | --- | --- | --- | --- | --- |
| 1 | beta.exposure | Raw | -0.609921129 | 0.240781932 | -2.533085117 | 0.032072609 | 12.2377315 | 0.412 | Bifidobacterium breve abundance in stool |
| 2 | beta.exposure | Outlier-corrected | NA | NA | NA | NA | 12.2377315 | 0.412 | Bifidobacterium breve abundance in stool |
| 3 | beta.exposure | Raw | 0.22552072 | 0.083758946 | 2.692497118 | 0.020935533 | 7.998488452 | 0.833 | Blautia sp000436935 abundance in stool |
| 4 | beta.exposure | Outlier-corrected | NA | NA | NA | NA | 7.998488452 | 0.833 | Blautia sp000436935 abundance in stool |
| 5 | beta.exposure | Raw | -0.509364662 | 0.214474588 | -2.37494179 | 0.035084441 | 15.61245662 | 0.359 | CAG-269 sp002372935 abundance in stool |
| 6 | beta.exposure | Outlier-corrected | NA | NA | NA | NA | 15.61245662 | 0.359 | CAG-269 sp002372935 abundance in stool |
| 7 | beta.exposure | Raw | 0.14131414 | 0.060084517 | 2.351922698 | 0.043168894 | 8.117337846 | 0.687 | CAG-274 sp000432155 abundance in stool |
| 8 | beta.exposure | Outlier-corrected | NA | NA | NA | NA | 8.117337846 | 0.687 | CAG-274 sp000432155 abundance in stool |
| 9 | beta.exposure | Raw | 0.509934635 | 0.175524439 | 2.905205894 | 0.01228454 | 13.62192548 | 0.574 | CHKCI006 sp900018345 abundance in stool |
| 10 | beta.exposure | Outlier-corrected | NA | NA | NA | NA | 13.62192548 | 0.574 | CHKCI006 sp900018345 abundance in stool |
| 11 | beta.exposure | Raw | -0.582167132 | 0.172270422 | -3.3793795 | 0.019687483 | 2.681397101 | 0.887 | Ezakiellaceae abundance in stool |
| 12 | beta.exposure | Outlier-corrected | NA | NA | NA | NA | 2.681397101 | 0.887 | Ezakiellaceae abundance in stool |
| 15 | beta.exposure | Raw | -0.48742289 | 0.151090194 | -3.226039217 | 0.009081705 | 5.964177564 | 0.919 | Actinobacteria abundance in stool |
| 16 | beta.exposure | Outlier-corrected | NA | NA | NA | NA | 5.964177564 | 0.919 | Actinobacteria abundance in stool |
| 17 | beta.exposure | Raw | 0.906865486 | 0.153762591 | 5.897829106 | 0.000103443 | 4.633273349 | 0.982 | Paenibacillus J abundance in stool |
| 18 | beta.exposure | Outlier-corrected | NA | NA | NA | NA | 4.633273349 | 0.982 | Paenibacillus J abundance in stool |
| 19 | beta.exposure | Raw | -0.478599416 | 0.13836621 | -3.45893276 | 0.003835906 | 17.01491359 | 0.428 | Parabacteroides johnsonii abundance in stool |
| 20 | beta.exposure | Outlier-corrected | NA | NA | NA | NA | 17.01491359 | 0.428 | Parabacteroides johnsonii abundance in stool |
| 21 | beta.exposure | Raw | -0.290152176 | 0.125434638 | -2.313174269 | 0.039249814 | 12.63650789 | 0.594 | Prevotella sp002437565 abundance in stool |
| 22 | beta.exposure | Outlier-corrected | NA | NA | NA | NA | 12.63650789 | 0.594 | Prevotella sp002437565 abundance in stool |
| 23 | beta.exposure | Raw | -0.648199982 | 0.203232597 | -3.189448897 | 0.015288362 | 5.394889177 | 0.776 | UBA737 sp002451855 abundance in stool |
| 24 | beta.exposure | Outlier-corrected | NA | NA | NA | NA | 5.394889177 | 0.776 | UBA737 sp002451855 abundance in stool |

**Supplementary Table 3: MR-Egger regression analyses of lipids.**

| id.exposure | id.outcome | outcome | exposure | egger_intercept | se | pval |
| --- | --- | --- | --- | --- | --- | --- |
| GCST90277238 | WoOryb | outcome | Sterol ester (27:1/14:0) levels | 0.00193062252104262 | 0.0539302212942117 | 0.971948431559234 |
| GCST90277239 | WoOryb | outcome | Sterol ester (27:1/15:0) levels | 0.0393119384791342 | 0.0211744785088821 | 0.0930369453200441 |
| GCST90277240 | WoOryb | outcome | Sterol ester (27:1/16:0) levels | 0.00605694045622114 | 0.0216959281384062 | 0.783127408587345 |
| GCST90277241 | WoOryb | outcome | Sterol ester (27:1/16:1) levels | -0.007003484 | 0.0313379494050659 | 0.826388109685144 |
| GCST90277242 | WoOryb | outcome | Sterol ester (27:1/17:0) levels | 0.022231501679137 | 0.0344424228071694 | 0.529061088719938 |
| GCST90277243 | WoOryb | outcome | Sterol ester (27:1/17:1) levels | 0.0190331134089942 | 0.0174056542282049 | 0.30257615472926 |
| GCST90277244 | WoOryb | outcome | Sterol ester (27:1/18:0) levels | -0.001306353 | 0.026856014845675 | 0.961739600955385 |
| GCST90277245 | WoOryb | outcome | Sterol ester (27:1/18:1) levels | 0.0163048871973111 | 0.0223443582904119 | 0.477600423630614 |
| GCST90277246 | WoOryb | outcome | Sterol ester (27:1/18:2) levels | 0.0380445788689508 | 0.0324095230056653 | 0.261504525824288 |
| GCST90277247 | WoOryb | outcome | Sterol ester (27:1/18:3) levels | -0.010660651 | 0.034589960397747 | 0.764253746484418 |
| GCST90277248 | WoOryb | outcome | Sterol ester (27:1/20:2) levels | 0.0389680576586187 | 0.0366208000508325 | 0.305294875351388 |
| GCST90277249 | WoOryb | outcome | Sterol ester (27:1/20:3) levels | 0.00173150722168179 | 0.0280674448967693 | 0.951747335711241 |
| GCST90277250 | WoOryb | outcome | Sterol ester (27:1/20:4) levels | -0.011769388 | 0.0152778235889491 | 0.454860334439034 |
| GCST90277251 | WoOryb | outcome | Sterol ester (27:1/20:5) levels | -0.000286428 | 0.0169522147501974 | 0.986716131118884 |
| GCST90277252 | WoOryb | outcome | Sterol ester (27:1/22:6) levels | -0.092419189 | 0.0315537645972578 | 0.0137163147155384 |
| GCST90277253 | WoOryb | outcome | Ceramide (d40:1) levels | -0.018902223 | 0.0236508519796905 | 0.435188284744527 |
| GCST90277254 | WoOryb | outcome | Ceramide (d40:2) levels | -0.03893118 | 0.0643200801837584 | 0.567170633257668 |
| GCST90277255 | WoOryb | outcome | Ceramide (d42:1) levels | -0.011171383 | 0.0253195766077458 | 0.66630633792463 |
| GCST90277256 | WoOryb | outcome | Ceramide (d42:2) levels | 0.0316490494487976 | 0.0230647409845776 | 0.18599082434643 |
| GCST90277257 | WoOryb | outcome | Cholesterol levels | -0.036564875 | 0.0419052867739901 | 0.395809150052262 |
| GCST90277258 | WoOryb | outcome | Diacylglycerol (16:0_18:1) levels | -0.015064407 | 0.0374978374763332 | 0.695570027806613 |
| GCST90277259 | WoOryb | outcome | Diacylglycerol (16:0_18:2) levels | 0.016249722362979 | 0.0138278688268026 | 0.2571298279436 |
| GCST90277260 | WoOryb | outcome | Diacylglycerol (16:1_18:1) levels | -0.001512301 | 0.0264071183349041 | 0.955358227666962 |
| GCST90277261 | WoOryb | outcome | Diacylglycerol (18:1_18:1) levels | -0.014471528 | 0.0252687394680684 | 0.578362702736943 |
| GCST90277262 | WoOryb | outcome | Diacylglycerol (18:1_18:2) levels | -0.024435306 | 0.0229257130266927 | 0.299850292198834 |
| GCST90277263 | WoOryb | outcome | Diacylglycerol (18:1_18:3) levels | -0.022040601 | 0.0321309366360732 | 0.508328039810608 |
| GCST90277264 | WoOryb | outcome | Phosphatidylcholine (16:0_0:0) levels | -0.042080643 | 0.0210196076157348 | 0.0605913665478053 |
| GCST90277265 | WoOryb | outcome | Phosphatidylcholine (18:0_0:0) levels | 0.00554674746187064 | 0.0295930686945474 | 0.853832178974953 |
| GCST90277266 | WoOryb | outcome | Phosphatidylcholine (18:1_0:0) levels | -0.008017913 | 0.117264321523064 | 0.946714425564642 |
| GCST90277267 | WoOryb | outcome | Phosphatidylcholine (18:2_0:0) levels | 0.0277637432893696 | 0.070090257688444 | 0.708359704994547 |
| GCST90277268 | WoOryb | outcome | Phosphatidylcholine (20:4_0:0) levels | -0.002200079 | 0.0150790973343513 | 0.886420542895535 |
| GCST90277269 | WoOryb | outcome | Phosphatidylethanolamine (18:0_0:0) levels | -0.016138258 | 0.0772674398104905 | 0.838748791682848 |
| GCST90277270 | WoOryb | outcome | Phosphatidylethanolamine (18:1_0:0) levels | 0.0112422737228665 | 0.0176010271656617 | 0.543324882692937 |
| GCST90277271 | WoOryb | outcome | Phosphatidylethanolamine (18:2_0:0) levels | 0.00827977286195628 | 0.0167655640035411 | 0.629065657849249 |
| GCST90277272 | WoOryb | outcome | Phosphatidylcholine (14:0_16:0) levels | -0.012773692 | 0.0226618812353418 | 0.586750567533905 |
| GCST90277273 | WoOryb | outcome | Phosphatidylcholine (14:0_18:1) levels | -0.036708646 | 0.0873404863860776 | 0.685332708983086 |
| GCST90277274 | WoOryb | outcome | Phosphatidylcholine (14:0_18:2) levels | -0.008204351 | 0.0347907496066931 | 0.816987309101483 |
| GCST90277275 | WoOryb | outcome | Phosphatidylcholine (15:0_18:1) levels | -0.009801185 | 0.0349920834268634 | 0.786509206292147 |
| GCST90277276 | WoOryb | outcome | Phosphatidylcholine (15:0_18:2) levels | 0.038625724046251 | 0.0167634531828166 | 0.0305868250137298 |
| GCST90277277 | WoOryb | outcome | Phosphatidylcholine (16:0_16:0) levels | 0.0168200603830514 | 0.033439749328741 | 0.621820323921637 |
| GCST90277278 | WoOryb | outcome | Phosphatidylcholine (16:0_16:1) levels | -0.042102216 | 0.0481255872765511 | 0.402183518806661 |
| GCST90277279 | WoOryb | outcome | Phosphatidylcholine (16:0_17:1) levels | 0.00749459087654355 | 0.0359186542895944 | 0.839363761038244 |
| GCST90277280 | WoOryb | outcome | Phosphatidylcholine (16:0_18:0) levels | 0.0248088835103083 | 0.031320663387437 | 0.436066108167005 |
| GCST90277281 | WoOryb | outcome | Phosphatidylcholine (16:0_18:1) levels | 0.0343508943762713 | 0.0278718626254616 | 0.238075274905959 |
| GCST90277282 | WoOryb | outcome | Phosphatidylcholine (16:0_18:2) levels | 0.020690999112695 | 0.0198846062598038 | 0.311143601182999 |
| GCST90277283 | WoOryb | outcome | Phosphatidylcholine (16:0_18:3) levels | 0.00162625215008443 | 0.0292600358484436 | 0.956591625562842 |
| GCST90277284 | WoOryb | outcome | Phosphatidylcholine (16:0_20:1) levels | 0.219806727885313 | 0.143224251791541 | 0.185445390710197 |
| GCST90277285 | WoOryb | outcome | Phosphatidylcholine (16:0_20:2) levels | 0.0336276891449119 | 0.0257373273441076 | 0.220607489308853 |
| GCST90277286 | WoOryb | outcome | Phosphatidylcholine (16:0_20:3) levels | -0.021420252 | 0.0405405653871045 | 0.613576522478766 |
| GCST90277287 | WoOryb | outcome | Phosphatidylcholine (16:0_20:4) levels | -0.00436271 | 0.0174496050936063 | 0.806802534437284 |
| GCST90277288 | WoOryb | outcome | Phosphatidylcholine (16:0_20:5) levels | -0.022282317 | 0.0171465363784709 | 0.212177490757914 |
| GCST90277289 | WoOryb | outcome | Phosphatidylcholine (16:0_22:4) levels | 0.00509703037410161 | 0.0192410812253689 | 0.794944132198337 |
| GCST90277290 | WoOryb | outcome | Phosphatidylcholine (16:0_22:5) levels | -0.000115849 | 0.0152505564606557 | 0.994027471093413 |
| GCST90277291 | WoOryb | outcome | Phosphatidylcholine (16:0_22:6) levels | -0.022019831 | 0.0290779257360339 | 0.466356941830575 |
| GCST90277292 | WoOryb | outcome | Phosphatidylcholine (16:1_18:0) levels | -0.036861056 | 0.0281035059720726 | 0.237599675525689 |
| GCST90277293 | WoOryb | outcome | Phosphatidylcholine (16:1_18:1) levels | 0.0081115222962857 | 0.0223432398046407 | 0.721996679134587 |
| GCST90277294 | WoOryb | outcome | Phosphatidylcholine (16:1_18:2) levels | 0.0194485212591382 | 0.0176307982170576 | 0.286299679044305 |
| GCST90277295 | WoOryb | outcome | Phosphatidylcholine (16:1_20:4) levels | -0.018208676 | 0.0283411067263221 | 0.532642966840028 |
| GCST90277296 | WoOryb | outcome | Phosphatidylcholine (17:0_18:1) levels | -0.140294484 | 0.080966119923499 | 0.126740164224447 |
| GCST90277297 | WoOryb | outcome | Phosphatidylcholine (17:0_18:2) levels | 0.00506756562286815 | 0.0249735331079367 | 0.84192639367697 |
| GCST90277298 | WoOryb | outcome | Phosphatidylcholine (17:0_20:4) levels | -0.012281672 | 0.0167129341621991 | 0.471890913740999 |
| GCST90277299 | WoOryb | outcome | Phosphatidylcholine (18:0_18:1) levels | -0.026640044 | 0.0321331110524166 | 0.419271448637089 |
| GCST90277300 | WoOryb | outcome | Phosphatidylcholine (18:0_18:2) levels | 0.0177613831318323 | 0.01506799363125 | 0.250052922183017 |
| GCST90277301 | WoOryb | outcome | Phosphatidylcholine (18:0_18:3) levels | 0.0338415495595615 | 0.0384046435329159 | 0.412122133290021 |
| GCST90277302 | WoOryb | outcome | Phosphatidylcholine (18:0_20:2) levels | 0.0498484397002901 | 0.0245082366536141 | 0.0724708047678599 |
| GCST90277303 | WoOryb | outcome | Phosphatidylcholine (18:0_20:3) levels | 0.0243529160106394 | 0.0247809126522816 | 0.343677686001994 |
| GCST90277304 | WoOryb | outcome | Phosphatidylcholine (18:0_20:4) levels | 0.00221019307646056 | 0.0133758027132603 | 0.870962871969666 |
| GCST90277305 | WoOryb | outcome | Phosphatidylcholine (18:0_20:5) levels | -0.020799385 | 0.0251759134716854 | 0.426268582545433 |
| GCST90277306 | WoOryb | outcome | Phosphatidylcholine (18:0_22:5) levels | -0.007210693 | 0.0198349664681359 | 0.72068709354452 |
| GCST90277307 | WoOryb | outcome | Phosphatidylcholine (18:0_22:6) levels | -0.033761204 | 0.0337838465599071 | 0.333484068760175 |
| GCST90277308 | WoOryb | outcome | Phosphatidylcholine (18:1_18:1) levels | -0.001955805 | 0.026236432676818 | 0.941804699323614 |
| GCST90277309 | WoOryb | outcome | Phosphatidylcholine (18:1_18:2) levels | 0.0330622417896613 | 0.0174263656725588 | 0.0772157438102545 |
| GCST90277310 | WoOryb | outcome | Phosphatidylcholine (18:1_18:3) levels | -0.00557269 | 0.019913224396751 | 0.785296821778688 |
| GCST90277311 | WoOryb | outcome | Phosphatidylcholine (18:1_20:2) levels | -0.009144775 | 0.0236415053331891 | 0.705668706480916 |
| GCST90277312 | WoOryb | outcome | Phosphatidylcholine (18:1_20:3) levels | 0.0596917053990756 | 0.03817474001234 | 0.143876675260154 |
| GCST90277313 | WoOryb | outcome | Phosphatidylcholine (18:1_20:4) levels | 0.00920706631857621 | 0.0142973872061598 | 0.528189270211234 |
| GCST90277314 | WoOryb | outcome | Phosphatidylcholine (18:2_18:2) levels | 0.0328102969494685 | 0.0277867106315463 | 0.257358311607435 |
| GCST90277315 | WoOryb | outcome | Phosphatidylcholine (18:2_20:1) levels | 0.00679269649519233 | 0.0223956395919489 | 0.76855204163781 |
| GCST90277316 | WoOryb | outcome | Phosphatidylcholine (18:2_20:3) levels | 0.00791391147912522 | 0.0214702686034122 | 0.719422670629011 |
| GCST90277317 | WoOryb | outcome | Phosphatidylcholine (18:2_20:4) levels | -0.009276646 | 0.0239760886448284 | 0.70253836242159 |
| GCST90277318 | WoOryb | outcome | Phosphatidylcholine (O-16:0_16:0) levels | 0.00975836332064841 | 0.0562773561981301 | 0.86914011793084 |
| GCST90277319 | WoOryb | outcome | Phosphatidylcholine (O-16:0_16:1) levels | 0.0209119642208942 | 0.0457021087407241 | 0.65810628856759 |
| GCST90277320 | WoOryb | outcome | Phosphatidylcholine (O-16:0_18:1) levels | 0.0245783971527744 | 0.0396196851498992 | 0.544332653149952 |
| GCST90277321 | WoOryb | outcome | Phosphatidylcholine (O-16:0_18:2) levels | 0.00989336300472676 | 0.0414126090385318 | 0.816009890687538 |
| GCST90277322 | WoOryb | outcome | Phosphatidylcholine (O-16:0_20:3) levels | 0.00535634777370579 | 0.0260718789453376 | 0.841346008757907 |
| GCST90277323 | WoOryb | outcome | Phosphatidylcholine (O-16:0_20:4) levels | 0.00564987438568859 | 0.0181591597457297 | 0.759718116697769 |
| GCST90277324 | WoOryb | outcome | Phosphatidylcholine (O-16:0_22:5) levels | -0.007158296 | 0.0258700150261277 | 0.791293654905694 |
| GCST90277325 | WoOryb | outcome | Phosphatidylcholine (O-16:1_16:0) levels | 0.0688087389577195 | 0.0489098116509676 | 0.202284740872665 |
| GCST90277326 | WoOryb | outcome | Phosphatidylcholine (O-16:1_18:0) levels | 0.0466096824039402 | 0.0590515935935165 | 0.452689999824494 |
| GCST90277327 | WoOryb | outcome | Phosphatidylcholine (O-16:1_18:1) levels | 0.0886280148674569 | 0.0422260100102144 | 0.0597143127332365 |
| GCST90277328 | WoOryb | outcome | Phosphatidylcholine (O-16:1_18:2) levels | -0.012850433 | 0.0435740911753675 | 0.773554162765316 |
| GCST90277329 | WoOryb | outcome | Phosphatidylcholine (O-16:1_20:3) levels | -0.015698171 | 0.0509785614135306 | 0.763008789698097 |
| GCST90277330 | WoOryb | outcome | Phosphatidylcholine (O-16:1_20:4) levels | -0.009705364 | 0.0189579456797748 | 0.617277209577342 |
| GCST90277331 | WoOryb | outcome | Phosphatidylcholine (O-16:2_18:0) levels | 0.0532221226565185 | 0.0509643627034279 | 0.326872220710988 |
| GCST90277332 | WoOryb | outcome | Phosphatidylcholine (O-17:0_15:0) levels | -0.086766181 | 0.0399675837136316 | 0.162106986389574 |
| GCST90277333 | WoOryb | outcome | Phosphatidylcholine (O-17:0_17:1) levels | 0.000834089092183825 | 0.0185497396805825 | 0.964540856451399 |
| GCST90277334 | WoOryb | outcome | Phosphatidylcholine (O-18:0_14:0) levels | 0.0486216150221992 | 0.042296247304493 | 0.294079863423118 |
| GCST90277335 | WoOryb | outcome | Phosphatidylcholine (O-18:0_16:1) levels | 0.0202224770512015 | 0.0358581860354574 | 0.585208256786187 |
| GCST90277336 | WoOryb | outcome | Phosphatidylcholine (O-18:0_20:4) levels | -0.02519687 | 0.0203122241322831 | 0.236720705901047 |
| GCST90277337 | WoOryb | outcome | Phosphatidylcholine (O-18:1_16:0) levels | 0.0329858735647823 | 0.0413642755865082 | 0.435584750905385 |
| GCST90277338 | WoOryb | outcome | Phosphatidylcholine (O-18:1_18:2) levels | 0.0330464005240716 | 0.0275805970105554 | 0.253977928422804 |
| GCST90277339 | WoOryb | outcome | Phosphatidylcholine (O-18:1_20:3) levels | -0.033631691 | 0.0303695867773078 | 0.300297823545559 |
| GCST90277340 | WoOryb | outcome | Phosphatidylcholine (O-18:1_20:4) levels | -0.012802409 | 0.0224098700937615 | 0.576267256221081 |
| GCST90277341 | WoOryb | outcome | Phosphatidylcholine (O-18:2_16:0) levels | 0.00540698039962118 | 0.0389305463577147 | 0.892295942000681 |
| GCST90277342 | WoOryb | outcome | Phosphatidylcholine (O-18:2_18:1) levels | 0.0252309846083897 | 0.0602921557113491 | 0.685406401889832 |
| GCST90277343 | WoOryb | outcome | Phosphatidylcholine (O-18:2_18:2) levels | -0.002850642 | 0.0304792076213589 | 0.927533547919429 |
| GCST90277344 | WoOryb | outcome | Phosphatidylcholine (O-18:2_20:4) levels | -0.042250527 | 0.0373047131103775 | 0.286661784207915 |
| GCST90277345 | WoOryb | outcome | Phosphatidylethanolamine (16:0_18:2) levels | 0.019693706239654 | 0.0212400963641751 | 0.368504815409619 |
| GCST90277346 | WoOryb | outcome | Phosphatidylethanolamine (16:0_20:4) levels | -0.021463226 | 0.0194680109767033 | 0.288842200893349 |
| GCST90277347 | WoOryb | outcome | Phosphatidylethanolamine (18:0_18:2) levels | -2.65E-05 | 0.0221736151334409 | 0.999064020752332 |
| GCST90277348 | WoOryb | outcome | Phosphatidylethanolamine (18:0_20:4) levels | -0.003027174 | 0.0255870189305519 | 0.908165579079416 |
| GCST90277349 | WoOryb | outcome | Phosphatidylethanolamine (18:1_18:1) levels | -0.004276051 | 0.0233934794020687 | 0.857784064564765 |
| GCST90277350 | WoOryb | outcome | Phosphatidylethanolamine (O-16:1_18:2) levels | 0.129619423946354 | 0.110545498430941 | 0.293797390122229 |
| GCST90277351 | WoOryb | outcome | Phosphatidylethanolamine (O-16:1_20:4) levels | -0.043964691 | 0.0241001857642547 | 0.0868463156143777 |
| GCST90277352 | WoOryb | outcome | Phosphatidylethanolamine (O-16:1_22:5) levels | -0.057115062 | 0.0534046542115237 | 0.34509857165905 |
| GCST90277353 | WoOryb | outcome | Phosphatidylethanolamine (O-18:1_18:2) levels | -0.013539998 | 0.0263446525398651 | 0.614307535088549 |
| GCST90277354 | WoOryb | outcome | Phosphatidylethanolamine (O-18:1_20:4) levels | -0.046961853 | 0.0335811836781218 | 0.187284374364355 |
| GCST90277355 | WoOryb | outcome | Phosphatidylethanolamine (O-18:2_18:1) levels | 0.032030122070696 | 0.0452834634930635 | 0.492873010111377 |
| GCST90277356 | WoOryb | outcome | Phosphatidylethanolamine (O-18:2_18:2) levels | 0.0618963607998112 | 0.0451502634786823 | 0.228740879545516 |
| GCST90277357 | WoOryb | outcome | Phosphatidylethanolamine (O-18:2_20:4) levels | -0.067211084 | 0.0366541054566713 | 0.0896932652629107 |
| GCST90277358 | WoOryb | outcome | Phosphatidylinositol (16:0_18:1) levels | -0.017913873 | 0.0353631681045151 | 0.621636082834084 |
| GCST90277359 | WoOryb | outcome | Phosphatidylinositol (16:0_18:2) levels | 0.000495927374557948 | 0.0259962488716535 | 0.985048990045688 |
| GCST90277360 | WoOryb | outcome | Phosphatidylinositol (16:0_20:4) levels | -0.172302977 | 0.111922451263974 | 0.15807049157965 |
| GCST90277361 | WoOryb | outcome | Phosphatidylinositol (18:0_18:1) levels | -0.026021956 | 0.0281693997059428 | 0.368534616163791 |
| GCST90277362 | WoOryb | outcome | Phosphatidylinositol (18:0_18:2) levels | -0.003665461 | 0.0167157365247973 | 0.828897683841859 |
| GCST90277363 | WoOryb | outcome | Phosphatidylinositol (18:0_20:3) levels | 0.00119298124725228 | 0.0196944362914014 | 0.952448242570323 |
| GCST90277364 | WoOryb | outcome | Phosphatidylinositol (18:0_20:4) levels | 4.03284794989139e-05 | 0.0188915781302354 | 0.998321581313087 |
| GCST90277365 | WoOryb | outcome | Phosphatidylinositol (18:1_18:1) levels | 0.0341131588650874 | 0.0371505504883152 | 0.375215270252561 |
| GCST90277366 | WoOryb | outcome | Phosphatidylinositol (18:1_18:2) levels | -0.039069618 | 0.0417930370963689 | 0.366913741442651 |
| GCST90277367 | WoOryb | outcome | Phosphatidylinositol (18:1_20:4) levels | -0.031352619 | 0.0256914983061423 | 0.242489737933853 |
| GCST90277368 | WoOryb | outcome | Sphingomyelin (d32:1) levels | -0.005490042 | 0.0182988039994797 | 0.768569037428704 |
| GCST90277369 | WoOryb | outcome | Sphingomyelin (d34:0) levels | 0.0417808164774156 | 0.0224653416016559 | 0.0856885802568209 |
| GCST90277370 | WoOryb | outcome | Sphingomyelin (d34:1) levels | 0.0224152417555123 | 0.0368819335223864 | 0.550940456915399 |
| GCST90277371 | WoOryb | outcome | Sphingomyelin (d34:2) levels | -0.005892142 | 0.0194918632938337 | 0.767608439987809 |
| GCST90277372 | WoOryb | outcome | Sphingomyelin (d36:1) levels | -0.005773949 | 0.0421103511720007 | 0.892462172100277 |
| GCST90277373 | WoOryb | outcome | Sphingomyelin (d36:2) levels | -0.0226645 | 0.0331265633379837 | 0.509406224090527 |
| GCST90277374 | WoOryb | outcome | Sphingomyelin (d38:1) levels | -0.03564798 | 0.0234373114453711 | 0.152206474353473 |
| GCST90277375 | WoOryb | outcome | Sphingomyelin (d38:2) levels | -0.095784371 | 0.069061962619176 | 0.20288019549205 |
| GCST90277376 | WoOryb | outcome | Sphingomyelin (d40:1) levels | 0.0360252858315691 | 0.0349849374597665 | 0.318449403571061 |
| GCST90277377 | WoOryb | outcome | Sphingomyelin (d40:2) levels | -0.039734123 | 0.0259894000390147 | 0.14711107179174 |
| GCST90277378 | WoOryb | outcome | Sphingomyelin (d42:2) levels | 0.0126658653405707 | 0.0228008025040925 | 0.584157410467994 |
| GCST90277379 | WoOryb | outcome | Triacylglycerol (46:1) levels | -0.023738305 | 0.0249165516213629 | 0.356896172147336 |
| GCST90277380 | WoOryb | outcome | Triacylglycerol (46:2) levels | -0.006450001 | 0.0372821009900745 | 0.865123603510901 |
| GCST90277381 | WoOryb | outcome | Triacylglycerol (48:0) levels | 0.0149401660249364 | 0.0193346486750237 | 0.461908032435273 |
| GCST90277382 | WoOryb | outcome | Triacylglycerol (48:1) levels | 0.0148777208776254 | 0.0289202845271739 | 0.613977654805307 |
| GCST90277383 | WoOryb | outcome | Triacylglycerol (48:2) levels | -0.007155899 | 0.0391152431736327 | 0.857464837796015 |
| GCST90277384 | WoOryb | outcome | Triacylglycerol (48:3) levels | 0.0243345559686221 | 0.0431875336971534 | 0.579709939885724 |
| GCST90277385 | WoOryb | outcome | Triacylglycerol (49:1) levels | -0.02237334 | 0.0313420294156959 | 0.487942350740533 |
| GCST90277386 | WoOryb | outcome | Triacylglycerol (49:2) levels | 0.020623931870569 | 0.0431771823125807 | 0.647448352587422 |
| GCST90277387 | WoOryb | outcome | Triacylglycerol (50:1) levels | -0.014524421 | 0.0274644735867313 | 0.605825845031575 |
| GCST90277388 | WoOryb | outcome | Triacylglycerol (50:2) levels | 0.0430803813859177 | 0.0424961664416998 | 0.334602840596544 |
| GCST90277389 | WoOryb | outcome | Triacylglycerol (50:3) levels | 0.0164930643007193 | 0.0269861801069775 | 0.549674196266747 |
| GCST90277390 | WoOryb | outcome | Triacylglycerol (50:4) levels | -0.000718208 | 0.0220332828840404 | 0.97431945911077 |
| GCST90277391 | WoOryb | outcome | Triacylglycerol (50:5) levels | 0.0139162406038859 | 0.0180369741060641 | 0.456641798971192 |
| GCST90277392 | WoOryb | outcome | Triacylglycerol (51:1) levels | 0.069124479192062 | 0.0521045096783798 | 0.217298798202046 |
| GCST90277393 | WoOryb | outcome | Triacylglycerol (51:2) levels | -0.0072864 | 0.0264726328848368 | 0.787811714182763 |
| GCST90277394 | WoOryb | outcome | Triacylglycerol (51:3) levels | -0.004267423 | 0.0282650959539106 | 0.882310247252132 |
| GCST90277395 | WoOryb | outcome | Triacylglycerol (51:4) levels | -0.0198662 | 0.035630959195703 | 0.594520997530795 |
| GCST90277396 | WoOryb | outcome | Triacylglycerol (52:2) levels | 0.011895864824774 | 0.03101896868736 | 0.708054794881519 |
| GCST90277397 | WoOryb | outcome | Triacylglycerol (52:3) levels | -0.01268775 | 0.021408836917297 | 0.561222876380112 |
| GCST90277398 | WoOryb | outcome | Triacylglycerol (52:4) levels | 0.00391923735166006 | 0.0238683136652956 | 0.871508212056138 |
| GCST90277399 | WoOryb | outcome | Triacylglycerol (52:5) levels | 0.0170236645619056 | 0.0251214923977861 | 0.507673083202483 |
| GCST90277400 | WoOryb | outcome | Triacylglycerol (52:6) levels | -2.78E-05 | 0.0313369862622804 | 0.999307330264059 |
| GCST90277401 | WoOryb | outcome | Triacylglycerol (53:2) levels | -0.040070676 | 0.025160517013726 | 0.132099757644197 |
| GCST90277402 | WoOryb | outcome | Triacylglycerol (53:3) levels | -0.029229868 | 0.0217394023354841 | 0.197521158822175 |
| GCST90277403 | WoOryb | outcome | Triacylglycerol (53:4) levels | 0.0187778236487934 | 0.0234538358293799 | 0.433787395808102 |
| GCST90277404 | WoOryb | outcome | Triacylglycerol (54:3) levels | -0.023590868 | 0.0222820244313612 | 0.305441086355136 |
| GCST90277405 | WoOryb | outcome | Triacylglycerol (54:4) levels | 0.00144560061595389 | 0.0225824320042534 | 0.949705474703893 |
| GCST90277406 | WoOryb | outcome | Triacylglycerol (54:5) levels | 0.0100565359155079 | 0.0249094356221785 | 0.692514841252809 |
| GCST90277407 | WoOryb | outcome | Triacylglycerol (54:6) levels | 0.0115891865080236 | 0.0281696650288112 | 0.688676228958869 |
| GCST90277408 | WoOryb | outcome | Triacylglycerol (54:7) levels | 0.0128557645004597 | 0.0255435915442716 | 0.624687609689383 |
| GCST90277409 | WoOryb | outcome | Triacylglycerol (56:3) levels | -0.00250985 | 0.0365088852389661 | 0.946546803183831 |
| GCST90277410 | WoOryb | outcome | Triacylglycerol (56:4) levels | -0.042253318 | 0.0324305842823098 | 0.221816340691049 |
| GCST90277411 | WoOryb | outcome | Triacylglycerol (56:5) levels | 0.0084723959801136 | 0.0308698219394506 | 0.787478339372937 |
| GCST90277412 | WoOryb | outcome | Triacylglycerol (56:6) levels | -0.013186641 | 0.0226395116560015 | 0.568374118593662 |
| GCST90277413 | WoOryb | outcome | Triacylglycerol (56:7) levels | 0.00435975707855211 | 0.0319409487778054 | 0.892945576650103 |
| GCST90277414 | WoOryb | outcome | Triacylglycerol (56:8) levels | 0.0186010872147677 | 0.0268633928481588 | 0.49704213761368 |
| GCST90277415 | WoOryb | outcome | Triacylglycerol (58:7) levels | -0.00151098 | 0.0372881326775722 | 0.968292905076318 |
| GCST90277416 | WoOryb | outcome | Triacylglycerol (58:8) levels | 0.000630689845945783 | 0.0395943954680852 | 0.987604544078632 |

**Supplementary Table 4:MRPRESSO analysis of lipids.**

|  | Exposure | MR Analysis | Causal Estimate | Sd | T-stat | P-value | RSSobs | Pvalue | phe |
| --- | --- | --- | --- | --- | --- | --- | --- | --- | --- |
| 1 | beta.exposure | Raw | -0.178630137 | 0.0715182103229156 | -2.497687452 | 0.0246179022942508 | 11.2206253092593 | 0.845 | Phosphatidylcholine (14:0_18:2) levels |
| 2 | beta.exposure | Outlier-corrected | NA | NA | NA | NA | 11.2206253092593 | 0.845 | Phosphatidylcholine (14:0_18:2) levels |
| 3 | beta.exposure | Raw | -0.156782063 | 0.0717832576521162 | -2.184103489 | 0.0538704639491284 | 10.9827256590412 | 0.568 | Phosphatidylcholine (18:2_20:1) levels |
| 4 | beta.exposure | Outlier-corrected | NA | NA | NA | NA | 10.9827256590412 | 0.568 | Phosphatidylcholine (18:2_20:1) levels |
| 5 | beta.exposure | Raw | 0.190188674571861 | 0.0862031662166454 | 2.20628409510945 | 0.0495392488514 | 11.40659622 | 0.605 | Phosphatidylcholine (O-18:0_16:1) levels |
| 6 | beta.exposure | Outlier-corrected | NA | NA | NA | NA | 11.40659622 | 0.605 | Phosphatidylcholine (O-18:0_16:1) levels |
| 7 | beta.exposure | Raw | 0.217184623194246 | 0.0790752990182642 | 2.74655456116685 | 0.0205996995704959 | 7.15664934847245 | 0.825 | Phosphatidylcholine (O-18:2_20:4) levels |
| 8 | beta.exposure | Outlier-corrected | NA | NA | NA | NA | 7.15664934847245 | 0.825 | Phosphatidylcholine (O-18:2_20:4) levels |
| 9 | beta.exposure | Raw | 0.151685377225052 | 0.0612749225522887 | 2.47548868128902 | 0.0257191255186944 | 18.097713156238 | 0.457 | Sphingomyelin (d32:1) levels |
| 10 | beta.exposure | Outlier-corrected | NA | NA | NA | NA | 18.097713156238 | 0.457 | Sphingomyelin (d32:1) levels |
| 11 | beta.exposure | Raw | 0.186732638229205 | 0.0672080373708407 | 2.77842718719565 | 0.0166990122591667 | 7.3475652265949 | 0.883 | Triacylglycerol (54:6) levels |
| 12 | beta.exposure | Outlier-corrected | NA | NA | NA | NA | 7.3475652265949 | 0.883 | Triacylglycerol (54:6) levels |

**Supplementary Table 5:Results of reverse Mendelian randomization: effects of CKD on gut microbiota.**

| exposure | id.exposure | outcome | id.outcome | method | nsnp | b | se | pval | lo_ci | up_ci | or | or_lci95 | or_uci95 |
| --- | --- | --- | --- | --- | --- | --- | --- | --- | --- | --- | --- | --- | --- |
| CKD | Be6RNR | Bifidobacterium breve abundance in stool | GCST90032223 | MR Egger | 5 | -0.052468694 | 0.0541818023897173 | 0.404283809617776 | -0.158665027 | 0.053727638374225 | 0.948884026060356 | 0.853282137585446 | 1.05519716779768 |
| CKD | Be6RNR | Bifidobacterium breve abundance in stool | GCST90032223 | Weighted median | 5 | -0.044776522 | 0.0267042782587085 | 0.0935904748299224 | -0.097116907 | 0.00756386342265147 | 0.95621115014656 | 0.907449912365962 | 1.00759254169818 |
| CKD | Be6RNR | Bifidobacterium breve abundance in stool | GCST90032223 | Inverse variance weighted | 5 | -0.045299128 | 0.0215424634194484 | 0.0354847337871861 | -0.087522356 | -0.0030759 | 0.955711558926802 | 0.916198388558865 | 0.996928825975132 |
| CKD | Be6RNR | Bifidobacterium breve abundance in stool | GCST90032223 | Simple mode | 5 | -0.033968819 | 0.041909495408359 | 0.46310354856183 | -0.11611143 | 0.0481737915271425 | 0.966601643316039 | 0.890376002749788 | 1.04935300814012 |
| CKD | Be6RNR | Bifidobacterium breve abundance in stool | GCST90032223 | Weighted mode | 5 | -0.04398554 | 0.0284112486748692 | 0.196497547073475 | -0.099671588 | 0.0117005070783759 | 0.956967794816908 | 0.905134626549802 | 1.01176922576428 |
| CKD | Be6RNR | Parabacteroides johnsonii abundance in stool | GCST90032499 | MR Egger | 5 | 0.100671419628655 | 0.0888794243921089 | 0.339693544660847 | -0.073532252 | 0.274875091437189 | 1.10591320068673 | 0.929106179608654 | 1.31636623918305 |
| CKD | Be6RNR | Parabacteroides johnsonii abundance in stool | GCST90032499 | Weighted median | 5 | 0.0359789493022585 | 0.034245089991224 | 0.293428149119584 | -0.031141427 | 0.103099325685058 | 1.03663402439172 | 0.969338472671154 | 1.10860151621274 |
| CKD | Be6RNR | Parabacteroides johnsonii abundance in stool | GCST90032499 | Inverse variance weighted | 5 | 0.0149153585928363 | 0.0415473717066858 | 0.71959775141816 | -0.06651749 | 0.0963482071379404 | 1.01502714765362 | 0.935646551299702 | 1.10114242289749 |
| CKD | Be6RNR | Parabacteroides johnsonii abundance in stool | GCST90032499 | Simple mode | 5 | 0.0237317040559761 | 0.0608605965570499 | 0.716457122108835 | -0.095555065 | 0.143018473307794 | 1.02401554181513 | 0.908868313264057 | 1.15375111506861 |
| CKD | Be6RNR | Parabacteroides johnsonii abundance in stool | GCST90032499 | Weighted mode | 5 | 0.0528011185113253 | 0.0317651859344889 | 0.171801021201082 | -0.009458646 | 0.115060882942923 | 1.05421995943382 | 0.990585946366085 | 1.12194174260769 |

**Supplementary Table 6:MR results of causal links between gut microbiota and CKD.**

| exposure | id.exposure | outcome | id.outcome | method | nsnp | b | se | pval | lo_ci | up_ci | or |
| --- | --- | --- | --- | --- | --- | --- | --- | --- | --- | --- | --- |
| Bifidobacterium breve abundance in stool | GCST90032223 | CKD | T4eVfC | MR Egger | 10 | -0.54652191 | 0.847142072285474 | 0.536897352320412 | -2.206920371 | 1.11387655195663 | 0.578959987668945 |
| Bifidobacterium breve abundance in stool | GCST90032223 | CKD | T4eVfC | Weighted median | 10 | -0.622055233 | 0.325817008775361 | 0.0562341592176972 | -1.26065657 | 0.0165461042842184 | 0.536839971835787 |
| Bifidobacterium breve abundance in stool | GCST90032223 | CKD | T4eVfC | Inverse variance weighted | 10 | -0.609921129 | 0.240781932356894 | 0.011306349451903 | -1.081853717 | -0.137988542 | 0.543393725203224 |
| Bifidobacterium breve abundance in stool | GCST90032223 | CKD | T4eVfC | Simple mode | 10 | -0.7833872 | 0.497453203133461 | 0.149756206199024 | -1.758395478 | 0.191621078258938 | 0.456855925225248 |
| Bifidobacterium breve abundance in stool | GCST90032223 | CKD | T4eVfC | Weighted mode | 10 | -0.719673122 | 0.376052849630734 | 0.087931454982613 | -1.456736707 | 0.0173904635467212 | 0.486911390702981 |
| Blautia sp000436935 abundance in stool | GCST90032237 | CKD | T4eVfC | MR Egger | 12 | 0.378903376251531 | 0.221316301672059 | 0.117669452358816 | -0.054876575 | 0.812683327528766 | 1.46068189236488 |
| Blautia sp000436935 abundance in stool | GCST90032237 | CKD | T4eVfC | Weighted median | 12 | 0.279920912723064 | 0.155292590437946 | 0.0714606828877808 | -0.024452565 | 0.584294389981437 | 1.32302517374139 |
| Blautia sp000436935 abundance in stool | GCST90032237 | CKD | T4eVfC | Inverse variance weighted | 12 | 0.225520720269289 | 0.113395831537524 | 0.0467241684348551 | 0.00326489045574074 | 0.447776550082836 | 1.25297499582675 |
| Blautia sp000436935 abundance in stool | GCST90032237 | CKD | T4eVfC | Simple mode | 12 | 0.235050713561732 | 0.235067890387332 | 0.338834511811526 | -0.225682352 | 0.695783778720903 | 1.26497291838843 |
| Blautia sp000436935 abundance in stool | GCST90032237 | CKD | T4eVfC | Weighted mode | 12 | 0.314952169408405 | 0.168468268254473 | 0.0883877676091236 | -0.015245636 | 0.645149975187171 | 1.37019377221091 |
| CAG-269 sp002372935 abundance in stool | GCST90032268 | CKD | T4eVfC | MR Egger | 13 | -0.703259805 | 1.06963016871264 | 0.524391905439928 | -2.799734936 | 1.39321532540001 | 0.494969167972919 |
| CAG-269 sp002372935 abundance in stool | GCST90032268 | CKD | T4eVfC | Weighted median | 13 | -0.381168533 | 0.274766508962698 | 0.165366992951605 | -0.919710891 | 0.157373824295425 | 0.683062761116935 |
| CAG-269 sp002372935 abundance in stool | GCST90032268 | CKD | T4eVfC | Inverse variance weighted | 13 | -0.509364662 | 0.214474588190692 | 0.0175517179231502 | -0.929734855 | -0.088994469 | 0.600877217504178 |
| CAG-269 sp002372935 abundance in stool | GCST90032268 | CKD | T4eVfC | Simple mode | 13 | -0.44107757 | 0.350110301247047 | 0.2316833628492 | -1.12729376 | 0.24513862093442 | 0.64334280085171 |
| CAG-269 sp002372935 abundance in stool | GCST90032268 | CKD | T4eVfC | Weighted mode | 13 | -0.471589403 | 0.298665990406205 | 0.140323559936252 | -1.056974744 | 0.113795938027832 | 0.624009676720323 |
| CAG-274 sp000432155 abundance in stool | GCST90032271 | CKD | T4eVfC | MR Egger | 10 | 0.118453947545104 | 0.191996522710311 | 0.554410189955272 | -0.257859237 | 0.494767132057314 | 1.1257550291255 |
| CAG-274 sp000432155 abundance in stool | GCST90032271 | CKD | T4eVfC | Weighted median | 10 | 0.111700325519586 | 0.0984774756372746 | 0.256680120119398 | -0.081315527 | 0.304716177768644 | 1.11817772110359 |
| CAG-274 sp000432155 abundance in stool | GCST90032271 | CKD | T4eVfC | Inverse variance weighted | 10 | 0.141314139831727 | 0.0705678397424696 | 0.0452278760584273 | 0.00300117393648683 | 0.279627105726967 | 1.15178641314936 |
| CAG-274 sp000432155 abundance in stool | GCST90032271 | CKD | T4eVfC | Simple mode | 10 | 0.174405644881391 | 0.143836680107735 | 0.2561782967778 | -0.107514248 | 0.45632553789255 | 1.19053840369294 |
| CAG-274 sp000432155 abundance in stool | GCST90032271 | CKD | T4eVfC | Weighted mode | 10 | 0.0860328209008384 | 0.0949275095273063 | 0.38839672934444 | -0.100025098 | 0.272090739574359 | 1.08984209731755 |
| CHKCI006 sp900018345 abundance in stool | GCST90032316 | CKD | T4eVfC | MR Egger | 14 | 0.00411417297538118 | 0.643821024486555 | 0.99500636452548 | -1.257775035 | 1.26600338096903 | 1.00412264780333 |
| CHKCI006 sp900018345 abundance in stool | GCST90032316 | CKD | T4eVfC | Weighted median | 14 | 0.4448790697587 | 0.238112226080875 | 0.0617120776337527 | -0.021820893 | 0.911579032877214 | 1.56030149678668 |
| CHKCI006 sp900018345 abundance in stool | GCST90032316 | CKD | T4eVfC | Inverse variance weighted | 14 | 0.509934635464831 | 0.179609927370185 | 0.00452377474371814 | 0.157899177819269 | 0.861970093110393 | 1.66518234751843 |
| CHKCI006 sp900018345 abundance in stool | GCST90032316 | CKD | T4eVfC | Simple mode | 14 | 0.509553850060861 | 0.318169050272684 | 0.133271777399286 | -0.114057488 | 1.13316518859532 | 1.66454839109388 |
| CHKCI006 sp900018345 abundance in stool | GCST90032316 | CKD | T4eVfC | Weighted mode | 14 | 0.45055900753447 | 0.287852108470668 | 0.141534459201859 | -0.113631125 | 1.01474914013698 | 1.56918912890441 |
| Ezakiellaceae abundance in stool | GCST90032377 | CKD | T4eVfC | MR Egger | 6 | -0.349280431 | 0.646523143485818 | 0.617703266929609 | -1.616465792 | 0.917904930687416 | 0.705195344224064 |
| Ezakiellaceae abundance in stool | GCST90032377 | CKD | T4eVfC | Weighted median | 6 | -0.408716595 | 0.385982968718822 | 0.289646233506924 | -1.165243214 | 0.347810023622781 | 0.664502528934145 |
| Ezakiellaceae abundance in stool | GCST90032377 | CKD | T4eVfC | Inverse variance weighted | 6 | -0.582167132 | 0.289842170929584 | 0.0445831792901812 | -1.150257787 | -0.014076477 | 0.55868630667245 |
| Ezakiellaceae abundance in stool | GCST90032377 | CKD | T4eVfC | Simple mode | 6 | -0.394592264 | 0.492394492625702 | 0.459287549085889 | -1.35968547 | 0.570500941389412 | 0.673954778754373 |
| Ezakiellaceae abundance in stool | GCST90032377 | CKD | T4eVfC | Weighted mode | 6 | -0.400762238 | 0.445239522069286 | 0.409320483732378 | -1.273431701 | 0.471907225346116 | 0.669809297365658 |
| Actinobacteria abundance in stool | GCST90032428 | CKD | T4eVfC | MR Egger | 11 | -0.273427484 | 0.431741109427093 | 0.54228536774406 | -1.119640058 | 0.572785090793028 | 0.760767502412171 |
| Actinobacteria abundance in stool | GCST90032428 | CKD | T4eVfC | Weighted median | 11 | -0.332931137 | 0.305880803229098 | 0.276403455668047 | -0.932457512 | 0.266595237027153 | 0.716819554584728 |
| Actinobacteria abundance in stool | GCST90032428 | CKD | T4eVfC | Inverse variance weighted | 11 | -0.48742289 | 0.237156686564735 | 0.0398521735302002 | -0.952249995 | -0.022595784 | 0.614207236123295 |
| Actinobacteria abundance in stool | GCST90032428 | CKD | T4eVfC | Simple mode | 11 | -0.129950487 | 0.596689767112295 | 0.831975785959844 | -1.299462431 | 1.03956145634842 | 0.878138908967669 |
| Actinobacteria abundance in stool | GCST90032428 | CKD | T4eVfC | Weighted mode | 11 | -0.293057381 | 0.308022853996748 | 0.363822854435695 | -0.896782174 | 0.310667413216649 | 0.745979334714639 |
| K10 abundance in stool | GCST90032440 | CKD | T4eVfC | MR Egger | 3 | -0.765235908 | 1.03965242846703 | 0.596056476889043 | -2.802954668 | 1.27248285206459 | 0.465224168050068 |
| K10 abundance in stool | GCST90032440 | CKD | T4eVfC | Weighted median | 3 | -0.839540284 | 0.423849263927807 | 0.0476190904285389 | -1.670284841 | -0.008795726 | 0.431909033452623 |
| K10 abundance in stool | GCST90032440 | CKD | T4eVfC | Inverse variance weighted | 3 | -0.781958583 | 0.332518867245937 | 0.0186917740501602 | -1.433695563 | -0.130221604 | 0.457509063528867 |
| K10 abundance in stool | GCST90032440 | CKD | T4eVfC | Simple mode | 3 | -0.841055646 | 0.452914600815778 | 0.204437734172049 | -1.728768264 | 0.0466569711491328 | 0.431255030209884 |
| K10 abundance in stool | GCST90032440 | CKD | T4eVfC | Weighted mode | 3 | -0.842031369 | 0.462843536293411 | 0.210487101001635 | -1.7492047 | 0.065141962389635 | 0.430834450279768 |
| Paenibacillus J abundance in stool | GCST90032497 | CKD | T4eVfC | MR Egger | 12 | 0.859335757278623 | 0.592699438367886 | 0.177723602478182 | -0.302355142 | 2.02102665647968 | 2.36159150263513 |
| Paenibacillus J abundance in stool | GCST90032497 | CKD | T4eVfC | Weighted median | 12 | 0.921876520171747 | 0.351757020473529 | 0.0087729765147491 | 0.232432760043629 | 1.61132028029986 | 2.51400354475178 |
| Paenibacillus J abundance in stool | GCST90032497 | CKD | T4eVfC | Inverse variance weighted | 12 | 0.906865486161902 | 0.265187183235576 | 0.000626859936234487 | 0.387098607020173 | 1.42663236530363 | 2.47654758173824 |
| Paenibacillus J abundance in stool | GCST90032497 | CKD | T4eVfC | Simple mode | 12 | 0.569309861888994 | 0.505091272674073 | 0.283669268877634 | -0.420669033 | 1.55928875633018 | 1.76704712395875 |
| Paenibacillus J abundance in stool | GCST90032497 | CKD | T4eVfC | Weighted mode | 12 | 0.702667185538334 | 0.432102304685395 | 0.132195883535528 | -0.144253332 | 1.54958770272171 | 2.01913092873828 |
| Parabacteroides johnsonii abundance in stool | GCST90032499 | CKD | T4eVfC | MR Egger | 15 | -0.551690007 | 0.52665535282691 | 0.31393908541532 | -1.583934499 | 0.480554484478892 | 0.575975584582724 |
| Parabacteroides johnsonii abundance in stool | GCST90032499 | CKD | T4eVfC | Weighted median | 15 | -0.384821494 | 0.194498963452783 | 0.0478692604326139 | -0.766039463 | -0.003603526 | 0.680572111311501 |
| Parabacteroides johnsonii abundance in stool | GCST90032499 | CKD | T4eVfC | Inverse variance weighted | 15 | -0.478599416 | 0.138366209761571 | 0.000542320273434209 | -0.749797187 | -0.207401645 | 0.619650657201748 |
| Parabacteroides johnsonii abundance in stool | GCST90032499 | CKD | T4eVfC | Simple mode | 15 | -0.440122773 | 0.260222840833313 | 0.112903644461775 | -0.950159541 | 0.0699139952442267 | 0.643957355789097 |
| Parabacteroides johnsonii abundance in stool | GCST90032499 | CKD | T4eVfC | Weighted mode | 15 | -0.364752985 | 0.207261380661886 | 0.100251937400302 | -0.770985291 | 0.0414793209700036 | 0.694368148955745 |
| Prevotella sp002437565 abundance in stool | GCST90032521 | CKD | T4eVfC | MR Egger | 13 | -0.018538752 | 0.369874019321425 | 0.960923869430327 | -0.74349183 | 0.706414325550649 | 0.981632033336669 |
| Prevotella sp002437565 abundance in stool | GCST90032521 | CKD | T4eVfC | Weighted median | 13 | -0.174868439 | 0.198114997344268 | 0.377419301949288 | -0.563173834 | 0.213436955302071 | 0.839567467425771 |
| Prevotella sp002437565 abundance in stool | GCST90032521 | CKD | T4eVfC | Inverse variance weighted | 13 | -0.290152176 | 0.134865649269134 | 0.0314433440199539 | -0.554488849 | -0.025815504 | 0.748149708308887 |
| Prevotella sp002437565 abundance in stool | GCST90032521 | CKD | T4eVfC | Simple mode | 13 | -0.279893715 | 0.297784896589753 | 0.365794634237262 | -0.863552112 | 0.303764682268505 | 0.755864074163857 |
| Prevotella sp002437565 abundance in stool | GCST90032521 | CKD | T4eVfC | Weighted mode | 13 | -0.152020916 | 0.221099770866966 | 0.504798296793034 | -0.585376467 | 0.28133463511616 | 0.858970314516453 |
| UBA737 sp002451855 abundance in stool | GCST90032628 | CKD | T4eVfC | MR Egger | 8 | -0.793777377 | 1.05915740936313 | 0.481929847944987 | -2.869725899 | 1.28217114583243 | 0.452133686395108 |
| UBA737 sp002451855 abundance in stool | GCST90032628 | CKD | T4eVfC | Weighted median | 8 | -0.539200971 | 0.358430908941223 | 0.132494549949686 | -1.241725553 | 0.163323610208093 | 0.583214071019223 |
| UBA737 sp002451855 abundance in stool | GCST90032628 | CKD | T4eVfC | Inverse variance weighted | 8 | -0.648199982 | 0.265128633586752 | 0.0144912031467805 | -1.167852103 | -0.12854786 | 0.522986315018936 |
| UBA737 sp002451855 abundance in stool | GCST90032628 | CKD | T4eVfC | Simple mode | 8 | -0.559460056 | 0.531689608166583 | 0.327655118922959 | -1.601571688 | 0.482651576163161 | 0.571517568125116 |
| UBA737 sp002451855 abundance in stool | GCST90032628 | CKD | T4eVfC | Weighted mode | 8 | -0.529543318 | 0.473817193833279 | 0.30061757969571 | -1.458225018 | 0.399138381789351 | 0.588873836288785 |

**Supplementary Table 7 :MR results of causal links between lipid and CKD.**

| exposure | id.exposure | outcome | id.outcome | method | nsnp | b | se | pval | lo_ci | up_ci | or |
| --- | --- | --- | --- | --- | --- | --- | --- | --- | --- | --- | --- |
| Phosphatidylcholine (14:0_18:2) levels | GCST90277274 | CKD | WoOryb | MR Egger | 16 | -0.106036882 | 0.320196804657608 | 0.74542712857351 | -0.733622619 | 0.52154885523703 | 0.899391476205023 |
| Phosphatidylcholine (14:0_18:2) levels | GCST90277274 | CKD | WoOryb | Weighted median | 16 | -0.169362906 | 0.119354984732932 | 0.155903456190555 | -0.403298676 | 0.06457286447488 | 0.844202481978724 |
| Phosphatidylcholine (14:0_18:2) levels | GCST90277274 | CKD | WoOryb | Inverse variance weighted | 16 | -0.178630137 | 0.088116742637681 | 0.0426421071875599 | -0.351338952 | -0.005921321 | 0.836415201619282 |
| Phosphatidylcholine (14:0_18:2) levels | GCST90277274 | CKD | WoOryb | Simple mode | 16 | -0.126003058 | 0.176135180771184 | 0.485363268459164 | -0.471228012 | 0.219221896229226 | 0.881612150736784 |
| Phosphatidylcholine (14:0_18:2) levels | GCST90277274 | CKD | WoOryb | Weighted mode | 16 | -0.170593808 | 0.145378169533175 | 0.2589127368224 | -0.455535021 | 0.114347403840727 | 0.843163990016376 |
| Phosphatidylcholine (18:2_20:1) levels | GCST90277315 | CKD | WoOryb | MR Egger | 11 | -0.197952381 | 0.155217880238314 | 0.234130405738338 | -0.502179426 | 0.10627466408839 | 0.820408919113393 |
| Phosphatidylcholine (18:2_20:1) levels | GCST90277315 | CKD | WoOryb | Weighted median | 11 | -0.173841355 | 0.0889402601777257 | 0.0506319981644284 | -0.348164265 | 0.000481555004052447 | 0.840430217181536 |
| Phosphatidylcholine (18:2_20:1) levels | GCST90277315 | CKD | WoOryb | Inverse variance weighted | 11 | -0.156782063 | 0.0724085149202193 | 0.030369045602909 | -0.298702753 | -0.014861374 | 0.85489035032863 |
| Phosphatidylcholine (18:2_20:1) levels | GCST90277315 | CKD | WoOryb | Simple mode | 11 | -0.123905179 | 0.159927431678418 | 0.456410553040615 | -0.437362945 | 0.189552587465555 | 0.883463608146068 |
| Phosphatidylcholine (18:2_20:1) levels | GCST90277315 | CKD | WoOryb | Weighted mode | 11 | -0.166572588 | 0.0911939463336382 | 0.0977214421845469 | -0.345312722 | 0.0121675471647296 | 0.846561364805758 |
| Phosphatidylcholine (O-18:0_16:1) levels | GCST90277335 | CKD | WoOryb | MR Egger | 12 | 0.0302818664192285 | 0.299000504923767 | 0.921332473450071 | -0.555759123 | 0.616322856069812 | 1.03074502542115 |
| Phosphatidylcholine (O-18:0_16:1) levels | GCST90277335 | CKD | WoOryb | Weighted median | 12 | 0.110916687253867 | 0.136734823977917 | 0.417261751897183 | -0.157083568 | 0.378916942250584 | 1.11730181749407 |
| Phosphatidylcholine (O-18:0_16:1) levels | GCST90277335 | CKD | WoOryb | Inverse variance weighted | 12 | 0.190188674571861 | 0.0948890540270359 | 0.0450350642190045 | 0.00420612867887044 | 0.376171220464851 | 1.20947777383221 |
| Phosphatidylcholine (O-18:0_16:1) levels | GCST90277335 | CKD | WoOryb | Simple mode | 12 | 0.121611793119959 | 0.199160018298655 | 0.553855864799456 | -0.268741843 | 0.511965428985323 | 1.1293156085838 |
| Phosphatidylcholine (O-18:0_16:1) levels | GCST90277335 | CKD | WoOryb | Weighted mode | 12 | 0.0671943593822749 | 0.156466495487438 | 0.675887430453443 | -0.239479972 | 0.373868690537653 | 1.06950332598114 |
| Phosphatidylcholine (O-18:1_20:3) levels | GCST90277339 | CKD | WoOryb | MR Egger | 10 | 0.028157977570111 | 0.212003371334474 | 0.897616938119643 | -0.38736863 | 0.443684585385679 | 1.02855816070623 |
| Phosphatidylcholine (O-18:1_20:3) levels | GCST90277339 | CKD | WoOryb | Weighted median | 10 | -0.162174404 | 0.112172595178033 | 0.148245147060787 | -0.382032691 | 0.0576838824657705 | 0.850292897054753 |
| Phosphatidylcholine (O-18:1_20:3) levels | GCST90277339 | CKD | WoOryb | Inverse variance weighted | 10 | -0.183882129 | 0.0910132434606641 | 0.0433430840540934 | -0.362268086 | -0.005496172 | 0.832033870601541 |
| Phosphatidylcholine (O-18:1_20:3) levels | GCST90277339 | CKD | WoOryb | Simple mode | 10 | -0.215273075 | 0.177265522445498 | 0.25549261835266 | -0.562713499 | 0.132167348895628 | 0.806321223864037 |
| Phosphatidylcholine (O-18:1_20:3) levels | GCST90277339 | CKD | WoOryb | Weighted mode | 10 | -0.150172903 | 0.11554061508691 | 0.225991326399439 | -0.376632508 | 0.0762867027063777 | 0.860559170415759 |
| Phosphatidylcholine (O-18:2_20:4) levels | GCST90277344 | CKD | WoOryb | MR Egger | 11 | 0.566976936251342 | 0.32404738871999 | 0.114102423323899 | -0.068155946 | 1.20210981814252 | 1.76292953929725 |
| Phosphatidylcholine (O-18:2_20:4) levels | GCST90277344 | CKD | WoOryb | Weighted median | 11 | 0.238264674202377 | 0.12735589819099 | 0.0613649038557206 | -0.011352886 | 0.487882234656716 | 1.26904503186057 |
| Phosphatidylcholine (O-18:2_20:4) levels | GCST90277344 | CKD | WoOryb | Inverse variance weighted | 11 | 0.217184623194246 | 0.0980863000124225 | 0.0268136655424062 | 0.024935475169898 | 0.409433771218594 | 1.24257348884866 |
| Phosphatidylcholine (O-18:2_20:4) levels | GCST90277344 | CKD | WoOryb | Simple mode | 11 | 0.200839335613327 | 0.175017599463759 | 0.277871824041633 | -0.142195159 | 0.543873830562293 | 1.22242835534322 |
| Phosphatidylcholine (O-18:2_20:4) levels | GCST90277344 | CKD | WoOryb | Weighted mode | 11 | 0.238645752924378 | 0.13850478091379 | 0.115609568636902 | -0.032823618 | 0.510115123515407 | 1.26952873007717 |
| Sphingomyelin (d32:1) levels | GCST90277368 | CKD | WoOryb | MR Egger | 16 | 0.17910053279049 | 0.111229910308769 | 0.129667275866391 | -0.038910091 | 0.397111156995677 | 1.19614098951498 |
| Sphingomyelin (d32:1) levels | GCST90277368 | CKD | WoOryb | Weighted median | 16 | 0.0723303045856103 | 0.0983809756004515 | 0.462213915243249 | -0.120496408 | 0.265157016762495 | 1.07501036628141 |
| Sphingomyelin (d32:1) levels | GCST90277368 | CKD | WoOryb | Inverse variance weighted | 16 | 0.151685377225052 | 0.0634216262178193 | 0.0167706349641056 | 0.0273789898381265 | 0.275991764611978 | 1.16379402272065 |
| Sphingomyelin (d32:1) levels | GCST90277368 | CKD | WoOryb | Simple mode | 16 | 0.272160613247181 | 0.165608251880746 | 0.121091418406782 | -0.05243156 | 0.596752786933443 | 1.31279783710265 |
| Sphingomyelin (d32:1) levels | GCST90277368 | CKD | WoOryb | Weighted mode | 16 | 0.174824051371745 | 0.0904327878908778 | 0.0723150317704829 | -0.002424213 | 0.352072315637866 | 1.1910366369128 |
| Triacylglycerol (54:6) levels | GCST90277407 | CKD | WoOryb | MR Egger | 13 | 0.0863056394551242 | 0.261486684460397 | 0.747556887596202 | -0.426208262 | 0.598819540997503 | 1.09013946702508 |
| Triacylglycerol (54:6) levels | GCST90277407 | CKD | WoOryb | Weighted median | 13 | 0.130703593602069 | 0.138099718896739 | 0.34392238173599 | -0.139971855 | 0.401379042639677 | 1.13962993764016 |
| Triacylglycerol (54:6) levels | GCST90277407 | CKD | WoOryb | Inverse variance weighted | 13 | 0.186732638229205 | 0.0937408034772743 | 0.0463699369416221 | 0.00300066341374741 | 0.370464613044663 | 1.20530498949075 |
| Triacylglycerol (54:6) levels | GCST90277407 | CKD | WoOryb | Simple mode | 13 | 0.108730151756073 | 0.202320472176285 | 0.600804623998141 | -0.287817974 | 0.505278277221591 | 1.11486146633785 |
| Triacylglycerol (54:6) levels | GCST90277407 | CKD | WoOryb | Weighted mode | 13 | 0.0832018886094089 | 0.138874185961979 | 0.560235780143605 | -0.188991516 | 0.355395293094887 | 1.08676119110771 |

**Supplementary Table 8:MR results of causal links between genus Herbidospora and phosphatidylcholine.**

| exposure | id.exposure | outcome | id.outcome | method | nsnp | b | se | pval | lo_ci | up_ci | or |
| --- | --- | --- | --- | --- | --- | --- | --- | --- | --- | --- | --- |
| Herbidospora abundance in stool | GCST90032428 | Phosphatidylcholine (14:0_18:2) levels | GCST90277274 | MR Egger | 23 | 0.521868285437355 | 0.4516264642889 | 0.26085176265196 | -0.363319585 | 1.4070561554436 | 1.6851730948418 |
| Herbidospora abundance in stool | GCST90032428 | Phosphatidylcholine (14:0_18:2) levels | GCST90277274 | Weighted median | 23 | 0.106229730193515 | 0.221521392392561 | 0.631550147205903 | -0.327952199 | 0.540411659282935 | 1.11207732490236 |
| Herbidospora abundance in stool | GCST90032428 | Phosphatidylcholine (14:0_18:2) levels | GCST90277274 | Inverse variance weighted | 23 | 0.344114284174393 | 0.172269582880162 | 0.0457672456233528 | 0.00646590172927447 | 0.681762666619511 | 1.4107398515345 |
| Herbidospora abundance in stool | GCST90032428 | Phosphatidylcholine (14:0_18:2) levels | GCST90277274 | Simple mode | 23 | -0.282845209 | 0.474110948454877 | 0.556877479746263 | -1.212102668 | 0.64641225041327 | 0.753636435301818 |
| Herbidospora abundance in stool | GCST90032428 | Phosphatidylcholine (14:0_18:2) levels | GCST90277274 | Weighted mode | 23 | -0.304640006 | 0.46826396317191 | 0.522059030999544 | -1.222437374 | 0.613157362114301 | 0.737388782372672 |

**Supplementary Table 9: The ids of the data used in this study.**

| data | id |
| --- | --- |
| Bifidobacterium breve | ebi-a-GCST90032223 |
| Blautia sp000436935 | ebi-a-GCST90032237 |
| CAG-269 sp002372935 | ebi-a-GCST90032268 |
| Eubacterium CAG-274 sp000432155 | ebi-a-GCST90032271 |
| Clostridium sp900018345 | ebi-a-GCST90032316 |
| Fenollaria | ebi-a-GCST90032377 |
| Actinbacteria | ebi-a-GCST90032428 |
| K10 | ebi-a-GCST90032440 |
| Paenibacillus J | ebi-a-GCST90032497 |
| Parabacteroides johnsonii | ebi-a-GCST90032499 |
| Prevotella sp002437565 | ebi-a-GCST90032521 |
| Rumenococcaceae UBA737 sp002451855 | ebi-a-GCST90032628 |
| Phosphatidylcholine (14:0_18:2) | ebi-a-GCST90277274 |
| Phosphatidylcholine (18:2_20:1) | ebi-a-GCST90277315 |
| Phosphatidylcholine (O-18:0_16:1) | ebi-a-GCST90277335 |
| Phosphatidylcholine (O-18:1_20:3) | ebi-a-GCST90277339 |
| Phosphatidylcholine (O-18:2_20:4) | ebi-a-GCST90277344 |
| Sphingomyelin (d32:1) | ebi-a-GCST90277368 |
| Triacylglycerol (54:6) | ebi-a-GCST90277407 |
| Chronic kidney disease | ebi-a-GCST90018602 |

**Supplementary Table 10: Single-nucleotide polymorphisms associated with exposure and outcome.**

| exposure | id.exposure | outcome | id.outcome | SNP | effect_allele.exposure | other_allele.exposure | effect_allele.outcome | other_allele.outcome | beta.exposure | se.exposure | pval.exposure | beta.outcome | se.outcome | pval.outcome |
| --- | --- | --- | --- | --- | --- | --- | --- | --- | --- | --- | --- | --- | --- | --- |
| Megamonas abundance in stool | GCST90032472 | CKD | T4eVfC | rs10025256 | C | A | C | A | 0.0658971 | 0.0147556 | 8e-06 | -0.07385492 | 0.0392058436077086 | 0.0595956497289246 |
| Megamonas abundance in stool | GCST90032472 | CKD | T4eVfC | rs10196495 | T | C | T | C | 0.0514484 | 0.0107683 | 1.8e-06 | -0.002380083 | 0.0367159272346668 | 0.948313924468744 |
| Megamonas abundance in stool | GCST90032472 | CKD | T4eVfC | rs112799669 | T | A | T | A | 0.109997 | 0.022906 | 1.6e-06 | 0.0467365865700673 | 0.0494728766304048 | 0.344816598714992 |
| Megamonas abundance in stool | GCST90032472 | CKD | T4eVfC | rs114347138 | T | A | T | A | 0.225993 | 0.0509299 | 9.1e-06 | -0.054251007 | 0.372144200565919 | 0.884095435247718 |
| Megamonas abundance in stool | GCST90032472 | CKD | T4eVfC | rs17164298 | C | T | C | T | 0.0484566 | 0.0109107 | 8.9e-06 | -0.019125993 | 0.0344766295592328 | 0.579064036978427 |
| Megamonas abundance in stool | GCST90032472 | CKD | T4eVfC | rs1999144 | C | T | C | T | 0.115859 | 0.0229254 | 4.3e-07 | 0.00510037226532482 | 0.0558884115609813 | 0.927286060027097 |
| Megamonas abundance in stool | GCST90032472 | CKD | T4eVfC | rs2191523 | C | T | C | T | 0.182006 | 0.0372802 | 1e-06 | 0.00132020208972347 | 0.03133964788446 | 0.966398556691763 |
| Megamonas abundance in stool | GCST90032472 | CKD | T4eVfC | rs2281616 | G | A | G | A | 0.222881 | 0.0471905 | 2.3e-06 | 0.16937713983363 | 0.125220585496699 | 0.176173814206502 |
| Megamonas abundance in stool | GCST90032472 | CKD | T4eVfC | rs2451340 | A | G | A | G | 0.146928 | 0.031252 | 2.6e-06 | -0.038074453 | 0.163793320193307 | 0.816185217703847 |
| Megamonas abundance in stool | GCST90032472 | CKD | T4eVfC | rs35481789 | T | C | T | C | 0.0548941 | 0.0121205 | 5.9e-06 | -0.020075828 | 0.032561966811972 | 0.537536711461231 |
| Megamonas abundance in stool | GCST90032472 | CKD | T4eVfC | rs35703110 | A | C | A | C | 0.0626145 | 0.0138911 | 6.6e-06 | 0.00511320025703635 | 0.0335883149515756 | 0.879004333895589 |
| Megamonas abundance in stool | GCST90032472 | CKD | T4eVfC | rs3783925 | G | A | G | A | -0.0497877 | 0.0111333 | 7.7e-06 | 0.0381744172379253 | 0.0401524818226252 | 0.341738316371279 |
| Megamonas abundance in stool | GCST90032472 | CKD | T4eVfC | rs535920 | T | C | T | C | -0.0524622 | 0.0113105 | 3.5e-06 | 0.0305694699318803 | 0.0469697374300011 | 0.515154091788735 |
| Megamonas abundance in stool | GCST90032472 | CKD | T4eVfC | rs58277973 | A | G | A | G | 0.126665 | 0.0270707 | 2.9e-06 | 0.0243683825395785 | 0.0457593035706181 | 0.594356236652894 |
| Megamonas abundance in stool | GCST90032472 | CKD | T4eVfC | rs6976718 | C | T | C | T | 0.0479094 | 0.010709 | 7.7e-06 | 0.113735419621315 | 0.0831971733277952 | 0.171606907078546 |
| Megamonas abundance in stool | GCST90032472 | CKD | T4eVfC | rs75824665 | C | G | C | G | 0.168146 | 0.0370934 | 5.8e-06 | 0.0399884760277882 | 0.0392114918912868 | 0.307816105930032 |
| Megamonas abundance in stool | GCST90032472 | CKD | T4eVfC | rs9826649 | C | A | C | A | 0.0548926 | 0.0120325 | 5.1e-06 | 0.0192786593513493 | 0.0609104646076598 | 0.751616829095918 |
| Gramella abundance in stool | GCST90032422 | CKD | T4eVfC | rs10005003 | T | C | T | C | -0.0317444 | 0.0067549 | 2.6e-06 | -0.071591343 | 0.0372326592230777 | 0.0545038714525496 |
| Gramella abundance in stool | GCST90032422 | CKD | T4eVfC | rs10792502 | A | G | A | G | -0.0460333 | 0.00977495 | 2.5e-06 | -0.031141788 | 0.0426073749967013 | 0.464839416439876 |
| Gramella abundance in stool | GCST90032422 | CKD | T4eVfC | rs11183780 | C | G | C | G | -0.0904016 | 0.0197893 | 4.9e-06 | 0.0131019359043192 | 0.0610051584347229 | 0.829948458148335 |
| Gramella abundance in stool | GCST90032422 | CKD | T4eVfC | rs142663429 | A | G | A | G | 0.102664 | 0.0211856 | 1.3e-06 | 1.03996606974913 | 0.461444146071075 | 0.0242137653121752 |
| Gramella abundance in stool | GCST90032422 | CKD | T4eVfC | rs17040530 | C | T | C | T | 0.0804913 | 0.0167421 | 1.5e-06 | 0.0572286586324033 | 0.145465340117408 | 0.694010901833038 |
| Gramella abundance in stool | GCST90032422 | CKD | T4eVfC | rs17143400 | C | T | C | T | 0.0324095 | 0.00673947 | 1.5e-06 | -0.050940936 | 0.0381639847665836 | 0.18194482522821 |
| Gramella abundance in stool | GCST90032422 | CKD | T4eVfC | rs3800857 | A | G | A | G | 0.0317051 | 0.0061141 | 2.2e-07 | -0.008372664 | 0.0640293091429547 | 0.895962805792957 |
| Gramella abundance in stool | GCST90032422 | CKD | T4eVfC | rs62438218 | C | T | C | T | 0.0695289 | 0.0157238 | 9.8e-06 | -0.083980426 | 0.072010028423739 | 0.243520511345492 |
| Gramella abundance in stool | GCST90032422 | CKD | T4eVfC | rs6911413 | T | G | T | G | -0.0343118 | 0.00656443 | 1.7e-07 | 0.0436988334765563 | 0.0386200264268865 | 0.257841723610728 |
| Gramella abundance in stool | GCST90032422 | CKD | T4eVfC | rs7020426 | G | A | G | A | -0.0312981 | 0.00631361 | 7.2e-07 | 0.0347575575409908 | 0.0337039443491301 | 0.302418522724789 |
| Gramella abundance in stool | GCST90032422 | CKD | T4eVfC | rs72696728 | C | A | C | A | 0.0314466 | 0.0066313 | 2.1e-06 | 0.108277766502106 | 0.403166942156526 | 0.788262088205336 |
| Gramella abundance in stool | GCST90032422 | CKD | T4eVfC | rs7907281 | A | T | A | T | 0.0726378 | 0.0142741 | 3.6e-07 | -0.034852437 | 0.0365961546802315 | 0.340918269810491 |
| Gramella abundance in stool | GCST90032422 | CKD | T4eVfC | rs9306374 | C | T | C | T | 0.0264559 | 0.00597542 | 9.5e-06 | 0.0378981386001735 | 0.0354055258953506 | 0.284438490036952 |
| UBA1446 sp002329245 abundance in stool | GCST90032603 | CKD | T4eVfC | rs10006312 | C | G | C | G | -0.0435817 | 0.00971985 | 7.3e-06 | 0.0488074657336574 | 0.0415064511307647 | 0.239634551476434 |
| UBA1446 sp002329245 abundance in stool | GCST90032603 | CKD | T4eVfC | rs10960848 | C | T | C | T | 0.0525611 | 0.0114895 | 4.8e-06 | 0.0738821559329844 | 0.0379448771104547 | 0.0515237398413214 |
| UBA1446 sp002329245 abundance in stool | GCST90032603 | CKD | T4eVfC | rs117196811 | T | C | T | C | 0.0770433 | 0.0168423 | 4.8e-06 | 0.0126672571285784 | 0.0436611258975653 | 0.771719332029015 |
| UBA1446 sp002329245 abundance in stool | GCST90032603 | CKD | T4eVfC | rs13296844 | C | A | C | A | 0.0330045 | 0.0072229 | 4.9e-06 | 0.00333473112999 | 0.0736683446321606 | 0.96389464024593 |
| UBA1446 sp002329245 abundance in stool | GCST90032603 | CKD | T4eVfC | rs2107160 | G | C | G | C | 0.0286974 | 0.00649491 | 9.9e-06 | 0.0186788790789053 | 0.0334174453433821 | 0.576191800445114 |
| UBA1446 sp002329245 abundance in stool | GCST90032603 | CKD | T4eVfC | rs2902051 | G | A | G | A | -0.0376642 | 0.00840478 | 7.4e-06 | 0.261926002348715 | 0.432614724796324 | 0.54488093219557 |
| UBA1446 sp002329245 abundance in stool | GCST90032603 | CKD | T4eVfC | rs62479642 | C | T | C | T | 0.0290726 | 0.00657189 | 9.7e-06 | -0.006620724 | 0.0352780955069641 | 0.851133489934488 |
| UBA1446 sp002329245 abundance in stool | GCST90032603 | CKD | T4eVfC | rs7078392 | G | A | G | A | 0.0512139 | 0.0114578 | 7.8e-06 | -0.009122347 | 0.0352021326304688 | 0.795525775215744 |
| UBA1446 sp002329245 abundance in stool | GCST90032603 | CKD | T4eVfC | rs72995423 | T | C | T | C | -0.0485665 | 0.0109899 | 9.9e-06 | -0.434787706 | 0.297096822656723 | 0.143343032934219 |
| CAG-145 abundance in stool | GCST90032255 | CKD | T4eVfC | rs12576787 | T | C | T | C | -0.105365 | 0.0193397 | 5.1e-08 | -0.016454792 | 0.0369262895342505 | 0.65587765509704 |
| CAG-145 abundance in stool | GCST90032255 | CKD | T4eVfC | rs3104762 | G | A | G | A | -0.102875 | 0.0231401 | 8.8e-06 | 0.0363789327682618 | 0.0434935021704462 | 0.402917402013587 |
| CAG-145 abundance in stool | GCST90032255 | CKD | T4eVfC | rs343435 | T | C | T | C | 0.0599192 | 0.013204 | 5.7e-06 | 0.0090547166940339 | 0.0412282918787713 | 0.826164105769204 |
| CAG-145 abundance in stool | GCST90032255 | CKD | T4eVfC | rs4140764 | G | C | G | C | -0.0464622 | 0.0105029 | 9.7e-06 | 0.0334260993606731 | 0.0401949206358619 | 0.405634714904541 |
| CAG-145 abundance in stool | GCST90032255 | CKD | T4eVfC | rs74620412 | A | G | A | G | -0.174002 | 0.0357546 | 1.1e-06 | -0.041810852 | 0.0828376332866593 | 0.61374667991149 |
| Eisenbergiella sp900066775 abundance in stool | GCST90032351 | CKD | T4eVfC | rs10016465 | G | A | G | A | -0.0606854 | 0.0128832 | 2.5e-06 | 0.01122646447842 | 0.0336440279127886 | 0.738618233720221 |
| Eisenbergiella sp900066775 abundance in stool | GCST90032351 | CKD | T4eVfC | rs112922627 | C | G | C | G | -0.105757 | 0.0232389 | 5.3e-06 | 0.160369395567359 | 0.191408389121268 | 0.402121184041619 |
| Eisenbergiella sp900066775 abundance in stool | GCST90032351 | CKD | T4eVfC | rs1656195 | C | G | C | G | 0.0609139 | 0.0127487 | 1.8e-06 | -0.024912141 | 0.0317661404596142 | 0.432901822198795 |
| Eisenbergiella sp900066775 abundance in stool | GCST90032351 | CKD | T4eVfC | rs2163950 | A | C | A | C | 0.172452 | 0.0384493 | 7.3e-06 | 0.0561591726324847 | 0.0409425502506134 | 0.170169959091583 |
| Eisenbergiella sp900066775 abundance in stool | GCST90032351 | CKD | T4eVfC | rs4744630 | T | C | T | C | 0.0523367 | 0.0117164 | 7.9e-06 | 0.0138254451530068 | 0.0313227774917619 | 0.658933351922172 |
| Phosphatidylethanolamine (18:1_0:0) levels | GCST90277270 | CKD | WoOryb | rs10015043 | G | A | G | A | 0.0886368 | 0.0198204 | 7.91e-06 | 0.00952246774400396 | 0.0366042010723545 | 0.79475049764025 |
| Phosphatidylethanolamine (18:1_0:0) levels | GCST90277270 | CKD | WoOryb | rs11917269 | T | G | T | G | 0.108543 | 0.0218194 | 6.75e-07 | 0.00829994013053416 | 0.0430272125157568 | 0.847037451831031 |
| Phosphatidylethanolamine (18:1_0:0) levels | GCST90277270 | CKD | WoOryb | rs192233385 | T | C | T | C | -0.914193 | 0.205935 | 9.2e-06 | 0.0182168370747923 | 0.0379038677808519 | 0.630796347826314 |
| Phosphatidylethanolamine (18:1_0:0) levels | GCST90277270 | CKD | WoOryb | rs2296637 | T | C | T | C | -0.145066 | 0.0322811 | 7.14e-06 | -0.0142907 | 0.0398419695592475 | 0.719831059188448 |
| Phosphatidylethanolamine (18:1_0:0) levels | GCST90277270 | CKD | WoOryb | rs35584640 | A | G | A | G | -0.108067 | 0.0236268 | 4.89e-06 | -0.001341835 | 0.0862141399930189 | 0.987582246467068 |
| Phosphatidylethanolamine (18:1_0:0) levels | GCST90277270 | CKD | WoOryb | rs6434115 | A | G | A | G | 0.0933379 | 0.0207398 | 6.92e-06 | -0.011214985 | 0.0570253525916104 | 0.844088409711151 |
| Phosphatidylethanolamine (18:1_0:0) levels | GCST90277270 | CKD | WoOryb | rs6768813 | C | T | C | T | 0.109509 | 0.0203534 | 7.74e-08 | 0.0210642799056231 | 0.0317902084452186 | 0.507585001079708 |
| Phosphatidylethanolamine (18:1_0:0) levels | GCST90277270 | CKD | WoOryb | rs7776911 | A | G | A | G | -0.137228 | 0.026822 | 3.22e-07 | 0.0174234357768352 | 0.0381034013171244 | 0.647478999514077 |
| Phosphatidylethanolamine (18:1_0:0) levels | GCST90277270 | CKD | WoOryb | rs9826895 | A | G | A | G | -0.103052 | 0.0220214 | 2.95e-06 | -0.014396106 | 0.0413220276020274 | 0.727548676501872 |
| Sterol ester (27:1/17:0) levels | GCST90277242 | CKD | WoOryb | rs1002079 | G | C | G | C | -0.142387 | 0.0280342 | 3.89e-07 | 0.0339019594705519 | 0.0381083384377292 | 0.373669689542799 |
| Sterol ester (27:1/17:0) levels | GCST90277242 | CKD | WoOryb | rs10241012 | G | A | G | A | 0.118223 | 0.025873 | 4.98e-06 | -0.036361503 | 0.0341981538064022 | 0.287664405929594 |
| Sterol ester (27:1/17:0) levels | GCST90277242 | CKD | WoOryb | rs11265455 | G | A | G | A | 0.119264 | 0.025622 | 3.3e-06 | -0.048674859 | 0.0364822449497656 | 0.182136091504855 |
| Sterol ester (27:1/17:0) levels | GCST90277242 | CKD | WoOryb | rs12296850 | G | A | G | A | 0.173088 | 0.0376135 | 4.26e-06 | 0.00674148824218092 | 0.0433633198487412 | 0.876454567645996 |
| Sterol ester (27:1/17:0) levels | GCST90277242 | CKD | WoOryb | rs12735602 | A | C | A | C | 0.0759366 | 0.0170987 | 9.09e-06 | -0.007835799 | 0.0327318512555063 | 0.81080031469428 |
| Sterol ester (27:1/17:0) levels | GCST90277242 | CKD | WoOryb | rs12921107 | C | T | C | T | -0.125335 | 0.024234 | 2.38e-07 | -0.001440707 | 0.0542218314191061 | 0.978802213316575 |
| Sterol ester (27:1/17:0) levels | GCST90277242 | CKD | WoOryb | rs13125535 | G | T | G | T | 0.0860677 | 0.0171841 | 5.61e-07 | -0.044607325 | 0.0343067724934227 | 0.193515856437849 |
| Sterol ester (27:1/17:0) levels | GCST90277242 | CKD | WoOryb | rs17791425 | A | G | A | G | 0.180099 | 0.0393889 | 4.9e-06 | -0.045626926 | 0.233839949934317 | 0.845298756214483 |
| Sterol ester (27:1/17:0) levels | GCST90277242 | CKD | WoOryb | rs1868072 | C | T | C | T | -0.082947 | 0.0186157 | 8.47e-06 | -0.078491543 | 0.0997021803158957 | 0.431129654378597 |
| Sterol ester (27:1/17:0) levels | GCST90277242 | CKD | WoOryb | rs2280696 | T | A | T | A | 0.203799 | 0.0251576 | 6.37e-16 | -0.000951764 | 0.0399780141518115 | 0.981006405954966 |
| Sterol ester (27:1/17:0) levels | GCST90277242 | CKD | WoOryb | rs2382335 | C | T | C | T | 0.101852 | 0.0226815 | 7.21e-06 | 0.00208286290625031 | 0.0403133280566464 | 0.958794147215509 |
| Sterol ester (27:1/17:0) levels | GCST90277242 | CKD | WoOryb | rs4246215 | T | G | T | G | -0.0845995 | 0.017008 | 6.7e-07 | -0.042556064 | 0.0319647283741239 | 0.183075696620972 |
| Sterol ester (27:1/17:0) levels | GCST90277242 | CKD | WoOryb | rs429358 | C | T | C | T | 0.121765 | 0.0216407 | 1.91e-08 | -0.013617019 | 0.0524204719123196 | 0.795044828413947 |
| Sterol ester (27:1/17:0) levels | GCST90277242 | CKD | WoOryb | rs635634 | C | T | C | T | -0.100165 | 0.0209797 | 1.84e-06 | -0.078589698 | 0.0346980015911096 | 0.0235149362991679 |
| Sterol ester (27:1/17:0) levels | GCST90277242 | CKD | WoOryb | rs79528439 | A | T | A | T | -0.131526 | 0.0265727 | 7.6e-07 | 0.0141978698903042 | 0.0350205921936382 | 0.685172560224419 |
| Sterol ester (27:1/17:0) levels | GCST90277242 | CKD | WoOryb | rs9827613 | G | A | G | A | -0.0753765 | 0.0169197 | 8.51e-06 | -0.01744764 | 0.0400085008252019 | 0.662765397699131 |
| Diacylglycerol (16:1_18:1) levels | GCST90277260 | CKD | WoOryb | rs10022344 | T | C | T | C | 0.105672 | 0.0237049 | 8.41e-06 | -0.019509926 | 0.0344429125513636 | 0.571093079067362 |
| Diacylglycerol (16:1_18:1) levels | GCST90277260 | CKD | WoOryb | rs10502551 | G | C | G | C | -0.212813 | 0.0415968 | 3.21e-07 | 0.00427478673756443 | 0.0418460616534736 | 0.918633601819225 |
| Diacylglycerol (16:1_18:1) levels | GCST90277260 | CKD | WoOryb | rs11775636 | A | G | A | G | -0.113375 | 0.0242701 | 3.05e-06 | -0.034368876 | 0.0326353099135234 | 0.292286302382711 |
| Diacylglycerol (16:1_18:1) levels | GCST90277260 | CKD | WoOryb | rs12050879 | A | G | A | G | -0.277766 | 0.0621538 | 7.99e-06 | -0.044810268 | 0.0392102910622047 | 0.253113720792559 |
| Diacylglycerol (16:1_18:1) levels | GCST90277260 | CKD | WoOryb | rs1260326 | C | T | C | T | -0.11192 | 0.0186705 | 2.15e-09 | 0.00417826564979557 | 0.031286311179199 | 0.893758960161603 |
| Diacylglycerol (16:1_18:1) levels | GCST90277260 | CKD | WoOryb | rs143808674 | G | A | G | A | -0.186449 | 0.0419048 | 8.76e-06 | -0.026099121 | 0.0629991286165867 | 0.678670901041311 |
| Diacylglycerol (16:1_18:1) levels | GCST90277260 | CKD | WoOryb | rs2182380 | G | A | G | A | 0.260746 | 0.0543597 | 1.65e-06 | -0.02207536 | 0.05402946600479 | 0.682847901127282 |
| Diacylglycerol (16:1_18:1) levels | GCST90277260 | CKD | WoOryb | rs2524075 | T | C | T | C | -0.107183 | 0.0218188 | 9.22e-07 | -0.023876197 | 0.0335432826086542 | 0.476586999870561 |
| Diacylglycerol (16:1_18:1) levels | GCST90277260 | CKD | WoOryb | rs3829088 | C | T | C | T | 0.101769 | 0.022403 | 5.65e-06 | 0.022238929470544 | 0.0414183476632048 | 0.591313031204655 |
| Diacylglycerol (16:1_18:1) levels | GCST90277260 | CKD | WoOryb | rs4788788 | A | C | A | C | -0.0932329 | 0.0210341 | 9.47e-06 | 0.0074252893326389 | 0.0342860642946857 | 0.828544458767468 |
| Diacylglycerol (16:1_18:1) levels | GCST90277260 | CKD | WoOryb | rs7955732 | T | G | T | G | -0.0886008 | 0.0182566 | 1.24e-06 | 0.0515019440533183 | 0.0722836117187173 | 0.476156318709189 |
| Diacylglycerol (16:1_18:1) levels | GCST90277260 | CKD | WoOryb | rs908150 | C | T | C | T | 0.0807078 | 0.0182166 | 9.56e-06 | 0.0144679644007325 | 0.0315098996005761 | 0.646122032286086 |
| Diacylglycerol (16:1_18:1) levels | GCST90277260 | CKD | WoOryb | rs964184 | C | G | C | G | -0.163958 | 0.0246535 | 3.17e-11 | -0.022074173 | 0.0352102216905501 | 0.530708313334719 |
| Ceramide (d40:1) levels | GCST90277253 | CKD | WoOryb | rs10025139 | C | T | C | T | 0.0898948 | 0.0200045 | 7.1e-06 | -0.046367383 | 0.0459194647323125 | 0.312612954130007 |
| Ceramide (d40:1) levels | GCST90277253 | CKD | WoOryb | rs11064535 | A | G | A | G | -0.158727 | 0.0359062 | 9.98e-06 | -0.056673258 | 0.0451294843371875 | 0.209191291406918 |
| Ceramide (d40:1) levels | GCST90277253 | CKD | WoOryb | rs11868222 | C | T | C | T | -0.0841618 | 0.0189682 | 9.25e-06 | -0.038632455 | 0.0489358333650632 | 0.429848310734935 |
| Ceramide (d40:1) levels | GCST90277253 | CKD | WoOryb | rs11982486 | C | T | C | T | 0.0893504 | 0.0195164 | 4.77e-06 | 0.281042071283774 | 0.269345082372049 | 0.296750333371834 |
| Ceramide (d40:1) levels | GCST90277253 | CKD | WoOryb | rs12366015 | G | A | G | A | -0.088126 | 0.0186203 | 2.25e-06 | 0.0442140570463705 | 0.0314727345871526 | 0.160069801069826 |
| Ceramide (d40:1) levels | GCST90277253 | CKD | WoOryb | rs12477218 | G | A | G | A | 0.0747848 | 0.0168687 | 9.41e-06 | -0.060839124 | 0.0324721209545542 | 0.0609883063846228 |
| Ceramide (d40:1) levels | GCST90277253 | CKD | WoOryb | rs1260326 | C | T | C | T | -0.0994691 | 0.0175128 | 1.4e-08 | 0.00417826564979557 | 0.031286311179199 | 0.893758960161603 |
| Ceramide (d40:1) levels | GCST90277253 | CKD | WoOryb | rs12927793 | A | G | A | G | -0.0791556 | 0.0166825 | 2.13e-06 | -0.081574524 | 0.0841390113004143 | 0.332285391418874 |
| Ceramide (d40:1) levels | GCST90277253 | CKD | WoOryb | rs1346910 | T | C | T | C | -0.102509 | 0.0220678 | 3.46e-06 | 0.033756035804265 | 0.0311143201871017 | 0.27796438438765 |
| Ceramide (d40:1) levels | GCST90277253 | CKD | WoOryb | rs2236124 | C | T | C | T | 0.127704 | 0.0234895 | 5.6e-08 | -0.073271134 | 0.0375692585790305 | 0.0511409797850548 |
| Ceramide (d40:1) levels | GCST90277253 | CKD | WoOryb | rs2255744 | G | A | G | A | -0.0882764 | 0.0199015 | 9.31e-06 | -0.03300322 | 0.0315918791734168 | 0.296173594529311 |

**Supplementary Note 1: Molecular Docking and Dynamics Results**

Molecular docking and molecular dynamics

To mechanistically support that Herbidospora-mediated PC(14:0_18:2) has a nephroprotective effect, we performed molecular docking between PC(14:0_18:2) and PPARγ. Conformational superposition revealed that small molecules are always located at the binding site (Supplementary Figure 6F), and the number of hydrogen bonds is maintained at 1--3 (Supplementary Figure 6I). ASP441, THR447, and ARG397 are the key hydrogen bonding residues for stable binding (Supplementary Figure 6J). Binding energy decomposition (Supplementary Figure 6G) revealed a strong and stable total binding energy (~-400 to -500 kJ/mol), which was predominantly driven by van der Waals (VDW) interactions, with minor contributions from electrostatic interactions (ELEs).

To further explore the mechanism, 100‑ns all‑atom MD simulations of the PPARγ–PC (14:0_18:2) complex were conducted. System stability was assessed through RMSD, Rg, and SASA analyses. RMSD values for PPARγ and the complex (Supplementary Figure 6A) remained stable, suggesting minimal conformational change. The ligand showed greater flexibility, likely due to its long fatty acyl chains (14:0 and 18:2) adapting within the hydrophobic pocket. Rg values (~ 2.45–2.55 nm, Supplementary Figure 6B) indicated preserved structural compactness. RMSF analysis (Supplementary Figure 6C) revealed low residue fluctuations (< 0.2 nm) with localized peaks (> 0.4 nm) near residues 320, 350, and 400—flexible loops adjacent to the LBD. The ligand–pocket distance ( 0.8–1.5 nm, Supplementary Figure 6D) and stable SASA (~ 10–15 nm², Supplementary Figure 6E) confirmed effective hydrophobic encapsulation without solvent exposure, supporting a stable PPARγ–ligand interaction.

Overall, the molecular docking and dynamics simulations demonstrate a strong and stable binding affinity between PC(14:0_18:2) and the PPARγ LBD, providing a plausible structural basis for potential modulation of PPARγ activity by this lipid metabolite.

A funnel plot of the heterogeneity test is shown in Supplementary Figure 3-Supplementary Figure 4, and there was no horizontal pleiotropy. As shown in Supplementary Tables 1- 4, MR analyses of the gut microbiota on CKD and lipids on CKD exhibited no heterogeneity, as assessed by MR‒Egger and MR-PRESSO analyses for detecting horizontal pleiotropy. Meanwhile, we applied the Bonferroni method for multiple testing correction, and set the corresponding significance p-value thresholds based on the number of taxa at different microbial taxonomic levels (phylum, class, order, family, genus, species): p < 4.545*10-3 at the phylum level, p < 2.632*10-3 at the class level, p < 2.083*10-3 at the order level, p < 8.065*10-4 at the family level, p < 3.425*10-4at the genus level, and p <2.392*10-4at the species level.[30]If the p-value is between the above significance p-value and 0.05, we consider that they have a potential causal relationship.The "leave-one-out" analysis outcomes revealed only slight variations in the error trends, implying the robustness of the MR analysis findings (Supplementary Figure 5-Supplementary Figure 6).
